# Tin—Tin π Bonding as a Conduit for Alkali‐Metal Reduction

**DOI:** 10.1002/anie.202524068

**Published:** 2025-12-21

**Authors:** Agustín Morales, Kyle G. Pearce, Louis J. Morris, Michael S. Hill, Claire L. McMullin, Emma Richards

**Affiliations:** ^1^ Department of Chemistry University of Bath Claverton Down Bath BA2 7AY UK; ^2^ Cardiff Catalysis Institute School of Chemistry Cardiff University Maindy Road Cardiff CF24 4HQ UK

**Keywords:** Density functional theory, Distannyne, Lithium, Potassium, Rubidium, Sodium

## Abstract

Reactions of the doubly reduced distannynes, [Ar′SnSnAr′M_2_], (Ar′ = C_6_H_3_‐2,6‐Dipp; M = Li, Na, K), with the successively heavier group 1 elements (M′) result in reduction of M and the isolation of [Ar′SnSnAr′M′_2_]. Although the viability of these observations, along with the reversible formation of [Ar′SnSnAr′K_2_] by treatment of [Ar′SnSnAr′Rb_2_] with potassium, is successfully predicted by a combined theoretical and thermochemical analysis, assessment of the bonding within [Ar′SnSnAr′M_2_] suggests that any M^+^ n*s* valence orbital contribution should be too high in energy to effect M^+^ reduction. Based on a consideration of the Sn─Sn π bonding and theoretical assessment of the resultant frontier orbitals, however, we suggest that the electron transfer necessary for M^+^ reduction, occurs intramolecularly and via a suitably disposed π* SOMO of the putative radical anions, [Ar′SnSnAr′M_2_]^•−^.

## Graphical abstract

Reactions of the doubly reduced distannynes, [Ar′SnSnAr′M_2_], (Ar′ = C_6_H_3_‐2,6‐Dipp; M ═ ′Li, Na, K), with the successively heavier alkali metals (M) result in reduction of M, which is proposed to occur via a suitably disposed π* SOMO of the putative radical anions, [Ar′SnSnAr′M_2_]^•−^.

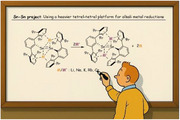

We have previously reported that the alkali metal cations (M^+^ ═ Li, Na, K, Rb) of either the chloroberyllate or alumanyl species, [(SiN^Dipp^)BeClM]_2_ (**I^M^**; (SiN^Dipp^) = (CH_2_SiMe_2_NDipp)_2_^2−^ where Dipp = 2,6‐*i*‐Pr_2_C_6_H_3_) or [(SiN^Dipp^)AlM]_2_ (**II^M^**), are prone to reduction by other elemental alkali metals (Scheme 1).^[^ ^1^, ^2^ ^]^ As well as providing a protocol for the synthesis of the otherwise inaccessible sodium alumanyl, [(SiN^Dipp^)AlNa]_2_ (**II^Na^**),^[^ ^2^, ^3^ ^]^ these observations partly contradict the group 1 reduction potentials [i.e., M_(aq)_^+^ + e^−^ → M_(s)_; where M ═ ′Li (*E* ^0^ = −3.04 V versus SHE), Na (−2.71 V), K (−2.93 V), Rb (−2.98 V), Cs (−2.92 V)].^[^ ^4^ ^]^ These commonly cited data are, however, decisively influenced by the M^+^ hydration enthalpies (Δ*H*_Hyd_) that decline incrementally as group 1 is descended (Li^+^ 506; Na^+^ 406; K^+^ 330; Rb^+^ 310; Cs^+^ 276 kJ mol^−1^).^[^ ^5^ ^]^ In contrast, the alkali metal coordination environments of **I^M^** and **II^M^** are largely provided by M^+^⋯π arene interactions with the *N*‐Dipp substituents of the (SiN^Dipp^) diamide ligands. To account for this structural feature and the heterogeneous nature of the “dissolving metal” reactions summarized in Scheme 1, we have devised a Hess’ law approach that allows thermodynamic assessment of these transformations using a combination of density functional theory (DFT) calculations and thermochemical free energies of atomization.^[^ ^1^, ^2^ ^]^ This protocol yields data consistent with experimental observation and is further validated by its successful prediction of the reversible interconversion of both **I^K^** and **I^Rb^** (ΔΔ*G* = 0.3 kcal mol^−1^) and **II^K^** and **II^Rb^** (ΔΔ*G* = 0.1 kcal mol^−1^) (Scheme 1). Adoption of the same approach has also allowed a rationalization of the narrower scope of M′/M^+^ redox reactivity displayed by crown ether derivatives of **I^M^**,^[^ ^6^ ^]^ and has prompted us to speculate that *s*‐block cations may even be prone to subtle environmental influences analogous to n*d*‐metal ligand field effects.^[^ ^2^ ^]^

**Scheme 1:**
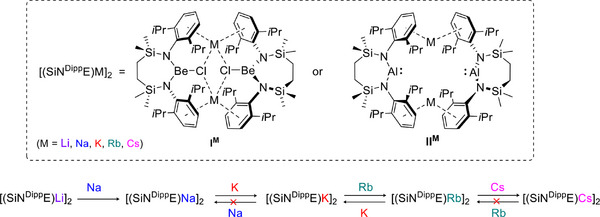
Alkali metal redox exchange within [(SiN^Dipp^)BeClM]_2_ (**I^M^**) and [(SiN^Dipp^)AlM]_2_ (**II^M^**).

A conclusion arising from these observations is that the respective [(SiN^Dipp^)BeCl]^−^ and [(SiN^Dipp^)Al]^−^ anions of **I^M^** and **II^M^** necessarily present greater redox resilience than their constituent M^+^ partners. In this regard, the resistance to reduction of the Be(II) center of **I^M^** is at least consistent with several prior attempts to effect alkali metal reduction of ligand‐supported beryllium chlorides.^[^ ^7^, ^8^, ^9^, ^10^ ^]^ In contrast, computational scrutiny of alumanyl anions has focused solely on their 3*p*‐derived frontier orbitals,^[^ ^3^, ^11^, ^12^ ^]^ and the electronic relevance of the M^+^ cations has been effectively disregarded. The tacit assumption that the n*s* valence orbitals lie to inaccessibly high energies is, thus, at odds with the viability of the chemistry summarised in Scheme 1 and provokes questions as to how and why M to M^+^ electron transfer may occur. From a broader perspective, although alkali metals comprise the most regularly deployed reductants in the synthesis of low oxidation state *p*‐, *d*‐ and *f‐*block species, the precise mode of electron transfer generally attracts little, if any, scrutiny.

Prompted by these considerations, therefore, we identified Power and co‐workers’ well characterized *meta*‐terphenyl‐stabilized distannyne, Ar′SnSnAr′, (Ar′ = C_6_H_3_‐2,6‐Dipp) and, more specifically, its further reduced derivative, [Ar′SnSnAr′K_2_] (**1^K^**), as a suitable platform for cogent investigation of group 1 redox interchange.^[^ ^13^, ^14^, ^15^, ^16^, ^17^, ^18^ ^]^ In contrast to the linear (D_∞_ *_h_*) structures of wholly carbon‐based alkynes (Figure 1a (i)), but in common with a variety of related heavier tetrel–tetrel‐bonded derivatives,^[^ ^13^ ^]^ the C(*ipso*)‐Sn─Sn‐C(*ipso*) unit of Ar′SnSnAr′, while still planar, displays a significant (C_2_ *_h_*) *trans*‐bending that is ascribed to a second order Jahn–Teller mixing of the π*_u_* and σ*, and σ and π*_g_* * orbitals (Figure 1a (ii)).^[^ ^13^, ^19^, ^20^ ^]^ The resultant loss of orbital degeneracy provides a LUMO of a*_g_* symmetry that lies in‐plane with the direction of the distortion. While this “slipped” π‐symmetric orbital is only weakly antibonding, the (b*_g_*) LUMO+1 continues to represent an unperturbed π* orbital due to its orthogonal orientation to the direction of the distortion. Of significance to the current work, the low‐lying and only weakly antibonding nature of the a*_g_* LUMO renders such distannynes susceptible to further one and two electron reduction by alkali metals without any significant perturbation to the Sn─Sn bond order. Structural studies performed by Power and co‐workers, for example, have shown that the Sn─Sn bonds of [Ar′SnSnAr′K_2_] (**1^K^**) and [Ar*SnSnAr*M_2_] [M ═ Na or K where Ar* = C_6_H_3_‐2,6‐Trip; Trip = C_6_H_2_‐2,4,6‐*i*‐Pr_3_] are only marginally elongated by ca. 0.1 Å in comparison to their unreduced analogs.^[^ ^21^, ^22^, ^23^ ^]^ The molecular encapsulation of the group 1 cations also provides a notable resemblance to the M^+^⋯π arene interactions presented by the **I^M^** and **II^M^** dimers (Scheme 1). In this study, therefore, we demonstrate that a broader scope of [Ar′SnSnAr′M_2_] (**1^M^**; M = Li, Na, K, Rb) derivatives is accessible by reaction of the already doubly reduced compounds with alternative alkali metals (M′). Furthermore, we begin to consider how the necessary M^+^/M′→M/M′^+^ redox exchange may be facilitated by the electronic accessibility of the Sn─Sn‐derived frontier π* orbitals.

**Figure 1:**
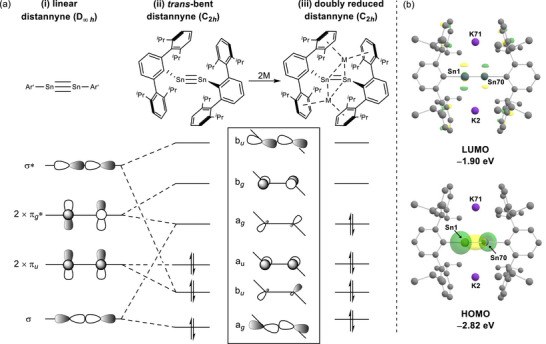
a) Schematic drawing of the second order Jahn‐Teller mixing of the σ and π*/π and σ* orbitals upon *trans*‐bending of (i) a hypothetical linear distannyne, (ii) the orbital occupancy of [Ar′SnSnAr′] and (iii) the electronic occupation of the a*_g_* LUMO of [Ar′SnSnAr′] upon twofold alkali metal reduction to [Ar′SnSnAr′M_2_]. b) Calculated Kohn–Sham orbitals [BP86/aug‐cc‐pVTZ‐PP(Sn)/def2‐TZVP(Rb&Cs)/def2‐TZVPP] and the relative energies (eV) of the HOMO and LUMO of [Ar′SnSnAr′K_2_] (**1^K^**).

Despite continued scrutiny of the Sn─Sn bonding of distannynes,^[^ ^24^, ^25^ ^]^ the electronic structures of doubly reduced dimetallynes have, to the best of our knowledge, evaded specific theoretical assessment. Our conjecture that the ordering of the orbitals shown in Figure 1a for [Ar′SnSnAr′] will be maintained in its doubly reduced form (Figure 1a (iii)) was, thus, supported by DFT calculations using Power's reported coordinates from the X‐ray analysis of **1^K^** as a starting point for optimization of the complete range of alkali metal derivatives (**1^M^**; M ═ Li, Na, K, Rb, Cs. See the Supporting Information for complete details).^[^ ^23^ ^]^ In agreement with expectation, in each case the highest energy valence electrons were calculated to reside in an a*_g_* symmetric “slipped” π* HOMO with the LUMO now represented by the unperturbed (b*_g_*) π* orbital (Figure 1b and see the Supporting Information). While the impact of alkali metal identity on these tin‐based orbitals was only marginal, the respective HOMO→LUMO gaps decline incrementally with increasing atomic weight of M, [**1^Li^** 1.07; **1^Na^** 1.03; **1^K^** 0.92; **1^Rb^** 0.90; **1^Cs^** 0.83 eV]. Further examination of the higher energy virtual canonical orbitals revealed that contributions from the M^+^ n*s* valence orbitals only become significant at considerably higher energies [i.e., **1^Li^** LUMO→LUMO+13, 1.73; **1^Na^** LUMO→LUMO+13, 1.40; **1^K^** LUMO→LUMO+19, 2.21; **1^Rb^** LUMO→LUMO+26, 2.15; **1^Cs^** LUMO→LUMO+12, 1.16 eV]. Although these latter data appear to argue against the potential for M^+^‐centered reduction, a thermodynamic assessment using the optimized **1^M^** molecular energies and the appropriate group 1 enthalpies and entropies of atomization (see the Supporting Information)^[^ ^26^ ^]^ again provided data supportive of M′/M^+^ redox behavior reminiscent of that displayed by **I^M^** and **II^M^**.

To provide a starting point for our synthetic study, a reaction was performed between Ar′SnCl and 5% (w/w) Li/LiCl^[^ ^27^ ^]^ in benzene. After filtration, red‐black single crystals of [Ar′SnSnAr′Li_2_] (**1^Li^**) were isolated, whereupon X‐ray diffraction analysis confirmed **1^Li^** as a further example of a doubly reduced distannyne (Figure 2a). With a viable sample of **1^Li^** in hand its reaction with 10% (w/w) Na/NaCl was performed and monitored by NMR spectroscopy. Although any changes in the resultant ^1^H NMR spectrum were barely perceptible beyond a marginal broadening of the signals associated with the Dipp *iso*‐propyl substituents, dark red crystals that deposited from the filtered reaction solution were confirmed to be **1^Na^** by a further single crystal X‐ray diffraction analysis (See the Supporting Information). Redissolution of **1^Na^** in benzene and treatment with potassium metal again induced minimal discernible changes in the resultant ^1^H NMR spectrum. Recourse to a further X‐ray diffraction experiment after filtration of the crude reaction and crystallization, however, confirmed the isolation of a polymorph of Power's previously reported compound (**1^K^**).* ^[^ ^23^ ^]^ Monitoring of an analogous reaction of **1^K^** with elemental rubidium again provided a ^1^H NMR spectrum consistent with the maintenance of a very similar solution structure, a deduction subsequently confirmed by solid‐state characterization of **1^Rb^** (Figure 2b). In contrast, whereas **1^K^** was reformed when **1^Rb^** was treated with potassium metal, attempted extension of this reactivity to the reduction of **1^Rb^** by elemental cesium, when monitored by ^1^H NMR spectroscopy either in benzene or toluene solvent, provided evidence for the generation of several, as yet, unidentified products (Figures S26 and S27). Discounting this final observation, however, these results confirm that the various **1^M^** display a range of alkali metal interconversion reactivity (Scheme 2) that is otherwise analogous to our previous observations of **I^M^** and **II^M^**.^[^ ^1^, ^2^, ^6^ ^]^

**Figure 2:**
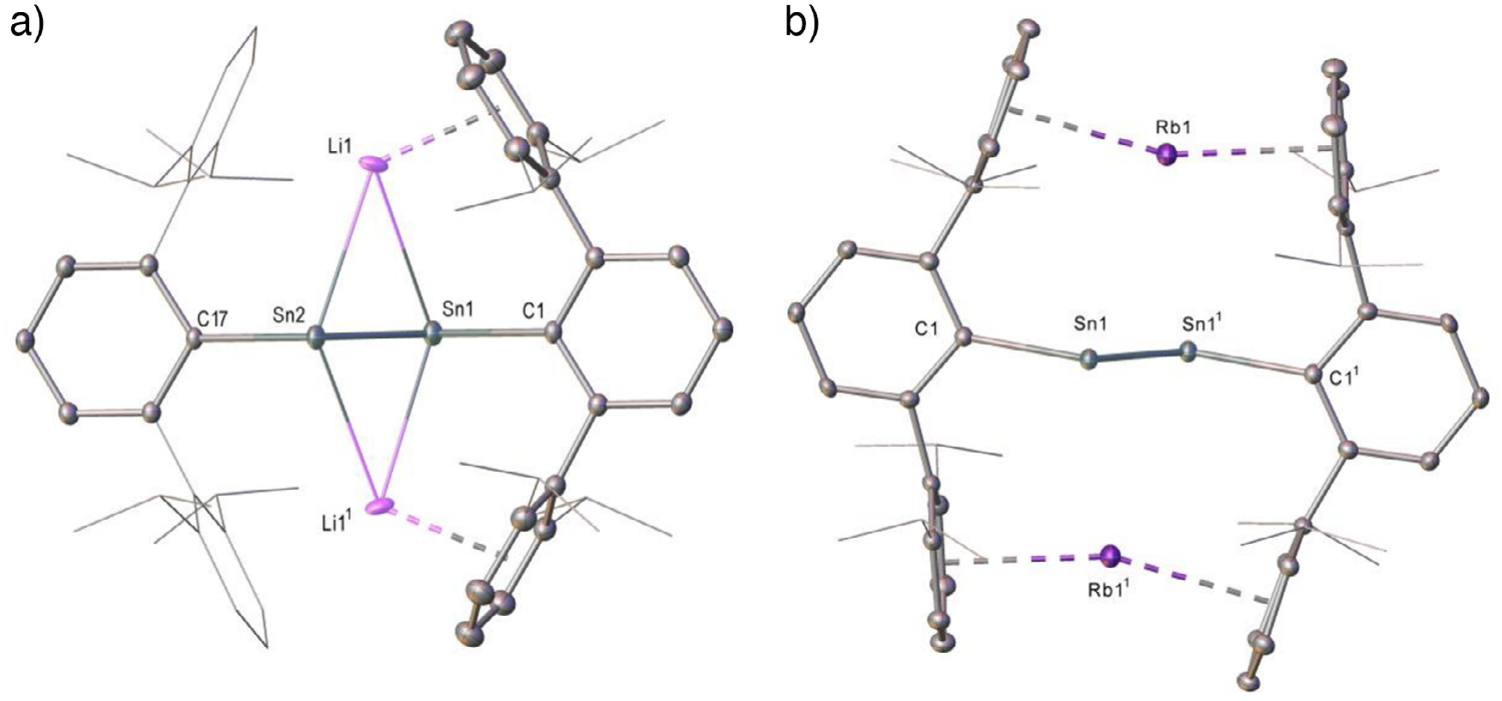
Displacement ellipsoid plots (30% probability) of a) compound **1^Li^** and b) compound **1^Rb^**. Hydrogen atoms plus disordered atoms and occluded benzene solvent have been removed for clarity. Wireframe view has been employed for some groups, also for visual ease. Selected bond lengths (Å) and angles (°): (**1^Li^**) Sn1‐Sn2 2.7682(4), Sn1‐C1 2.272(4), Sn1‐Li1 3.150(6), Sn2‐Li1 3.080(5), C1‐Sn1‐Sn2 103.39(10); C17‐Sn2‐Sn1 105.10(9)°; (**1^Rb^**) Sn1‐Sn1^1^ 2.7834(4), Sn1‐C1 2.283(3), Sn1‐Rb1 3.7787(4), Sn1‐Rb1^1^ 3.7111(4), C1‐Sn1‐Sn1^1^ 108.12(7). Symmetry operations to generate equivalent atoms: (**1^Li^**) ^1 ^+ *x*, 1/2‐*y*, +*z*; ^2^ 2‐*x*, 1‐*y*,1‐*z*; (**1^Rb^**) ^1^ 3/2‐*x*, 3/2‐*y*, 3/2‐*z*.

**Scheme 2:**
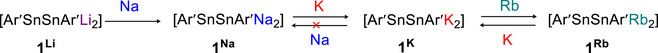
Alkali metal redox exchange within [Ar′SnSnAr′M_2_] (**1^M^**).

Consistent with precedent,^[^ ^28^, ^29^, ^30^ ^]^ the smaller size of the Li^+^ cation gives rise to a more asymmetric interaction with a single *ortho*‐Dipp substituent. Although this M^+^ encapsulation evolves into more symmetric twofold η^6^‐bonding for the larger and more diffuse K, Rb, and Cs monocations, the change alkali metal identity impacts only marginally, albeit irregularly, on the Sn─Sn bond lengths in the solid‐state structures across all four **1^M^** variants [**1^Li^** 2.7682(4); **1^Na^** 2.7839(3); **1^K^** 2.7769(3), 2.7754(3); **1^Rb^** 2.7834(4) Å]. Similarly, the various C─Sn─Sn angles across the *trans*‐bent C(*ipso*)‐Sn─Sn‐C(*ipso*) units describe no significant trend [**1^Li^** 103.39(10); **1^Na^** 105.47(8); **1^K^** 105.96(6); **1^Rb^** 108.12(7)°] beyond that which may be ascribed to the increasing M^+ ^radii.^[^ ^31^ ^]^ In an analogous manner to Power's earlier structural analysis of **1^K^**, the M^+^ cations of all four compounds are coordinated by polyhapto interactions with an *ortho*‐Dipp substituent of each terphenyl ligand and are, thus, constrained to reside on opposite sides of the C(*ipso*)‐Sn─Sn‐C(*ipso*) planes.

With the viability of **1^M^** interchange confirmed, we considered how this system may be further employed to address the mode of the electron transfer necessarily invoked during M′/M^+^ to M′^+^/M substitution. The starting point for this assessment considered the outcome of the further one electron reduction of each species. The resultant hypothetical radical anions (**1^M^** ^•−^; M ═ Li, Na, K, Rb) were, therefore, optimized at the same level of theory as **1^M^** (vide supra). Consistent with the expectation presented in Figure 1, the additional electron was in each case identified to reside in the (b*_g_*) orbital formerly ascribed as the π* antibonding LUMO of the doubly reduced distannynes (Figure 1b). While this does not induce any significant reordering of the remaining occupied or virtual orbitals, it is notable that the resultant SOMO orbitals are oriented toward the M^+^ cations with about half of the spin density (see the Supporting Information for full details) distributed equally across the two tin centers (Figure 3a). While any virtual orbitals comprising discernible M^+^ n*s* wavefunction character remain at considerably higher energy [**1^Li^** ^•−^ SOMO→LUMO+13, 1.61; **1^Na^** ^•−^ SOMO→LUMO+11, 0.99; **1^K^** ^•−^ SOMO→LUMO+8, 0.90; **1^Rb^** ^•−^ SOMO→LUMO+27, 1.97 eV], second order perturbation theory analysis of the NBO interactions estimates significant σ‐donation energies between the Sn─Sn orbitals and each M^+^ cation [Δ*E* ^(2)^: 13.0 (**1^Li^** ^•−^); 10.7 (**1^Na^** ^•−^); 9.8 (**1^K^** ^•−^); 18.6 (**1^Rb^** ^•−^) kcal mol^−1^]. We have previously characterized similar, albeit more energetically advantageous interactions (Δ*E* ^(2)^ ≈ 25 kcal mol) in [(SiN^Dipp^)MgNa]_2_, in which the Mg─Mg bonded unit and the Na^+^ cations present a topological similarity to the tin‐tin bonds and alkali metal centers of **1^M^** ^•−^ (Figure 4a).^[^ ^32^ ^]^ The heterobimetallic Mg(I) compound was also shown to spontaneously extrude the entirety of its sodium content as the elemental metal when treated with non‐reducible bases.^[^ ^33^ ^]^ This behavior was rationalized by quantum theory of atoms in molecules (QTAIM) analysis that identified a weak but significant bond critical point (BCP, *ρ*(r) = 0.0034) between the Mg─Mg σ bond and the Na^+^ cations. Although primarily ionic in nature (∇^2^ *ρ*(r) = +0.0074), the electron density of this BCP was significantly augmented (*ρ*(r) = 0.005) upon Na^+^ coordination by THF, leading us to suggest that this observation represented the trajectory of intramolecular electron transfer and initiation of Na^+^ → Na(0) reduction (Figure 4a).

**Figure 3:**
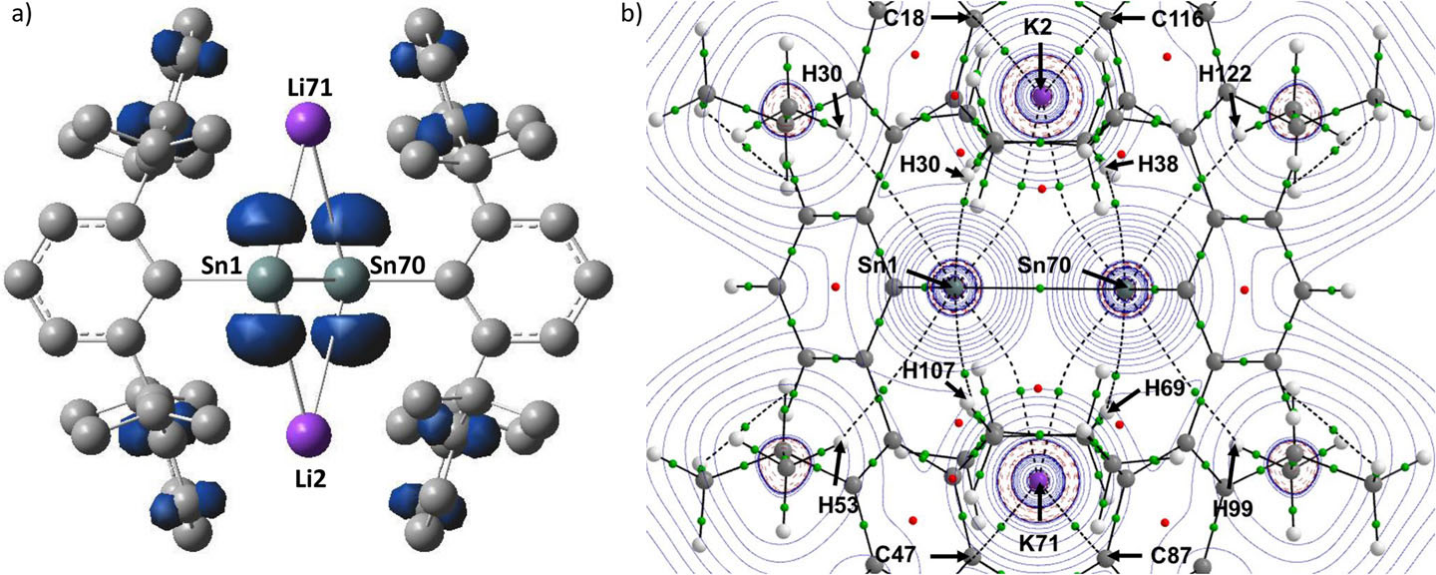
a) Spin density plot of the calculated **1^K^** ^•−^; b) QTAIM Laplacian plot of **1^K^** ^•−^. [BP86/aug‐cc‐pVTZ‐PP(Sn)/def2‐TZVPP] with BCPs identified in green and ring critical points (RCPs) in red.

**Figure 4:**
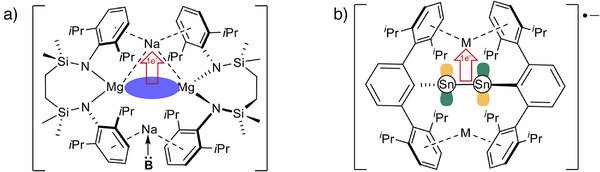
a) Base‐induced intramolecular σ‐[Mg─Mg] to Na^+^ (3*s*) electron transfer in [(SiN^Dipp^)MgNa]_2_ (B: = Lewis base); b) Proposed π* SOMO to M^+^ electron transfer in **1^M^** ^•−^.

Intrigued by the potential for analogous intramolecular electron transfer in the current systems, QTAIM topological analysis was performed on each of the doubly reduced derivatives (**1^M^**) and their radical anionic analogs (**1^M^** ^•−^). In all cases, BCPs were located between the tin atoms and the various alkali metal cations. The representative QTAIM Laplacian plot of **1^K^** ^•−^ is shown in Figure 3b and selected data relating to the BCPs located between the Sn─Sn bonds and M^+^ for all eight species are presented in Table 1.

**Table 1:** Selected QTAIM BCP data for the Sn→M^+^ bond paths in **1^M^** and **1^M^** ^•−^. [BP86/aug‐cc‐pVTZ‐PP(Sn)/def2TZV‐PP].

	**1^Li^**	**1^Li•−^**	**1^Na^**	**1^Na•−^**	**1^K^**	**1^K•−^**	**1^Rb^**	**1^Rb•−^**
**ρ(r) (a.u.^−3^)**	0.0120	0.01521	0.0116	0.0125	0.0106	0.0111	0.0103	0.0110
**∇^2^ρ(r) (a.u.^−5^)**	+0.0173	+0.0279	+0.0192	+0.0221	+0.0170	+0.0202	+0.0146	+0.0179

While the electron densities [ρ(r)] describe a decreasing trend with increasing alkali metal atomic weight, addition of a further electron ensures that the corresponding values of **1^M^** ^•−^ are higher across all four (**1^M^/1^M^** ^•−^) pairs comprising the same constituent M^+^. These *ρ*(r) values, even for the charge neutral species (**1^M^**), are notably higher than those located between the group 1 and group 2 centers in our earlier study of the spontaneous extrusion of Na(0) by [(SiN^Dipp^)MgNa]_2_. Indicative of largely ionic character, the Laplacians associated with each Sn→M^+^ BCP of **1^M^** are positive, a trend that is enhanced for each successive radical anion variant. The preference for higher hapticity π‐engagement of the heavier M^+^ homologs is also reflected by the more uniform Sn→M^+^ QTAIM properties for the heavier alkali metals, whereby polyhapto‐π encapsulation provides an increasingly stabilising structural influence.^[^ ^11^, ^34^, ^35^ ^]^ Although we can neither discount the potential significance of the Ar′ ligands in the mediation of electron transfer nor delineate the mode or chronology of cation exchange, we suggest that these data support a mode of M^+^ reduction via the Sn─Sn derived π* SOMO (Figure 4b) of the putative radical anions **1^M^** ^•−^.

To assess this hypothesis, a toluene solution of **1^Na^** was exposed in an EPR tube to a portion of freshly melted potassium metal within the resonator cavity of a CW spectrometer. Under these conditions, a broad but persistent signal (*g*_iso_ = 2.0024) was observed to develop, simulation of which identified a poorly resolved hyperfine coupling a(^117/119^Sn) of 57.49 MHz (20.5 G) (Figure S101), visible as weak satellite lines on a central broad resonance. Although this latter value is somewhat in excess of the hyperfine values reported in Power and co‐workers’ study of the isolable ditin‐based radical anion, [Sn_2_{C_6_H_3_‐2,6‐(2,4,6‐*i*‐Pr_3_C_6_H_2_)_2_}_2_]^•−^[K(THF)_6_]^+^ [a(^117^Sn) = 8.3; a(^119^Sn) = 8.5 G],^[^ ^21^, ^23^, ^36^ ^]^ it should be emphasized that these earlier data represent a SOMO resulting from single electron transfer to the a*_g_* HOMO of the neutral *trans*‐bent distannyne derivative (Figure 1a). In contrast, we tentatively suggest that the signal recorded in the current work indeed arises through single electron population of the higher energy b*_g_* π* orbital depicted in Figure 1 and the transient generation of **1^Na^** ^•−^. Although we reiterate the imprudence of further speculation about the mode of K^+^ for Na^+^ redox exchange, this conclusion was supported by computation of the hyperfine coupling for **1^Na^** ^•−^. Calculations at the ωB97X‐D4/BS6 level of theory provided a(Sn) = 58.5078 MHz, which provides a notable correlation with the observed value when converted to Gauss using the experimental g value (20.8765 G; see the Supporting Information for complete details).

With these emerging principles in hand, we are continuing to identify further systems with which to investigate and exploit the synthetic potential of such group 1 element redox exchange.

## Supporting Information

Full experimental and instrumental details, NMR spectra, details of the X‐ray analysis of compounds **1^M^** (M = Li, Na, K, Rb),^[^ ^37^ ^]^ and the methods employed in the quantum chemical investigations of this chemistry are available in the Supporting Information to this article.

## Conflict of Interests

The authors declare no conflict of interest.

## Supporting information



Supporting Information

Supporting Information

## Data Availability

The data that support the findings of this study are available in the Supporting Information of this article.

## Footnotes

* While the resultant bond lengths and angles are entirely commensurate with this earlier structure, any metric information described in the current work arises from data obtained through the newly‐performed analysis of **1^K^**.
