# Supplementary material for: Tin—Tin π Bonding as a Conduit for Alkali‐Metal Reduction
Source: Angew Chem Int Ed Engl. 2025 Dec 21;65(5):e24068. doi: 10.1002/anie.202524068 (PMC12851012; doi:10.1002/anie.202524068)
Supplement: Supplementary file 1 — Supporting Information [file ANIE-65-e24068-s001.pdf]

Synthetic Details

General Considerations

All manipulations were carried out using standard Schlenk line and glovebox techniques under an inert atmosphere of argon. NMR experiments were conducted in J-Young's NMR tubes and prepared in a glovebox. NMR spectra were recorded on a Bruker BioSpin GmbH spectrometer operating at 400.15 MHz (^{1}H) and 100.62 (^{13}C) or on an Agilent ProPulse spectrometer operating at 194.3 MHz (^{7}Li). No signals were observed by $^{119}Sn\{^{1}H\}$ NMR spectroscopy. Elemental analyses were performed by the Elemental Analysis Services Team at London Metropolitan University. Solvents were dried by passage through a commercially available solvent purification system and stored under argon in ampoules over 4 Å molecular sieves. C_6D_6 was purchased from Merck, dried over potassium, distilled and stored over molecular sieves. Li/LiCl, Na/NaCl,[1] and $[^{Dipp}TerSnCl]_2$ ($^{Dipp}Ter = C_6H_3$-2,6-(2,6-*i*-$Pr_2C_6H_3)_2$) were synthesised according to literature procedures.[2]

Synthesis of $[^{Dipp}TerSnLi]_2$ (1^{Li})

$[^{Dipp}TerSnCl]$ (30 mg, 0.05 mmol) and 5% w/w Li/LiCl (170 mg) were introduced into a J. Young's NMR tube and C_6D_6 (0.6 cm^3) was added. The reaction mixture was sonicated for 20 minutes, observing a deep green colouration, confirmed to be $^{Dipp}TerSnSn^{Dipp}Ter$ spectroscopically.[2] After a further 20 minutes a red colouration was observed, and all $^{Dipp}TerSnSn^{Dipp}Ter$ was consumed after heating at 40 °C overnight, affording a dark-red solution. The solution was filtered into a vial and dark-red (almost black) crystals of $[^{Dipp}TerSnLi]_2$ were grown from the saturated C_6D_6 solution and isolated. Yield: 12 mg, 46%.

Note: Two species were observed in the ^{1}H NMR spectrum. All attempts to crystallise the minor species resulted in same crystal structure, which could suggest these are different isomers, especially when you consider that the minor species disappears upon reduction with potassium and rubidium (*vide infra*). The same outcome is observed when performed in alternative solvents such as toluene. This reaction does also proceed with Li metal, but the hardness of the metal requires extended sonication and heating which leads to decomposition to ^{Dipp}Ter-H.

Major isomer:

^{1}H NMR (C_6D_6): δ = 7.22-6.83 (m, Ar-H, 9H), 2.90 (sept., C$\underline{H}$($CH_3)_2$, $^{3}J_{HH}$ = 6.88 Hz, 4H), 1.13 (2 overlapping d, CH(C$\underline{H}_3)_2$, $^{3}J_{HH}$ = 6.88 Hz, 24H).

$^{13}C\{^{1}H\}$ NMR (C_6D_6): δ =146.9 (^{Dipp}Ar-C), 141.1 (Ar-C), 139.7 (^{Dipp}Ar-C), 131.5 (Ar-C), 122.9 (^{Dipp}Ar-C), 30.8 ($\underline{C}$H($CH_3)_2$), 24.5 (CH($\underline{C}H_3)_2$), 24.4 (CH($\underline{C}H_3)_2$).

Minor isomer:

^{1}H NMR (C_6D_6): δ = 7.22-6.83 (m, Ar-H, 9H), 3.34 (sept., C$\underline{H}$($CH_3)_2$, $^{3}J_{HH}$ = 6.80 Hz, 4H), 1.35 (d, CH(C$\underline{H}_3)_2$, $^{3}J_{HH}$ = 6.80 Hz, 12H), 1.07 (d, CH(C$\underline{H}_3)_2$, $^{3}J_{HH}$ = 6.80 Hz, 12H).

$^{13}C\{^{1}H\}$ NMR (C_6D_6): δ = 174.4 (identified by HMBC, *i*-Ar-C), 149.1 (*o*-^{Dipp}Ar-C), 148.5 (Ar-C), 146.7 (Ar-C), 125.8 (Ar-C), 123.8 (Ar-C), 122.1 (^{Dipp}Ar-C), 31.2 ($\underline{C}H(CH_3)_2$), 26.8 ($CH(\underline{C}H_3)_2$), 23.9 ($CH(\underline{C}H_3)_2$).

Sequential Reduction of [^{Dipp}TerSnLi]$_2$ (1^{Li})

[^{Dipp}TerSnLi]$_2$ (20 mg, 0.02 mmol) was dissolved in C_6D_6 (0.6 cm^3), 10% w/w Na/NaCl was added and the reaction mixture was sonicated for 20 minutes. An essentially identical spectrum was observed by ^{1}H NMR spectroscopy but black precipitate was observed. The solution was filtered into a vial in the glovebox and allowed to slowly evaporate until crystal formation occurred, confirming the formation of **[^{Dipp}TerSnNa]$_2$** by X-ray diffraction. The crystals were redissolved in C_6D_6 and the solution was introduced into a J. Young's NMR tube and reanalysed spectroscopically looking identical to before. K metal was added to the solution and sonicated for 5 minutes, observing only one species by ^{1}H NMR spectroscopy, with a slight difference in the broadness of the Dipp-alkyl resonances relative to **[^{Dipp}TerSnNa]$_2$**. The solution was filtered into a vial and dark-red crystals were grown from solution, confirming the formation of **[^{Dipp}TerSnK]$_2$**. The crystals were redissolved in C_6D_6 and decanted into a J. Young's NMR tube and reanalysed spectroscopically, observing no change by ^{1}H NMR spectroscopy. Rb metal was added to the solution and sonicated for 3 minutes observing black ppt formation and a slight broadening in the Dipp-alkyl resonances. The solution was filtered into a vial and crystals formed on the solvent line of the solution on top of the glovebox freezer, confirming the formation of [**^{Dipp}TerSnRb]$_2$**, through X-ray diffraction. The crystals were redissolved in C_6D_6 and reanalysed by NMR, observing an identical spectrum. K metal was added and sonicated for 5 minutes in 2.5 minute intervals (ensuring temperature stays below 40 °C). Filtration into a vial in the glovebox and slow evaporation resulted in the formation of crystals of **[^{Dipp}TerSnK]$_2$**, confirmed by X-ray diffraction.

Note: Each full data crystal structure was obtained through this route.

Synthesis of [^{Dipp}TerSnNa]$_2$ (1^{Na})

[^{Dipp}TerSnCl] (20 mg, 0.036 mmol) and 10% w/w Na/NaCl (180 mg) were introduced into a J. Young's NMR tube and C_6D_6 (0.6 cm^3) was added. The reaction mixture was sonicated for 4 x 5 minutes, observing a dark red colouration and full consumption of the starting material. The solution was filtered into a vial and dark-red crystals of [^{Dipp}TerSnNa]$_2$ were deposited from the saturated C_6D_6 solution and isolated. Yield: 9 mg, 47%.

Note: Three Dipp resonances were observed in the ^{1}H NMR spectrum in a 6.5:6.5:87 ratio, the two minor species appear to be exchanging, observed by EXSY (**Figure S17**), whilst the major is not. All crystallisation attempts resulted in same crystal structure, these species could be different isomers, especially when you consider that these minor species disappear upon reduction with potassium and rubidium (*vide infra*). This reaction does work with Na metal, but the hardness of the metal requires extended sonication and heating which leads to decomposition to ^{Dipp}Ter-H.

^{1}H NMR (C_6D_6): δ = 7.32-6.84 (m, Ar-H, 9H), 2.91 (sept., $C\underline{H}(CH_3)_2$, $^3J_{HH}$ = 6.86 Hz, 4H), 1.14 (2 overlapping d, $CH(C\underline{H}_3)_2$, $^3J_{HH}$ = 6.86 Hz, 24H).

^{13}C{^{1}H} NMR (C_6D_6): δ = 171.9 (identified by HMBC, *i*-Ar-C), 146.9 (^{Dipp}Ar-C), 141.0 (Ar-C), 139.7 (Ar-C), 122.8 (Ar-C), 30.8 ($\underline{C}H(CH_3)_2$), 24.5 ($CH(\underline{C}H_3)_2$), 24.4 ($CH(\underline{C}H_3)_2$).

Synthesis of [^{Dipp}TerSnK]$_2$ (1^K)

Finely cut potassium was added to a J. Young's NMR tube containing a C_6D_6 solution of [^{Dipp}TerSnCl] (20 mg, 0.036 mmol). The solution was sonicated for 5 minutes with intermittent agitation, resulting in a green colouration. After a further 15 minutes of sonication a dark-red solution was present and only one species was observed spectroscopically. The solution was decanted away from the K metal and dark red crystals were grown from the saturated solution and isolated. Yield: 8 mg, 40%.

^{1}H NMR (C_6D_6): δ = 7.36-6.78 (m, Ar-H, 9H), 2.91 (sept., $C\underline{H}(CH_3)_2$, $^3J_{HH}$ = 6.84 Hz, 4H), 1.14 (2 overlapping d, $CH(C\underline{H}_3)_2$, $^3J_{HH}$ = 6.84 Hz, 24H).

^{13}C{^{1}H} NMR (C_6D_6): δ = 146.9 (^{Dipp}Ar-C), 141.1 (^{Dipp}Ar-C), 139.7 (Ar-C), 131.5 (Ar-C), 122.9 (^{Dipp}Ar-C), 30.8 ($\underline{C}H(CH_3)_2$), 24.5 ($CH(\underline{C}H_3)_2$), 24.4 ($CH(\underline{C}H_3)_2$).

Synthesis of [^{Dipp}TerSnRb]$_2$ (1^{Rb})

[^{Dipp}TerSnCl] (20 mg, 0.036 mmol), Rb metal and C_6D_6 (*ca.* 0.6 cm^3) were introduced into a J. Young's NMR tube. Ensuring that the temperature does not exceed 40 °C, the solution was sonicated for 24 minutes in 2 minute intervals, affording a brown solution which when settled was actually dark-red. The solution was filtered into a vial and dark-red crystals of **[LSnRb]$_2$** were grown from the saturated benzene solution and isolated. Yield: 12 mg, 55 %.

Note: This compound decomposes at temperatures above 40 °C and when stored for prolonged periods of time in solution.

^{1}H NMR (C_6D_6): δ = 7.36-6.96 (m, Ar-H, 9H), 2.91 (sept., $C\underline{H}(CH_3)_2$, $^3J_{HH}$ = 6.81 Hz, 4H), 1.13 (2 overlapping d, $CH(C\underline{H}_3)_2$, $^3J_{HH}$ = 6.81 Hz, 24H).

^{13}C{^{1}H} NMR (C_6D_6): δ = 146.9 (^{Dipp}Ar-C), 141.1 (^{Dipp}Ar-C), 139.7 (Ar-C), 131.5 (Ar-C), 122.9 (^{Dipp}Ar-C), 30.8 ($\underline{C}H(CH_3)_2$), 24.5 ($CH(\underline{C}H_3)_2$), 24.4 ($CH(\underline{C}H_3)_2$).

NMR Spectra

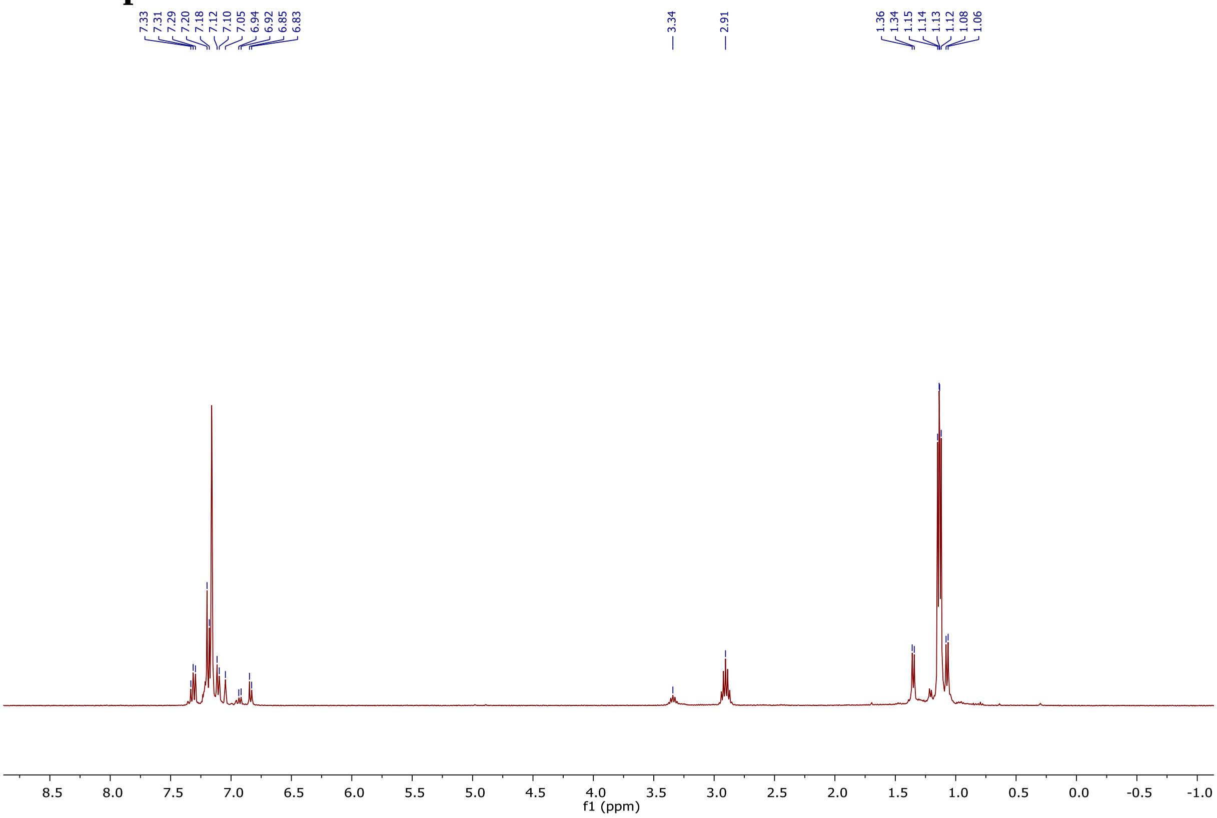

Figure S1. ^{1}H NMR Spectrum (C_6D_6, 298 K, 400.15 MHz) for **[^{Dipp}TerSnLi]$_2$ (1^{Li})**.

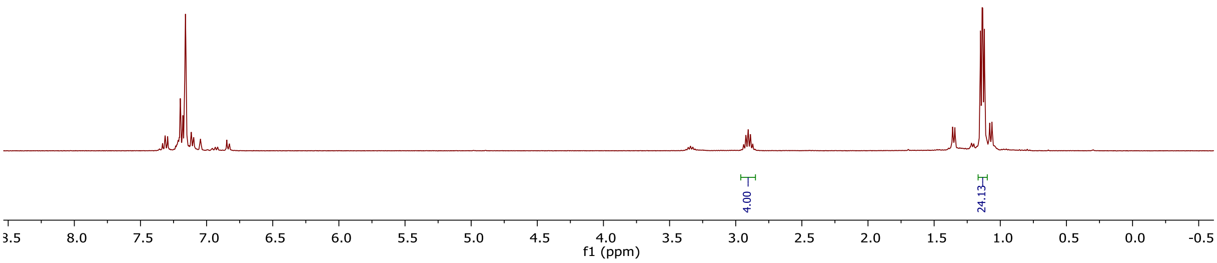

Figure S2. ^{1}H NMR Spectrum (C_6D_6, 298 K, 400.15 MHz) for **[^{Dipp}TerSnLi]$_2$ (1^{Li})**, focussed on the major species.

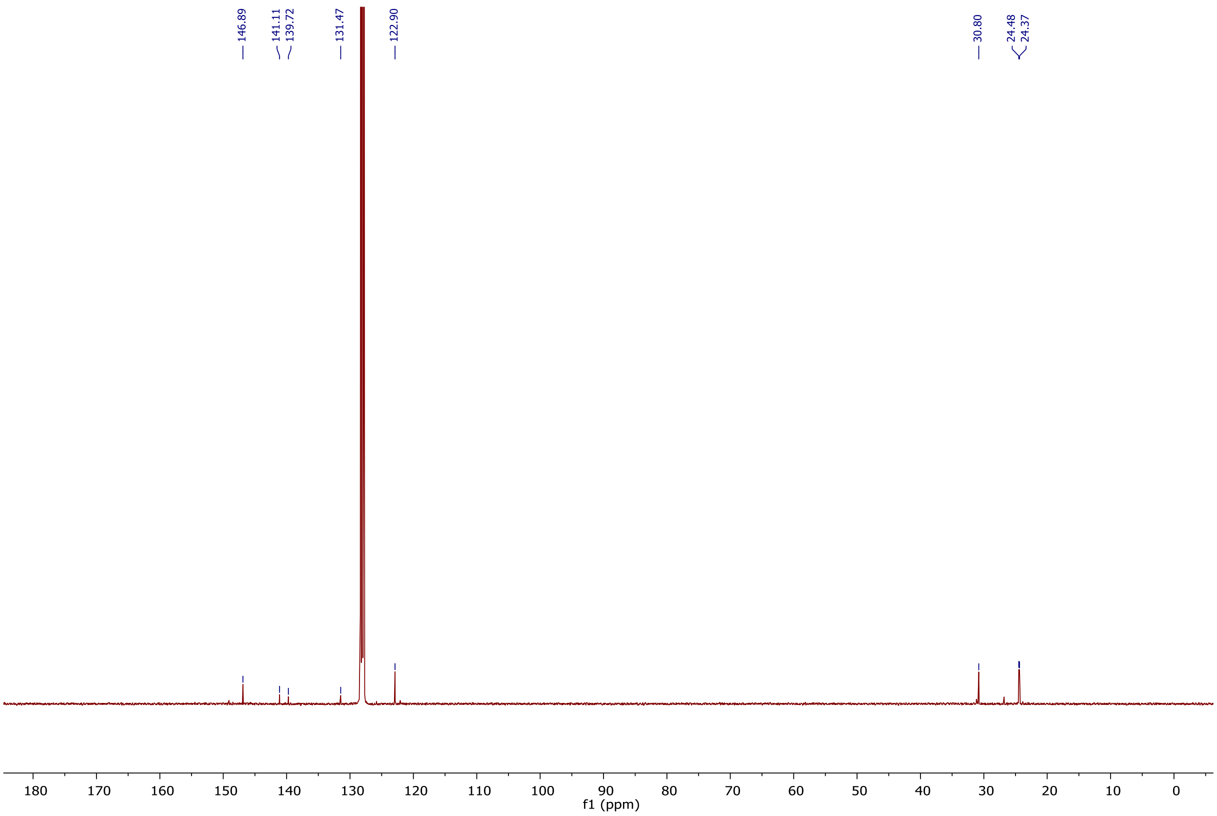

Figure S3. $^{13}C\{^{1}H\}$ NMR Spectrum (C_6D_6, 298 K, 100.62 MHz) for **[^{Dipp}TerSnLi]$_2$ (1^{Li})**, focussed on the major species.

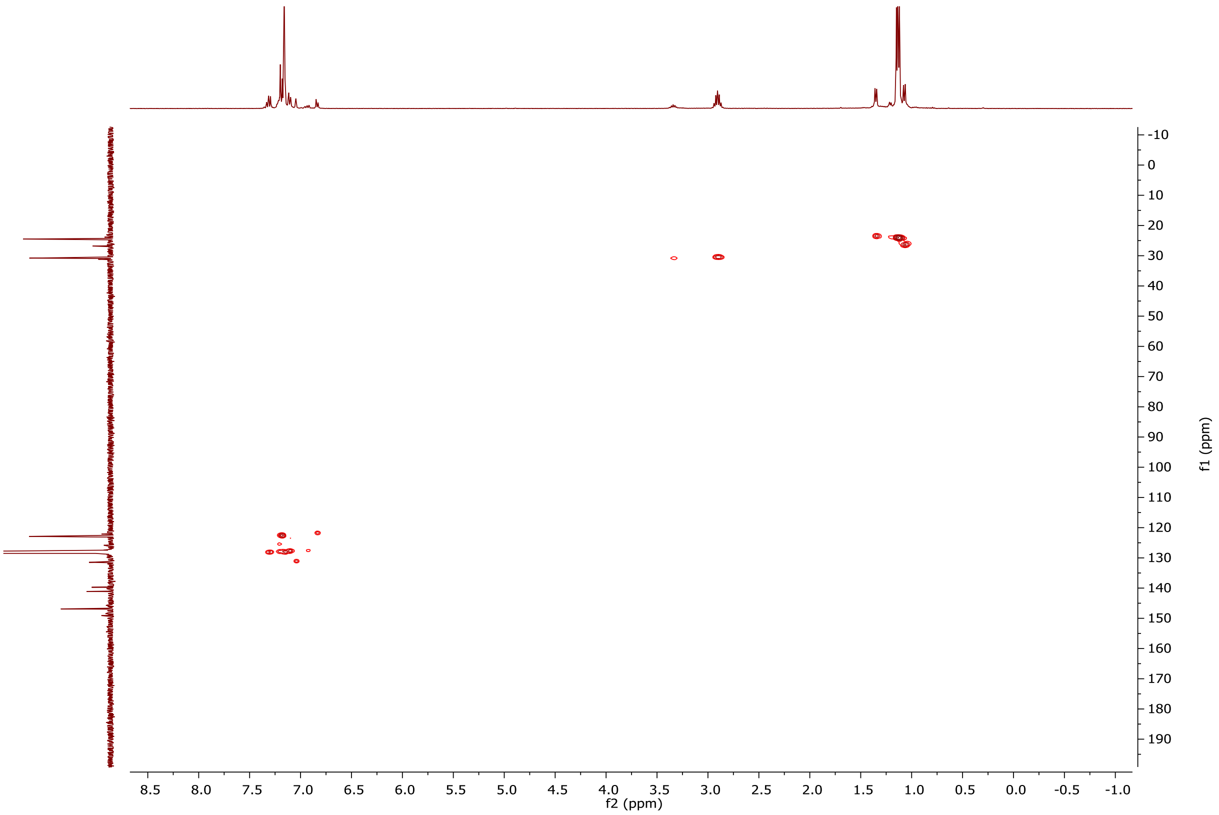

Figure S4. ^{1}H-^{13}C HSQC trace (C_6D_6, 298 K, 400.13, 100.62 MHz) for **[^{Dipp}TerSnLi]$_2$ (1^{Li})**, focussed on the major species.

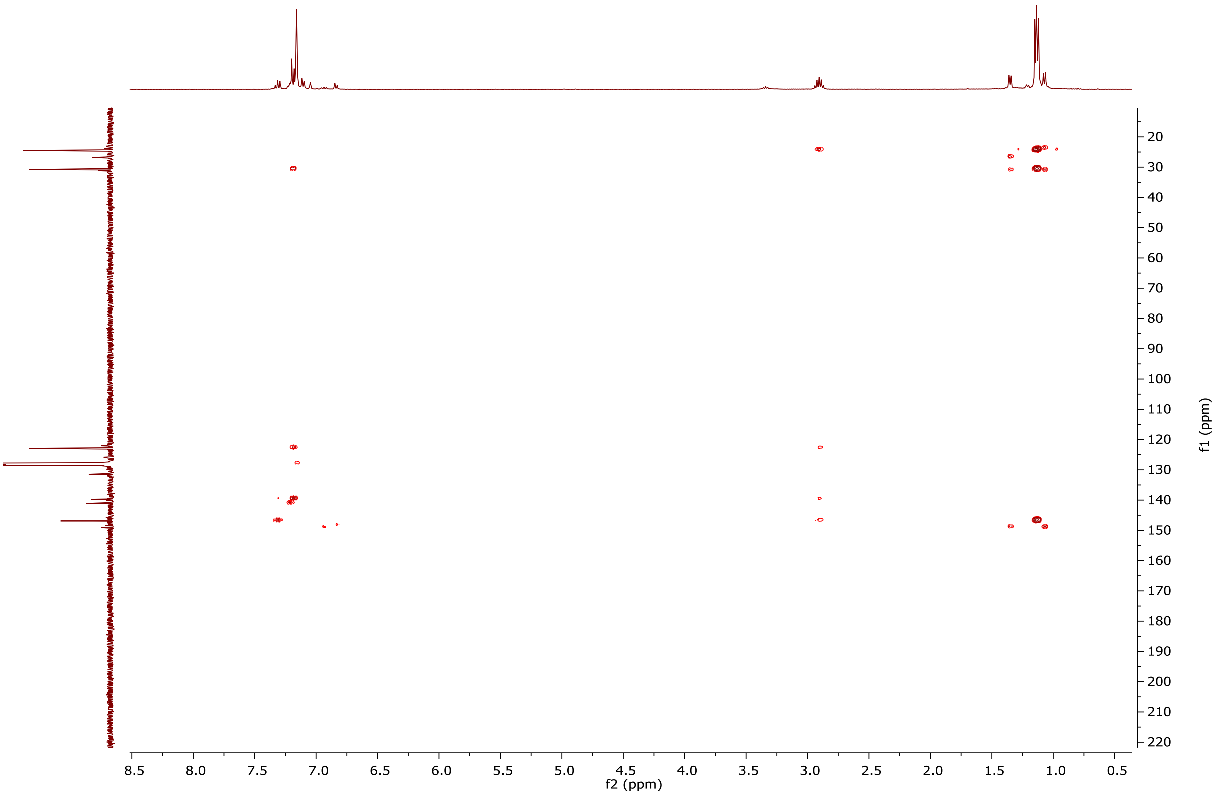

Figure S5. ^{1}H-^{13}C HMBC trace (C_6D_6, 298 K, 400.13, 100.62 MHz) for **[^{Dipp}TerSnLi]$_2$ (1^{Li})**, focussed on the major species.

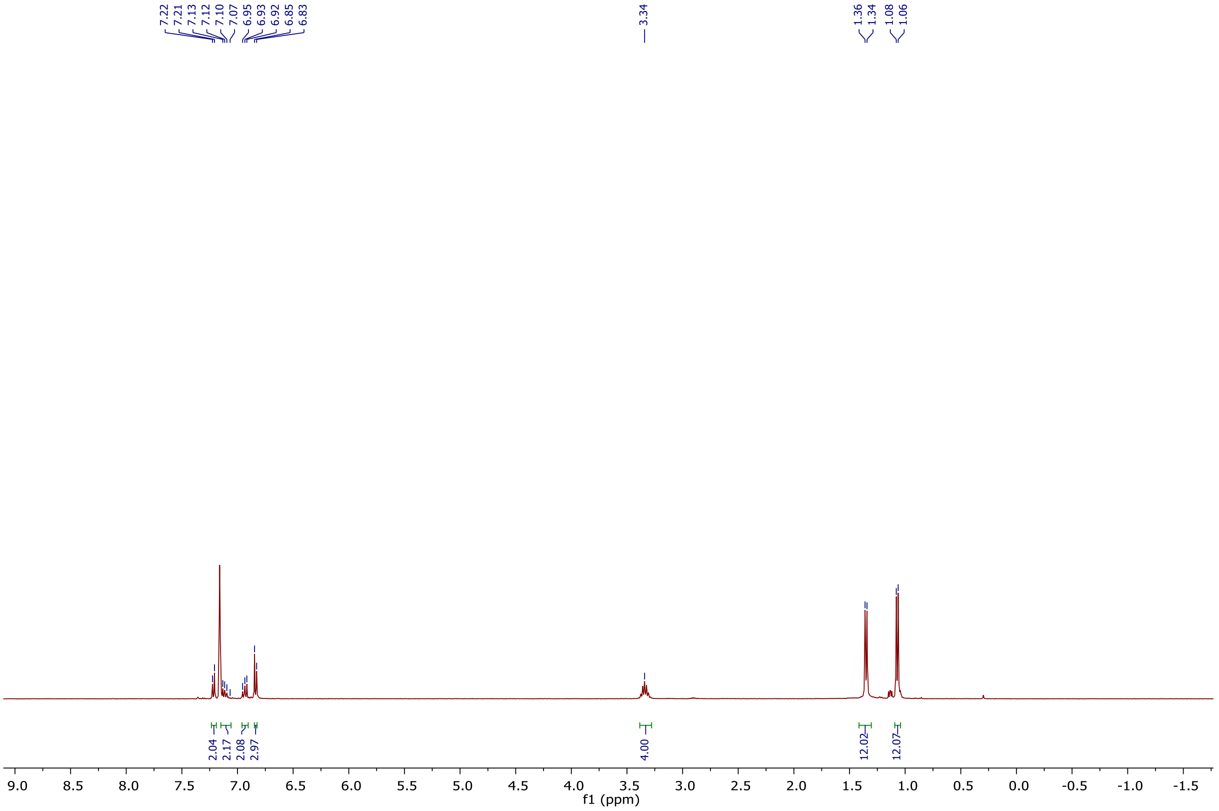

Figure S6. ^{1}H NMR Spectrum (C_6D_6, 298 K, 400.15 MHz) for **[^{Dipp}TerSnLi]$_2$ (1^{Li})**, isolated minor component.

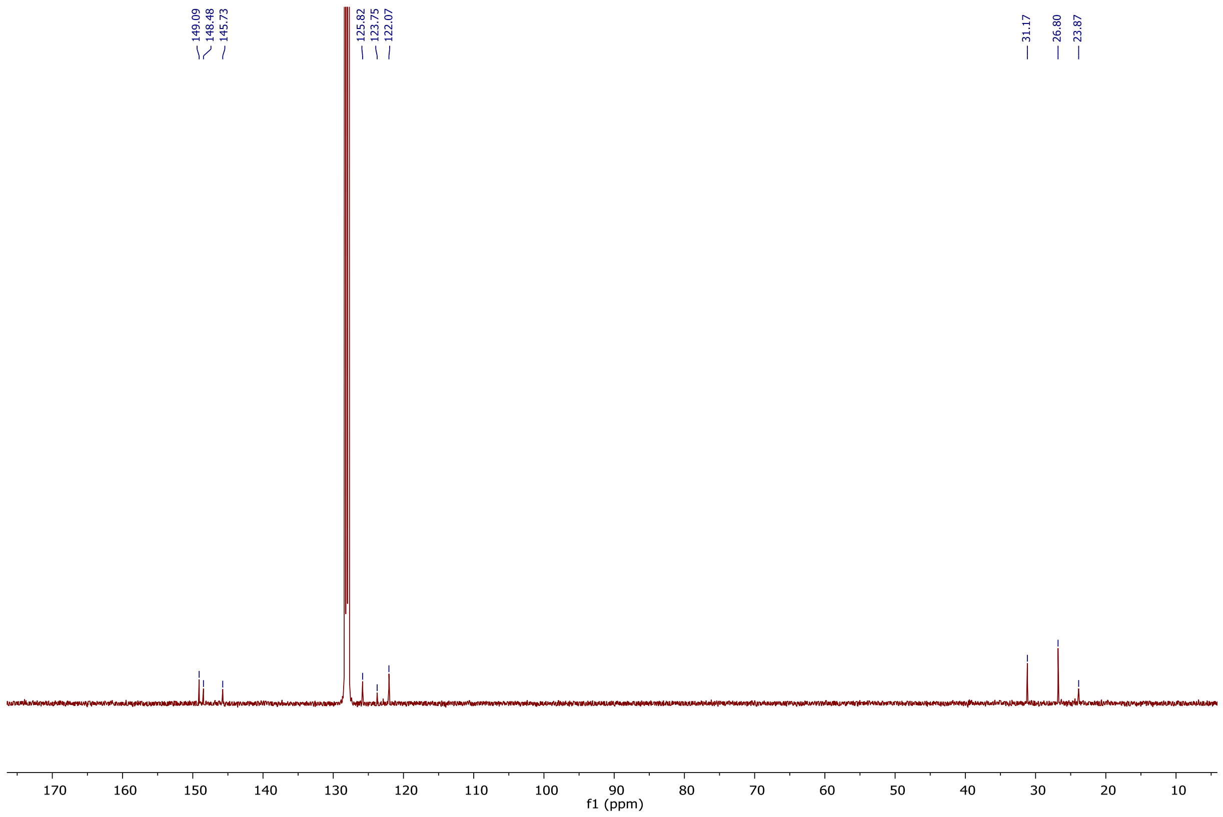

Figure S7. $^{13}C\{^{1}H\}$ NMR Spectrum (C_6D_6, 298 K, 100.62 MHz) for **[^{Dipp}TerSnLi]$_2$ (1^{Li})**, isolated minor component.

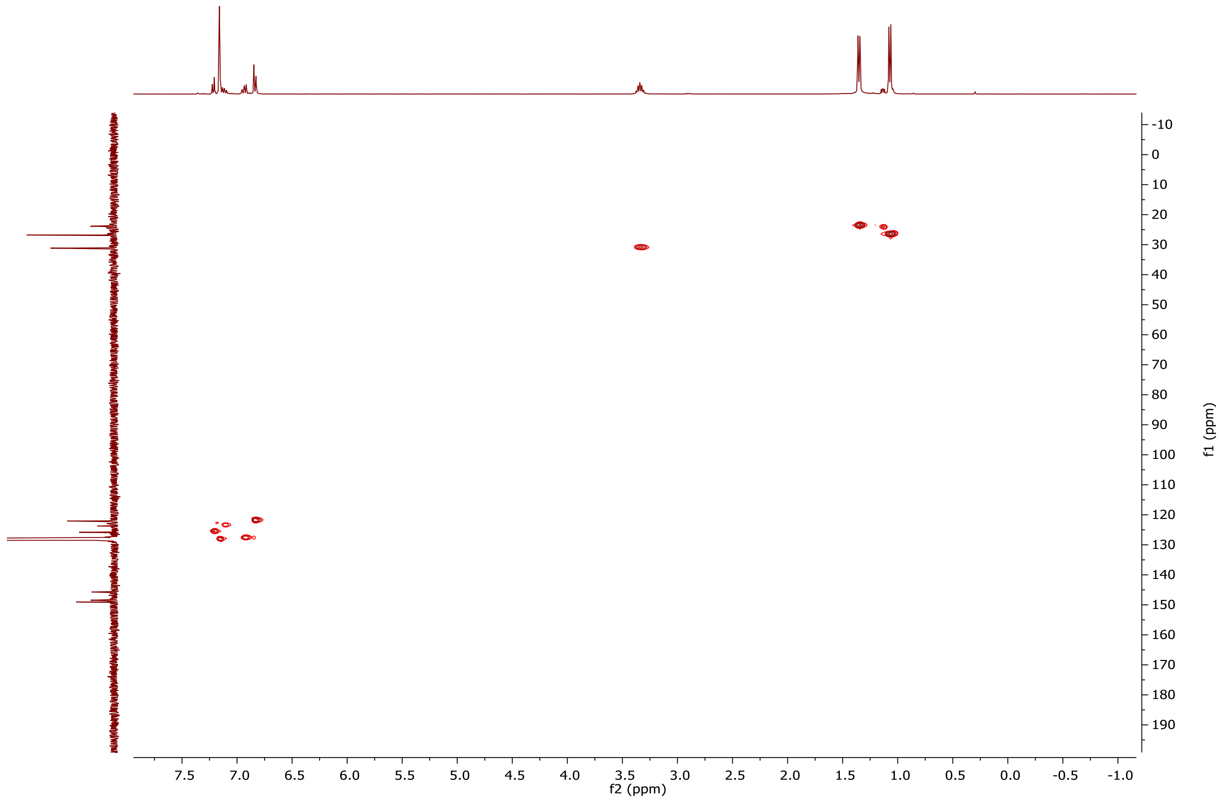

Figure S8. ^{1}H-^{13}C HSQC trace (C_6D_6, 298 K, 400.13, 100.62 MHz) for **[^{Dipp}TerSnLi]$_2$ (1^{Li})**, isolated minor component.

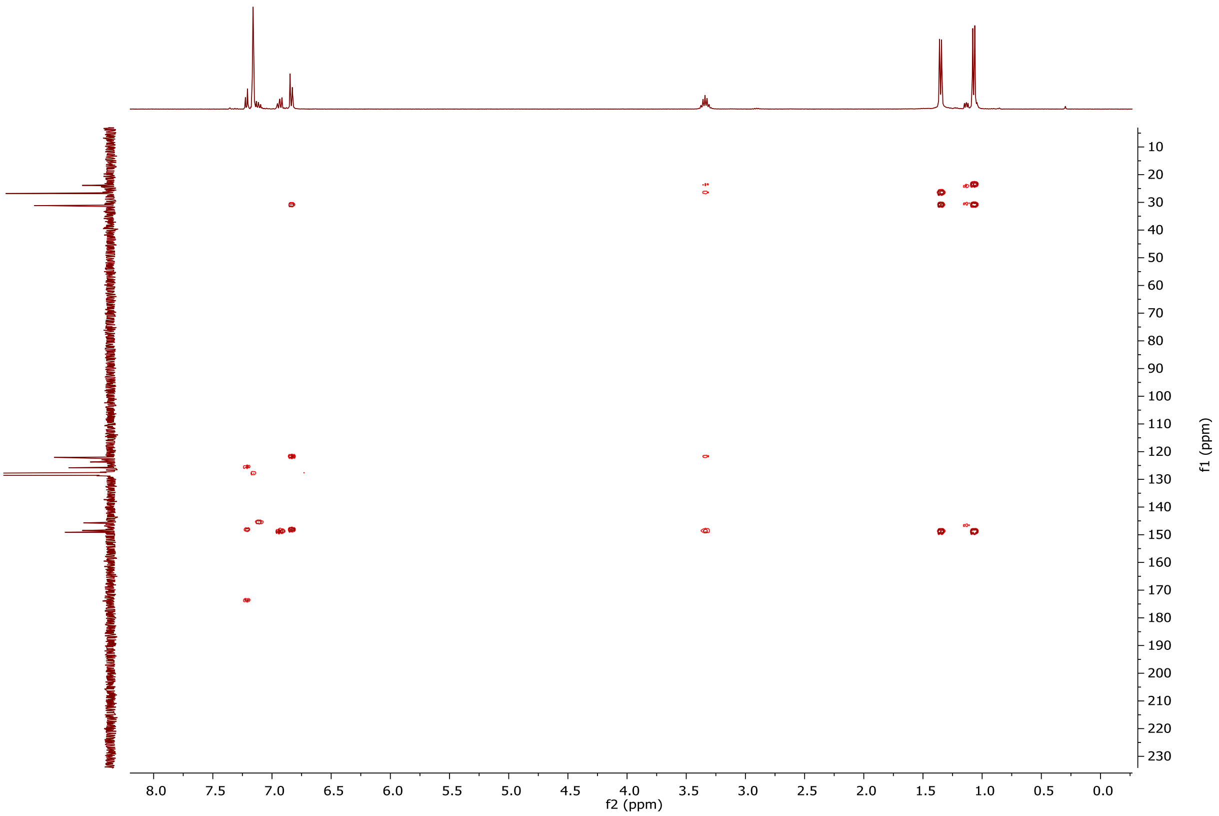

Figure S9. ^{1}H-^{13}C HMBC trace (C_6D_6, 298 K, 400.13, 100.62 MHz) for **[^{Dipp}TerSnLi]$_2$ (1^{Li})**, isolated minor component.

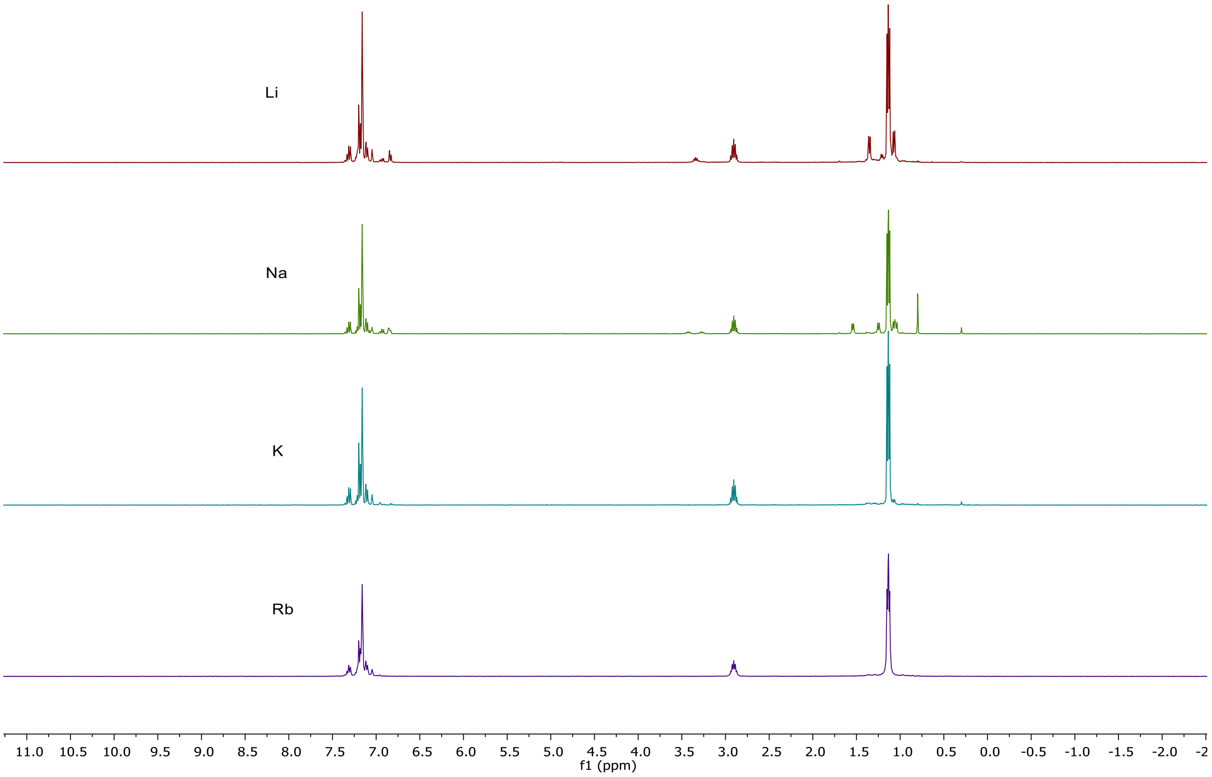

Figure S10. Overlayed ^{1}H NMR Spectra (C_6D_6, 298 K, 400.15 MHz) for the sequential reduction of **[LSnLi]$_2$**.

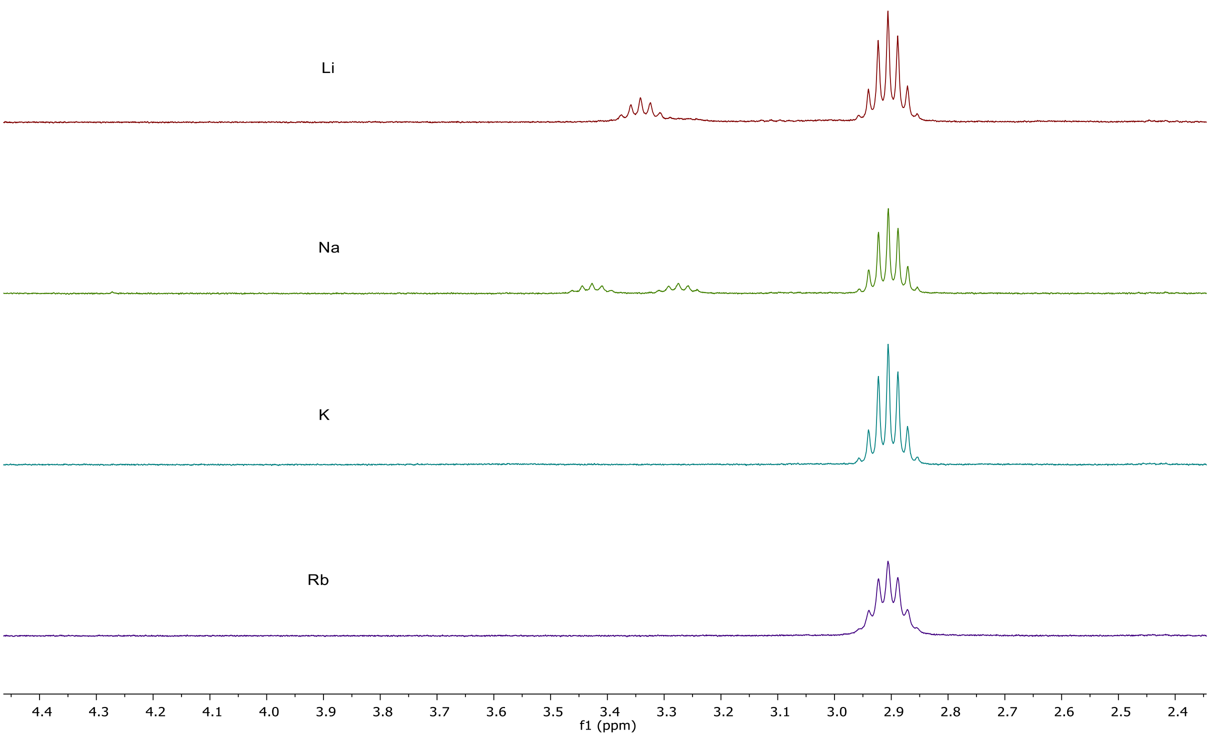

Figure S11. Expanded Dipp-methine region from the overlayed ^{1}H NMR Spectra (C_6D_6, 298 K, 400.15 MHz) of the sequential reduction of **[LSnLi]₂**.

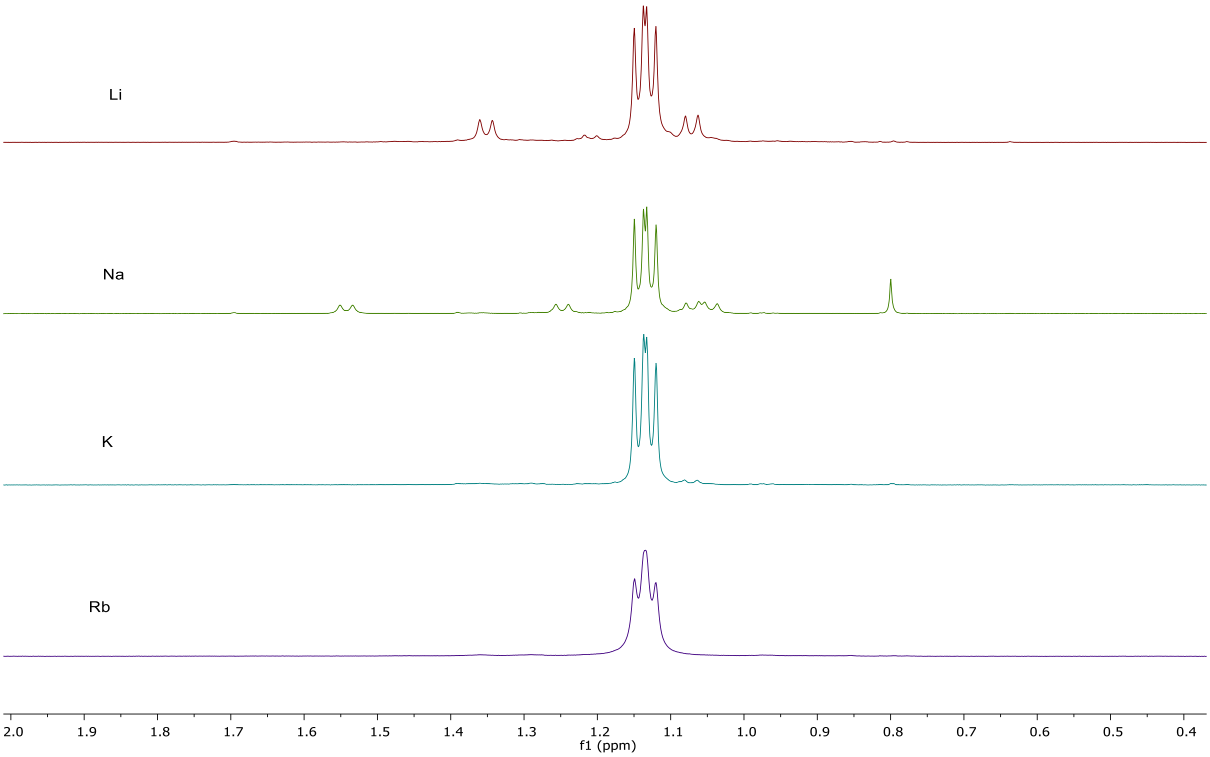

Figure S12. Expanded Dipp-alkyl region from the overlayed ^{1}H NMR Spectra (C_6D_6, 298 K, 400.15 MHz) of the sequential reduction of **$[LSnLi]_2$**.

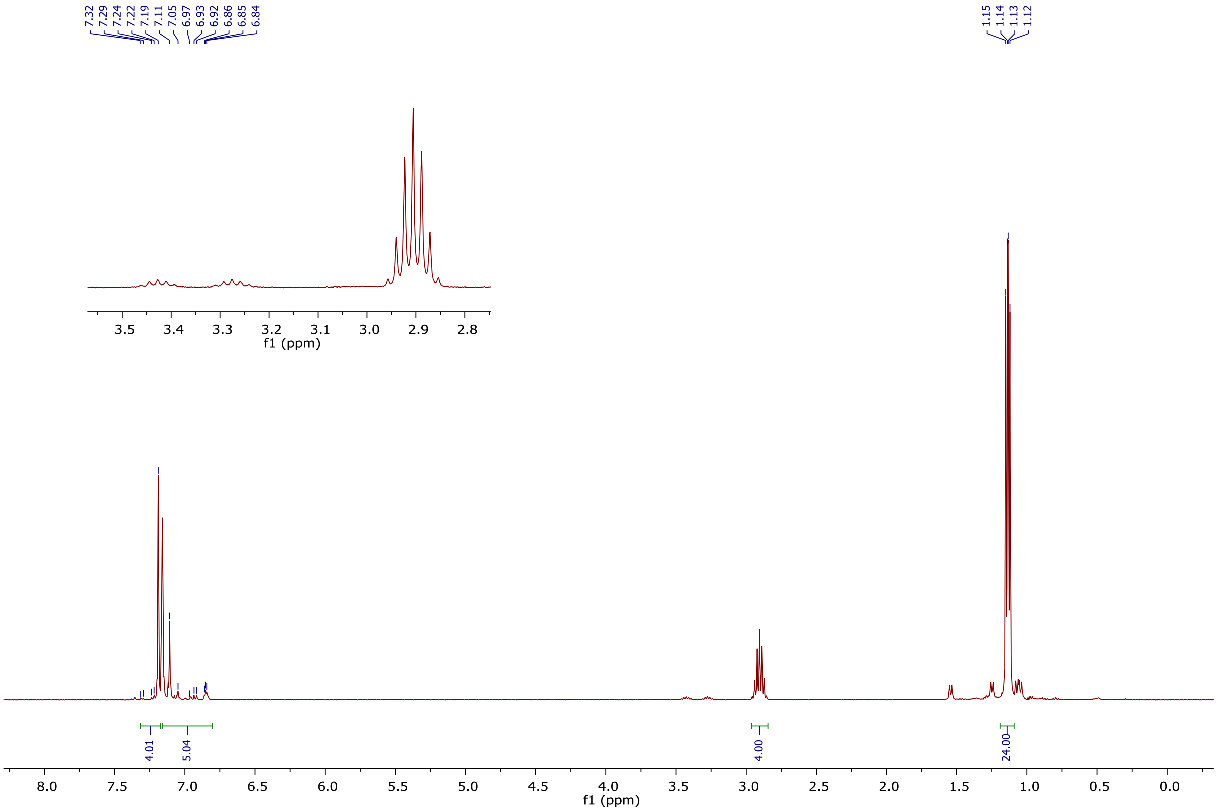

Figure S13. ^{1}H NMR Spectrum (C_6D_6, 298 K, 400.15 MHz) for **[^{Dipp}TerSnNa]$_2$ (1^{Na})**, expanded region showing minor component.

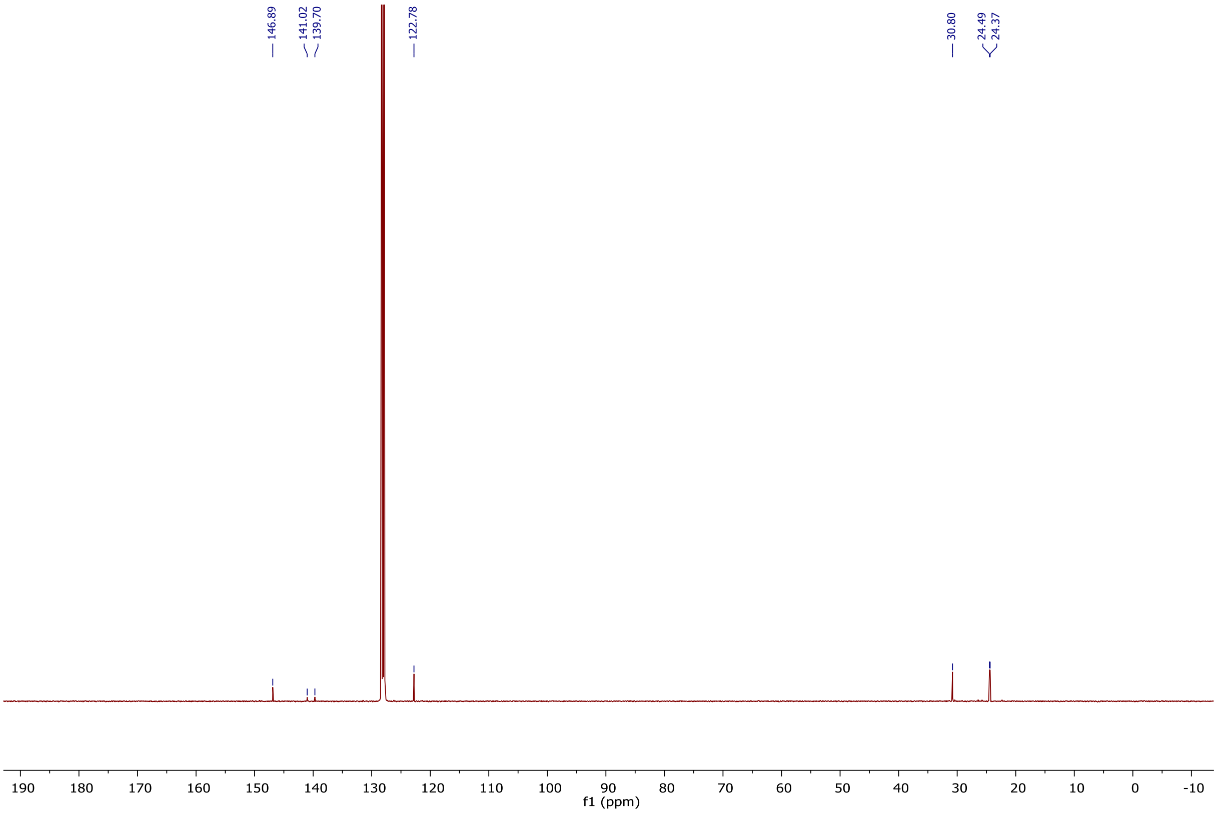

Figure S14. $^{13}C\{^{1}H\}$ NMR Spectrum (C_6D_6, 298 K, 100.62 MHz) for **[^{Dipp}TerSnNa]$_2$ (1^{Na})**.

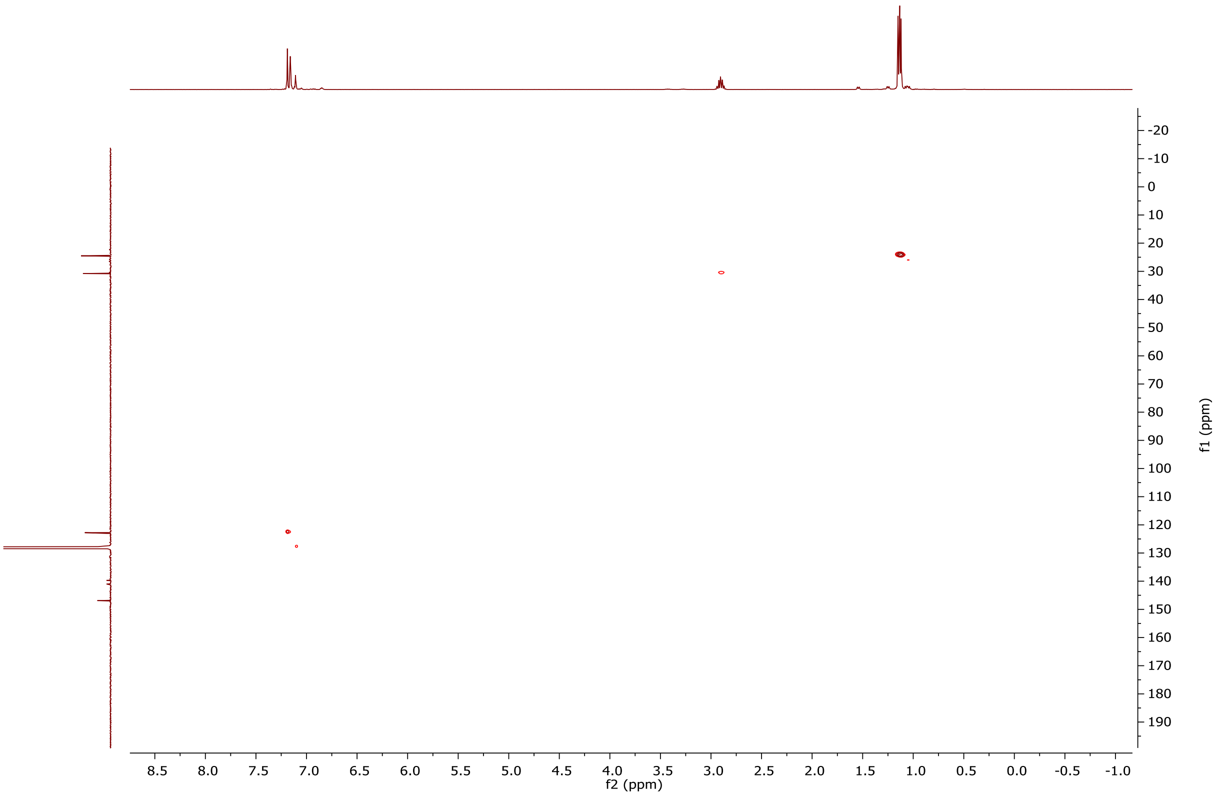

Figure S15. ^{1}H-^{13}C HSQC trace (C_6D_6, 298 K, 400.13, 100.62 MHz) for **[^{Dipp}TerSnNa]$_2$ (1^{Na})**.

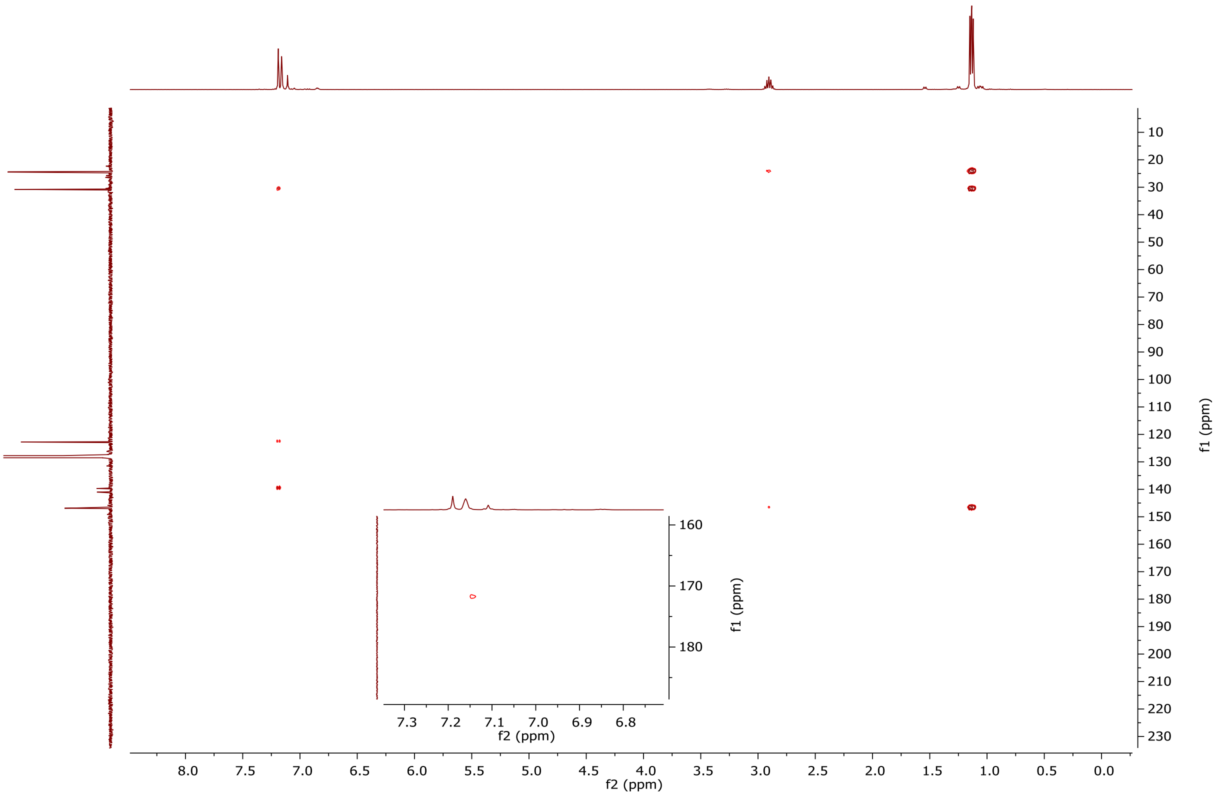

Figure S16. ^{1}H-^{13}C HMBC trace (C_6D_6, 298 K, 400.13, 100.62 MHz) for **[^{Dipp}TerSnNa]$_2$ (1^{Na})**.

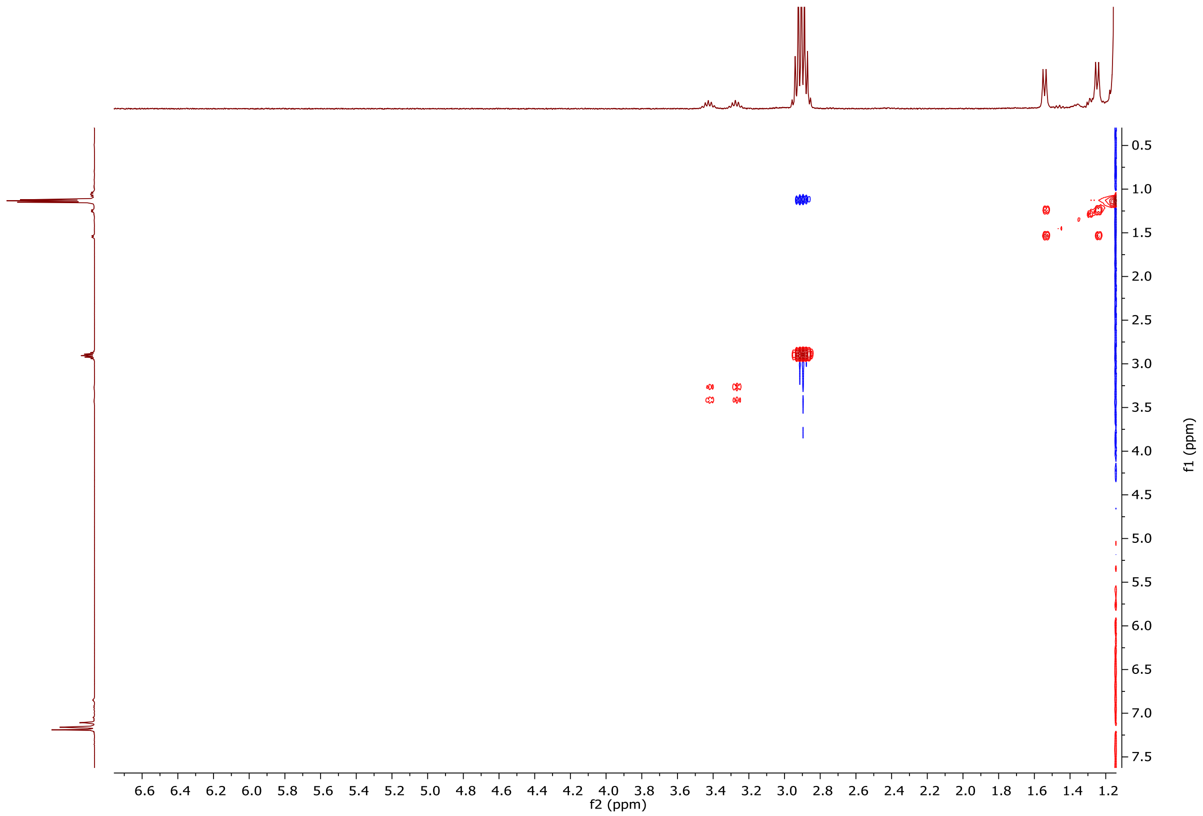

Figure S17. ^{1}H EXSY NMR Spectra (C_6D_6, 298 K, 400.13 MHz) for **[^{Dipp}TerSnNa]$_2$ (1^{Na})**, focussed on the Dipp-methine region.

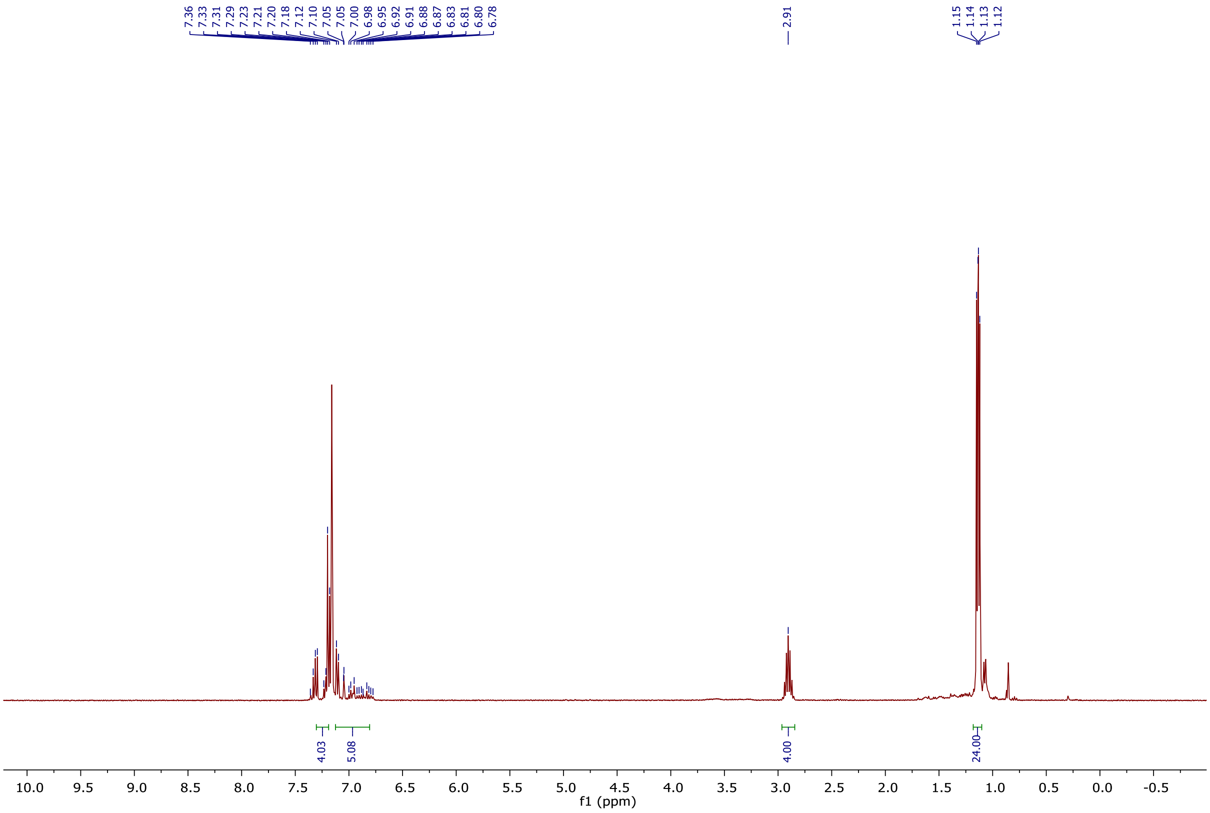

Figure S18. ^{1}H NMR Spectrum (C_6D_6, 298 K, 400.15 MHz) for **[^{Dipp}TerSnK]$_2$ (1^{K})**.

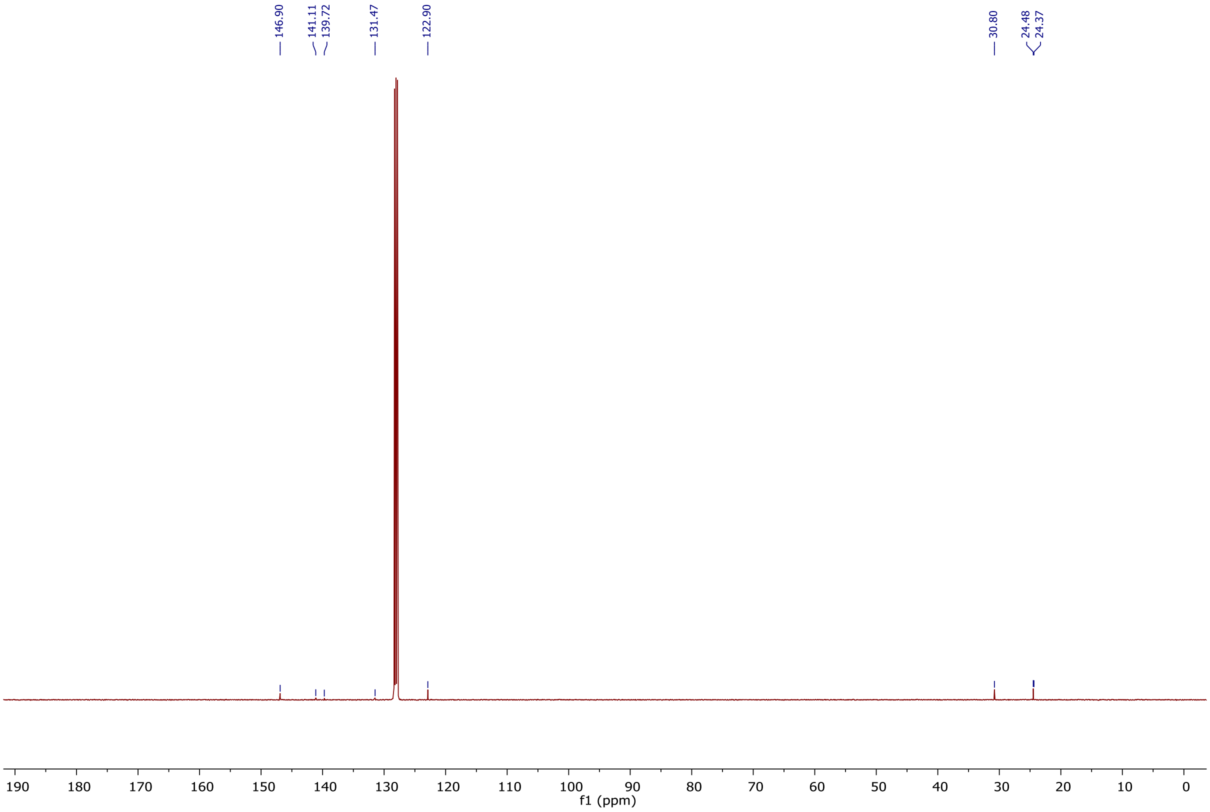

Figure S19. $^{13}C\{^{1}H\}$ NMR Spectrum (C_6D_6, 298 K, 100.62 MHz) for **[^{Dipp}TerSnK]$_2$ (1^K)**.

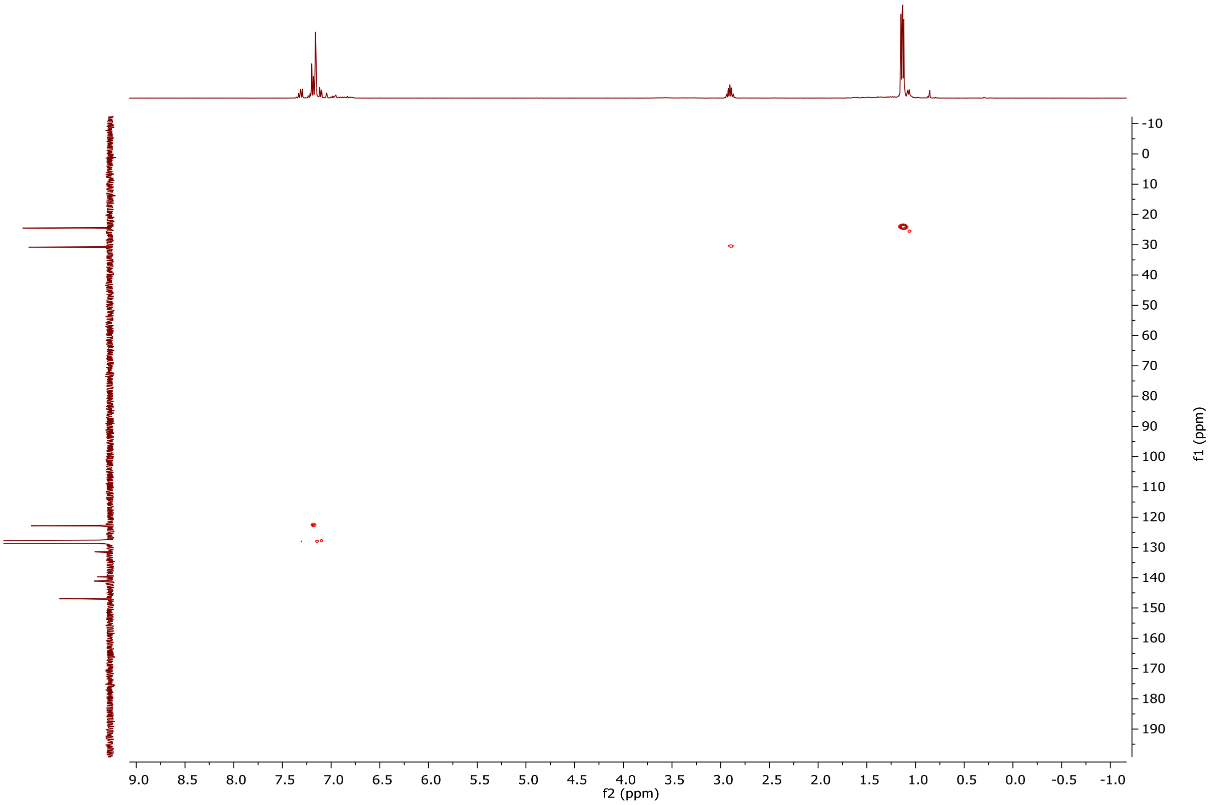

Figure S20. ^{1}H-^{13}C HSQC trace (C_6D_6, 298 K, 400.13, 100.62 MHz) for **[^{Dipp}TerSnK]$_2$ (1^K)**.

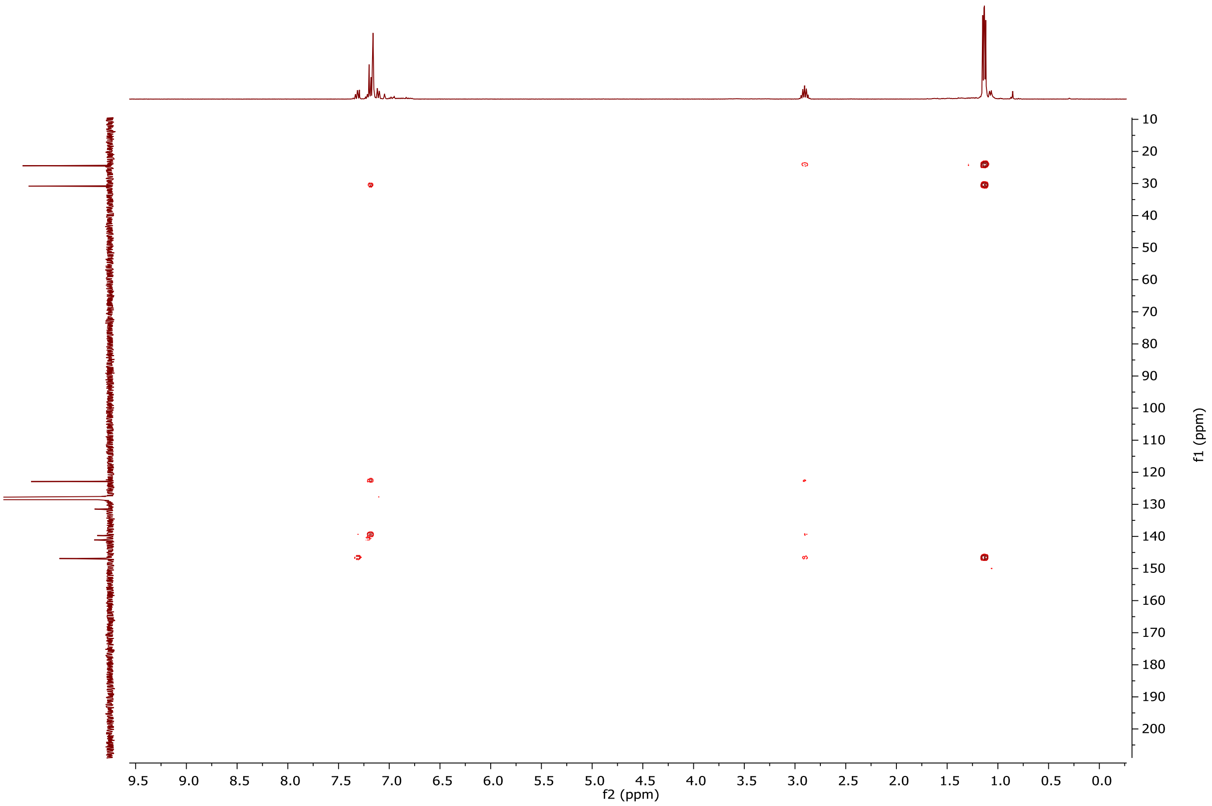

Figure S21. ^{1}H-^{13}C HMBC trace (C_6D_6, 298 K, 400.13, 100.62 MHz) for **[^{Dipp}TerSnK]$_2$ (1^{K})**.

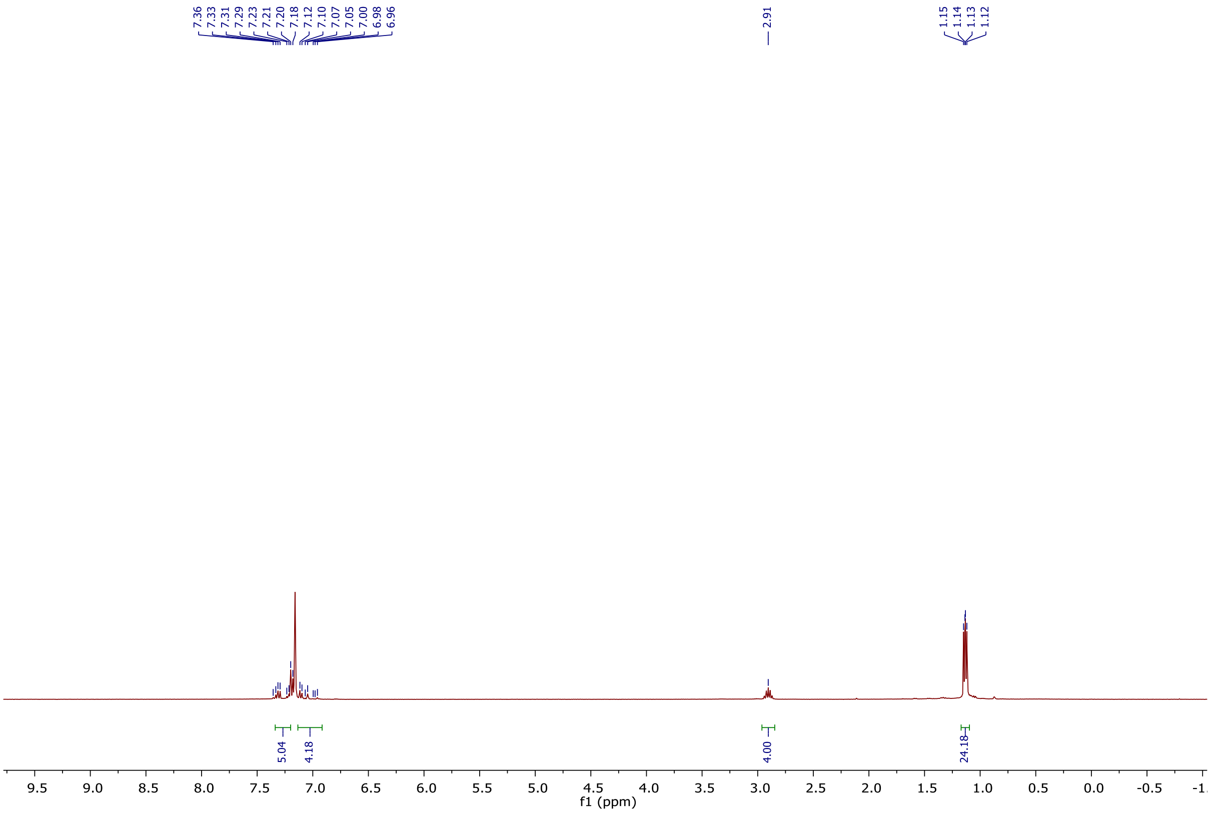

Figure S22. ^{1}H NMR Spectrum (C_6D_6, 298 K, 400.15 MHz) for **[^{Dipp}TerSnRb]$_2$ (1^{Rb})**.

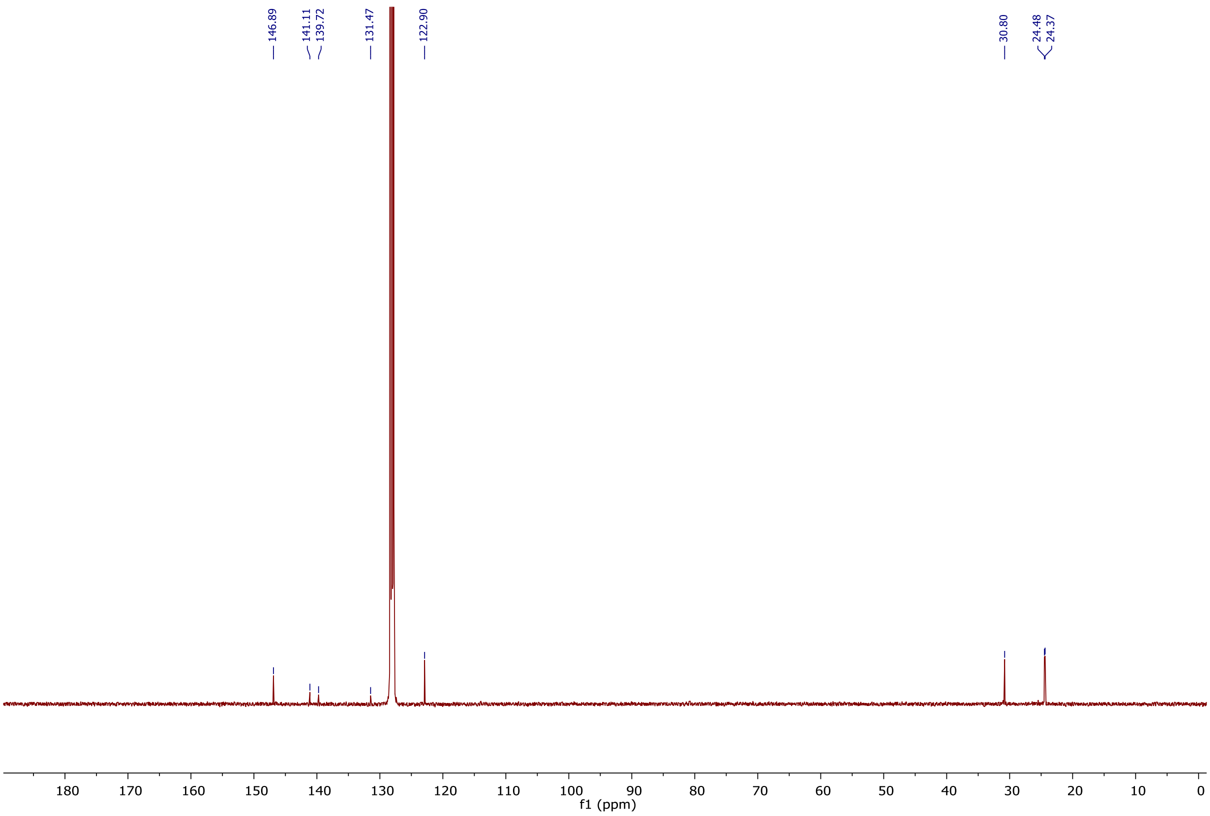

Figure S23. $^{13}C\{^{1}H\}$ NMR Spectrum (C_6D_6, 298 K, 100.62 MHz) for **[^{Dipp}TerSnRb]$_2$ (1^{Rb})**.

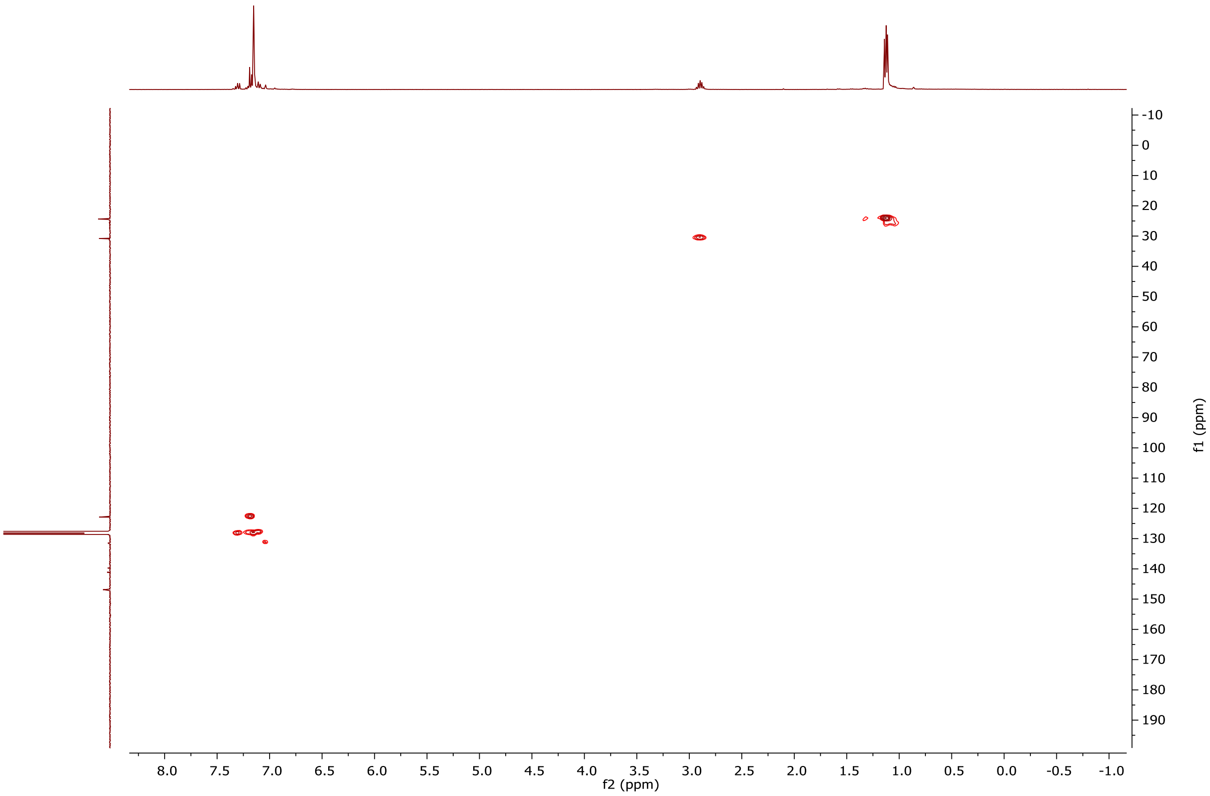

Figure S24. ^{1}H-^{13}C HSQC trace (C_6D_6, 298 K, 400.13, 100.62 MHz) for **[^{Dipp}TerSnRb]$_2$ (1^{Rb})**.

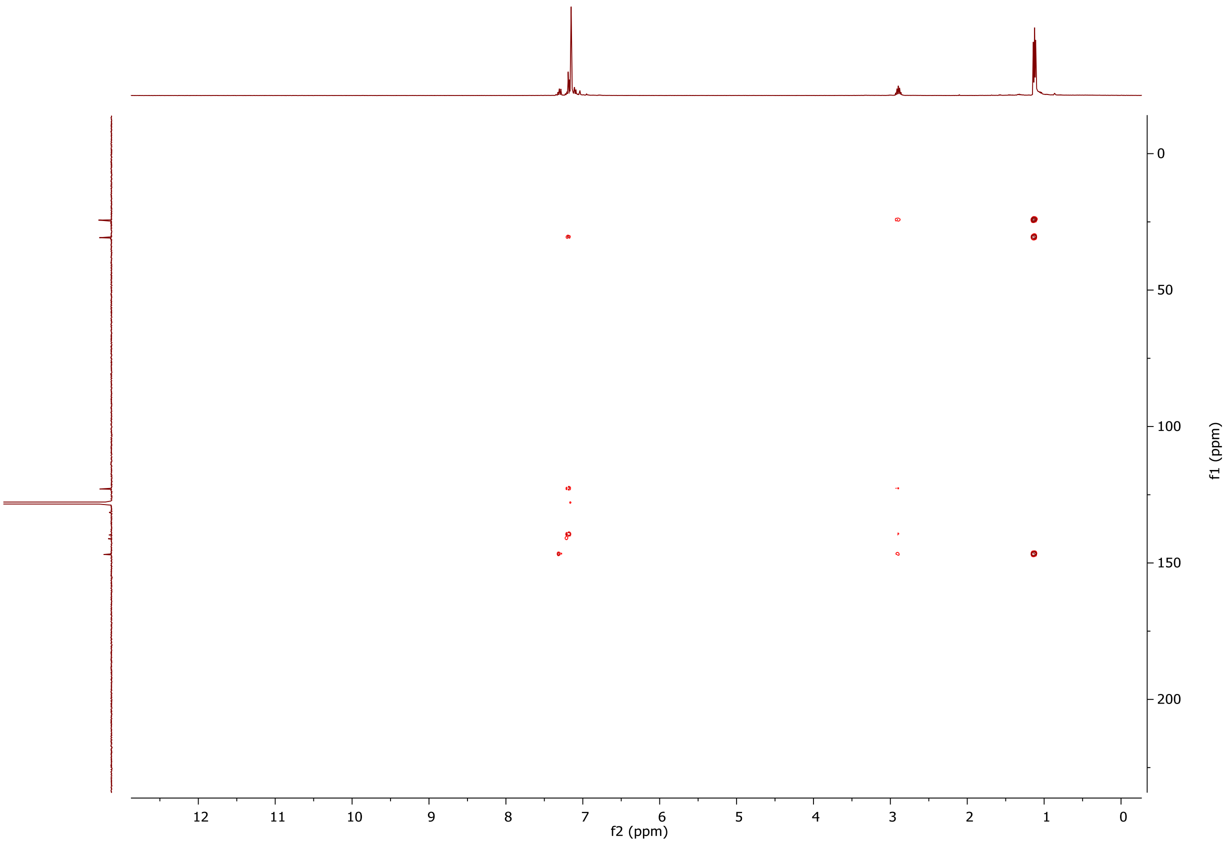

Figure S25. ^{1}H-^{13}C HMBC trace (C_6D_6, 298 K, 400.13, 100.62 MHz) for **[^{Dipp}TerSnRb]$_2$ (1^{Rb})**.

Crystallographic Details

Single Crystal X-ray diffraction data for compound $[^{Dipp}TerSnLi]_2$ (**1**$^{\mathbf{Li}}$) was collected on an Agilent SuperNova EosS2 diffractometer using Cu-Kα (1.54184 Å) radiation, whilst compounds $[^{Dipp}TerSnNa]_2$ **(1**$^{\mathbf{Na}}$**)**, $[^{Dipp}TerSnK]_2$ (**1**$^{\mathbf{K}}$) and $[^{Dipp}TerSnRb]_2$ (**1**$^{\mathbf{Rb}}$) were collected on XtaLAB Synergy, Dualflex, HyPix-Arc 100 diffractometer using Mo-Kα radiation (λ = 0.71073 Å), Cu-Kα (1.54184 Å) radiation and Mo-Kα radiation (λ = 0.71073 Å), respectively. In each case, the crystals were maintained at 150 K during data collection. Using Olex2,[3] the structures were solved with the olex2.solve[4] structure solution program or ShelXT and refined with the ShelXL[5] refinement package using Least−Squares minimisation.

The asymmetric unit for **1**$^{\mathbf{Li}}$ is comprised of half of a dimeric unit, plus two and a half benzene molecules. All moieties are completed by virtue of space-group inversion symmetry. Distance and ADP restraints were employed, on merit, in the disordered solvent regions to assist convergence.

In this series of compounds, it is notable that the rubidium (**1**$^{\mathbf{Rb}}$) and potassium (**1**$^{\mathbf{K}}$) congeners are isostructural. The asymmetric unit in each case comprises half of a dimer (with some minor disorder in the tin position) plus one molecule of benzene solvent. Both compounds crystallise in space group *I*2/*a* with comparable cell parameters when one allows for the difference of alkali metal in conjunction with packing effects.

In contrast, the sodium analogue (**1**$^{\mathbf{Na}}$) was seen to crystallise in an orthorhombic space group (*Pnma*) with an asymmetric unit that also includes half of a dimer molecule, in this instance accompanied by an additional *two* molecules of benzene in the asymmetric unit relative to the rubidium (**1**$^{\mathbf{Rb}}$) and potassium (**1**$^{\mathbf{K}}$) compounds. The solvent manifested as one complete benzene moiety, half of a benzene (located proximate to a crystallographic inversion centre) and one full benzene with half site-occupancy (located on a crystallographic mirror plane) and which exhibited disorder. Atoms Sn1, Sn2, C1, C4 C17 and C20 of the main feature in **1**$^{\mathbf{Na}}$ also lie on the same mirror as the latter solvent moiety and this serves to complete the molecule. Ultimately, the disordered benzene was treated using the solvent mask available in Olex-2, as efforts to model same, otherwise, failed. However, the main problem with the initial refinement was the puzzling presence of electron-density maxima of 5.85 and 5.83 e Å^{-3}, both located on the space-group mirror, at a distance of 2.787 Å from each other.

Somewhat puzzled, several data collections were then effected for crystals from the **1**$^{\mathbf{Na}}$ batch, over fears that the initial crystal may have lacked 'Rolls-Royce' quality. The detector distance was varied in three of these collections [39, 45 and 74 mm from sample] which spread out the diffraction spots in the raw frames, thereby minimising the impact of some spot streaking on the integrated backgrounds. In all cases, the final refinements emerged as presented here, with the same two, high, residual maxima. Scrutiny of the raw data revealed zero evidence of any twinning. Finally, the data were integrated in

space group *P*1, in order to ascertain if the residual, unassigned, electron-density might arise from the possible imposition of incorrect space group symmetry. The resultant refinement in *P*1 served only to confirm that *Pnma* is the optimal space group for the sodium congener.

The distance between the residual maxima (2.787 Å) in $\mathbf{1^{Na}}$ was noted to be very similar to the Sn1-Sn2 distance in this compound (2.7837(5)Å). The prevalence of some minor disorder of the tin positions in the subsequently determined structures of $\mathbf{1^{Rb}}$ and $\mathbf{1^{K}}$ caused us to revisit $\mathbf{1^{Na}}$ with fresh eyes, on the basis that the unassigned electron density therein could represent the tin core in a very minor disordered constituent within the crystal which (unlike in the rubidium and potassium cases) does *not* share ligand positions with the major component.

On this basis, the model presented herein includes the assignment of the contentious electron-density maxima as Sn1a/Sn2a, each with a site-occupancy of 5.5% (relative to the formula of one complete dimer based on two full tin atoms) and located co-incident with the crystallographic mirror plane that bisects the major component of the dimer. Clearly, it was not possible to credibly locate any associated light atoms with an occupancy of 5.5% using data derived from X-rays. The level of the second component corresponds with approximately 6% of a by-product that was spectroscopically detected.

Table S1. Crystal Data and Structure Refinement for Compounds **1**$^{\mathbf{M}}$ (M = Li, Na, K, Rb).

Identification code	s25msh01 (**1**$^{\mathbf{Li}}$)	y25msh27 (**1**$^{\mathbf{Na}}$)	y25msh31 (**1**$^{\mathbf{K}}$)	y25msh07 (**1**$^{\mathbf{Rb}}$)
Empirical formula	$C_{84}H_{98}Li_2Sn_2$	$C_{84}H_{98}Na_2Sn_2$	$C_{78}H_{92}K_2Sn_2$	$C_{78}H_{92}Rb_2Sn_2$
Formula weight	1358.88	1390.98	1345.09	1437.83
Crystal system	orthorhombic	orthorhombic	monoclinic	monoclinic
Space group	*Pnma*	*Pnma*	*I*2/*a*	*I*2/*a*
a/ Å	16.0753(2)	16.1037(3)	15.15510(10)	15.0380(4)
b/ Å	26.2283(3)	26.4128(6)	18.79650(10)	18.9837(4)
c/ Å	16.8962(2)	16.9079(4)	25.1316(2)	25.0499(6)
α/ °	90	90	90	90
β/ °	90	90	102.4710(10)	101.677(3)
γ/ °	90	90	90	90
U/ Å^3	7123.91(15)	7191.7(3)	6990.14(9)	7003.2(3)
Z	4	4	4	4
ρ_{calc}/ g cm^{-3}	1.267	1.285	1.278	1.364
μ/ mm^{-1}	5.886	0.750	7.043	2.135
F(000)	2832.0	2896.0	2792.0	2936.0
Crystal size/ mm^3	0.09 × 0.07 × 0.03	0.16 × 0.09 × 0.02	0.14 × 0.1 × 0.08	0.09 × 0.07 × 0.04
2θ range for data collection/°	7.59 to 146.898	4.66 to 63.522	5.922 to 160.752	4.424 to 63.41
Index ranges	$-19 \le h \le 18$, $-32 \le k \le 32$, $-19 \le l \le 20$	$-21 \le h \le 23$, $-35 \le k \le 33$, $-19 \le l \le 23$	$-17 \le h \le 19$, $-23 \le k \le 19$, $-31 \le l \le 31$	$-21 \le h \le 21$, $-27 \le k \le 27$, $-36 \le l \le 36$
Reflections collected	66380	45633	47577	52892
Independent reflections, R_{int}	7286, 0.0577	10239, 0.0463	7550, 0.0347	10086, 0.0714
Data/restraints/parameters	7286/276/423	10239/2/406	7550/8/348	10086/44/390
Goodness–of–fit on F^2	1.018	1.056	1.029	1.047
Final *R*1, *wR*2 [I>=2σ(I)]	0.0402, 0.0949	0.0407, 0.1042	0.0342, 0.0845	0.0449, 0.0940
Final *R*1, *wR*2 [all data]	0.0493, 0.1002	0.0634, 0.1127	0.0354, 0.0852	0.0735, 0.1009
Largest diff. peak/hole/ e Å^{-3}	1.02/-0.88	1.05/-0.66	1.19/-1.27	1.22/-0.99

Computational Details

The current computational methodology is the same as our previously published work.[6,7] DFT calculations were performed with Gaussian 16 (C.01).[8] In this study, six different methodologies have been assessed, A-F, which are described in Table S3 for reactions **1-4** (Scheme S1) and highlight the different functional, basis set and solvation correction combinations for each method. Initial BP86[9] (Methods A-E) and M06-2X[10] (Method F) optimizations were performed using the 'grid = ultrafine' option, with all stationary points being fully characterized via analytical frequency calculations as minima with all positive eigenvalues. Dispersion corrections were applied using Grimme's D3 parameters with Becke–Johnson damping ($D3^{BJ}$) for the BP86 functional, and standard D3 parameters[11] for M06-2X calculations. Corrections for the effect of benzene ($\varepsilon = 2.2706$) solvent were introduced using the polarizable continuum model (PCM) for methods A and D, conductor-like polarizable continuum model (CPCM) for method B, whilst methods D, E and F use the SMD continuum universal solvation model.[12]

Method A: BP86-$D3^{BJ}$(PCM=C_6H_6)/6-311++G**&def2-TZVPP//BP86/6-31G**&SDDALL

Method B: BP86-$D3^{BJ}$(CPCM=C_6H_6)/ZORA-def2-TZVPP/SARC//BP86/6-31G**&SDDALL
(Methodology employed in *Nat. Commun.* 2023, **14**, 8147-8152 and more recently in *Chem. Eur. J. 2025*, e202502197)

Method C: BP86-$D3^{BJ}$(SMD=C_6H_6)/ZORA-def2-TZVPP/SARC//BP86/6-31G**&SDDALL

Method D: BP86-$D3^{BJ}$(PCM=C_6H_6)/def2-TZVPP//BP86/def2-SVP

Method E: BP86-$D3^{BJ}$(SMD=C_6H_6)/def2-TZVPP//BP86/def2-SVP

Method F: M06-2X-D3(SMD=C_6H_6)/def2-TZVPP//BP86/def2-SVP

Table S2. Different basis set and solvation single point energy correction details for the six different methodology approaches used to work out the Gibbs free energy of the reactions shown in Scheme S1.

Method	Functional	Optimisation Basis Set	Single Point Energy Basis Set	Solvation Energy Single Point
A	BP86	6-31G** & SDDALL **BS1**	6-311++G** & def2-TZVPP **BS2**	PCM=C_6H_6
B	BP86	6-31G** & SDDALL **BS1**	ZORA-def2-TZVPP/SARC **BS3**	CPCM=C_6H_6
C	BP86	6-31G** & SDDALL **BS1**	ZORA-def2-TZVPP/SARC **BS3**	SMD=C_6H_6
D	BP86	def2-SVP **BS4**	def2-TZVPP **BS5**	PCM=C_6H_6
E	BP86	def2-SVP **BS4**	def2-TZVPP **BS5**	SMD=C_6H_6
F	M06-2X	def2-SVP **BS4**	def2-TZVPP **BS5**	SMD=C_6H_6

Optimisation calculations for methods A-C, describe the Na, K, Rb and Cs centres with the Stuttgart relativistic effective core potentials (RECPs)[13] and associated basis sets (SDDALL), with the 6-31G** basis set for all other atoms (BS1).[14] A polarization function was also added to K (ζ_d = 1.000), Rb (ζ_d = 0.491) and Cs (ζ_d = 0.306).[15] All energies were recomputed with a larger basis set featuring 6-311++G** basis sets on all atoms (method A, BS2),[16] except for Rb and Cs, where def2-TZVPP was employed.[17] In methods B and C, the all-electron ZORA-def2-TZVPP basis set was applied to all atoms except for Rb and Cs atoms, where ZORA(-SARC-)TZVPP was instead employed (BS3).[18] The Gibbs solvation energies and larger basis set energy correction (computing the electronic energy of M(0) in the gas phase) was computed with ORCA 5.0.1.,[19] where a combined solvation correction was obtained implicitly using the CPCM(C_6H_6) (Method B) and SMD(C_6H_6) (Method C) approach and a dispersion ($D3^{BJ}$) correction at the same basis set level.

For methods D-F, geometry optimisations were performed using the def2-SVP basis set (BS4) for all atoms, with all energies corrected by a single-point calculation with the larger basis set, def2-TZVPP (BS5). The energy solvation correction was computed with Gaussian 16 using two solvent models: PCM(C_6H_6) (method D) and SMD(C_6H_6) (methods E-F), and included the def2-TZVPP basis set.[17]

The Quantum Theory of Atoms in Molecules (QTAIM, AIMALL program[20]) was performed on the BP86/BS1-optimised geometries of $[LSnM]_2$ ($\mathbf{1^M}$) and $[LSnM]_2^{\bullet-}$ ($\mathbf{1^{M\bullet-}}$) radical anions (L = ^{Dipp}Ter, M = Li, Na, K, Rb and Cs). The topological analyses used wavefunction files obtained with Gaussian 16

(C.01) at the BP86 level of theory with the keyword output=wfx using the def2-TZVPP (BS5)[17] for H, Li, C, Na and K, aug-cc-pVTZ-PP (BS6)[21] basis set for Sn atoms, and def2-TZVP for Rb and Cs (BS7).[17] Contour plots were generated in the AIMStudio package, using critical point (CP) visualisation threshold values 0.02 a_0^{-3} (solid line BCP) and 0.005 a_0^{-3} (dashed line BCP = weak). The topological critical-point analysis satisfies the Poincaré–Hopf condition, confirming the internal consistency of the QTAIM partitioning. NBO analyses were carried out with NBO7[22] within Gaussian (C.01) at the same methodology level as the QTAIM calculations. The NBO energies of donor-acceptor interactions ("$\Delta E^{(2)}$") between the various molecular fragments of the structures were estimated with second-order perturbation theory analysis of the Fock matrix in the NBO basis, as calculated by NBO7, with selected donor-acceptor NBO interactions provided. Molecular and Natural Bonding Orbitals were represented using the Chemcraft program,[23] and the spin density maps with GaussView 6.1.1.[24]

Single-point EPR calculations were carried out using ORCA 6.0,[25] with the BP86/BS1 optimised geometry of $\mathbf{1}^{\mathbf{Na\bullet-}}$ used to compute a hyperfine coupling constant. Benchmarking of the methodology against the experimental obtained hyperfine coupling constant was carried out for the functional and basis set. The effect of solvation, benzene (ε = 2.2706), was considered using the SMD continuum universal solvation model. A dispersion correction was also applied to the methodology (see Table **1**). Additional keywords that were used include SOMF(1X),[26] which invokes the SOMF(1X) treatment of the spin-orbit coupling (SOC) operator that is useful for property calculations where heavy elements are involved. Furthermore, Resolution-of-the-Identity approximation (RI-J) was employed to improve the computational cost for the functionals with no Hartree-Fock exchange (*e.g.* BP86-D3^{BJ}, TPSS-D3^{BJ}, BLYP-D3^{BJ}). In the case of hybrid functionals (M06-D4, PBE0-D3^{BJ}, TPSSh-D3^{BJ}, B3LYP-D3^{BJ}, ωB97X-D4, CAM-B3LYP-D3^{BJ}) RIJCOSX[27] was used instead to speed up the calculation. In the %EPRNMR section, hyperfine keywords for all the atoms, AISO and ADIP, were included to request the isotropic Fermi-contact part of the HFC and its dipolar part, respectively. For Sn atoms, AORB was also considered, which calculates the spin-orbit contribution. "Picture-change" effects were also included.

Due to the presence of heavy elements, two all-electron def2-basis set with scalar relativistic Hamiltonians were tested: the zeroth order regular approximation (ZORA, BS3) and the exact 2-component (X2C, BS6). For BS3, ZORA-def2-TZVPP is combined with the Segmented All-electron Relativistically Contracted (SARC) basis set; ZORA-SARC-def2-TZVPP is used for Sn atoms and ZORA-def2-TZVPP for C and H atoms.[18] The universal auxiliary basis set (SARC/J) is used for the resolution of the identity approximation to Coulomb integrals. In the case of BS6, the Karlsruhe X2C basis set, X2C-TZVPPall,[28] was employed to include spin-orbit coupling and conceptualized to computed hyperfine coupling constants. Auxiliary X2C/J fitting basis for the all-electron x2c-XVPall basis sets was used.[29]

Breakdown of Energy Contributions

Table S3. Detailed contributions to relative energies as the successive corrections to the initial SCF energy are included. Terms used are:

ΔE_{BS1}	SCF energy computed with the BP86 functional and BS1
ΔG_{BS1}	Free energy at 298.15 K and 1 atm with the BP86 functional and BS1
$\Delta G_{BS1/PCM/C_6H_6}$	Free energy corrected for Benzene solvent (PCM) with the BP86 functional and BS1
$\Delta G_{BS1/D3^{BJ}/PCM/C_6H_6}$	Free energy corrected for Benzene solvent (PCM) and dispersion ($D3^{BJ}$) with the BP86 functional and BS1
ΔE_{BS2}	SCF energy computed with the BP86 functional and BS2
$\Delta E_{BS3/D3^{BJ}/CPCM/C_6H_6}$	SCF energy computed with Benzene solvent (CPCM) and dispersion ($D3^{BJ}$) with the BP86 functional and BS3
$\Delta E_{BS3/D3^{BJ}/SMD/C_6H_6}$	SCF energy computed with Benzene solvent (SMD) and dispersion ($D3^{BJ}$) with the BP86 functional and BS3
ΔG_A	Overall Free energy computed at the BP86-$D3^{BJ}$; PCM=Benzene/BS2//BP86/BS1 level
ΔG_B	Overall Free energy computed at the BP86-$D3^{BJ}$; CPCM=Benzene/BS3//BP86/BS1 level
ΔG_C	Overall Free energy computed at the BP86-$D3^{BJ}$; SMD=Benzene/BS3//BP86/BS1 level
ΔE_{BS4}	SCF energy computed with the BP86 functional and BS4
ΔG_{BS4}	Free energy at 298.15 K and 1 atm and with the BP86 functional and BS4
$\Delta G_{BS4/PCM/C_6H_6}$	Free energy corrected for Benzene solvent (PCM) with the BP86 functional and BS4
$\Delta G_{BS4/D3^{BJ}/PCM/C_6H_6}$	Free energy corrected for Benzene solvent (PCM) and dispersion ($D3^{BJ}$) with the BP86 functional and BS4
$\Delta G_{BS4/SMD/C_6H_6}$	Free energy corrected for Benzene solvent (SMD) with the BP86 functional and BS4
$\Delta G_{BS4/D3^{BJ}/SMD/C_6H_6}$	Free energy corrected for Benzene solvent (SMD) and dispersion ($D3^{BJ}$) with the BP86 functional and BS4
ΔE_{BS5}	SCF energy computed with BP86 functional and BS5
ΔG_D	Overall Free energy computed at the BP86-$D3^{BJ}$;PCM=Benzene/BS5//BP86/BS4 level
ΔG_E	Overall Free energy computed at the BP86-$D3^{BJ}$;SMD=Benzene/BS5//BP86/BS4 level
$\Delta E_{M062X/BS4}$	SCF energy computed with the M062X functional and BS4
$\Delta G_{M062X/BS4}$	Free energy at 298.15 K and 1 atm and with the M062X functional and BS4
$\Delta G_{M062X/BS4/SMD/C_6H_6}$	Free energy corrected for Benzene solvent (SMD) with the M062X functional and BS4
$\Delta G_{M062X/BS4/D3^{BJ}/SMD/C_6H_6}$	Free energy corrected for Benzene solvent (SMD) and dispersion ($D3^{BJ}$) with the M062X functional and BS4
$\Delta E_{M062X/BS5}$	SCF energy computed with the M062X functional and BS5
ΔG_F	Overall Free energy computed at the M062X-$D3^{BJ}$;SMD=Benzene/BS5//M062X/BS4 level

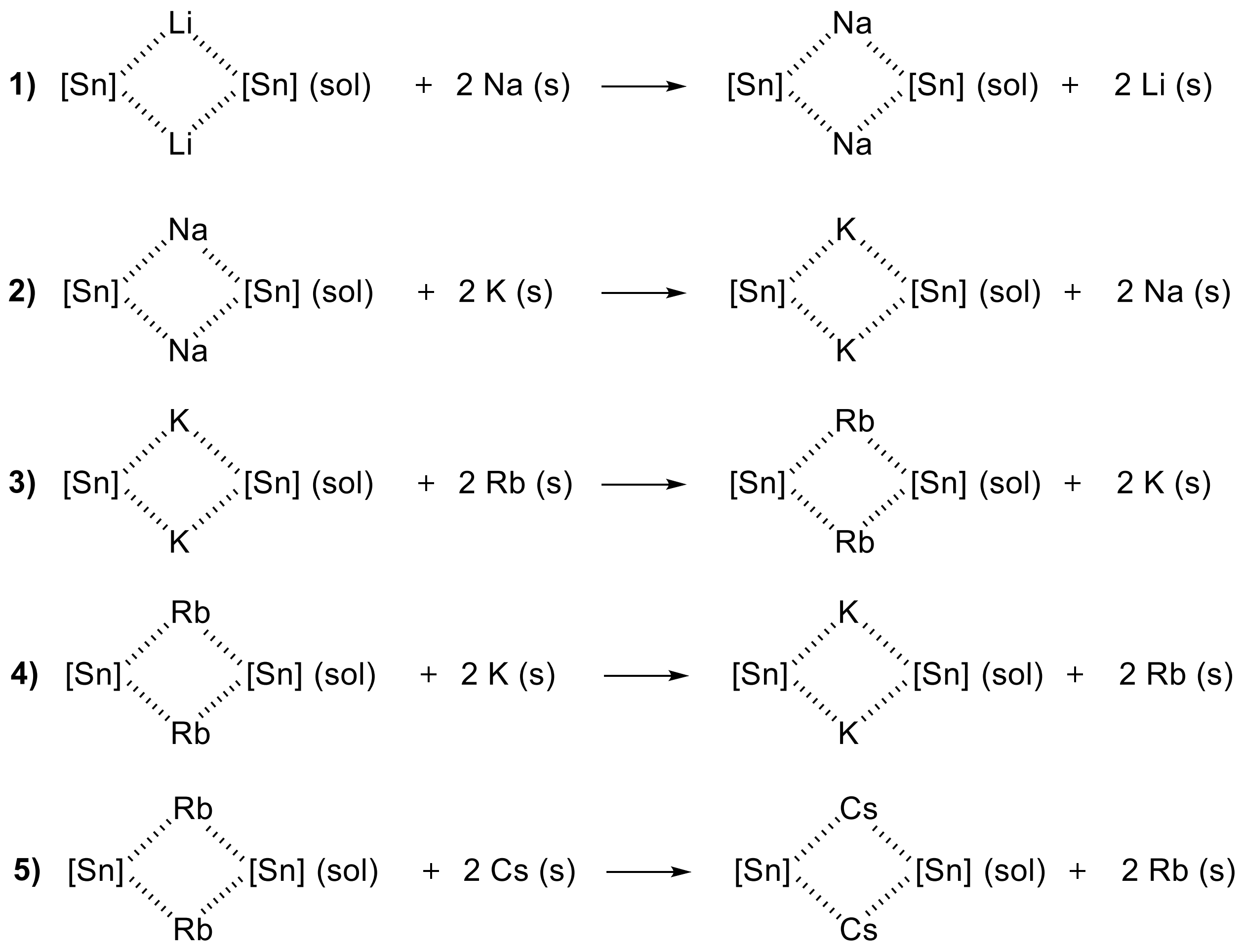

Scheme S1. Reactions of the doubly reduced distannyne analogues, $\mathbf{1^M}$, replace the alkali metal cations.

Table S4. Relative formation energy corrections (kcal mol^{-1}) for computed reductions of $\mathbf{1^M}$ (reaction: $\mathbf{1^M} \rightarrow \mathbf{1^{M'}}$), for methods A-C in solution.

	ΔE_{BS1}	ΔG_{BS1}	$\Delta G_{BS1/PCM/C_6H_6}$	$\Delta G_{BS1/D3^{BJ}/PCM/C_6H_6}$	ΔE_{BS2}	$\Delta E_{BS3/D3^{BJ}/CPCM/C_6H_6}$	$\Delta E_{BS3/D3^{BJ}/SMD/C_6H_6}$
$\mathbf{1^{Li}} \rightarrow \mathbf{1^{Na}}$	21.0	23.3	17.8	23.5	18.7	23.0	21.7
$\mathbf{1^{Na}} \rightarrow \mathbf{1^{K}}$	11.1	8.7	6.8	17.1	0.3	12.4	12.3
$\mathbf{1^{K}} \rightarrow \mathbf{1^{Rb}}$	9.1	8.9	7.9	8.1	11.9	4.7	4.5
$\mathbf{1^{Rb}} \rightarrow \mathbf{1^{K}}$	−9.1	−8.9	−7.9	−8.1	−11.9	−4.7	−4.5
$\mathbf{1^{Rb}} \rightarrow \mathbf{1^{Cs}}$	0.9	1.1	0.8	0.1	0.1	2.8	2.7

Table S5. Relative Gibbs formation energies (kcal mol^{-1}) for computed reductions of $\mathbf{1^M}$ (reaction: $\mathbf{1^M} \rightarrow \mathbf{1^{M'}}$), for methods A-C in solution.

	ΔG_A	ΔG_B	ΔG_C
$\mathbf{1^{Li}} \rightarrow \mathbf{1^{Na}}$	21.2	25.3	23.9
$\mathbf{1^{Na}} \rightarrow \mathbf{1^{K}}$	6.3	10.0	9.9
$\mathbf{1^{K}} \rightarrow \mathbf{1^{Rb}}$	10.9	4.5	4.2
$\mathbf{1^{Rb}} \rightarrow \mathbf{1^{K}}$	−10.9	−4.5	−4.2
$\mathbf{1^{Rb}} \rightarrow \mathbf{1^{Cs}}$	−0.8	2.9	2.9

Table S6. Relative formation energy corrections (kcal mol^{-1}) for computed reductions of $\mathbf{1^M}$ (reaction: $\mathbf{1^M} \rightarrow \mathbf{1^{M'}}$), for methods D and E in solution.

	ΔE_{BS4}	ΔG_{BS4}	$\Delta G_{BS4/PCM/C_6H_6}$	$\Delta G_{BS4/D3^{BJ}/PCM/C_6H_6}$	$\Delta G_{BS4/SMD/C_6H_6}$	$\Delta G_{BS4/D3^{BJ}/SMD/C_6H_6}$	ΔE_{BS5}
$\mathbf{1^{Li}} \rightarrow \mathbf{1^{Na}}$	16.1	18.2	11.5	16.8	16.1	21.3	18.1
$\mathbf{1^{Na}} \rightarrow \mathbf{1^{K}}$	4.1	2.5	0.9	10.7	1.8	11.6	1.1
$\mathbf{1^{K}} \rightarrow \mathbf{1^{Rb}}$	13.1	12.0	10.4	11.3	12.5	13.4	11.9
$\mathbf{1^{Rb}} \rightarrow \mathbf{1^{K}}$	−13.1	−12.0	−10.4	−11.3	−12.5	−13.4	−11.9
$\mathbf{1^{Rb}} \rightarrow \mathbf{1^{Cs}}$	3.3	3.5	3.3	3.2	3.6	3.5	0.3

Table S7. Relative Gibbs formation energies (kcal mol^{-1}) for computed reductions of $\mathbf{1^M}$ (reaction: $\mathbf{1^M} \rightarrow \mathbf{1^{M'}}$), for methods D and E in solution.

	ΔG_D	ΔG_E
$\mathbf{1^{Li}} \rightarrow \mathbf{1^{Na}}$	18.7	23.3
$\mathbf{1^{Na}} \rightarrow \mathbf{1^{K}}$	7.8	8.6
$\mathbf{1^{K}} \rightarrow \mathbf{1^{Rb}}$	10.1	12.2
$\mathbf{1^{Rb}} \rightarrow \mathbf{1^{K}}$	−10.1	−12.2
$\mathbf{1^{Rb}} \rightarrow \mathbf{1^{Cs}}$	0.2	0.5

Table S8. Relative formation energy corrections and Gibbs formation energy (kcal mol^{-1}) for computed reductions of $\mathbf{1^M}$ (reaction: $\mathbf{1^M} \rightarrow \mathbf{1^{M'}}$), for method F in solution.

	$\Delta E_{M062X/BS4}$	$\Delta G_{M062X/BS4}$	$\Delta G_{M062X/BS4/SMD/C_6H_6}$	$\Delta G_{M062X/D3^{BJ}/BS4/SMD/C_6H_6}$	$\Delta E_{M062X/BS5}$	ΔG_F
$\mathbf{1^{Li}} \rightarrow \mathbf{1^{Na}}$	18.2	20.3	17.5	17.9	20.6	20.2
$\mathbf{1^{Na}} \rightarrow \mathbf{1^{K}}$	6.0	3.9	2.7	3.6	1.7	−0.3
$\mathbf{1^{K}} \rightarrow \mathbf{1^{Rb}}$	11.0	9.4	10.4	10.7	9.1	8.7
$\mathbf{1^{Rb}} \rightarrow \mathbf{1^{K}}$	−11.0	−9.4	−10.4	−10.7	−9.1	−8.7
$\mathbf{1^{Rb}} \rightarrow \mathbf{1^{Cs}}$	1.2	1.7	1.8	2.1	−0.9	0.0

Table S9. Summaries of Gibbs energy (in kcal mol^{-1}) for reactions **1-5** following different methodologies (A-F) in solution and using a combined experimental and computational Hess cycle.[6,7]

Reaction	ΔG (*sol*) / kcal mol^{-1}						ΔG (*sol&s*) / kcal mol^{-1}					
	**A**	**B**	**C**	**D**	**E**	**F**	**A**	**B**	**C**	**D**	**E**	**F**
**1**	21.2	25.3	23.9	18.7	23.3	20.2	4.1	2.0	2.6	1.3	12.8	8.6
**2**	6.3	10.0	9.9	7.8	8.6	−0.3	0.6	2.1	2.6	2.2	1.6	−8.2
**3**	10.9	4.5	4.2	10.1	12.2	8.7	7.9	1.0	1.4	7.3	3.1	5.2
**4**	−10.9	−4.5	−4.2	−10.1	−12.2	−8.7	−7.9	−1.0	−1.4	−7.3	−3.1	−5.2
**5**	−0.8	2.9	2.9	0.2	0.5	0.0	−2.4	1.3	2.0	−1.1	−0.3	−1.7

QTAIM Analysis for 1^{M} and $1^{M\bullet-}$

M = Li

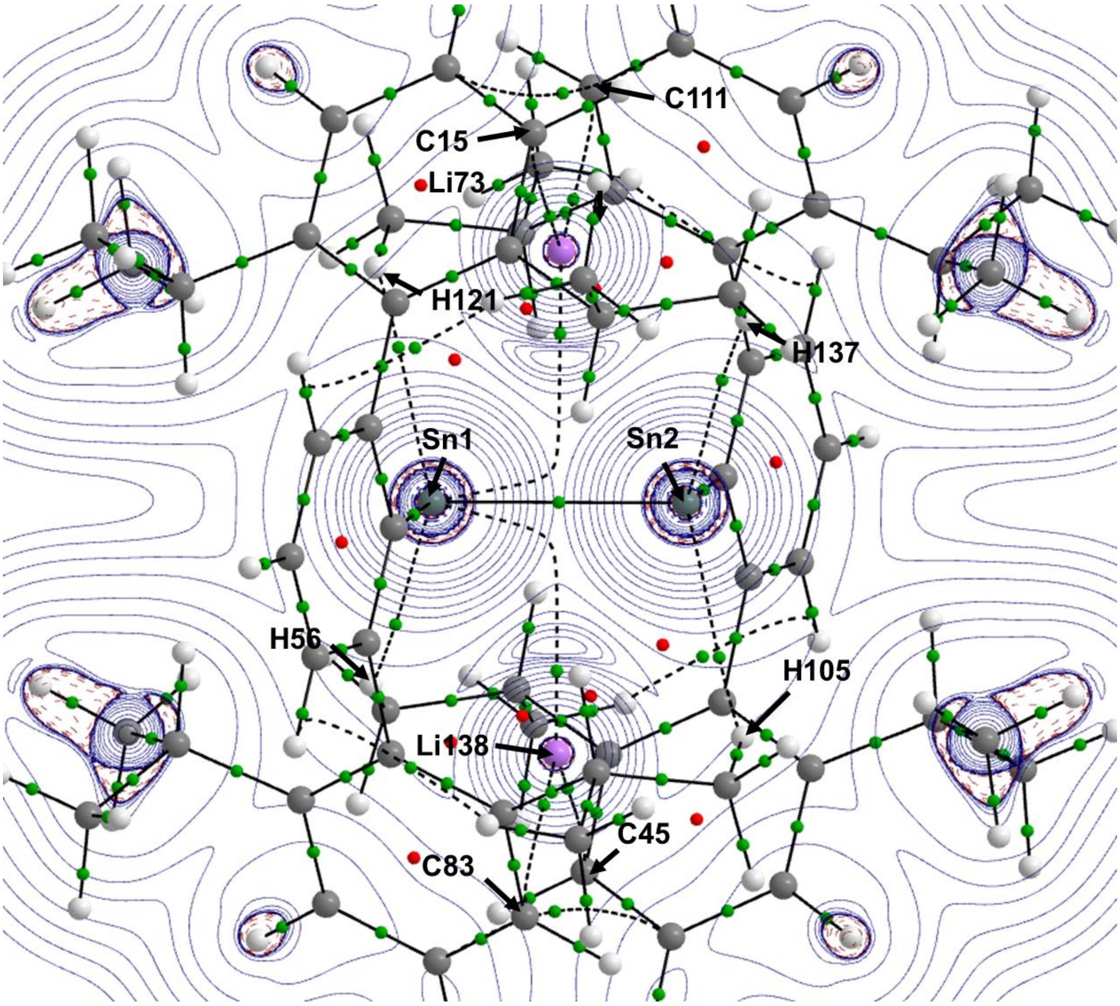

Figure S26. Contour plot of the Laplacian ($\nabla^2\rho(r)$) in the {Sn1-Sn2-Li73} plane of **[LSnLi]₂, 1^{Li}**.

Table S10. Selected QTAIM BCP data for **$[LSnLi]_2$, 1^{Li}**.

Atoms	ρ(r)	∇²ρ(r)	ε	G(r)	V(r)	H(r)
Sn1-Sn2	0.048934	+0.016915	0.546108	+0.017395	−0.030561	−0.013166
Sn1-Li138	0.011783	+0.017371	0.897138	+0.005181	−0.006020	−0.000839
Sn1-Li73	0.012251	+0.017554	0.782441	+0.005346	−0.006303	−0.000957
C83-Li138	0.009863	+0.046475	0.998047	+0.009513	−0.007407	+0.002106
C46-Li138	0.009869	+0.046507	0.998794	+0.009520	−0.007413	+0.002107
C15-Li73	0.007507	+0.038233	1.342704	+0.007596	−0.005634	+0.001962
Li73-C111	0.007487	+0.038146	1.343835	+0.007577	−0.005618	+0.001959
Sn1-H121	0.006867	+0.012249	0.108159	+0.002705	−0.002348	+0.000357
Sn1-H56	0.005977	+0.009559	0.426155	+0.002153	−0.001917	+0.000236
Sn2-H105	0.005979	+0.009560	0.426492	+0.002154	−0.001918	+0.000236
Sn2-H37	0.006873	+0.012262	0.108329	+0.002708	−0.002351	+0.000357

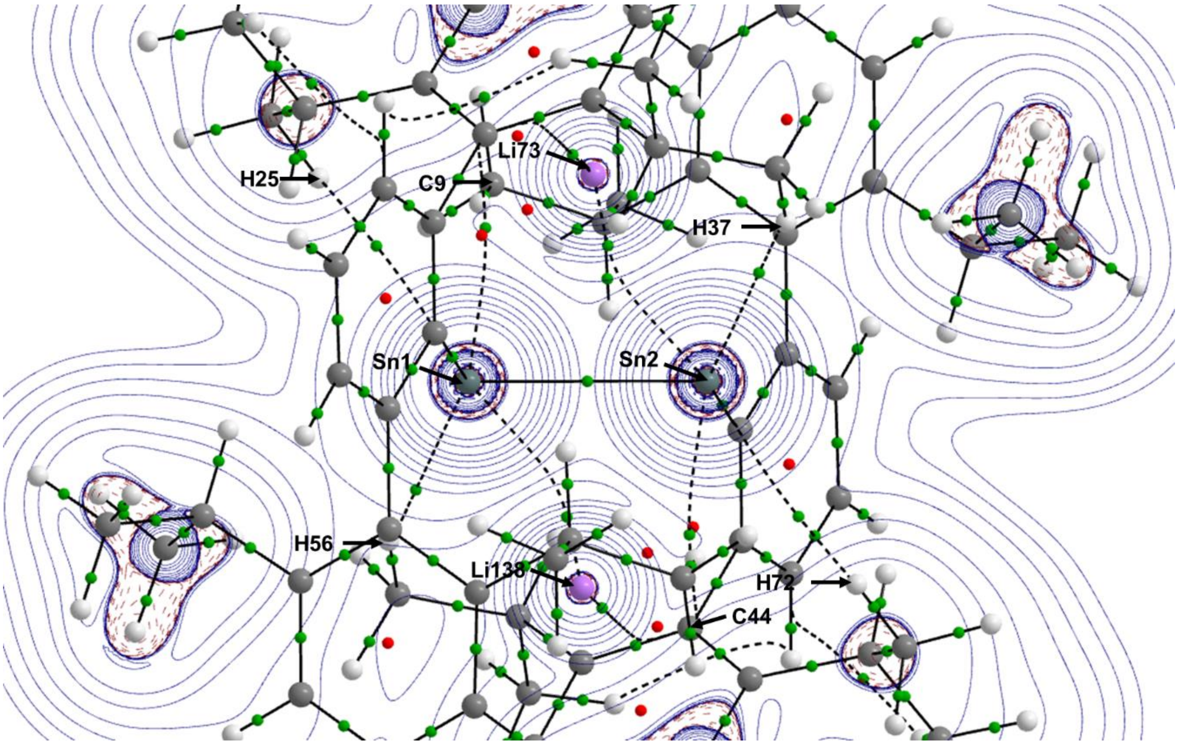

Figure S27. Contour plot of the Laplacian ($\nabla^2\rho(r)$) in the {Sn1-Sn2-Li73} plane of **$[LSnLi]_2^{\bullet-}$** radical anion, **$1^{Li\bullet-}$.**

Table S11. Selected QTAIM BCP data for **$[LSnLi]_2^{\bullet-}$** radical anion, **$1^{Li\bullet-}$.**

**Atoms**	**ρ(r)**	**$\nabla^2\rho(r)$**	**ε**	**G(r)**	**V(r)**	**H(r)**
Sn1-Sn2	0.041464	+0.016170	0.753545	+0.013290	−0.022537	−0.009247
Sn1-Li138	0.015206	+0.027885	1.824670	+0.008064	−0.009156	−0.001092
Sn2-Li73	0.015209	+0.027900	1.821645	+0.008067	−0.009159	−0.001092
C44-Li138	0.013748	+0.075383	4.708210	+0.015881	−0.012917	+0.002964
C9-Li73	0.013741	+0.075332	4.687054	+0.015869	−0.012906	+0.002963
Sn1-H25	0.005725	+0.009899	0.115800	+0.002167	−0.001860	+0.000307
Sn1-H56	0.007690	+0.012870	0.200131	+0.002916	−0.002615	+0.000301
Sn1-C9	0.010138	+0.015247	1.210648	+0.003769	−0.003727	+0.000042
Sn2-C44	0.010136	+0.015227	1.194507	+0.003765	−0.003724	+0.000041
Sn2-H72	0.005721	+0.009893	0.116318	+0.002166	−0.001859	+0.000307
Sn2-H37	0.007687	+0.012863	0.200486	+0.002915	−0.002614	+0.000301

M = Na

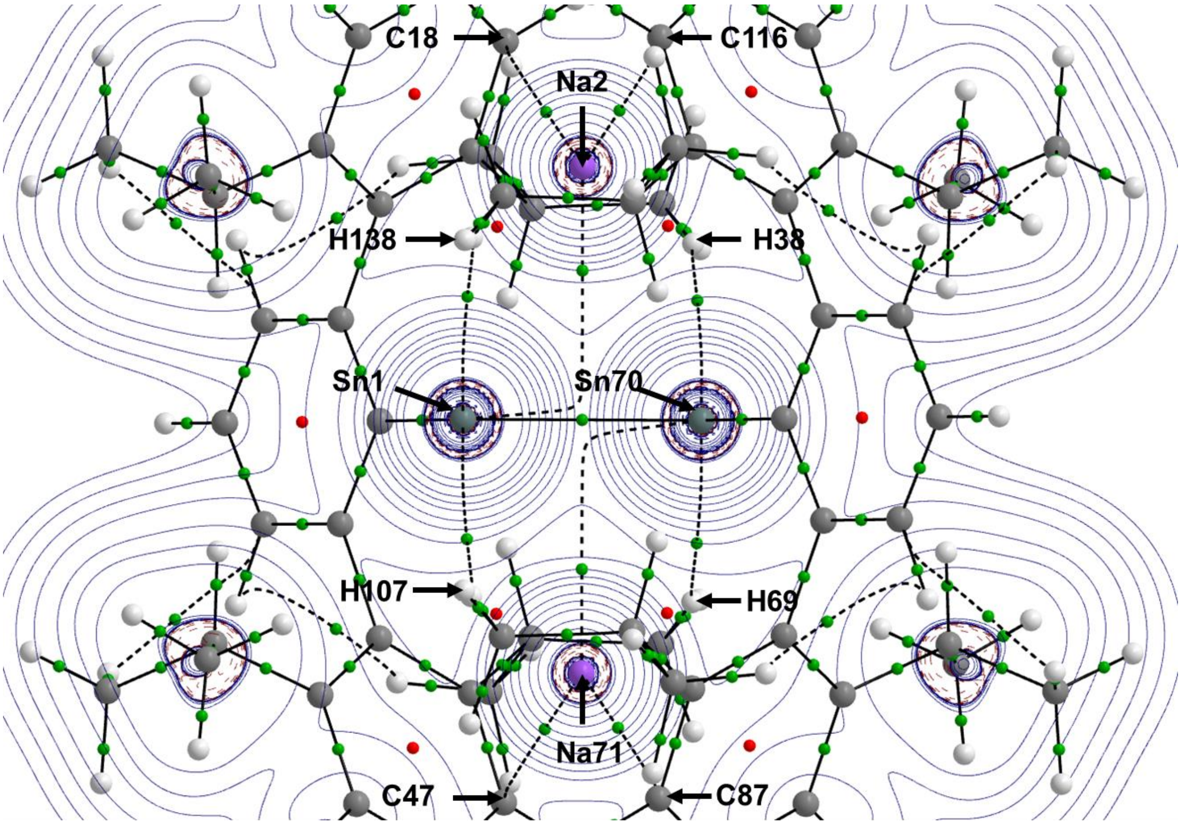

Figure S28. Contour plot of the Laplacian ($\nabla^2\rho(r)$) in the {Sn1-Sn2-Na2} plane of **[LSnNa]$_2$, 1^{Na}**.

Table S12. Selected QTAIM BCP data for **[LSnNa]$_2$, 1^{Na}**.

Atoms	ρ(r)	∇²ρ(r)	ε	G(r)	V(r)	H(r)
Sn1-Sn70	0.047730	+0.014403	0.491597	0.016054	−0.028508	−0.012454
Sn1-Na2	0.011618	+0.019240	2.736099	0.004842	−0.004874	−0.000032
Sn70-Na71	0.011619	+0.019240	2.736277	0.004842	−0.004874	−0.000032
Na2-C18	0.008672	+0.037721	2.041608	0.007607	−0.005785	+0.001822
Na2-C116	0.008662	+0.037666	2.038309	0.007604	−0.005776	+0.001828
C47-Na71	0.008660	+0.037666	2.044987	0.007596	−0.005775	+0.001821
Na71-C87	0.008672	+0.037723	2.043418	0.007608	−0.005785	+0.001823
Sn1-H138	0.005710	+0.009677	0.231996	0.002127	−0.001836	+0.000291
Sn1-H107	0.005726	+0.009705	0.231523	0.002134	−0.001843	+0.000291
H38-Sn70	0.005727	+0.009705	0.231930	0.002135	−0.001843	+0.000292
H69-Sn70	0.005716	+0.009687	0.232114	0.002130	−0.001838	+0.000292

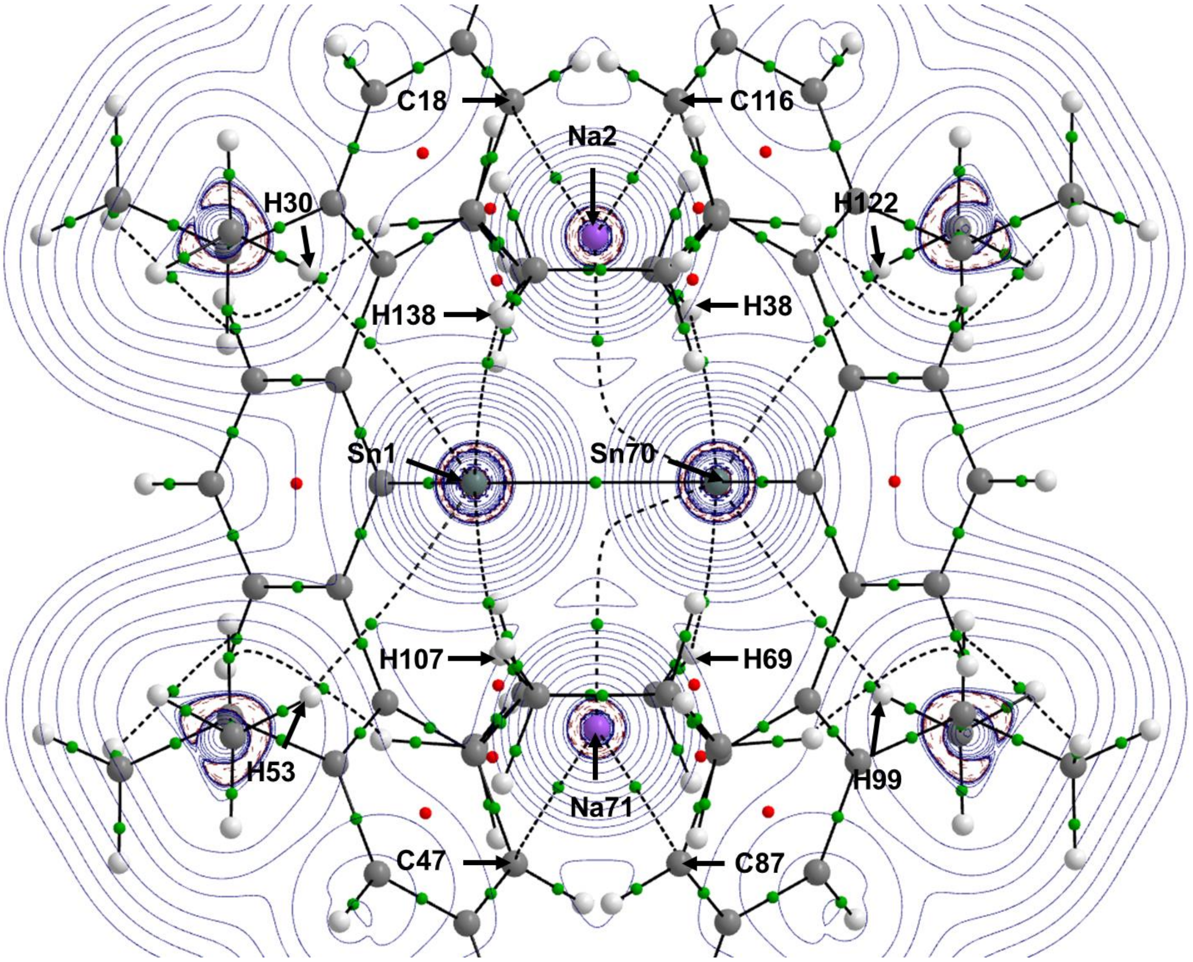

Figure S29. Contour plot of the Laplacian ($\nabla^2\rho(r)$) in the {Sn1-Sn2-Na2} plane of **[LSnNa]$_2$$^{\bullet-}$** radical anion, **1$^{Na\bullet-}$**.

Table S13. Selected QTAIM BCP data for **[LSnNa]$_2$$^{\bullet-}$** radical anion, **1$^{Na\bullet-}$**.

Atoms	ρ(r)	∇²ρ(r)	ε	G(r)	V(r)	H(r)
Sn1-Sn70	0.042336	+0.013923	0.529654	+0.013228	−0.022975	−0.009747
Na2-Sn70	0.012176	+0.021143	62.322054	+0.005311	−0.005336	−0.000025
Sn70-Na71	0.012175	+0.021142	62.237949	+0.005311	−0.005336	−0.000025
Na2-C116	0.007502	+0.032628	2.618774	+0.006542	−0.004927	+0.001615
Na2-C18	0.007502	+0.032634	2.626118	+0.006543	−0.004928	+0.001615
Na71-C87	0.007490	+0.032582	2.637377	+0.006532	−0.004919	+0.001613
C47-Na71	0.007498	+0.032618	2.628690	+0.006540	−0.004926	+0.001614
Sn1-H138	0.006170	+0.009744	0.190095	+0.002191	−0.001946	+0.000245
Sn1-H30	0.005248	+0.009064	0.067483	+0.001961	−0.001656	+0.000305
Sn1-H53	0.005257	+0.009074	0.066320	+0.001964	−0.001660	+0.000304
Sn1-H107	0.006165	+0.009736	0.189947	+0.002189	−0.001944	+0.000245
H38-Sn70	0.006170	+0.009742	0.190286	+0.002191	−0.001946	+0.000245
Sn70-H122	0.005255	+0.009071	0.066518	+0.001963	−0.001659	+0.000304
H69-Sn70	0.006162	+0.009733	0.189956	+0.002188	−0.001943	+0.000245
Sn70-H99	0.005239	+0.009050	0.068158	+0.001958	−0.001653	+0.000305

M = K

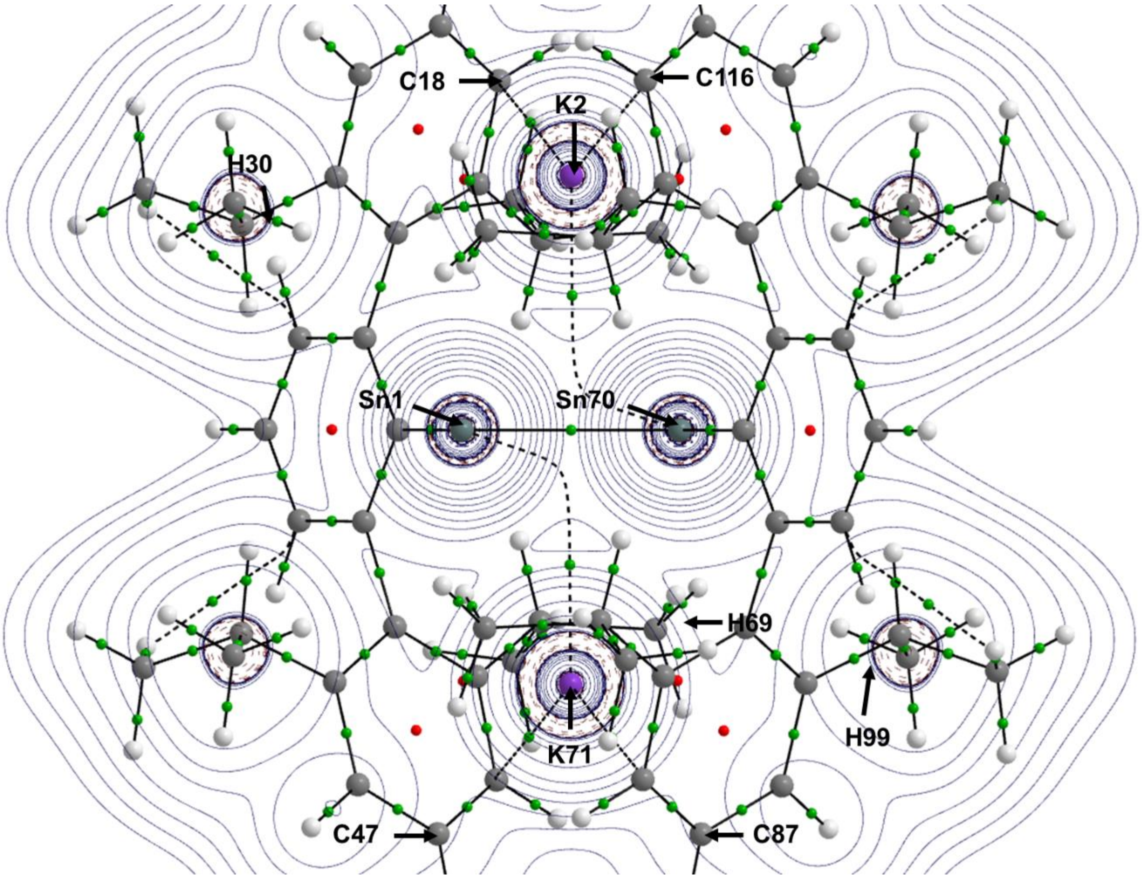

Figure S30. Contour plot of the Laplacian ($\nabla^2\rho(r)$) in the {Sn1-Sn2-K2} plane of **[LSnK]$_2$, 1^K**.

Table S14. Selected QTAIM BCP data for **[LSnK]$_2$, 1^K**.

Atoms	ρ(r)	∇²ρ(r)	ε	G(r)	V(r)	H(r)
Sn1-Sn70	0.047610	+0.013529	0.440037	+0.015718	−0.028054	−0.012336
K2-Sn70	0.010556	+0.017044	5.373425	+0.004069	−0.003876	+0.000193
Sn1-K71	0.010556	+0.017044	5.373425	+0.004069	−0.003876	+0.000193
K2-C18	0.010122	+0.039399	3.943375	+0.008057	−0.006264	+0.001793
K2-C116	0.010218	+0.039705	3.601401	+0.008126	−0.006327	+0.001799
C47-K71	0.010218	+0.039705	3.601401	+0.008126	−0.006327	+0.001799
K71-C87	0.010122	+0.039399	3.943375	+0.008057	−0.006264	+0.001793

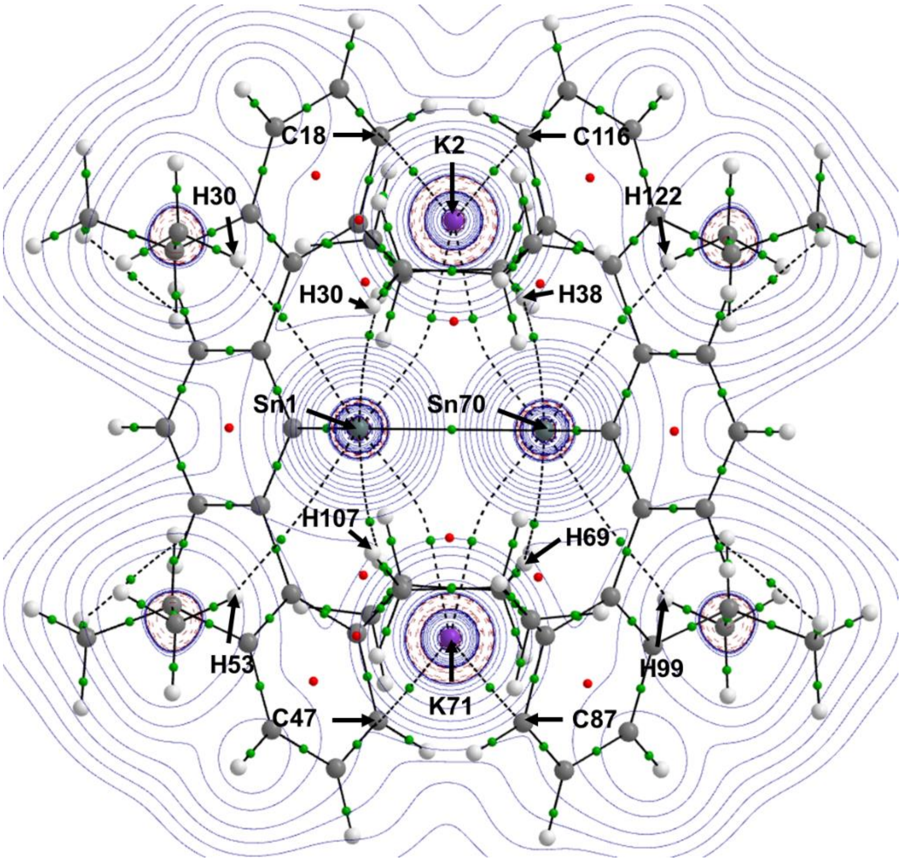

Figure S31. Contour plot of the Laplacian ($\nabla^2\rho(r)$) in the {Sn1-Sn2-K2} plane of **[LSnK]$_2$$^{\bullet-}$** radical anion, **1$^{K\bullet-}$**.

Table S15. Selected QTAIM BCP data for **[LSnK]$_2$$^{\bullet-}$** radical anion, **1$^{K\bullet-}$**.

Atoms	ρ(r)	∇²ρ(r)	ε	G(r)	V(r)	H(r)
Sn1-Sn70	0.042054	+0.012892	0.449191	+0.012790	−0.022358	−0.009568
Sn1-K2	0.011040	+0.020364	3.454984	+0.004818	−0.004546	+0.000272
K2-Sn70	0.010965	+0.019798	5.633001	+0.004696	−0.004442	+0.000254
Sn1-K71	0.010965	+0.019798	5.633001	+0.004696	−0.004442	+0.000254
Sn70-K71	0.011040	+0.020364	3.454984	+0.004818	−0.004546	+0.000272
K2-C18	0.009576	+0.037686	4.464285	+0.007667	−0.005913	+0.001754
K2-C116	0.009681	+0.038096	4.372325	+0.007761	−0.005998	+0.001763
K71-C87	0.011040	+0.020364	3.454984	+0.004818	−0.004546	+0.000272
C47-K71	0.010965	+0.019798	5.633001	+0.004696	−0.004442	+0.000254
Sn1-H138	0.005035	+0.007811	0.147336	+0.001719	−0.001484	+0.000235
Sn1-H30	0.005139	+0.008360	0.043569	+0.001828	−0.001566	+0.000262
Sn1-H53	0.005144	+0.008464	0.062811	+0.001847	−0.001579	+0.000268
Sn1-H107	0.005476	+0.008482	0.139940	+0.001887	−0.001654	+0.000233
H38-Sn70	0.005476	+0.008482	0.139940	+0.001887	−0.001654	+0.000233
Sn70-H122	0.005144	+0.008464	0.062811	+0.001847	−0.001579	+0.000268
H69-Sn70	0.005035	+0.007811	0.147336	+0.001719	−0.001484	+0.000235
Sn70-H99	0.005139	+0.008360	0.043569	+0.001828	−0.001566	+0.000262

M = Rb

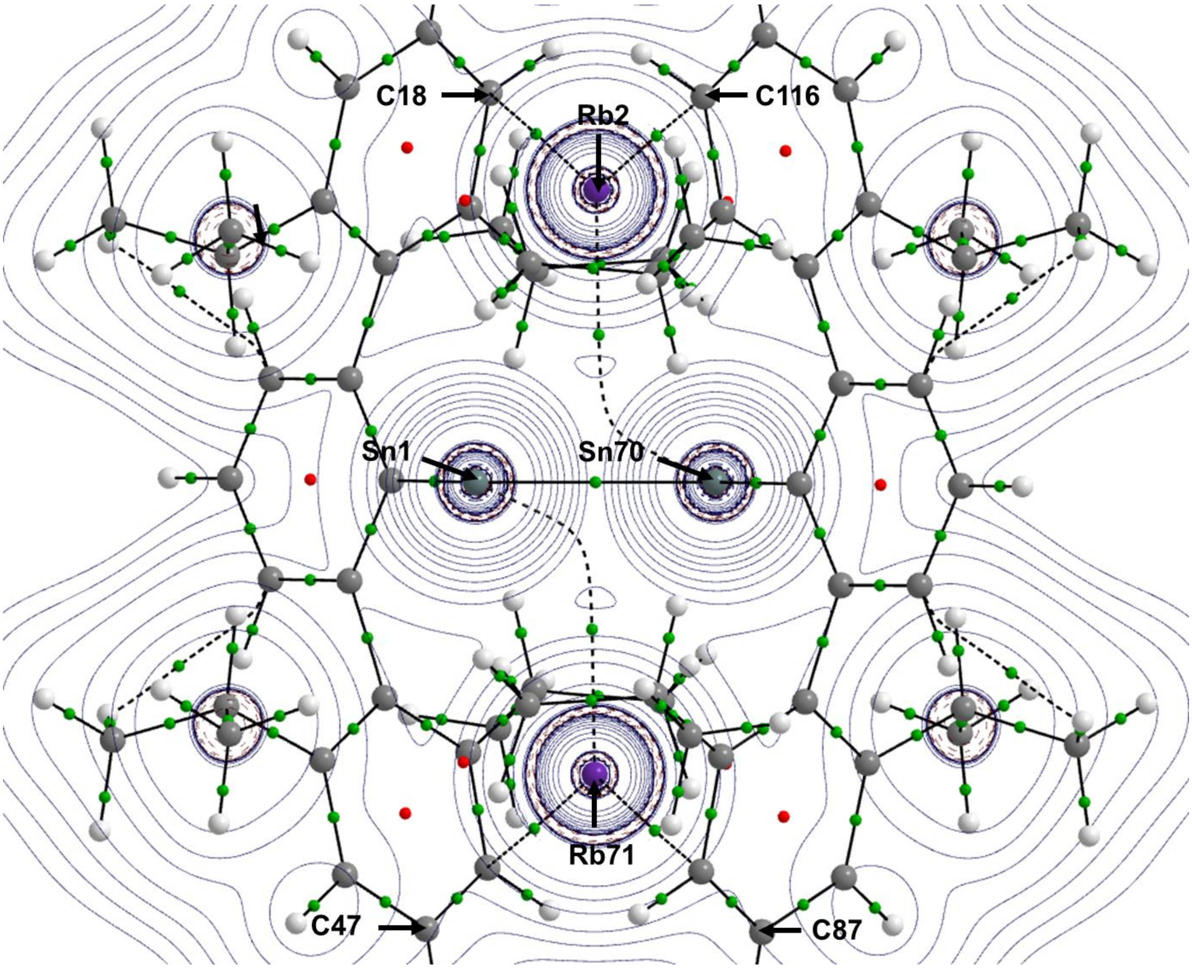

Figure S32. Contour plot of the Laplacian ($\nabla^2\rho(r)$) in the {Sn1-Sn2-Rb2} plane of **$[LSnRb]_2$, 1^{Rb}**.

Table S16. Selected QTAIM BCP data for **$[LSnRb]_2$, 1^{Rb}**.

Atoms	ρ(r)	∇²ρ(r)	ε	G(r)	V(r)	H(r)
Sn1-Sn70	0.046543	+0.013285	0.433770	0.015028	−0.026735	−0.011707
Rb2-Sn70	0.010303	+0.014607	9.869283	0.003467	−0.003343	0.000124
Sn1-Rb71	0.010303	+0.014607	9.869283	0.003467	−0.003343	0.000124
Rb2-C18	0.010579	+0.036025	4.302456	0.007600	−0.006238	0.001362
Rb2-C116	0.010538	+0.035915	4.379112	0.007572	−0.006209	0.001363
C47-Rb71	0.010538	+0.035915	4.379112	0.007572	−0.006210	0.001362
Rb71-C87	0.010579	+0.036025	4.302456	0.007600	−0.006238	0.001362

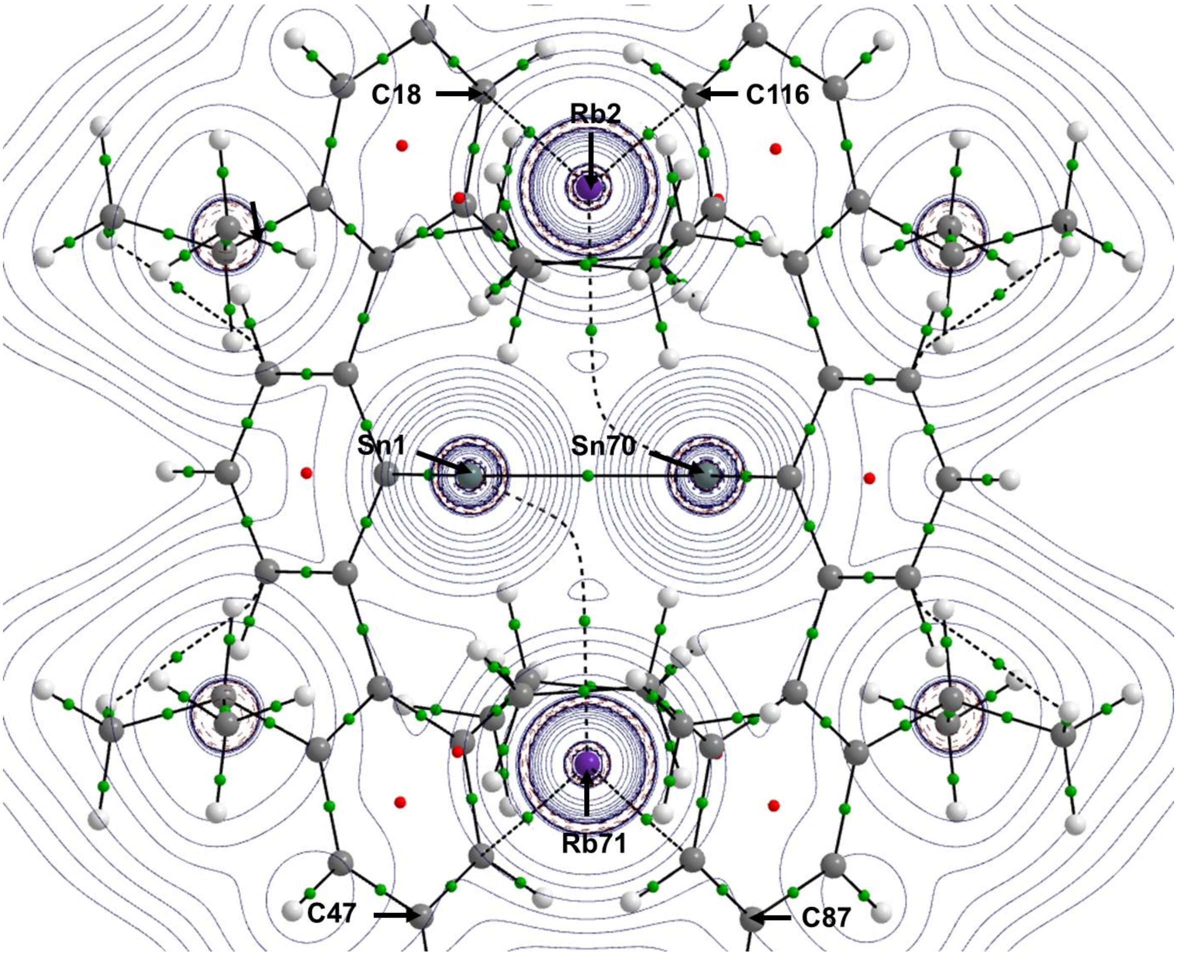

Figure S33. Contour plot of the Laplacian ($\nabla^2\rho(r)$) in the {Sn1-Sn2-Rb2} plane of **[LSnRb]$_2$$^{\bullet-}$** radical anion, **1$^{Rb\bullet-}$**.

Table S17. Selected QTAIM BCP data for **[LSnRb]$_2$$^{\bullet-}$** radical anion, **1$^{Rb\bullet-}$**.

Atoms	ρ(r)	∇²ρ(r)	ε	G(r)	V(r)	H(r)
Sn1-Sn70	0.040394	+0.012668	0.443589	+0.011881	−0.020594	−0.008713
Sn1-Rb2	0.011016	+0.018118	1.898733	+0.004348	−0.004221	+0.000127
Rb2-Sn70	0.010836	+0.017490	2.778052	+0.004185	−0.004055	+0.000130
Sn1-Rb71	0.010836	+0.017490	2.778052	+0.004185	−0.004055	+0.000130
Sn70-Rb71	0.004672	+0.007254	0.127993	+0.001589	−0.001366	+0.000223
Rb2-C18	0.009946	+0.034398	5.963210	+0.007201	−0.005851	+0.001350
Rb2-C116	0.010013	+0.034637	5.605600	+0.007257	−0.005902	+0.001355
C47-Rb71	0.010836	+0.017490	2.778052	+0.004185	−0.004055	+0.000130
Rb71-C87	0.011016	+0.018118	1.898733	+0.004348	−0.004221	+0.000127
Sn1-H30	0.005061	+0.007972	0.031479	+0.001760	−0.001527	+0.000233
Sn1-H53	0.005119	+0.008135	0.041628	+0.001795	−0.001556	+0.000239
Sn70-H122	0.005119	+0.008135	0.041628	+0.001795	−0.001556	+0.000239
Sn70-H99	0.005061	+0.007972	0.031479	+0.001760	−0.001527	+0.000233

M = Cs

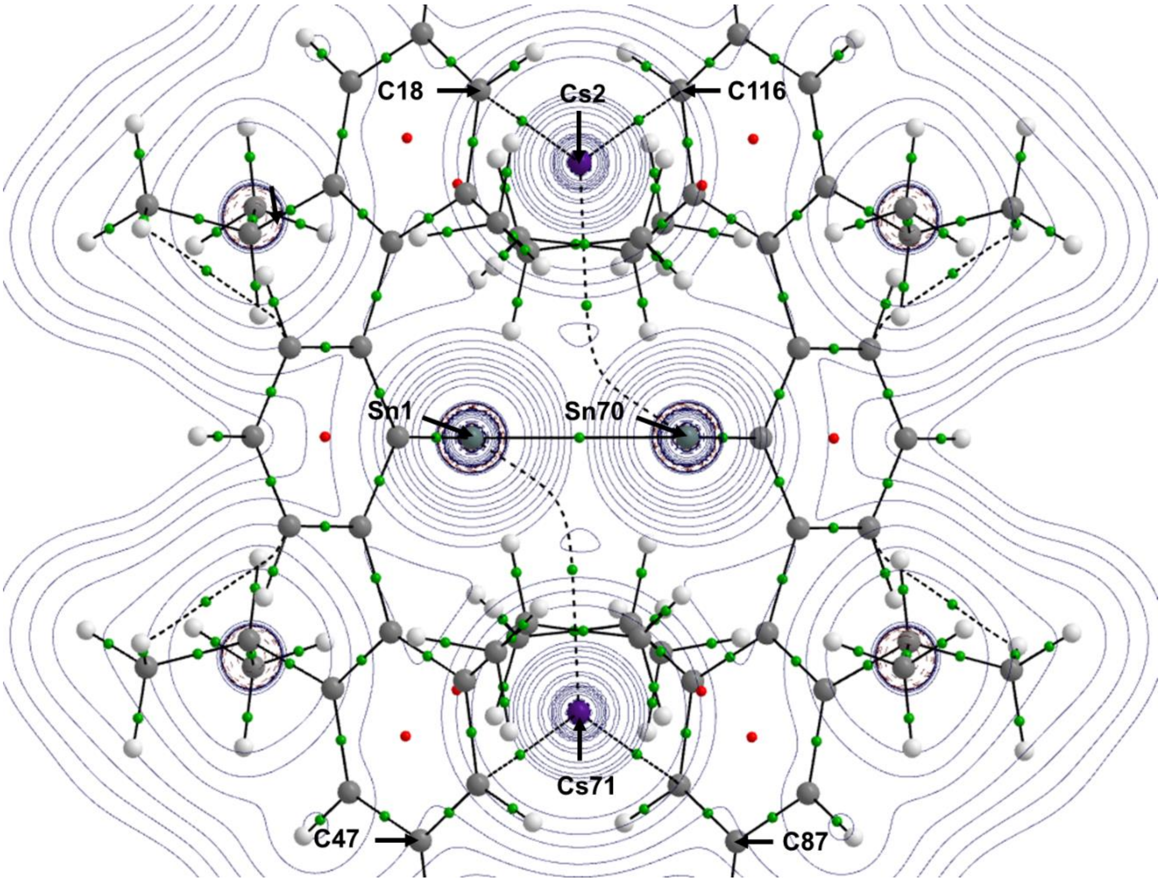

Figure S34. Contour plot of the Laplacian ($\nabla^2\rho(r)$) in the {Sn1-Sn2-Cs2} plane of **[LSnCs]**$_2$, **1**^{Cs}.

Table S18. Selected QTAIM BCP data for **[LSnCs]**$_2$, **1**^{Cs}.

Atoms	ρ(r)	∇²ρ(r)	ε	G(r)	V(r)	H(r)
Sn1-Sn70	0.046153	+0.013183	0.409754	+0.014796	−0.026297	−0.011501
Cs2-Sn70	0.010136	+0.013672	8.466274	+0.003092	−0.003026	+0.000066
Sn1-Cs71	0.004234	+0.007607	0.156836	+0.001604	−0.001305	+0.000299
Cs2-C18	0.011347	+0.037163	5.726739	+0.007594	−0.006461	+0.001133
Cs2-C116	0.011459	+0.037356	4.683308	+0.007637	−0.006506	+0.001131
C47-Cs71	0.011459	+0.037356	4.683308	+0.007637	−0.006506	+0.001131
Cs71-C87	0.011347	+0.037163	5.726739	+0.007594	−0.006461	+0.001133

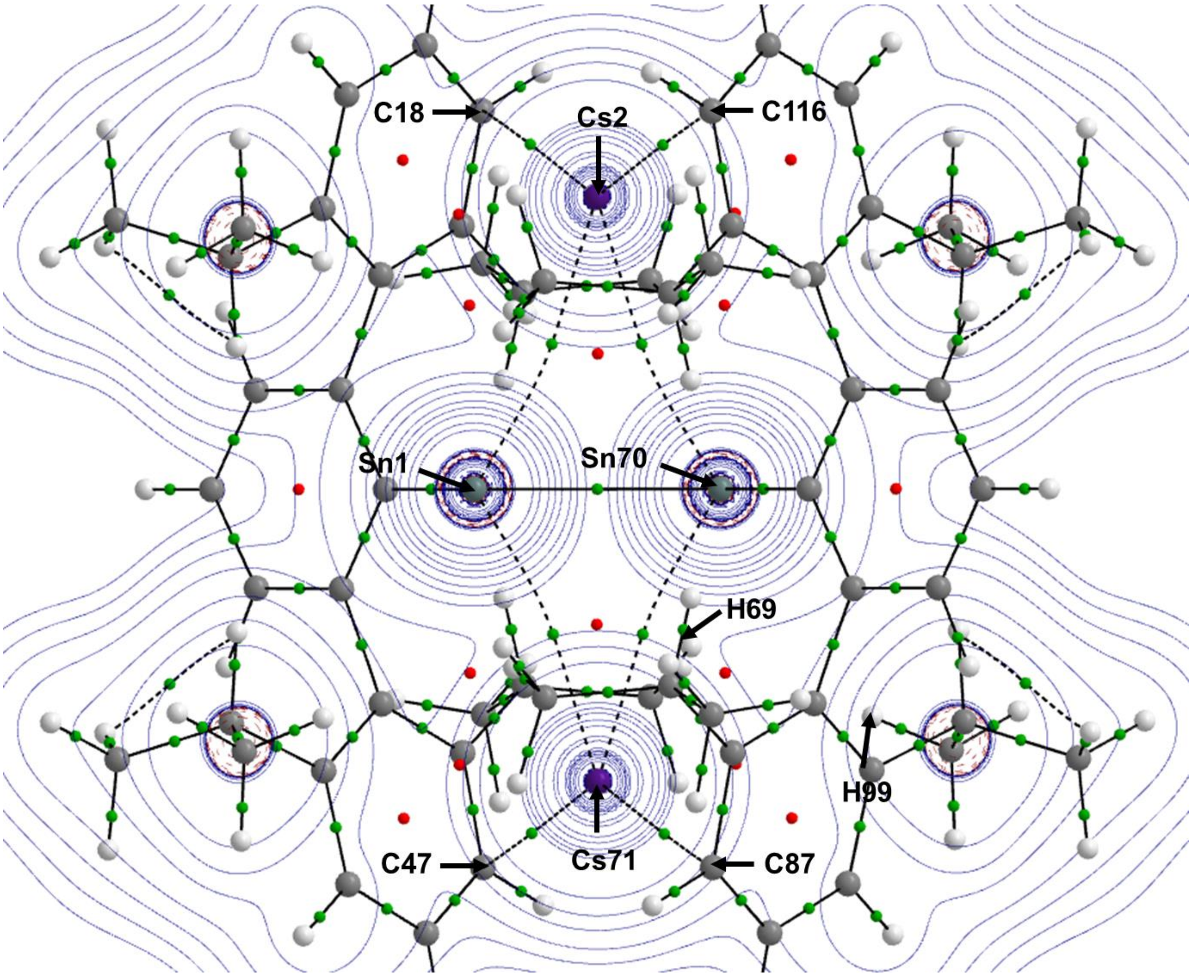

Figure S35. Contour plot of the Laplacian ($\nabla^2\rho(r)$) in the {Sn1-Sn2-Cs2} plane of **$[LSnCs]_2^{\bullet -}$** radical anion, **$1^{Cs\bullet -}$**.

Table S19. Selected QTAIM BCP data for **$[LSnCs]_2^{\bullet -}$** radical anion, **$1^{Cs\bullet -}$**.

Atoms	ρ(r)	∇²ρ(r)	ε	G(r)	V(r)	H(r)
Sn1-Sn70	0.039195	+0.012692	0.430388	+0.011312	−0.019451	−0.008139
Sn1-Cs2	0.011311	+0.017702	1.514955	+0.004062	−0.004054	+0.000008
Cs2-Sn70	0.011147	+0.017264	1.847060	+0.003955	−0.003938	+0.000017
Sn1-Cs71	0.011147	+0.017264	1.847060	+0.003955	−0.003938	+0.000017
Sn70-Cs71	0.011311	+0.017702	1.514955	+0.004062	−0.004054	+0.000008
Cs2-C18	0.010777	+0.035982	9.248644	+0.007328	−0.006181	+0.001147
Cs2-C116	0.010817	+0.036023	7.369217	+0.007333	−0.006183	+0.001150
C47-Cs71	0.010817	+0.036023	7.369217	+0.007333	−0.006183	+0.001150
Cs71-C87	0.010777	+0.035982	9.248644	+0.007328	−0.006181	+0.001147

Second Order Perturbation Theory Analysis of Donor Acceptor NBO interactions between molecular fragments in $[LSnM]_2$ (M= Li, Na, K, Rb and Cs)

M = Li

Table S20. Selected donor–acceptor NBO interaction energies, $E^{(2)}$, in kcal mol^{-1} for **$[LSnLi]_2$, 1^{Li}**.

Donor Orbital	Acceptor Orbital	$E^{(2)}$ kcal mol^{-1}
81. LP(1) Sn1	243. LV(1) Li73	8.3
82. LP(1) Sn2	243. LV(1) Li73	8.3
83. BD(1) Sn1-Sn2	243. LV(1) Li73	5.0
84. BD(2) Sn1-Sn2	243. LV(1) Li73	0.7
81. LP(1) Sn1	244. LV(1) Li138	9.3
82. LP(1) Sn2	244. LV(1) Li138	9.3
83. BD(1) Sn1-Sn2	244. LV(1) Li138	2.3
84. BD(2) Sn1-Sn2	244. LV(1) Li138	2.0

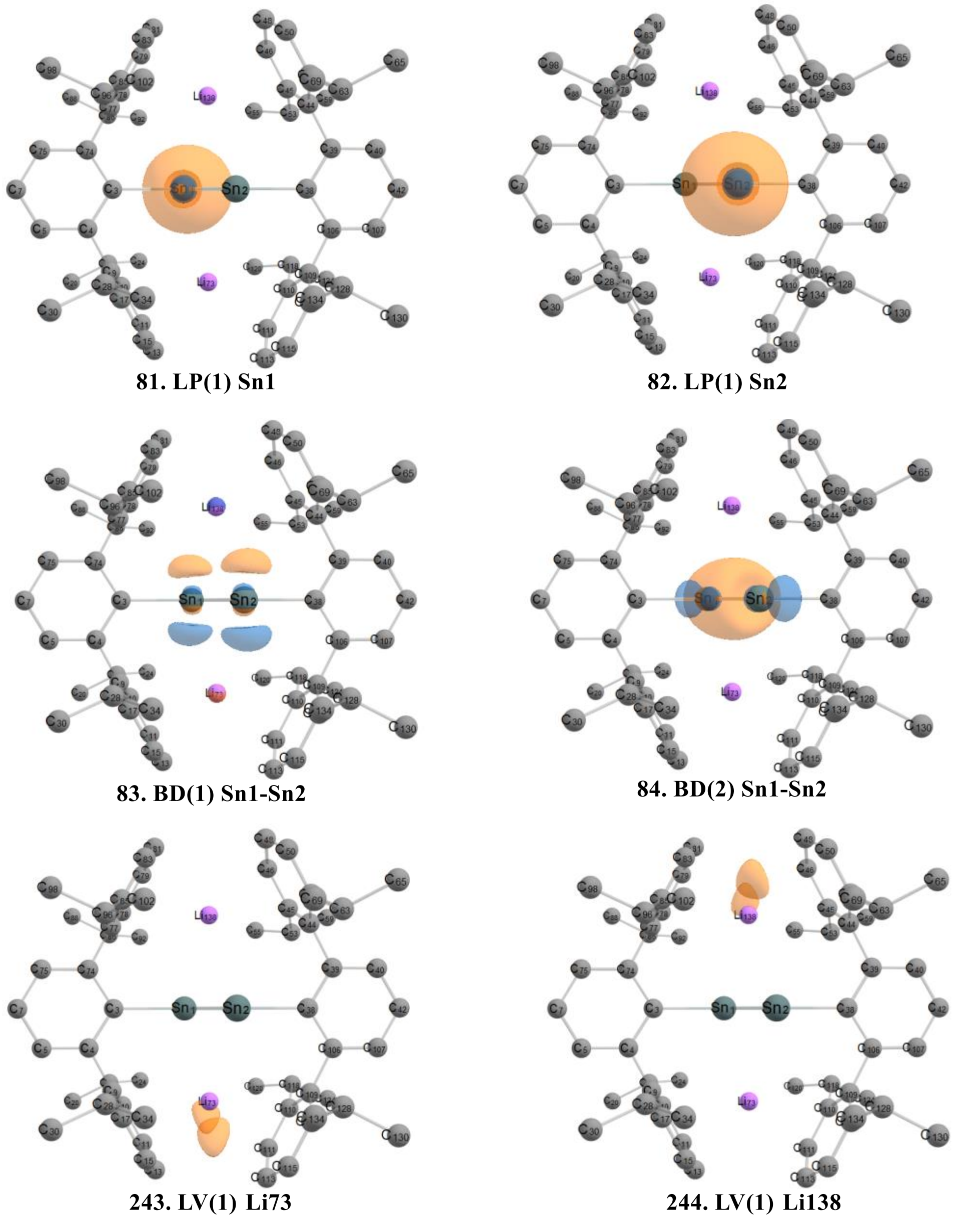

Figure S36. Selected NBO orbitals for compound **[LSnLi]**$_2$, **1^{Li}.**

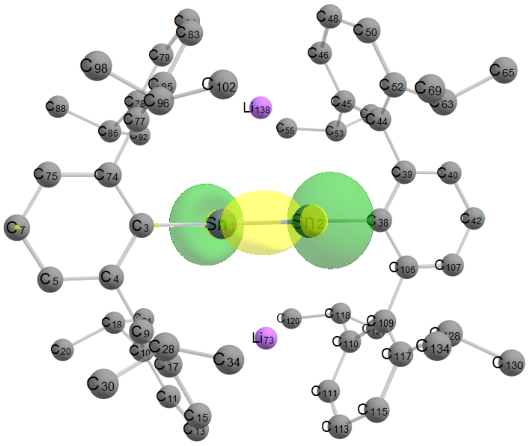

HOMO
-3.04 eV

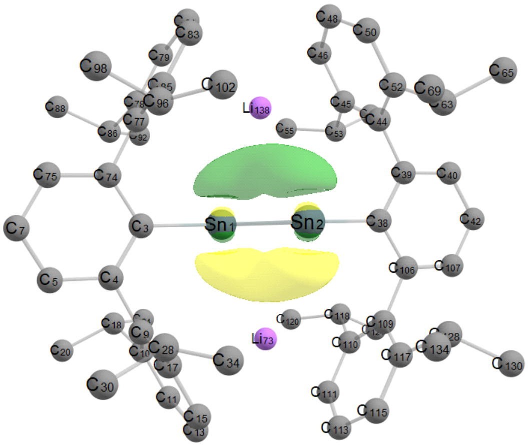

HOMO-1
-3.60 eV

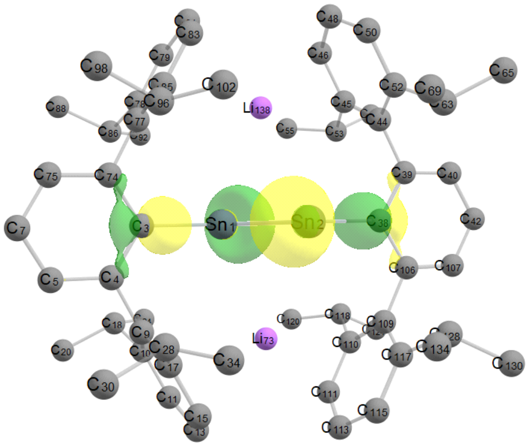

HOMO-2
-4.96 eV

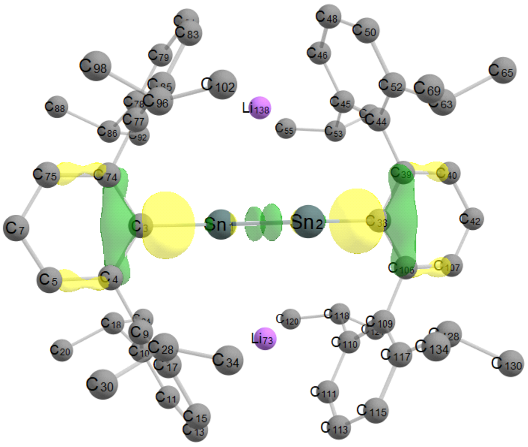

HOMO-3
-4.96 eV

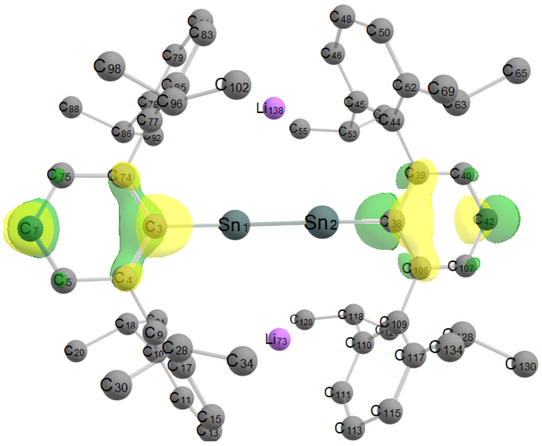

HOMO-4
-5.69 eV

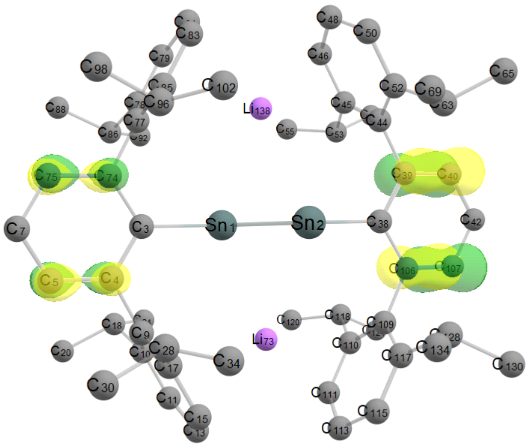

HOMO-5
-5.73 eV

Figure S37. Molecular orbitals HOMO-5 to HOMO for compound **[LSnLi]$_2$, 1^{Li}.**

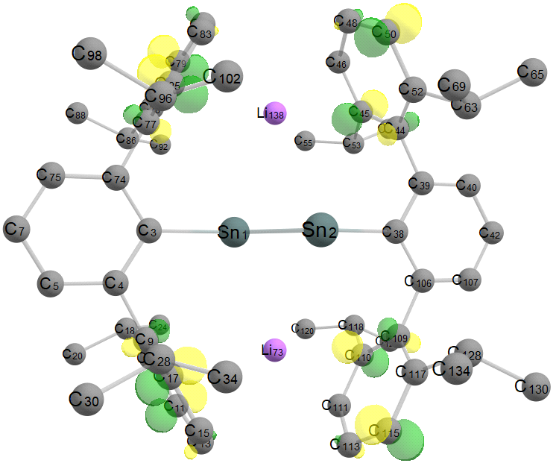

LUMO+4
-1.59 eV

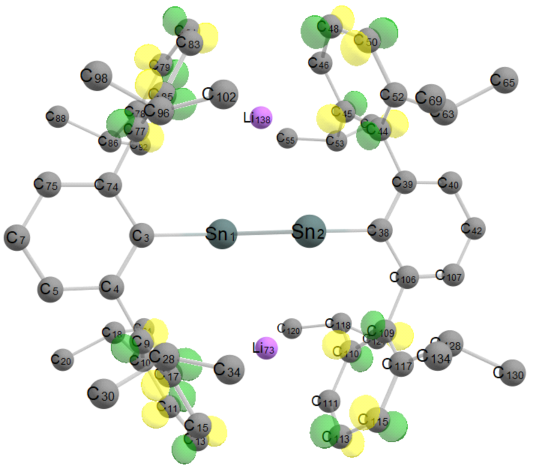

LUMO+5
-1.41 eV

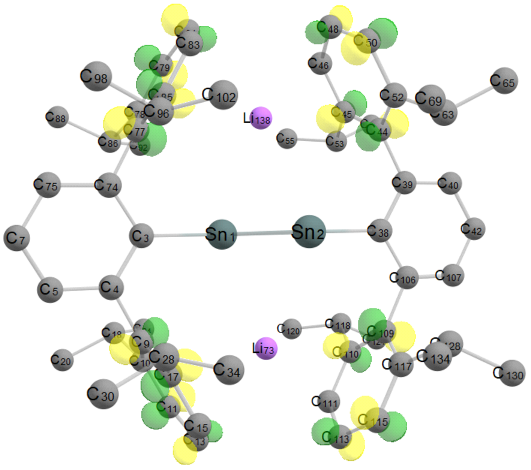

LUMO+2
-1.69 eV

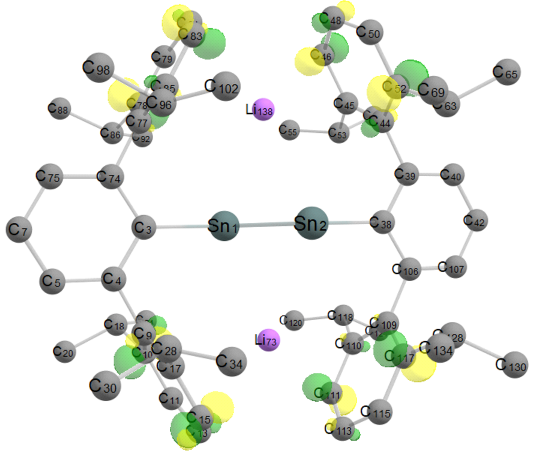

LUMO+3
-1.62 eV

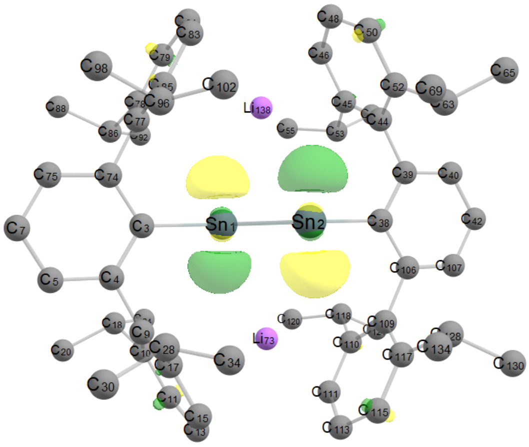

LUMO
-2.01 eV

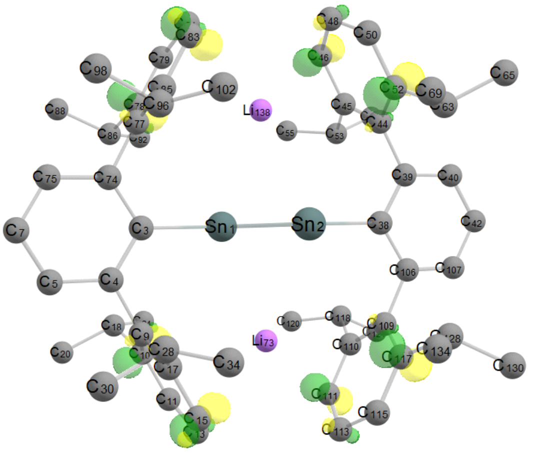

LUMO+1
-1.74 eV

Figure S38. Molecular orbitals LUMO to LUMO+5 for compound **[LSnLi]$_2$, 1^{Li}**.

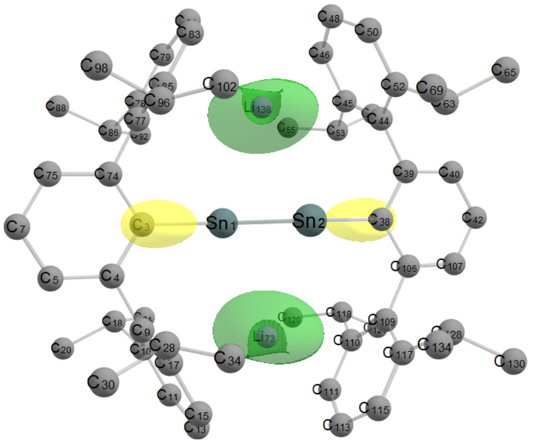

LUMO+13
-0.26 eV

Figure S39. Lowest unoccupied molecular orbital involving the Li atoms.

M = Na

Table S21. Selected donor - acceptor interaction energies, $E^{(2)}$, in kcal mol^{-1} for **$[LSnNa]_2$, 1^{Na}**.

Donor Orbital	Acceptor Orbital	$E^{(2)}$ kcal/mol
89. LP(1) Sn1	251. LV(1) Na2	5.1
90. LP(1) Sn70	251. LV(1) Na2	5.1
92. BD(1) Sn1-Sn70	251. LV(1) Na2	5.3
93. BD(2) Sn1-Sn70	251. LV(1) Na2	0.8
89. LP(1) Sn1	252. LV(1) Na71	5.1
90. LP(1) Sn70	252. LV(1) Na71	5.1
92. BD(1) Sn1-Sn70	252. LV(1) Na71	5.3
93. BD(2) Sn1-Sn70	252. LV(1) Na71	0.8

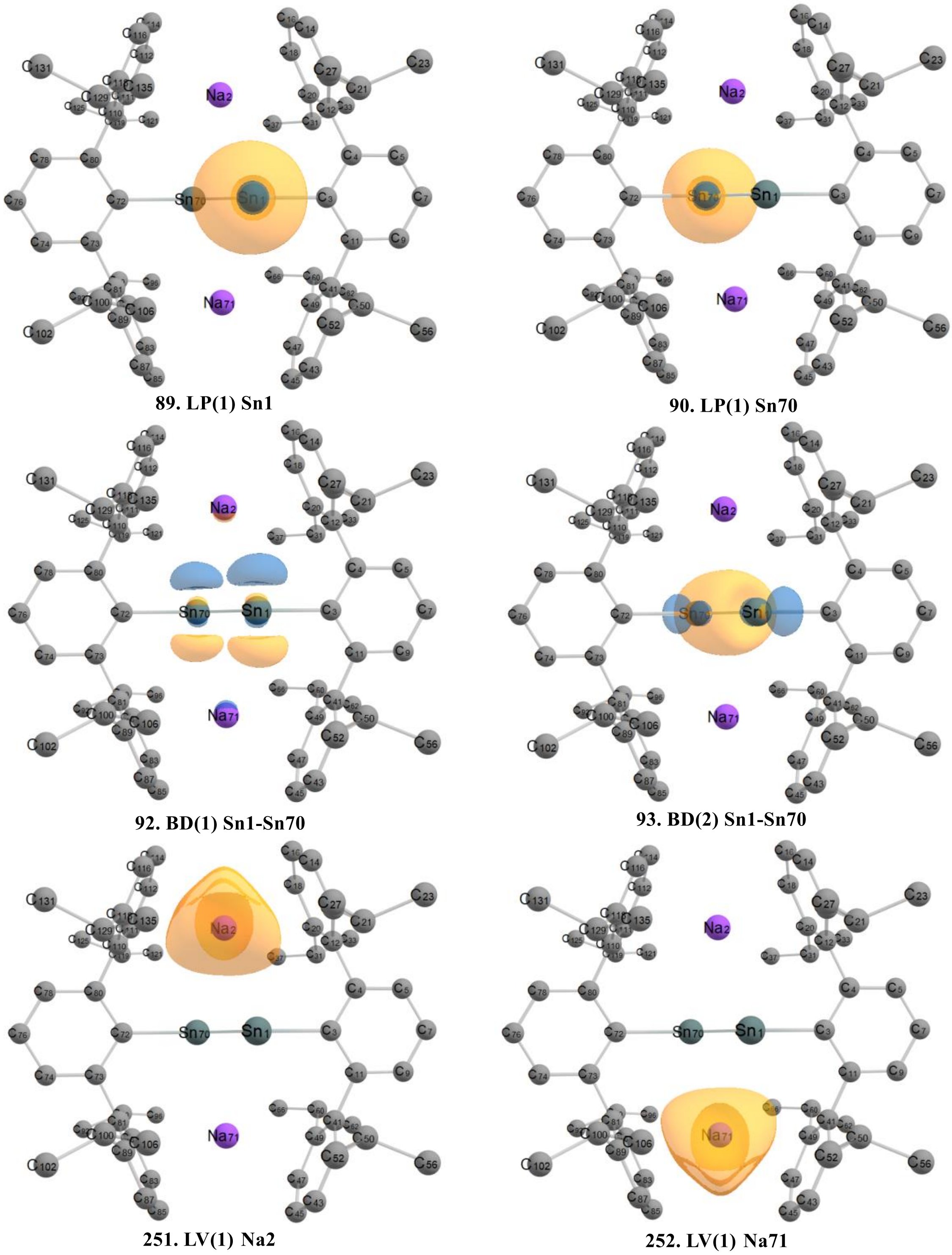

Figure S40. NBOs for compound **[LSnNa]$_2$, 1^{Na}.**

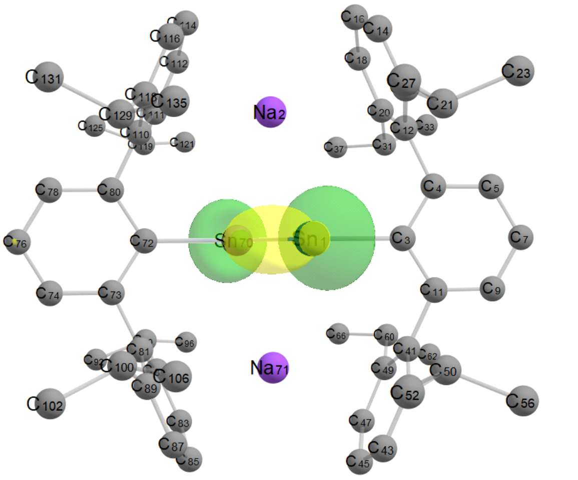

HOMO
-2.99 eV

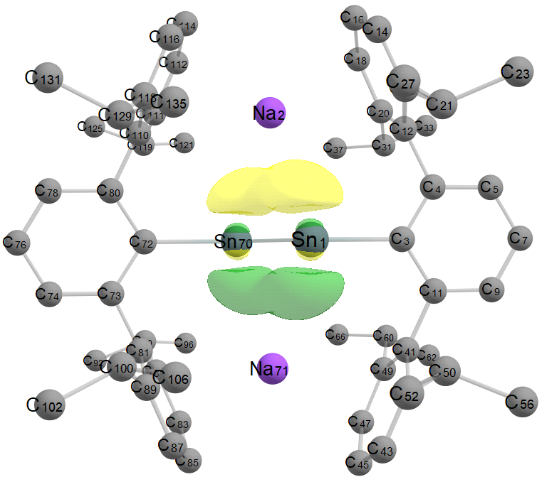

HOMO-1
-3.52 eV

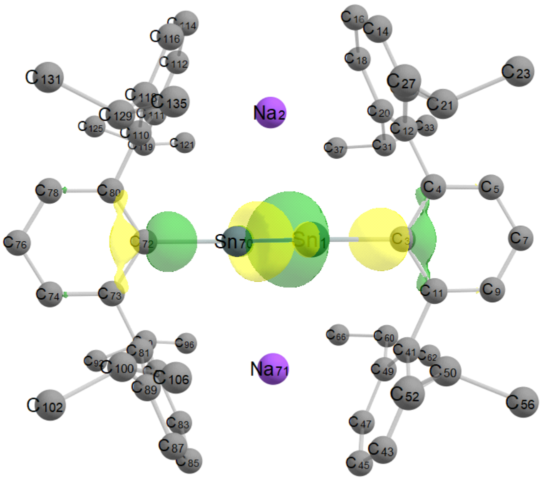

HOMO-2
-4.93 eV

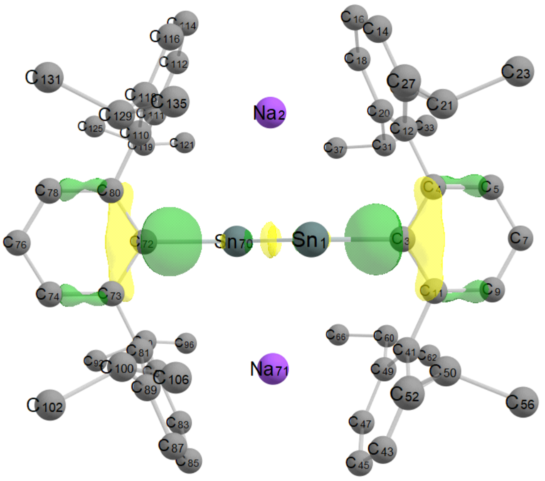

HOMO-3
-4.98 eV

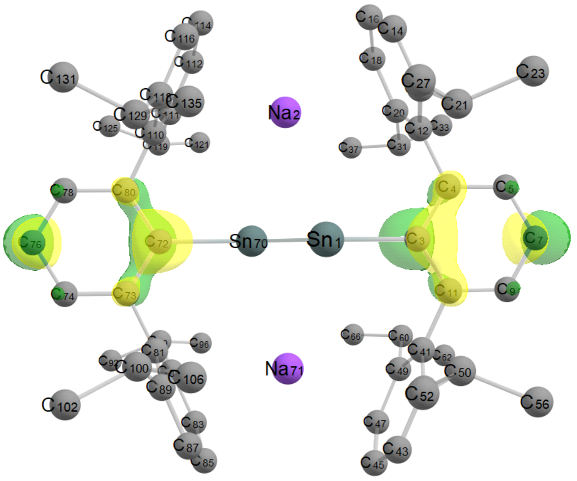

HOMO-4
-5.69 eV

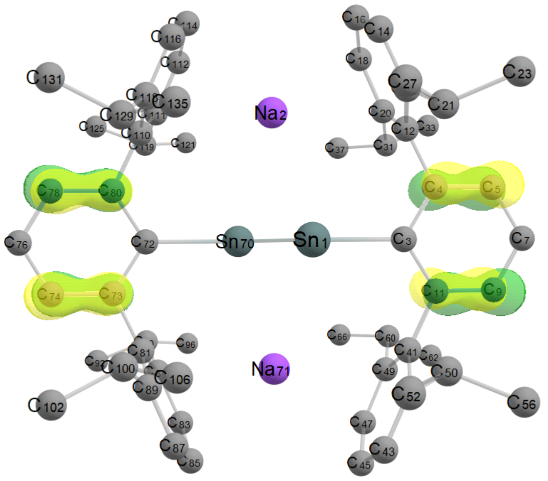

HOMO-5
-5.70 eV

Figure S41. Molecular orbitals HOMO-5 to HOMO for compound **[LSnNa]$_2$, 1^{Na}**.

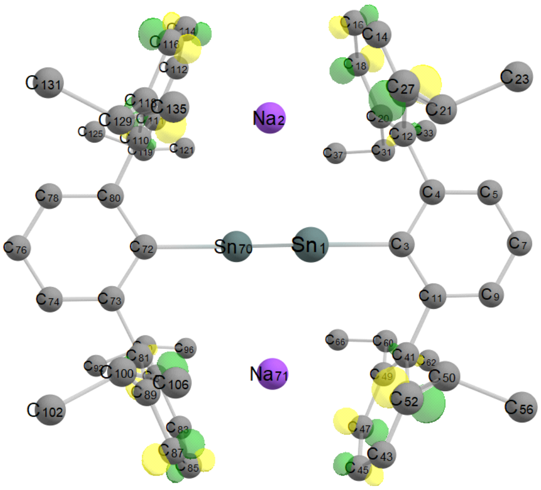

LUMO+4
-1.51 eV

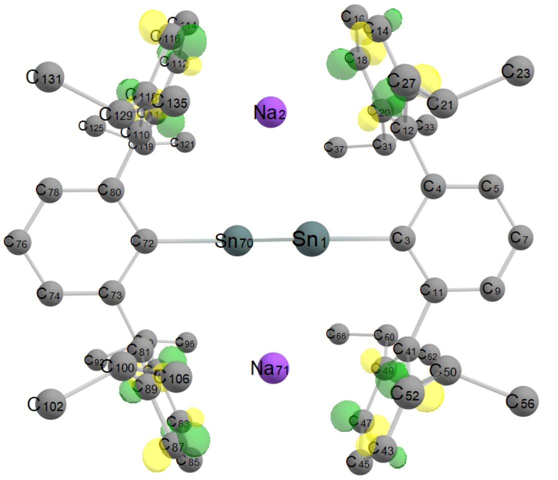

LUMO+5
-1.45 eV

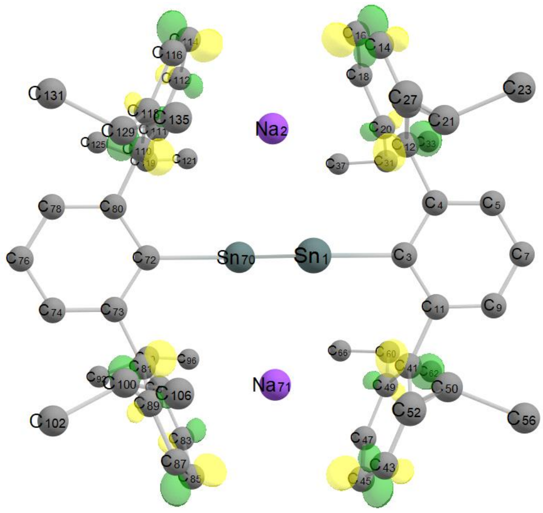

LUMO+2
-1.66 eV

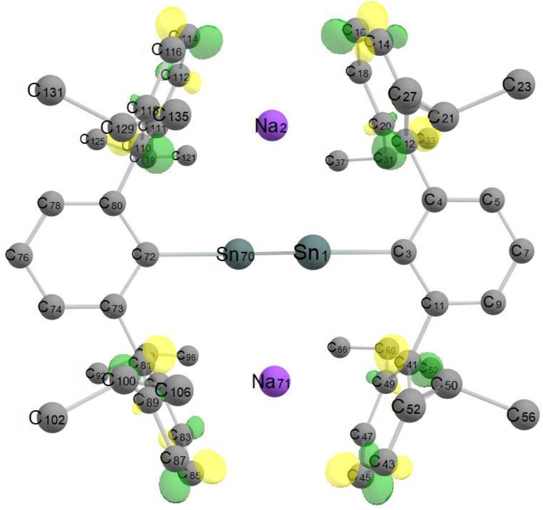

LUMO+3
-1.54 eV

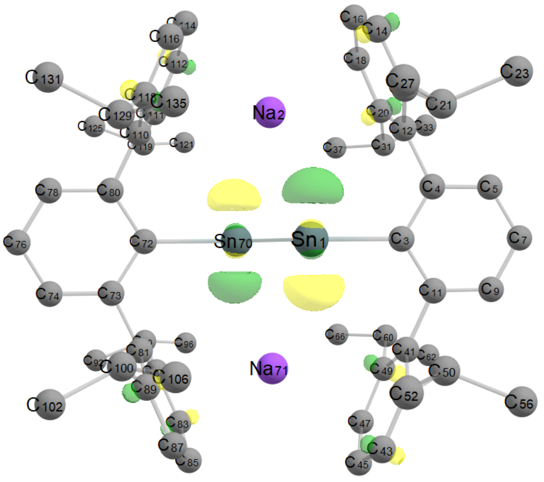

LUMO
-1.96 eV

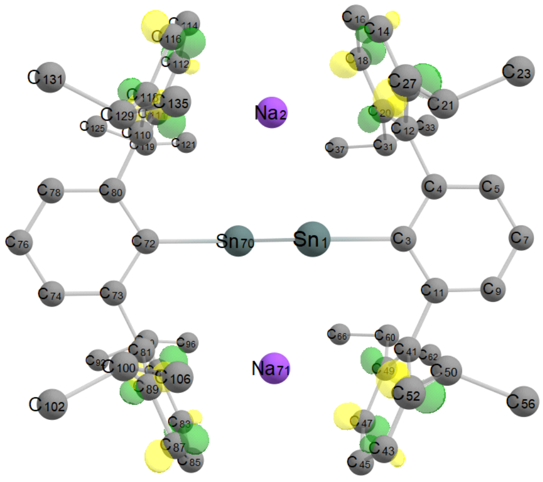

LUMO+1
-1.68 eV

Figure S42. Molecular orbitals LUMO to LUMO+5 for compound **$[LSnNa]_2$, 1^{Na}**.

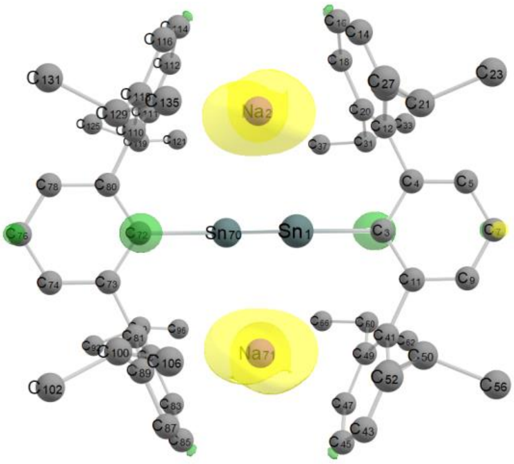

LUMO+13
-0.56 eV

Figure S43. Lowest unoccupied molecular orbital involving the Na atoms.

M = K

Table S22. Selected donor - acceptor interaction energies, $E^{(2)}$, in kcal mol^{-1} for **[LSnK]$_2$**, **1^K**.

Donor Orbital	Acceptor Orbital	$E^{(2)}$ kcal/mol
97. LP(1) Sn1	259. LV(1) K2	3.7
98. LP(1) Sn70	259. LV(1) K2	3.5
100. BD(1) Sn1-Sn70	259. LV(1) K2	6.5
101. BD(2) Sn1-Sn70	259. LV(1) K2	0.7
97. LP(1) Sn1	260. LV (1) K71	3.5
98. LP(1) Sn70	260. LV (1) K71	3.7
100. BD(1) Sn1-Sn70	260. LV (1) K71	6.5
101. BD(2) Sn1-Sn70	260. LV (1) K71	0.7

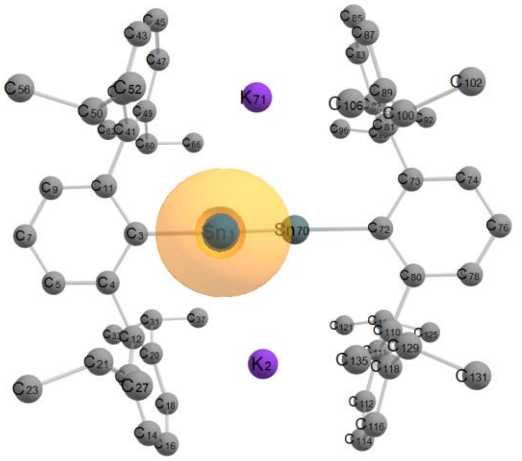

97. LP(1) Sn1

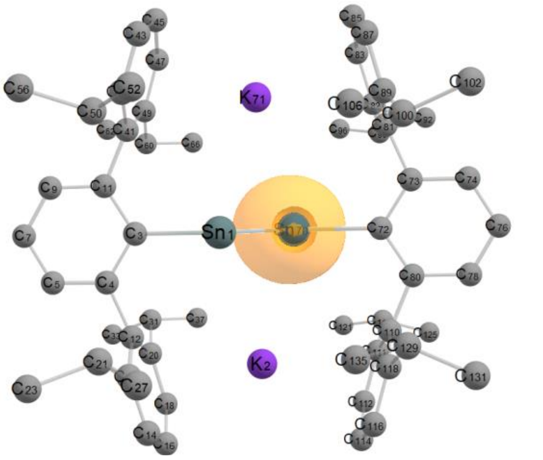

98. LP(1) Sn70

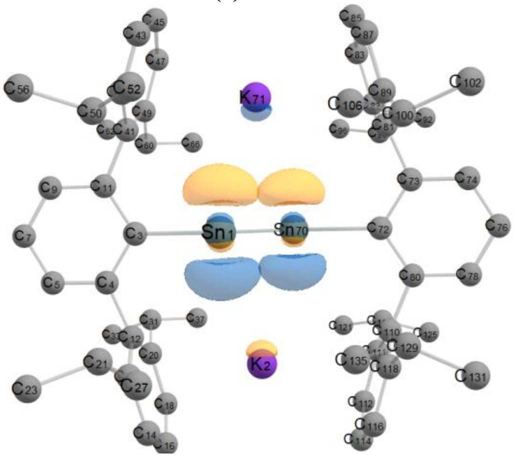

100. BD(1) Sn1-Sn70

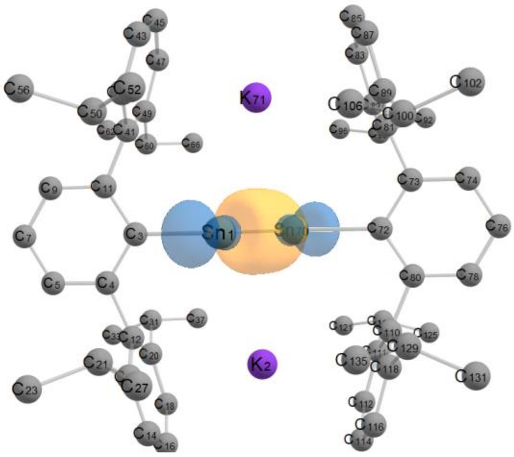

101. BD(2) Sn1-Sn70

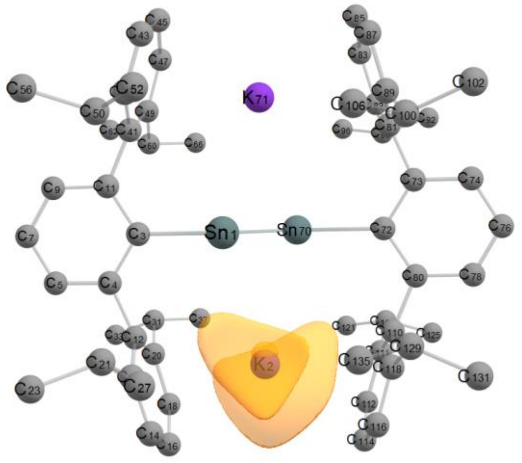

259. LV(1) K2

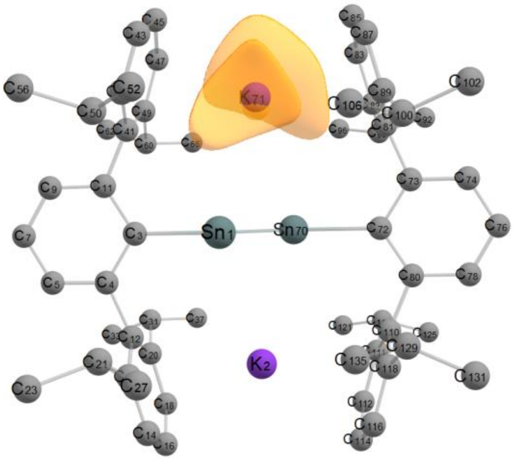

260. LV(1) K71

Figure S44. NBOs for compound **[LSnK]$_2$**, **1^K**.

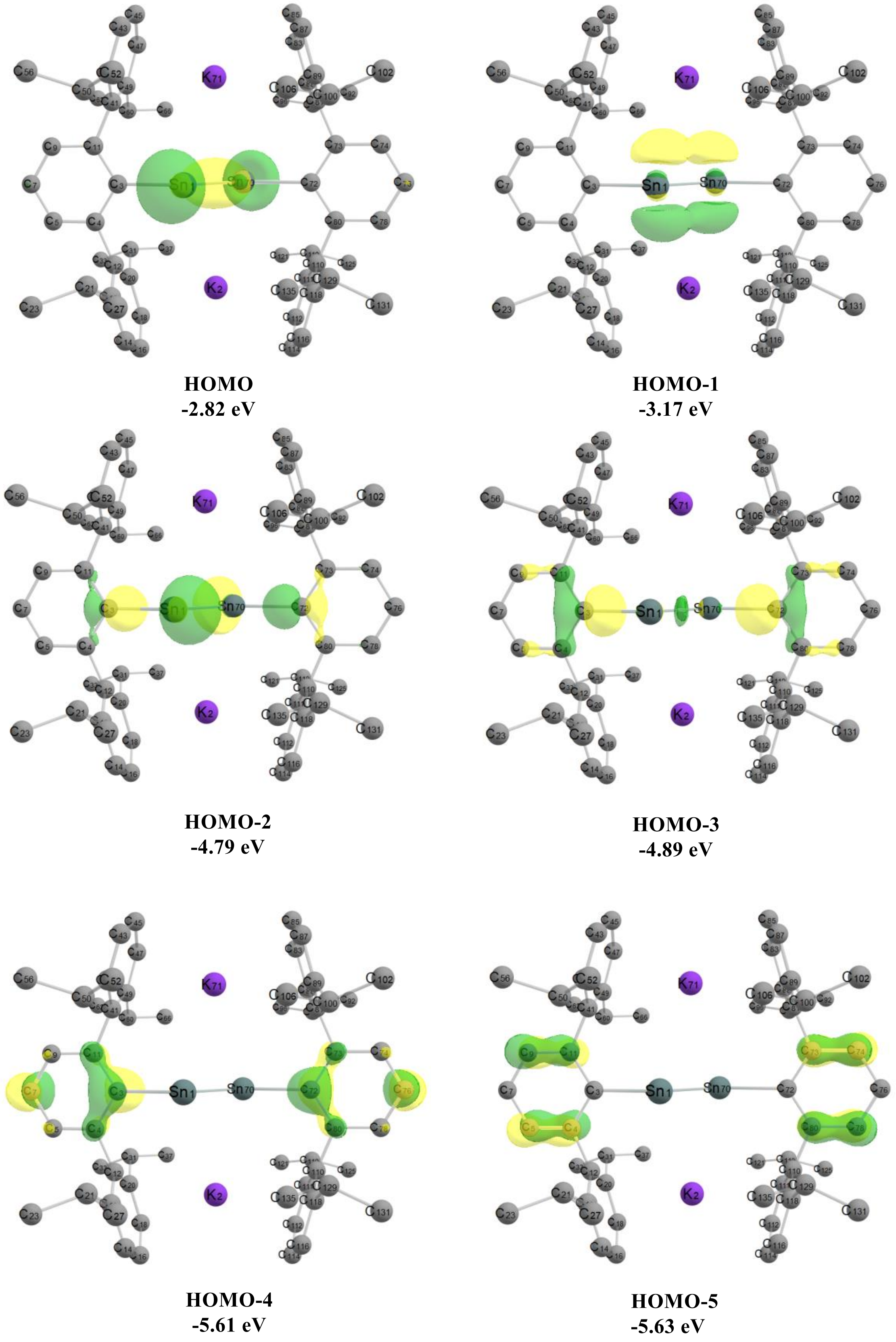

Figure S45. Molecular orbitals HOMO-5 to HOMO for compound **[LSnK]$_2$, 1^K.**

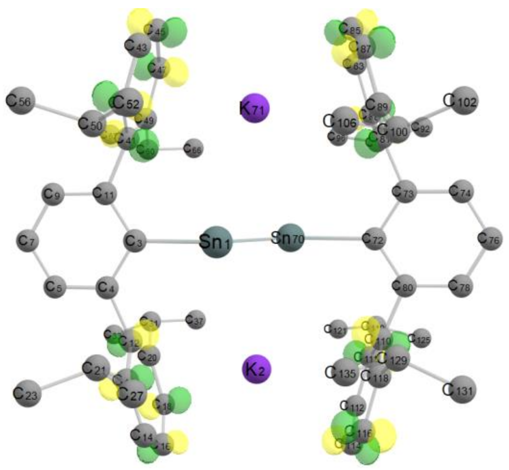

LUMO+4
-1.47 eV

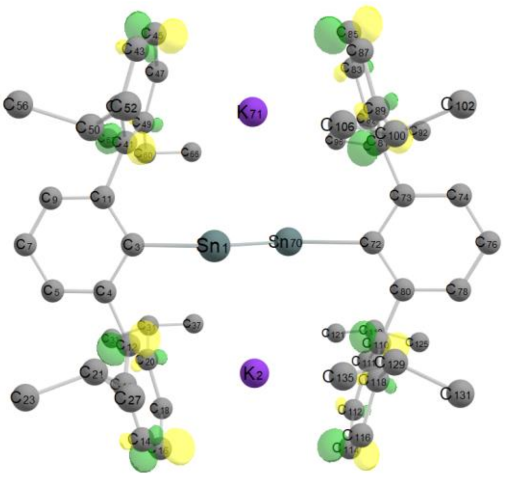

LUMO+5
-1.46 eV

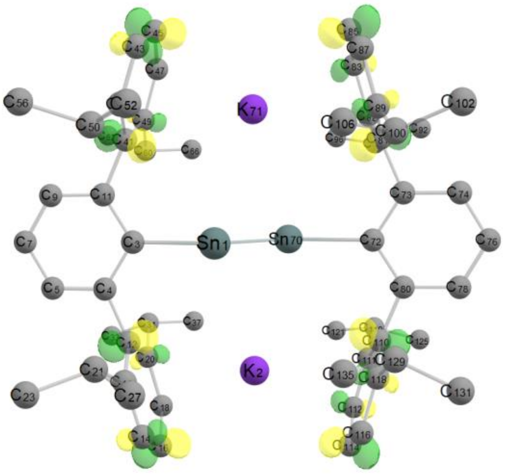

LUMO+2
-1.59 eV

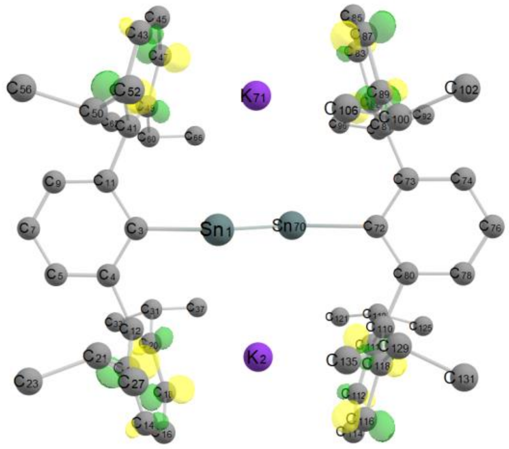

LUMO+3
-1.49 eV

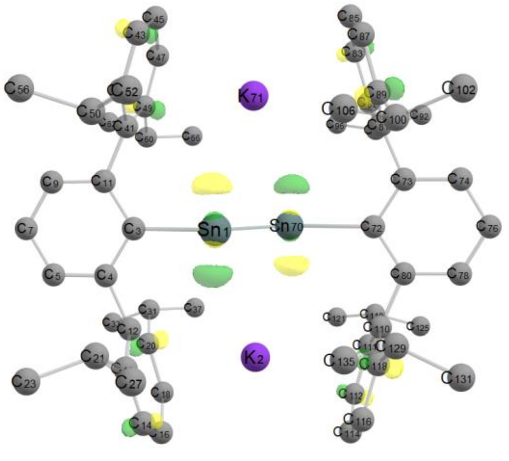

LUMO
-1.90 eV

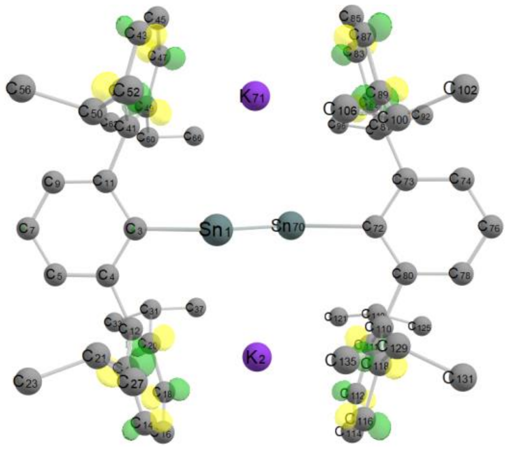

LUMO+1
-1.65 eV

Figure S46. Molecular orbitals LUMO to LUMO+5 for compound **[LSnK]$_2$, 1^K**.

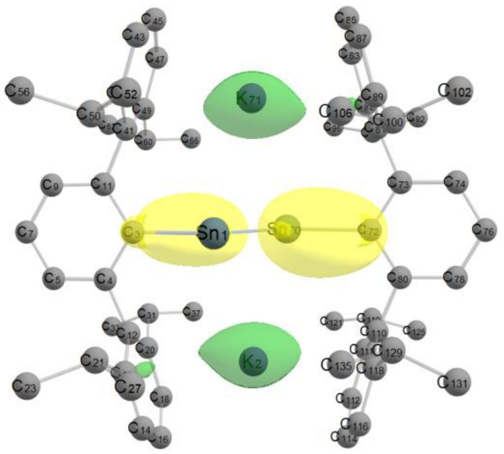

LUMO+19
0.31 eV

Figure S47. Lowest unoccupied molecular orbitals involving the K atoms.

M = Rb

Table S23. Selected donor - acceptor interaction energies, $E^{(2)}$, in kcal mol^{-1} for **[LSnRb]$_2$**, **1^{Rb}**.

Donor Orbital	Acceptor Orbital	E(2) kcal/mol
87. LP(1) Sn1	249. LV(1) Rb2	2.9
88. LP(1) Sn70	249. LV(1) Rb2	2.7
90. BD(1) Sn1-Sn70	249. LV(1) Rb2	1.4
91. BD(2) Sn1-Sn70	249. LV(1) Rb2	19.1
87. LP(1) Sn1	250. LV(1) Rb71	2.7
88. LP(1) Sn70	250. LV(1) Rb71	2.9
90. BD(1) Sn1-Sn70	250. LV(1) Rb71	1.4
91. BD(2) Sn1-Sn70	250. LV(1) Rb71	19.1

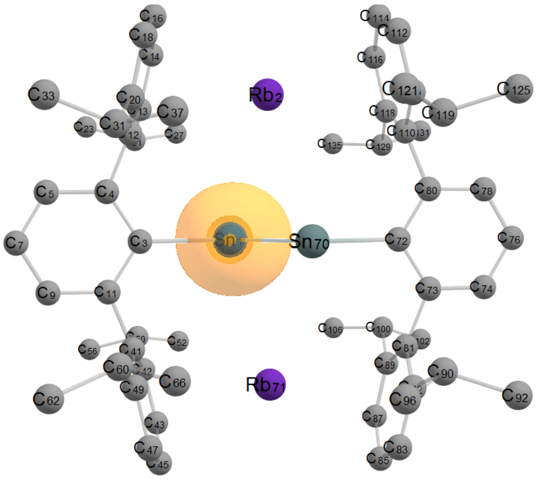

87. LP(1) Sn1

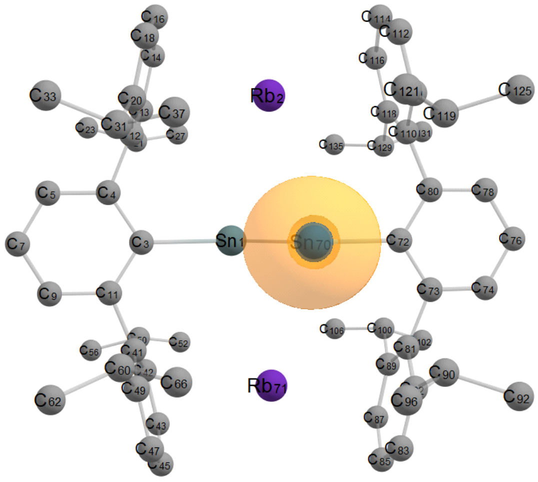

88. LP(1) Sn70

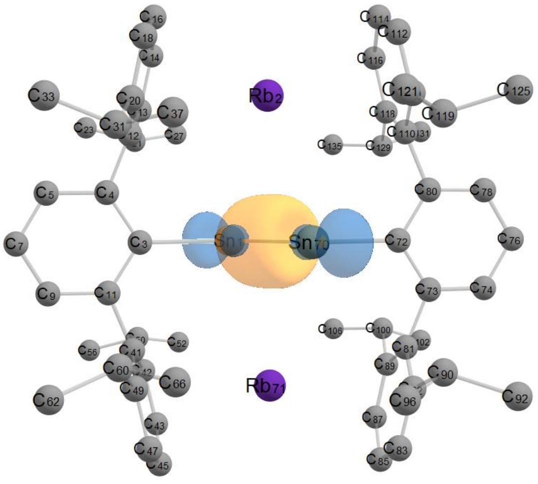

90. BD(1) Sn1-Sn70

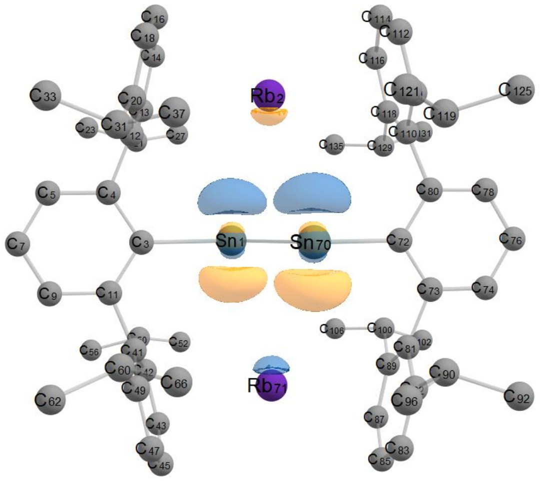

91. BD(2) Sn1-Sn70

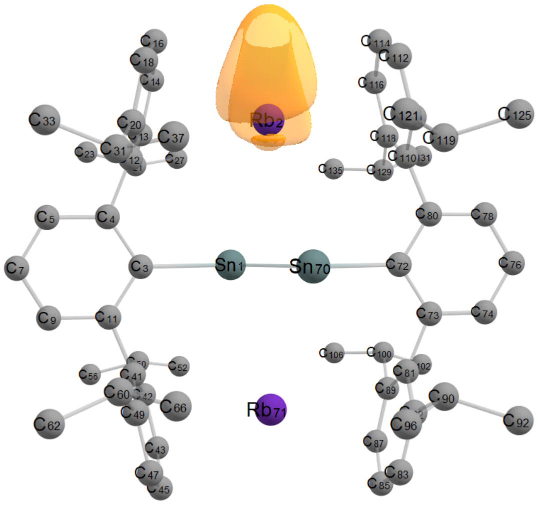

249. LV(1) Rb2

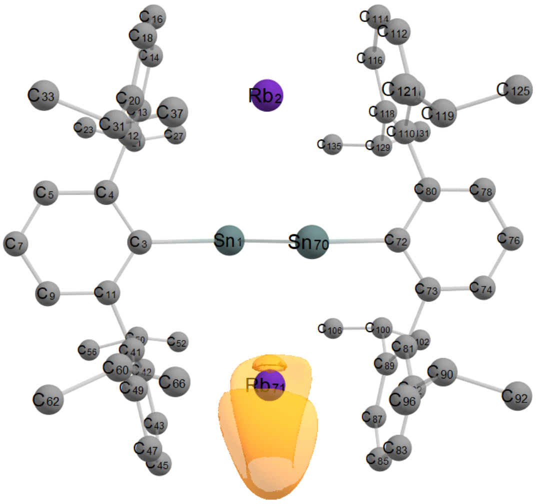

250. LV(1) Rb271

Figure S48. NBOs for compound **[LSnRb]**$_2$, **1**$^{\mathbf{Rb}}$**.**

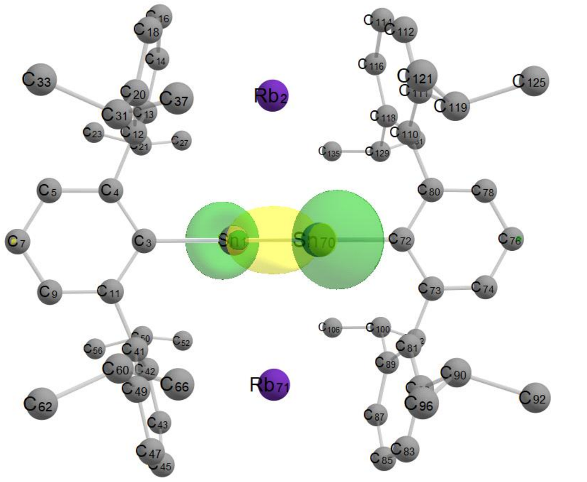

HOMO
-2.77 eV

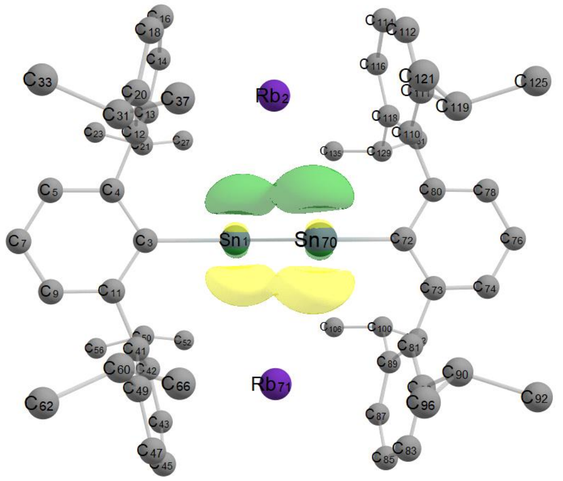

HOMO-1
-3.11 eV

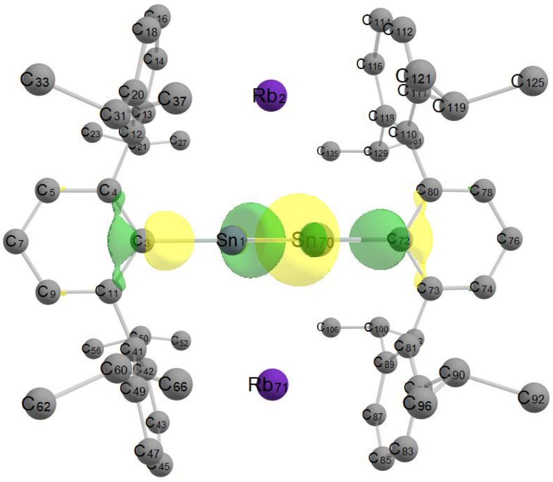

HOMO-2
-4.74 eV

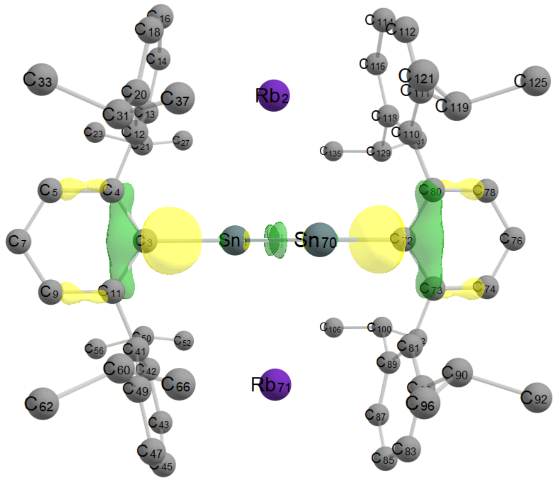

HOMO-3
-4.90 eV

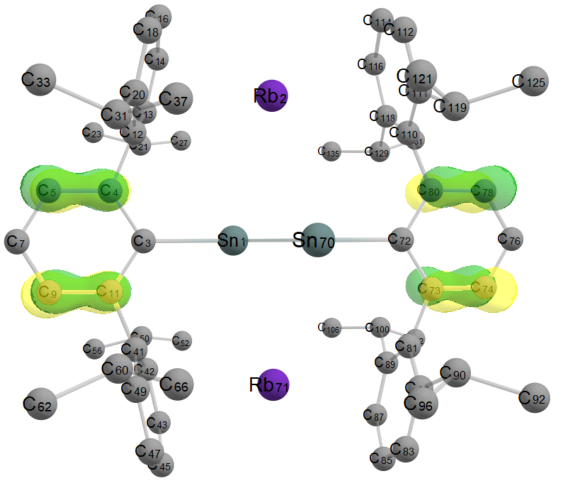

HOMO-4
-5.60 eV

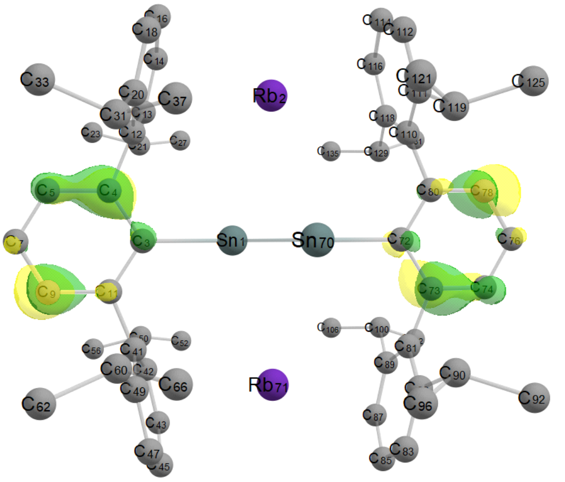

HOMO-5
-5.60 eV

Figure S49. Molecular orbitals HOMO-5 to HOMO for compound **[LSnRb]$_2$, 1^{Rb}.**

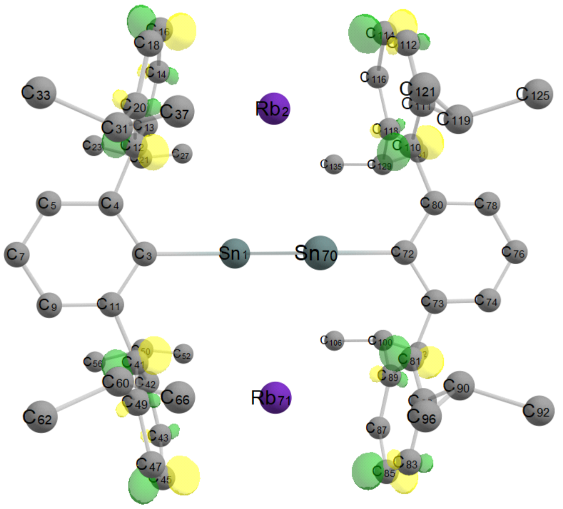

LUMO+4
-1.46 eV

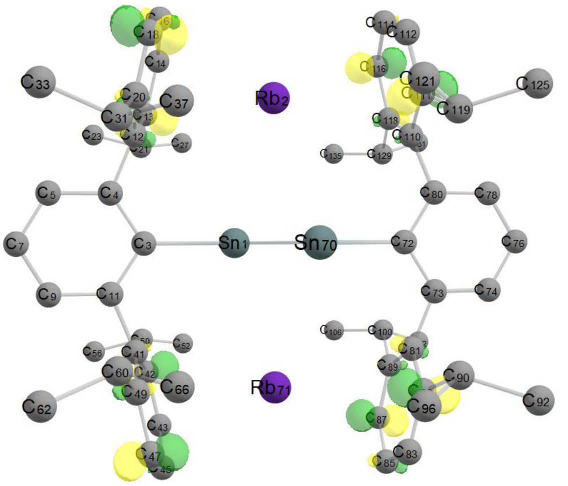

LUMO+5
-1.44 eV

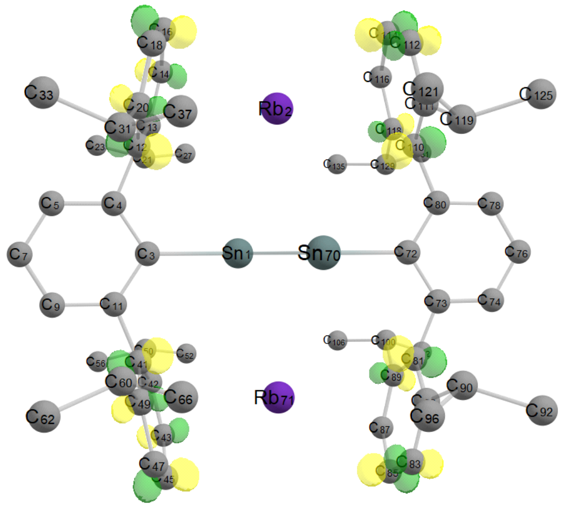

LUMO+2
-1.54 eV

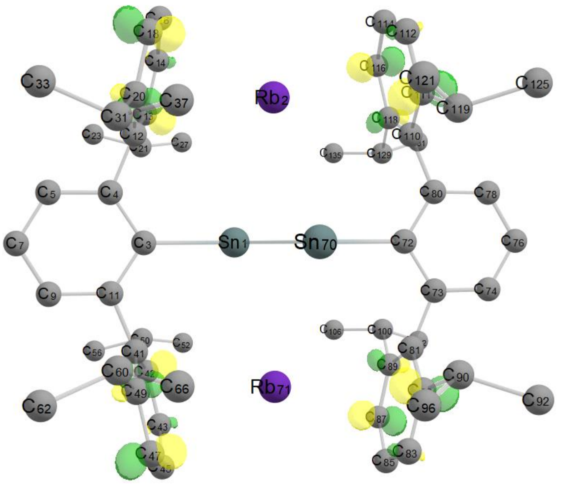

LUMO+3
-1.48 eV

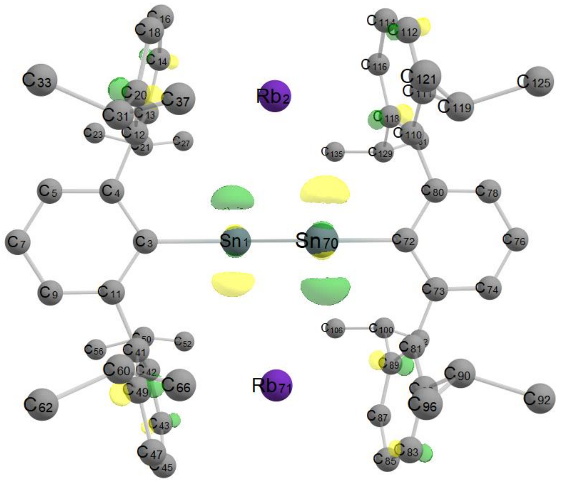

LUMO
-1.87 eV

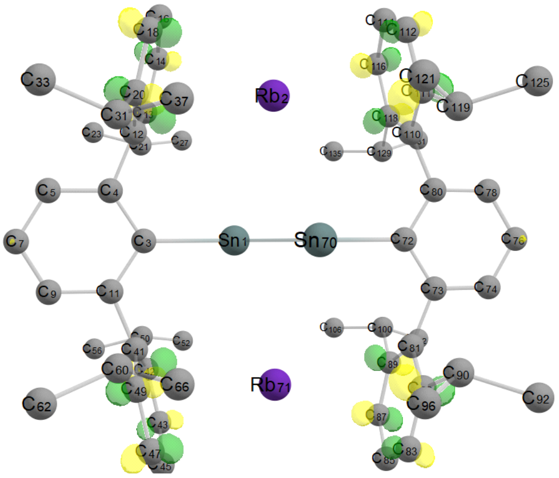

LUMO+1
-1.63 eV

Figure S50. Molecular orbitals LUMO to LUMO+5 for compound **[LSnRb]**$_2$, **1**$^{\mathbf{Rb}}$.

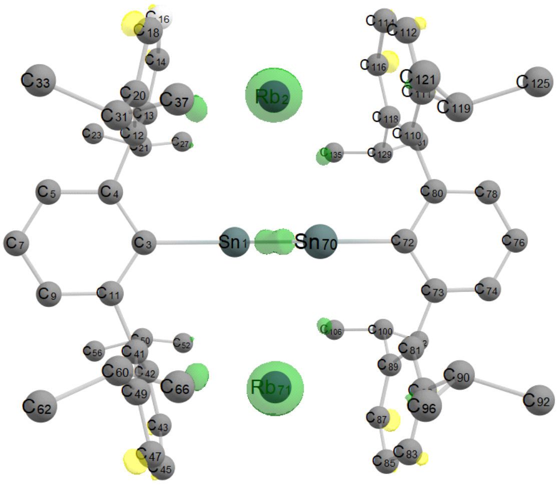

LUMO+26
0.28 eV

Figure S51. Most accessible LUMO located at Rb atoms.

M = Cs

Table S24. Selected donor acceptor interaction energies, $E^{(2)}$, in kcal mol^{-1} for **$[LSnCs]_2$, 1^{Cs}**.

Donor Orbital	Acceptor Orbital	$E^{(2)}$ kcal/mol
87. LP(1) Sn1	249. LV(1) Cs2	2.4
88. LP(1) Sn70	249. LV(1) Cs2	2.2
90. BD(1) Sn1-Sn70	249. LV(1) Cs2	1.2
91. BD(2) Sn1-Sn70	249. LV(1) Cs2	26.3
87. LP(1) Sn1	250. LV(1) Cs71	2.2
88. LP(1) Sn70	250. LV(1) Cs71	2.4
90. BD(1) Sn1-Sn70	250. LV(1) Cs71	1.2
91. BD(2) Sn1-Sn70	250. LV(1) Cs71	26.6

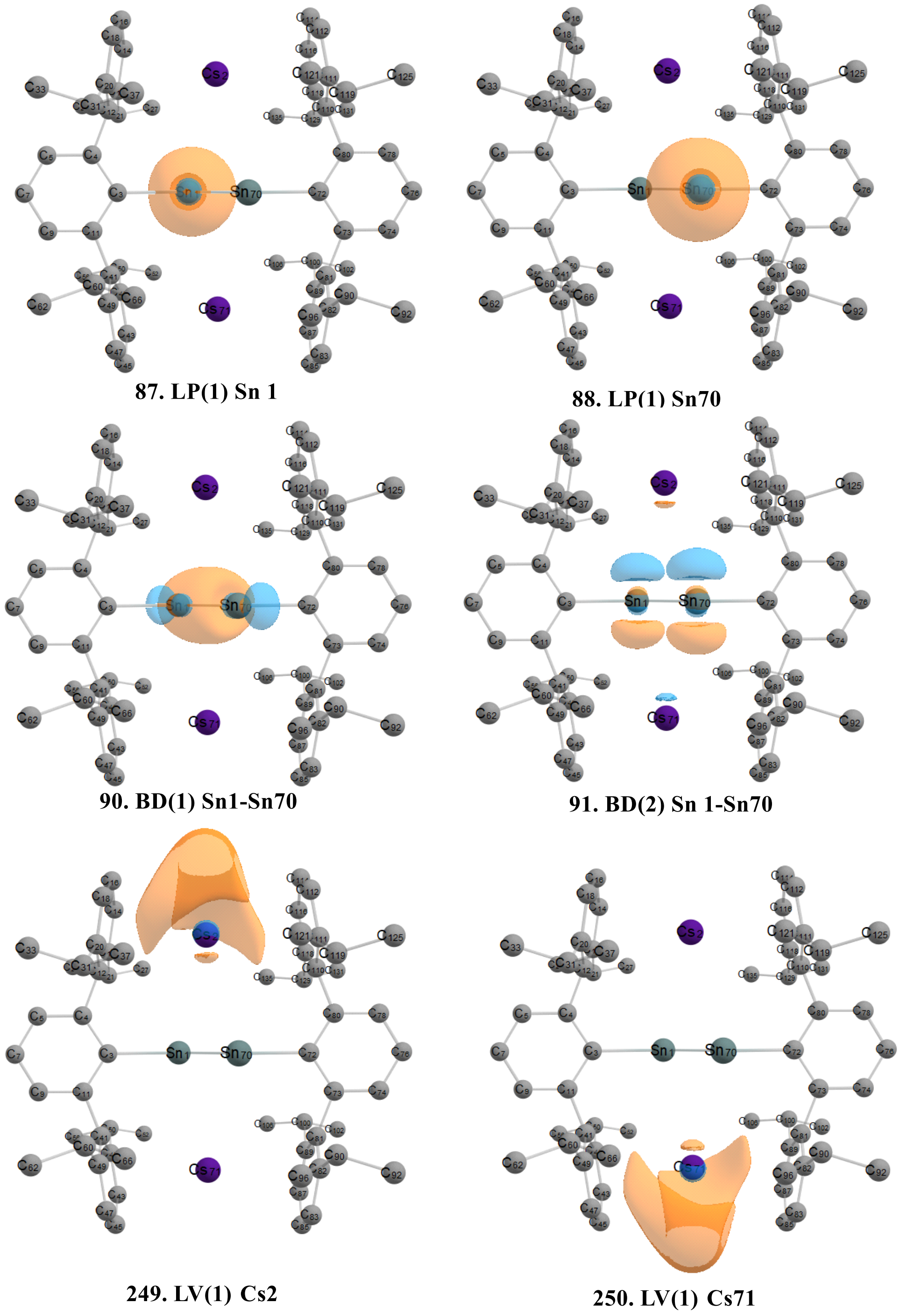

Figure S52. NBOs for compound **[LSnCs]**$_2$, **1**^{Cs}.

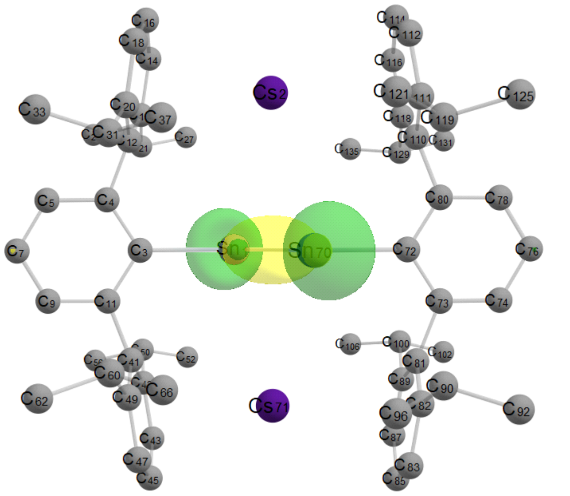

HOMO
-2.74 eV

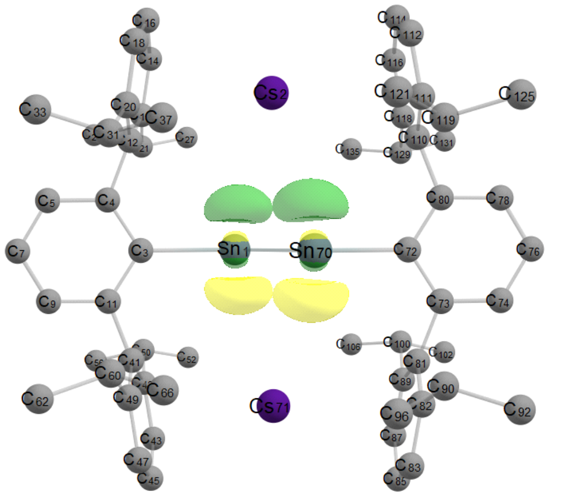

HOMO-1
-3.02 eV

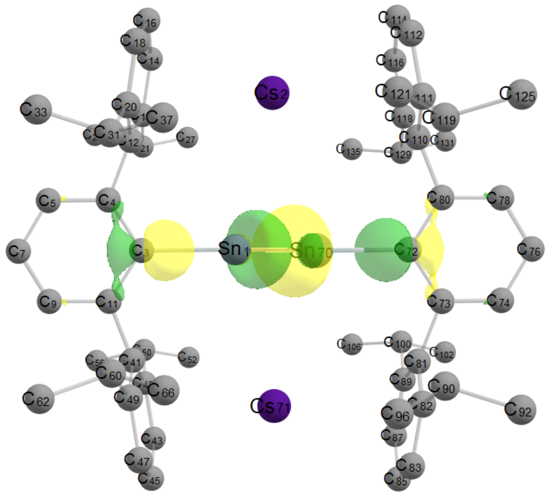

HOMO-2
-4.71 eV

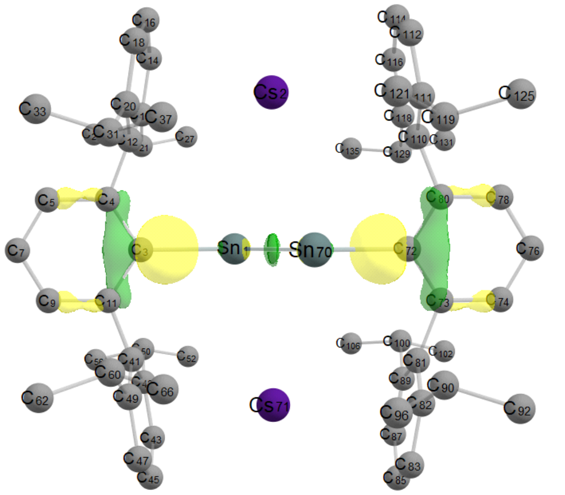

HOMO-3
-4.89 eV

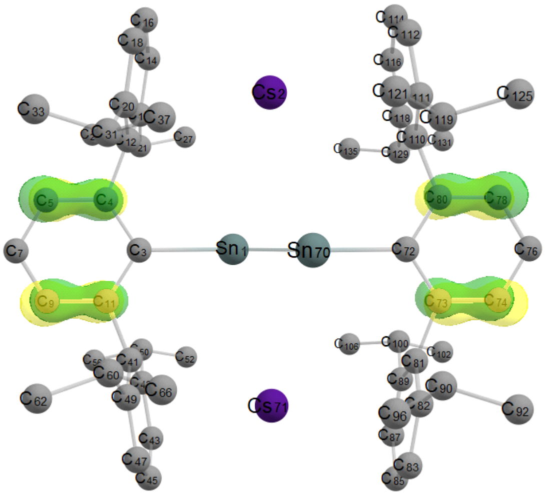

HOMO-4
-5.56 eV

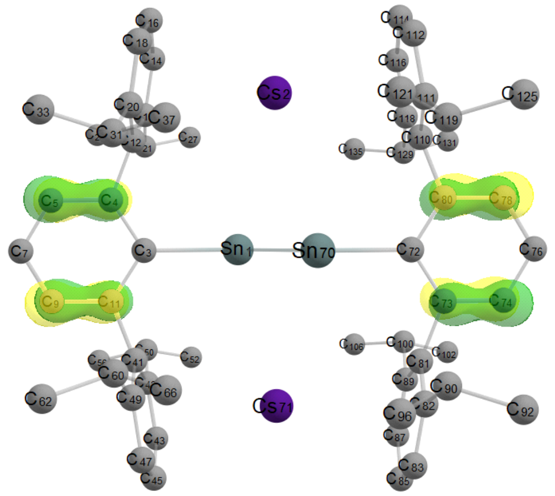

HOMO-5
-5.57 eV

Figure S53. Molecular orbitals HOMO-5 to HOMO for compound **[LSnCs]$_2$, 1^{Cs}.**

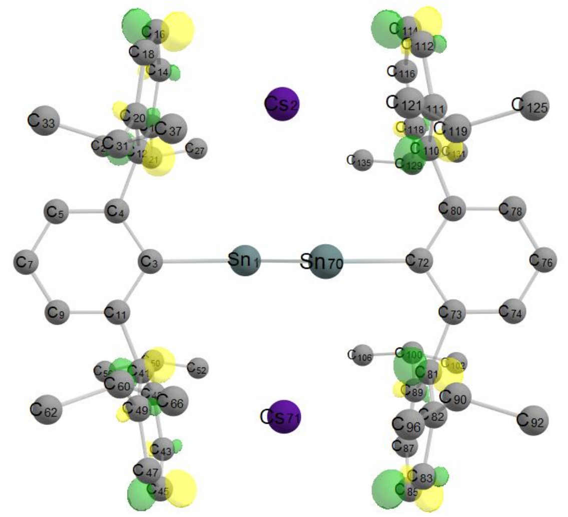

LUMO+4
-1.46 eV

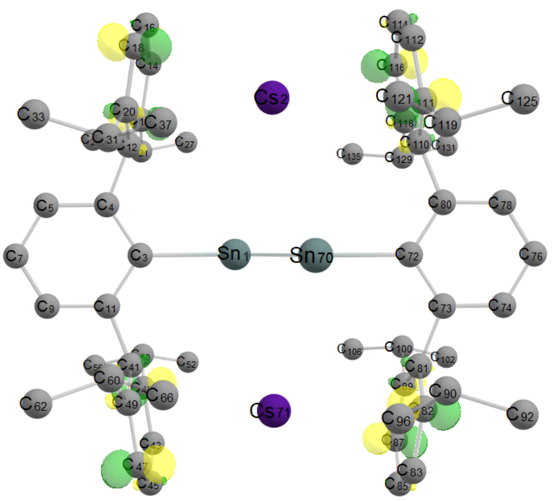

LUMO+5
-1.44 eV

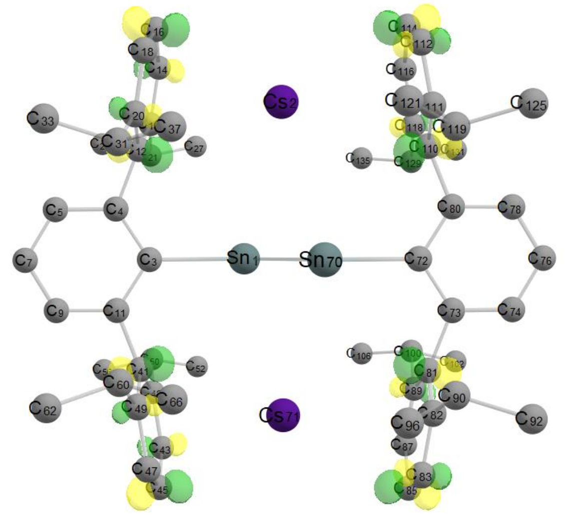

LUMO+2
-1.54 eV

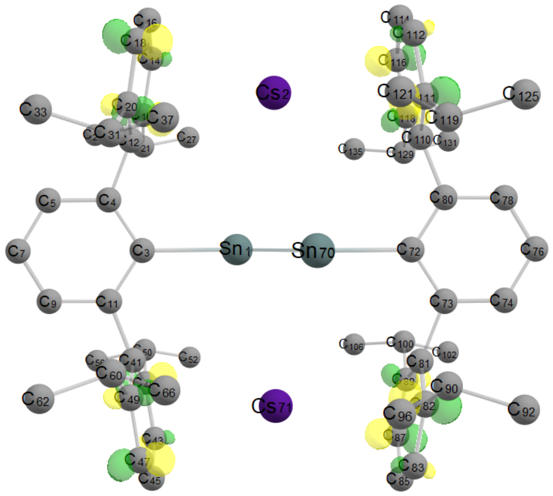

LUMO+3
-1.49 eV

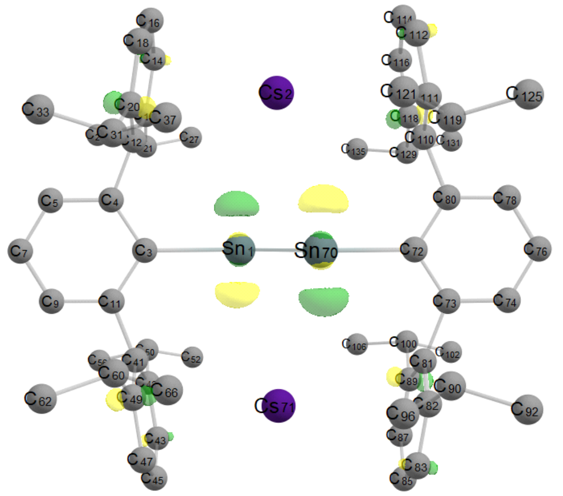

LUMO
-1.91 eV

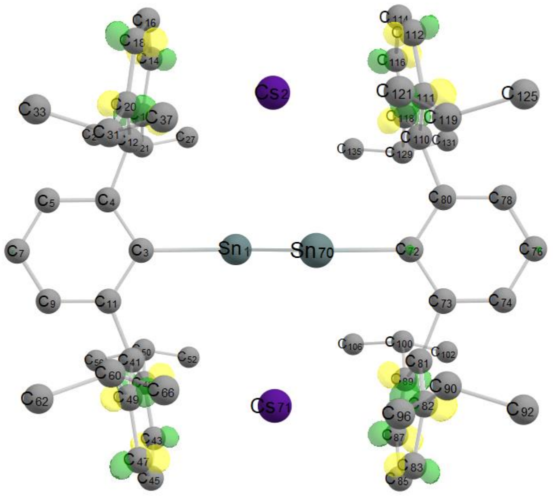

LUMO+1
-1.64 eV

Figure S54. Molecular orbitals LUMO to LUMO+5 for compound **[LSnCs]$_2$, 1^{Cs}.**

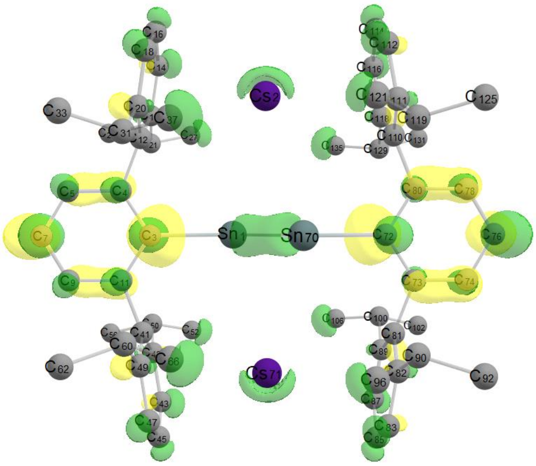

LUMO+12
-0.75 eV

Figure S55. Lowest unoccupied molecular orbital involving the Cs atoms.

Second Order Perturbation Theory Analysis of Donor Acceptor NBO interactions between molecular fragments in $[LSnM]_2$ radical anions (M= Li, Na, K, Rb and Cs) and Mulliken spin density.

M = Li

Table S25. Selected donor – acceptor NBO interaction energies, $E^{(2)}$, in kcal mol^{-1} for **$[LSnLi]_2^{\bullet-}$** radical anion, **$1^{Li\bullet-}$**. (Alpha spin NBOs).

Donor Orbital	Acceptor Orbital	$E^{(2)}$ kcal/mol
81. LP(1) Sn1	244. LV(1) Li73	3.8
82. LP(2) Sn1	244. LV(1) Li73	1.0
83. LP(1) Sn2	244. LV (1) Li73	5.7
84. LP(2) Sn2	244. LV(1) Li73	2.5
81. LP(1) Sn1	245. LV(1) Li138	5.7
82. LP(2) Sn1	245. LV(1) Li138	2.5
83. LP(1) Sn2	245. LV(1) Li138	3.8
84. LP(2) Sn2	245. LV(1) Li138	1.0

Table S26. Selected donor – acceptor NBO interaction energies, $E^{(2)}$, in kcal mol^{-1} for **[LSnLi]$_2$$^{\bullet-}$** radical anion, **1$^{Li\bullet-}$**. (Beta spin NBOs).

Donor Orbital	Acceptor Orbital	E$^{(2)}$ kcal/mol
81. LP(1) Sn1	243. LV(1) Li73	3.9
82. LP(1) Sn2	243. LV(1) Li73	5.0
83. BD(1) Sn1-Sn2	243. LV(1) Li73	3.2
84. BD(2) Sn1-Sn2	243. LV(1) Li73	0.8
81. LP(1) Sn1	244. LV(1) Li138	5.0
82. LP(1) Sn2	244. LV(1) Li138	3.9
83. BD(1) Sn1-Sn2	244. LV(1) Li138	3.2
84. BD(2) Sn1-Sn2	244. LV(1) Li138	0.8

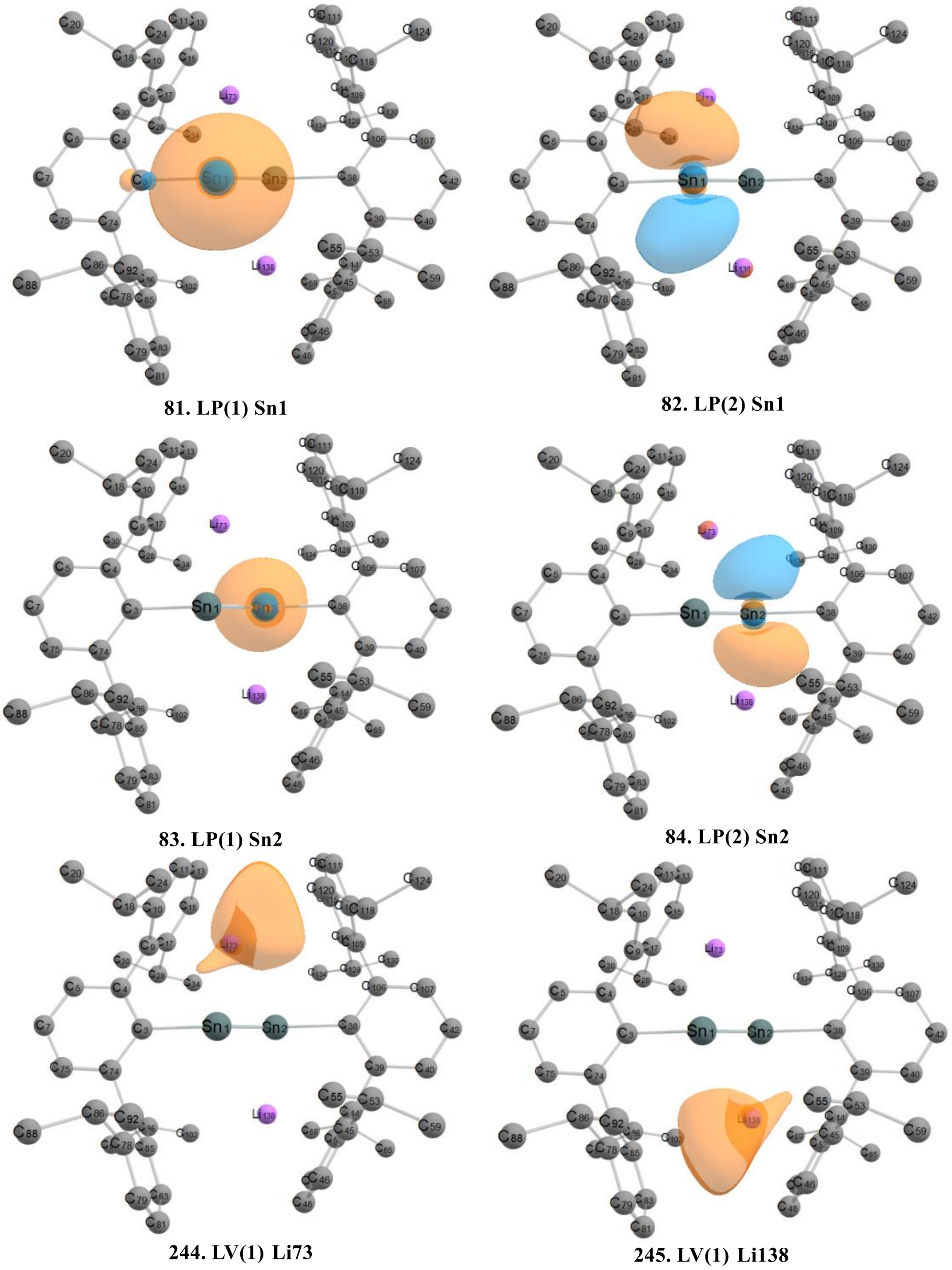

Figure S56. Selected alpha NBO orbitals for compound **$[LSnLi]_2^{\bullet-}$** radical anion, **$1^{Li\bullet-}$**.

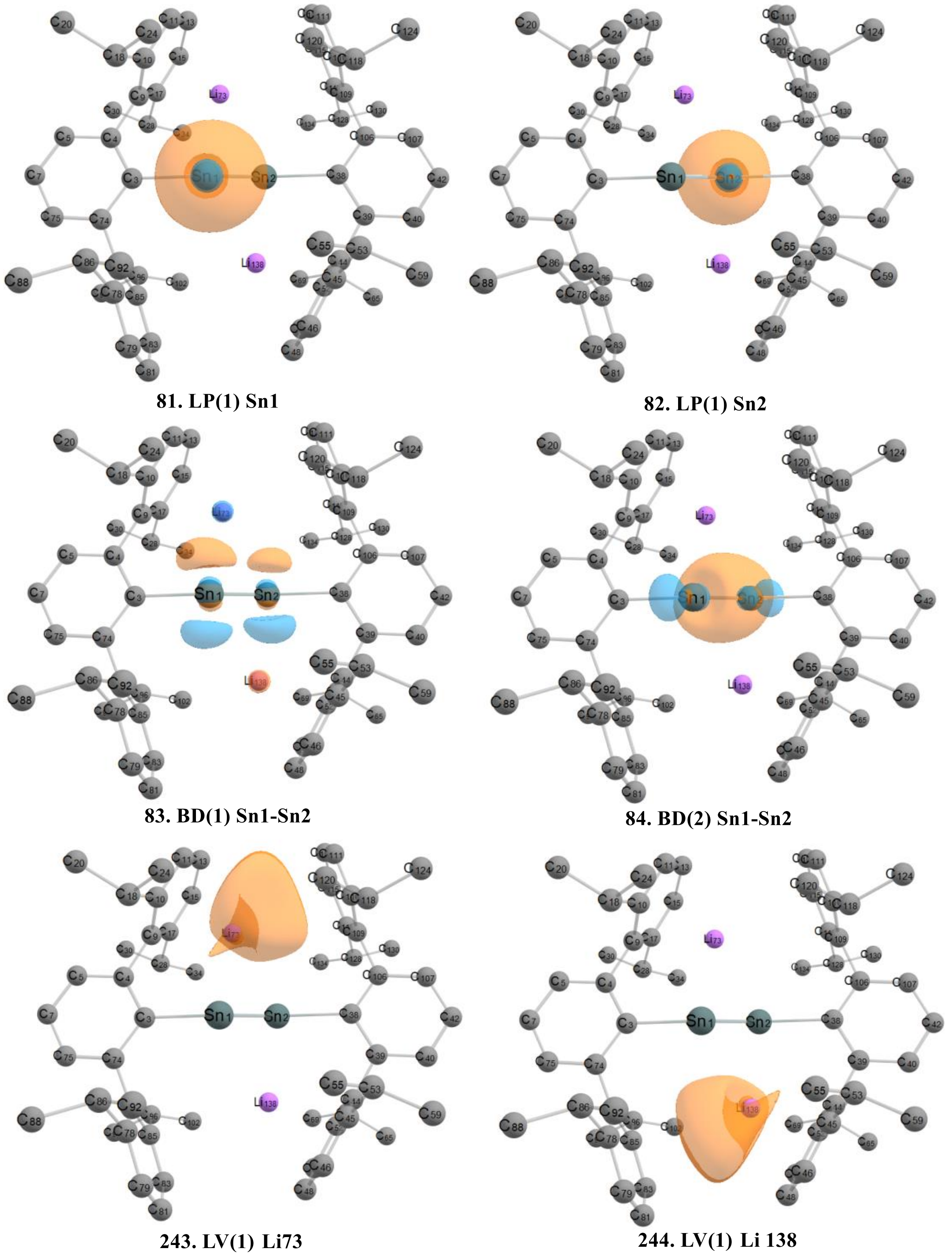

Figure S57. Selected beta NBO orbitals for compound $\mathbf{[LSnLi]_2^{\bullet-}}$ radical anion, $\mathbf{1^{Li\bullet-}}$.

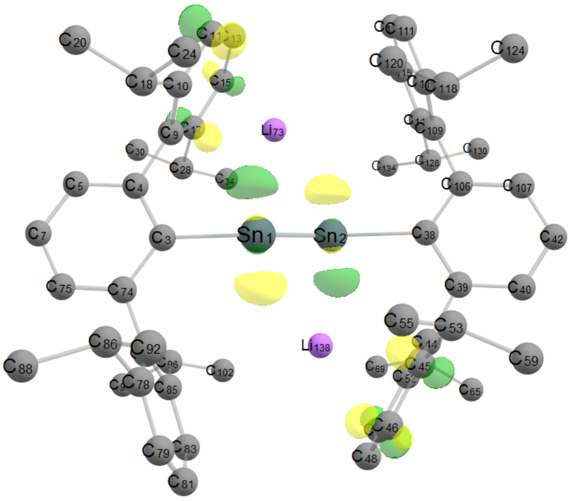

SOMO
0.31 eV

Figure S58. Alpha SOMO of **[LSnLi]$_2$$^{\bullet-}$** radical anion.

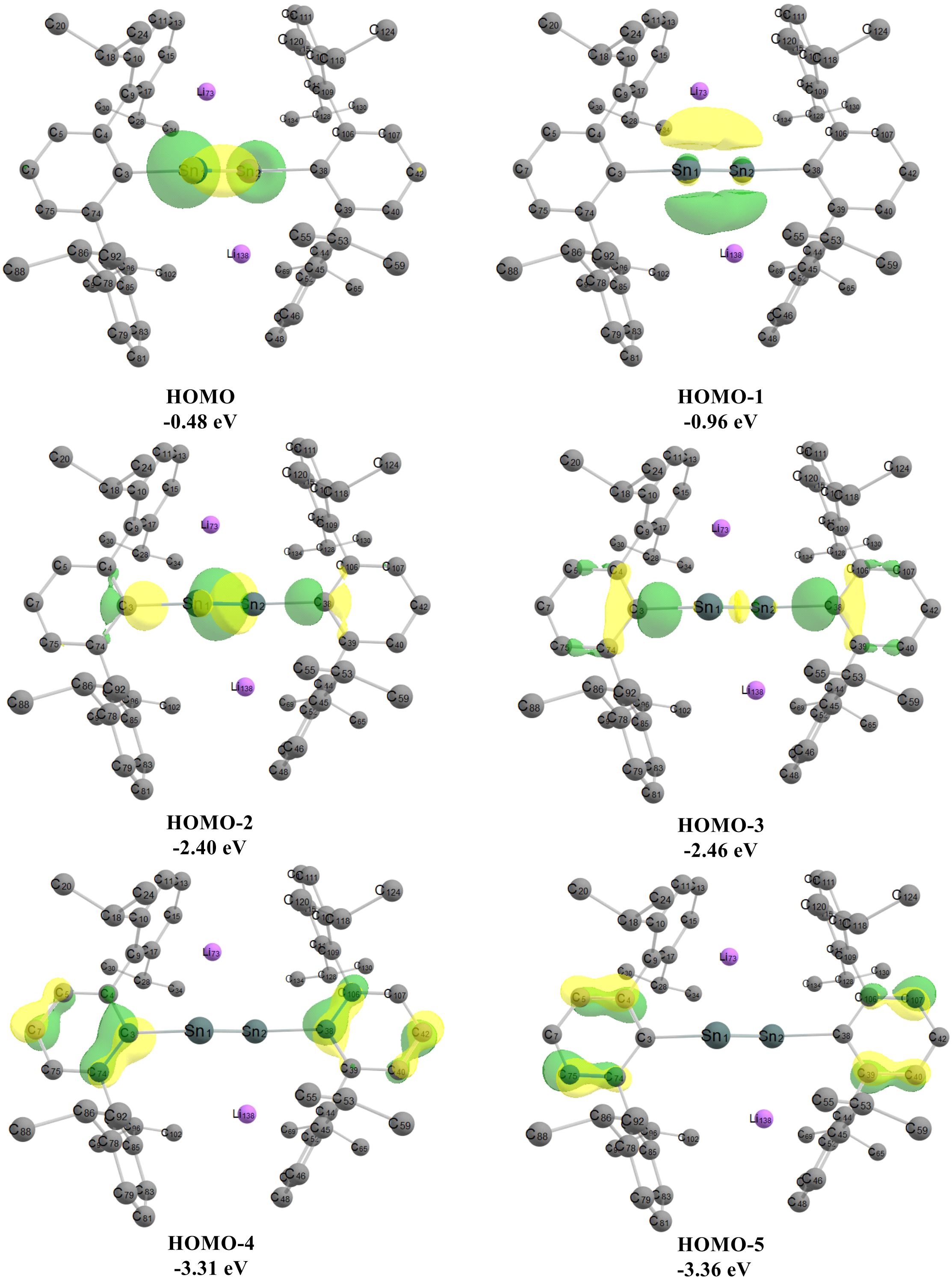

Figure S59. Alpha molecular orbitals HOMO-5 to HOMO for compound **[LSnLi]$_2$$^{•−}$** radical anion, **1$^{Li•−}$**.

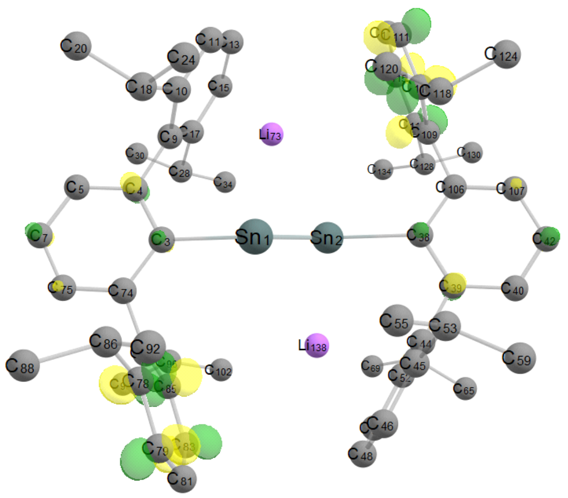

LUMO+4
1.05 eV

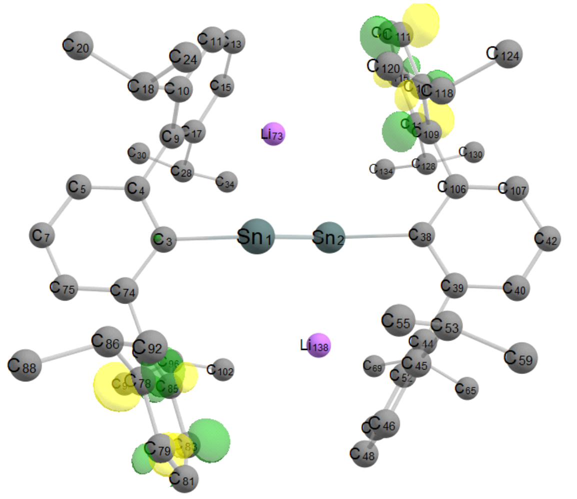

LUMO+5
1.12 eV

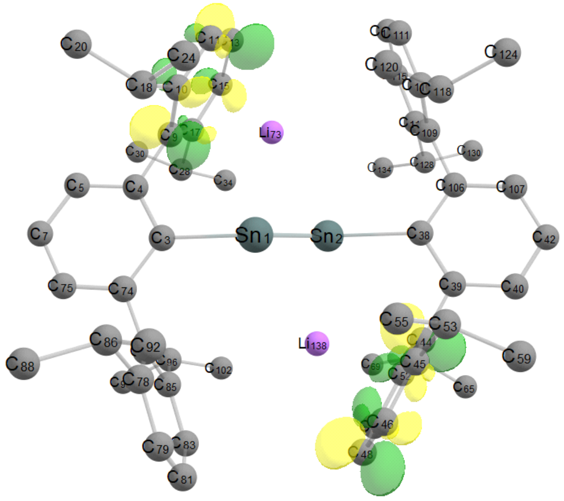

LUMO+2
0.78 eV

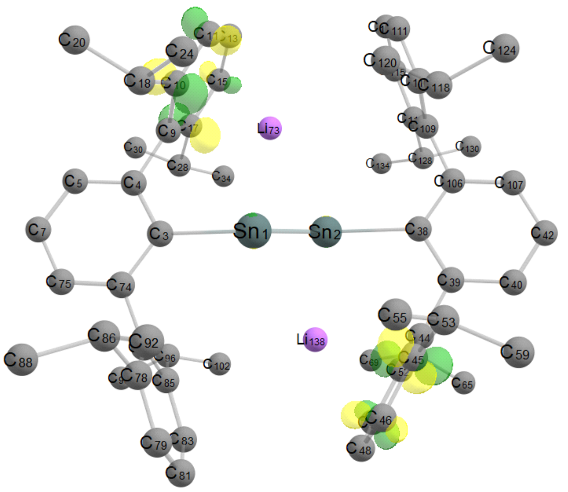

LUMO+3
0.87 eV

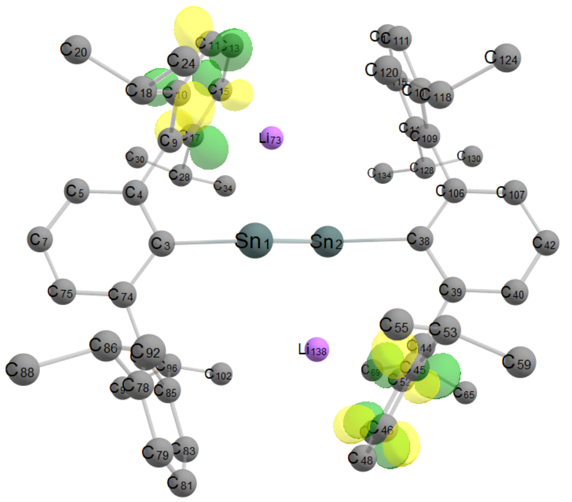

LUMO
0.66 eV

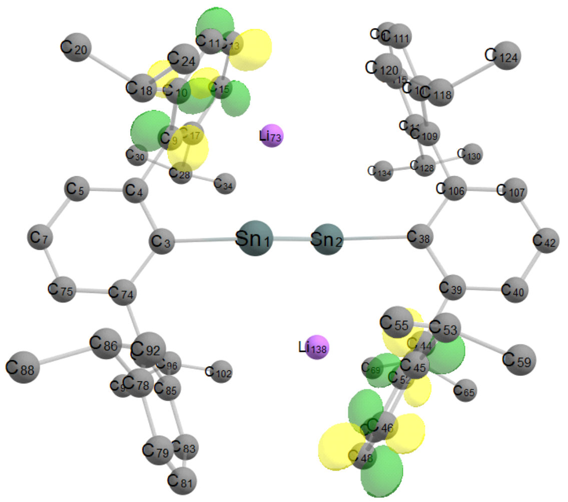

LUMO+1
0.75 eV

Figure S60. Alpha molecular orbitals LUMO to LUMO+5 for compound **[LSnLi]$_2^{\bullet-}$** radical anion, **1$^{Li\bullet-}$**.

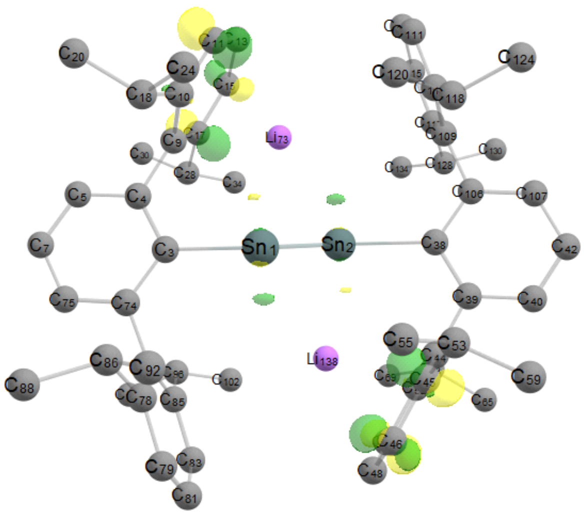

SOMO
0.58 eV

Figure S61. Beta SOMO of **[LSnLi]$_2^{\bullet-}$** radical anion, **$1^{Li\bullet-}$**.

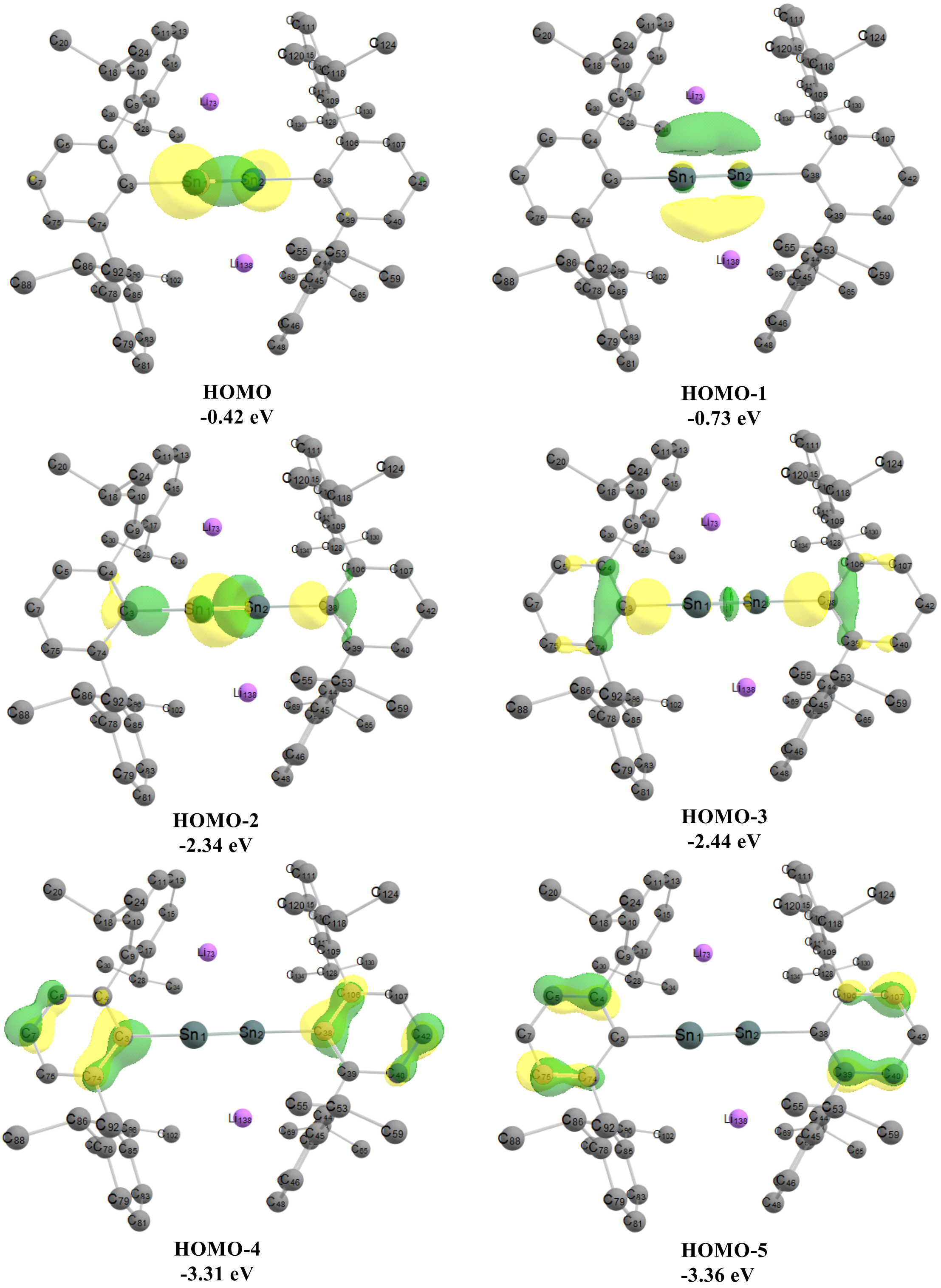

Figure S62. Beta molecular orbitals HOMO-5 to HOMO for compound $\mathbf{[LSnLi]_2^{\bullet-}}$ radical anion, $\mathbf{1^{Li\bullet-}}$.

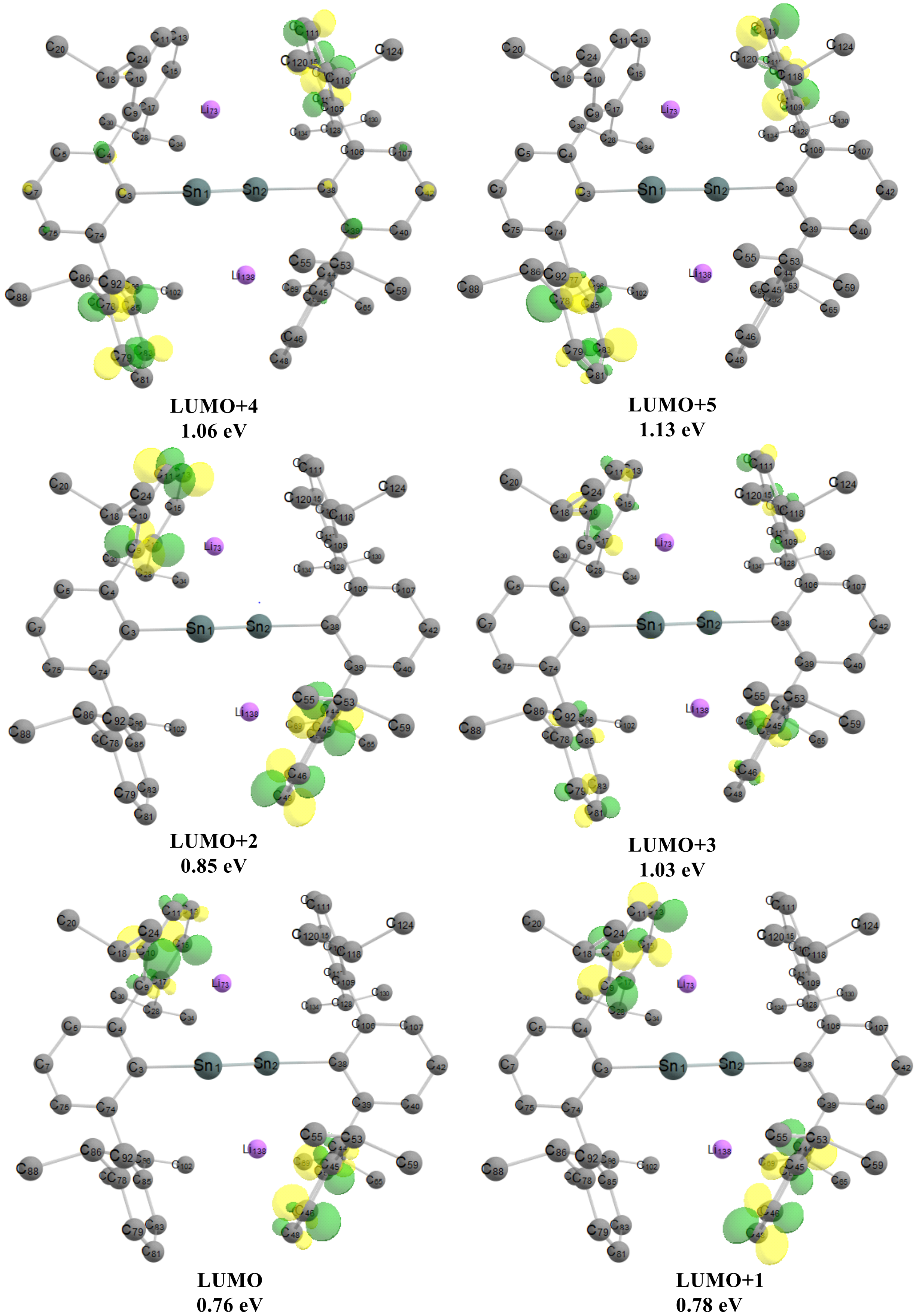

Figure S63. Beta molecular orbitals LUMO to LUMO+5 for compound **[LSnLi]$_2$$^{\bullet-}$** radical anion, **1$^{Li\bullet-}$**.

Table S27. Selected Mulliken spin density values for **[LSnLi]**$_2^{\bullet-}$ radical anion ($\mathbf{1}^{\mathbf{Li}\bullet-}$) with hydrogens summed into heavy atoms. (All other atoms have a Mulliken spin density value < |±0.05|).

**Atom**	**Mulliken Spin density**
Sn1	0.231
Sn2	0.232
C11	0.099
C17	0.094
C45	0.094
C50	0.099
Li73	0.053
Li138	0.053

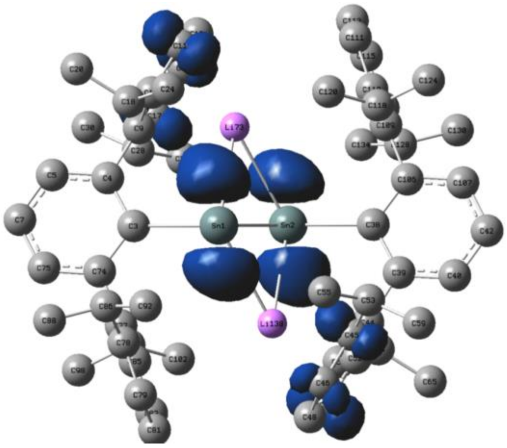

Figure S64. Spin density plot of compound $\mathbf{1}^{\mathbf{Li}\bullet-}$**.**

M = Na

Table S28. Selected donor - acceptor interaction energies, $E^{(2)}$, in kcal mol^{-1} for r **$[LSnNa]_2^{\bullet-}$** radical anion, **$1^{Na\bullet-}$**. (Alpha spin NBOs).

Donor Orbital	Acceptor Orbital	$E^{(2)}$ kcal/mol
89. LP(1) Sn1	252. LV(1) Na2	2.8
90. LP(2) Sn1	252. LV(1) Na2	2.1
91. LP(1) Sn70	252. LV(1) Na2	2.8
92. LP(2) Sn70	252. LV(1) Na2	2.1
94. BD(1) Sn1-Sn70	252. LV(1) Na2	0.4
89. LP(1) Sn1	253. LV(1) Na71	2.8
90. LP(2) Sn1	253. LV(1) Na71	2.1
91. LP(1) Sn70	253. LV(1) Na71	2.8
92. LP(2) Sn70	253. LV(1) Na71	2.1
94. BD(1) Sn1-Sn70	253. LV(1) Na71	0.4

Table S29. Selected donor - acceptor interaction energies, $E^{(2)}$, in kcal mol^{-1}, for **$[LSnNa]_2^{\bullet-}$** radical anion, **$1^{Na\bullet-}$**. (Beta spin NBOs).

Donor Orbital	Acceptor Orbital	$E^{(2)}$ kcal/mol
89. LP(1) Sn1	251. LV(1) Na2	2.7
90. LP(1) Sn70	251. LV(1) Na2	2.7
92. BD(1) Sn1-Sn70	251. LV(1) Na2	3.6
93. BD(2) Sn1-Sn70	251. LV(1) Na2	0.5
89. LP(1) Sn 1	252. LV(1) Na71	2.7
90. LP(1) Sn70	252. LV(1) Na71	2.7
92. BD(1) Sn1-Sn70	252. LV(1) Na71	3.6
93. BD(2) Sn1-Sn70	252. LV(1) Na71	0.5

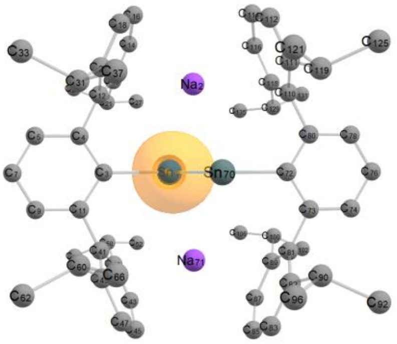

89. LP (1) Sn1

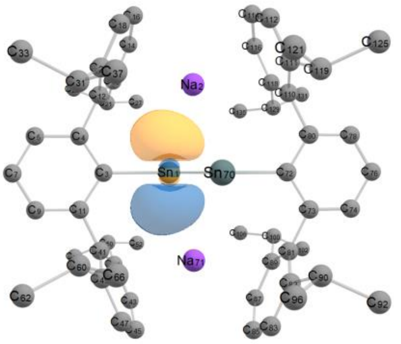

90. LP(2) Sn1

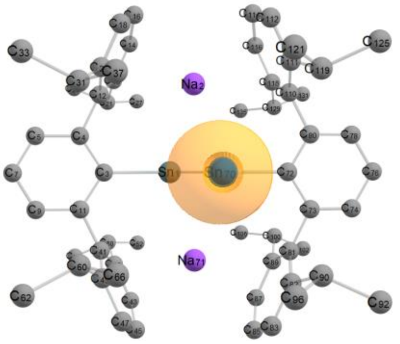

91. LP(1) Sn70

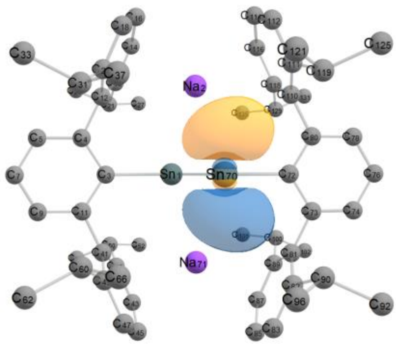

92. LP(2) Sn70

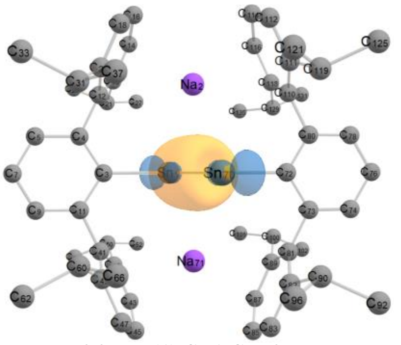

94. BD(1) Sn1-Sn70

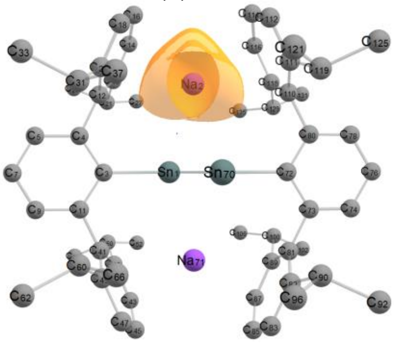

252. LV(1) Na2

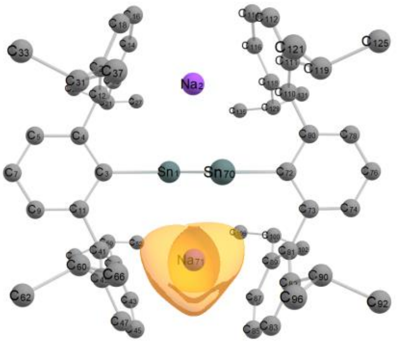

253. LV(1) Na71

Figure S65. Selected alpha NBO orbitals for compound **$[LSnNa]_2^{\bullet-}$** radical anion, **$1^{Na\bullet-}$**.

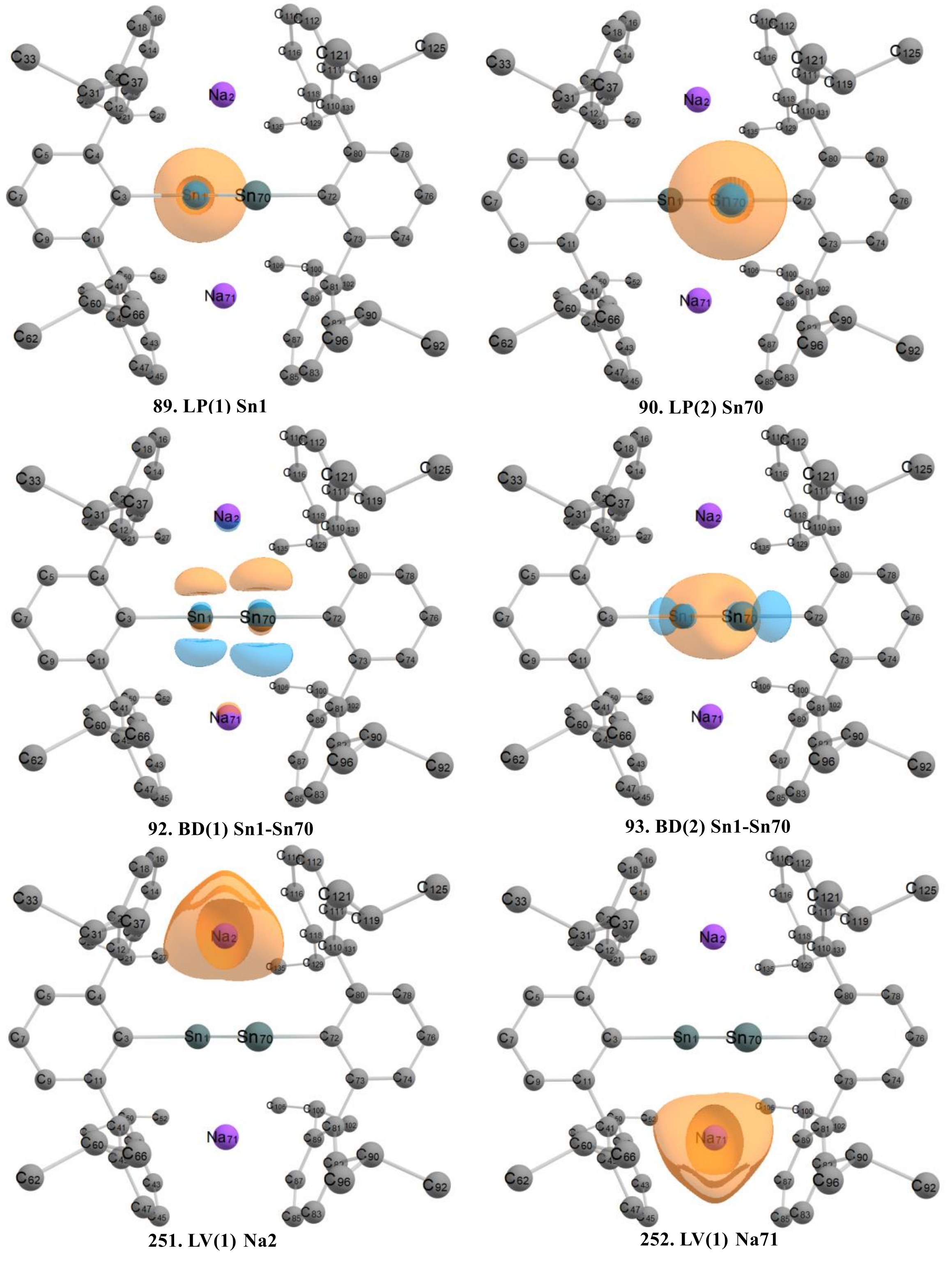

Figure S66. Selected beta NBO orbitals for compound **[LSnNa]$_2^{\bullet-}$** radical anion, **1**$^{\mathbf{Na\bullet-}}$.

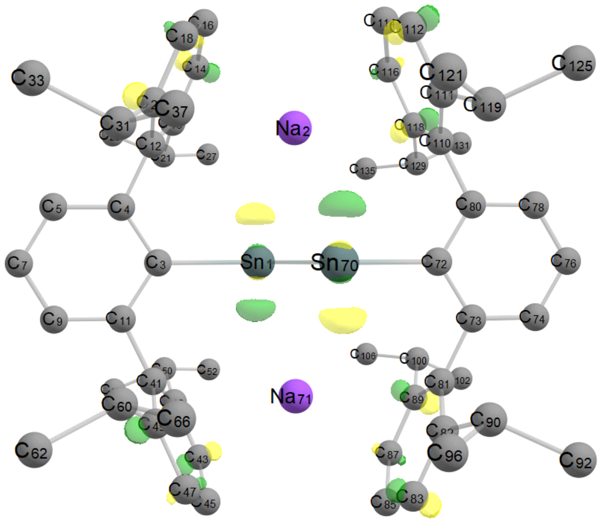

SOMO
0.48 eV

Figure S67. Alpha SOMO of **[LSnNa]$_2^{\bullet-}$** radical anion, **1$^{Na\bullet-}$**.

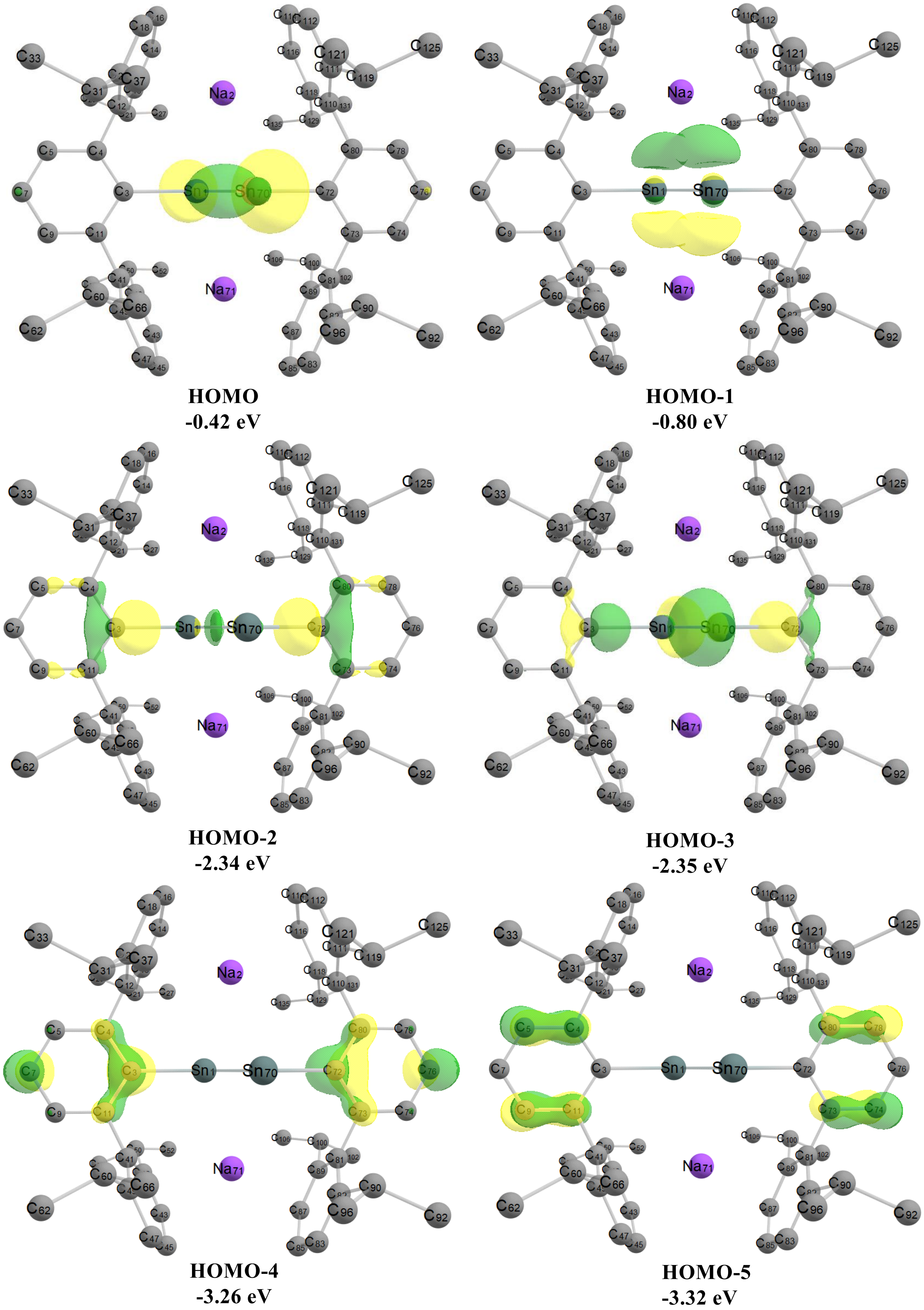

Figure S68. Alpha molecular orbitals HOMO-5 to HOMO for compound **$[LSnNa]_2^{\bullet-}$** radical anion, **$1^{Na\bullet-}$**.

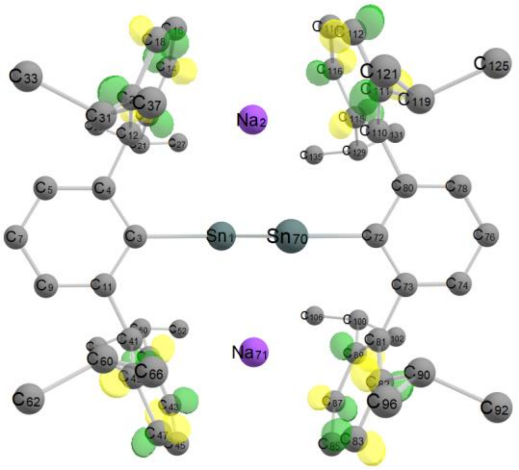

LUMO+4
0.96 eV

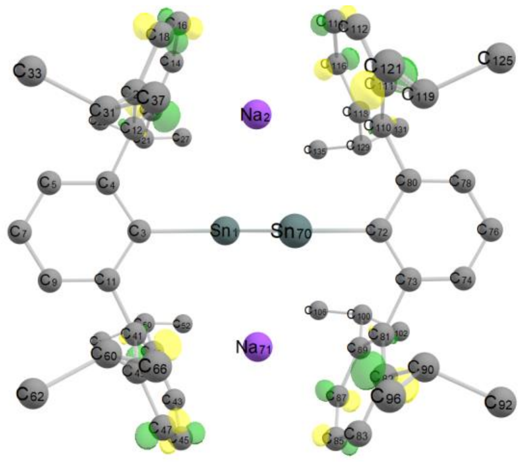

LUMO+5
1.00 eV

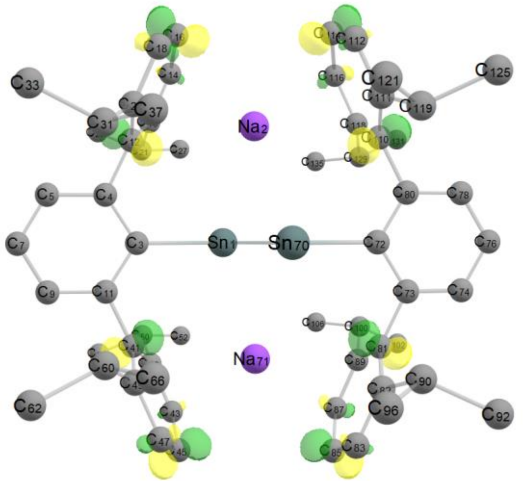

LUMO+2
0.91 eV

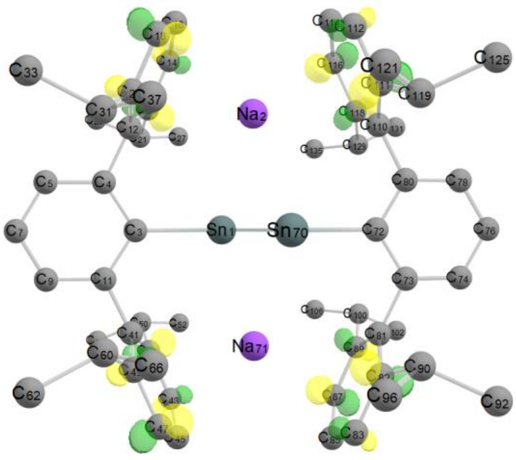

LUMO+3
0.94 eV

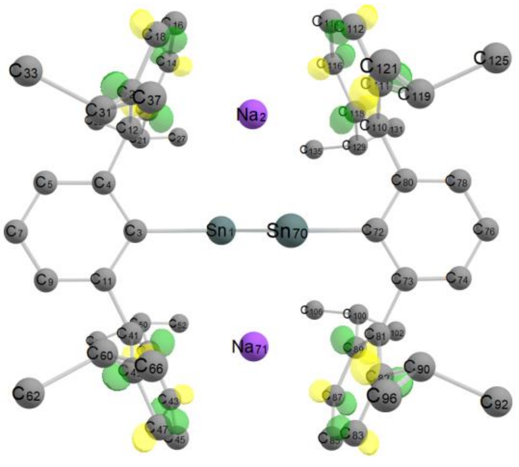

LUMO
0.72 eV

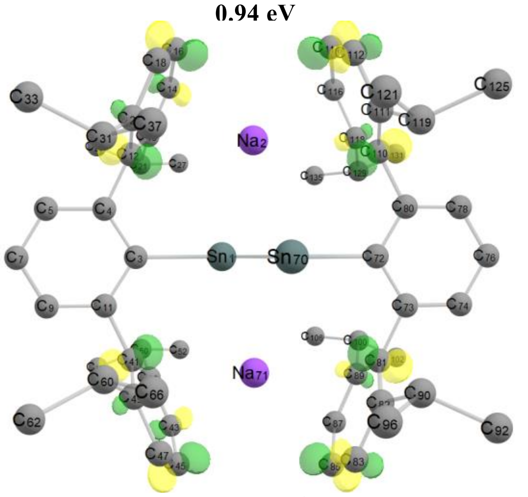

LUMO+1
0.80 eV

Figure S69. Alpha molecular orbitals LUMO to LUMO+5 for compound **[LSnNa]$_2^{\bullet-}$** radical anion, **$1^{Na\bullet-}$**.

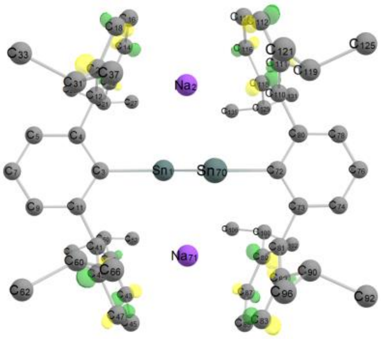

SOMO
0.68 eV

Figure S70. Beta SOMO of **$[LSnNa]_2^{\bullet-}$** radical anion, **$1^{Na\bullet-}$**.

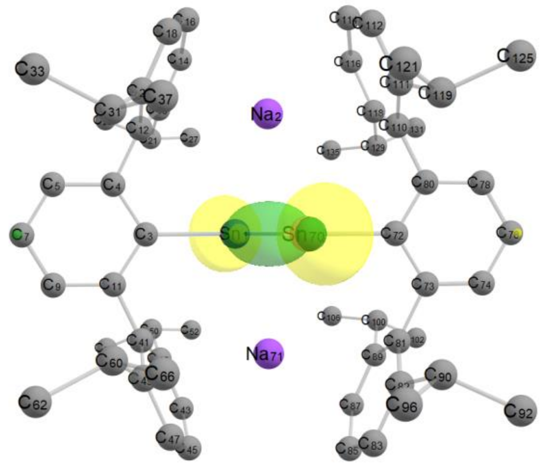

HOMO
-0.36 eV

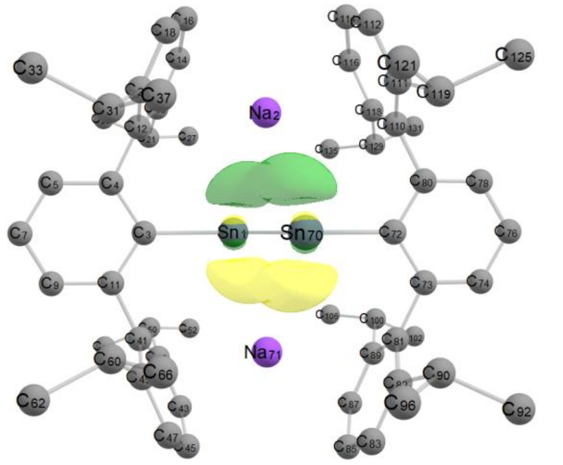

HOMO-1
-0.59 eV

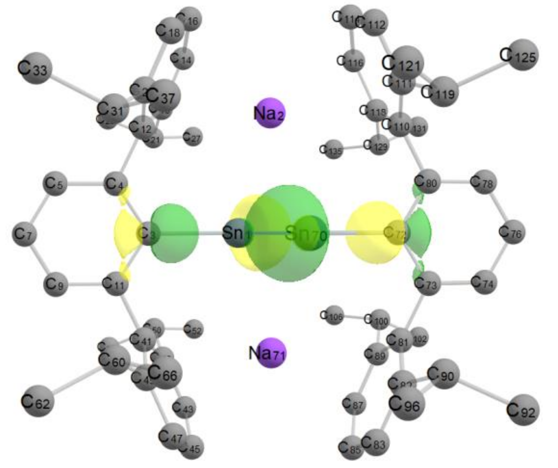

HOMO-2
-2.30 eV

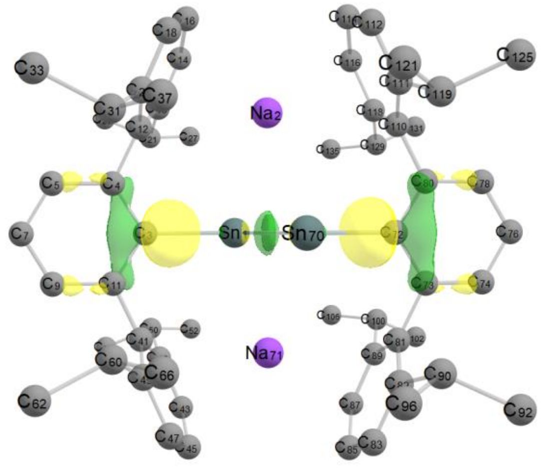

HOMO-3
-2.33 eV

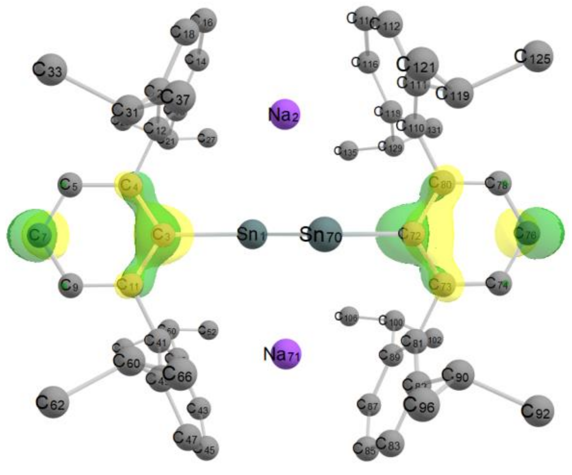

HOMO-4
-3.25 eV

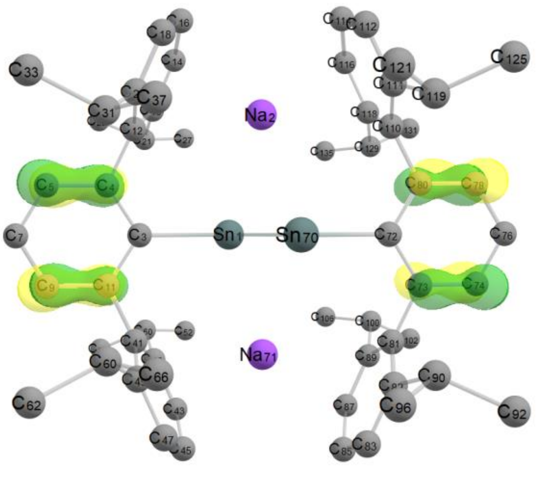

HOMO-5
-3.31 eV

Figure S71. Beta molecular orbitals HOMO-5 to HOMO for compound **[LSnNa]$_2$$^{\bullet-}$** radical anion, **1$^{Na\bullet-}$**.

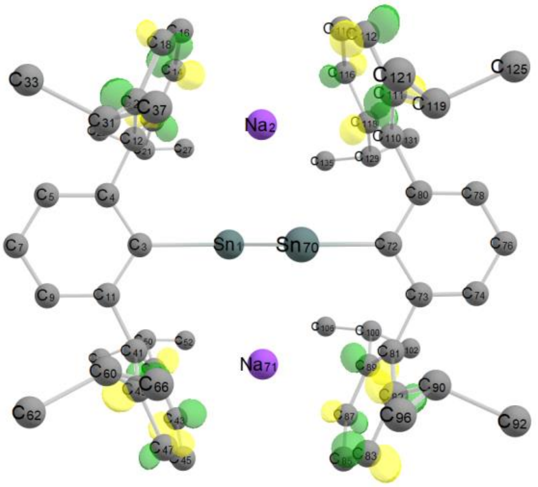

LUMO+4
1.05 eV

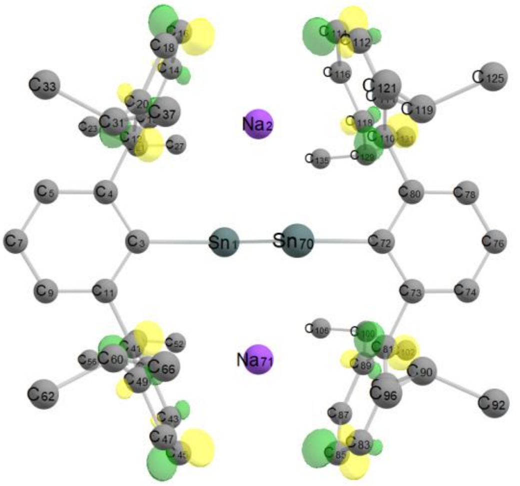

LUMO+5
1.06 eV

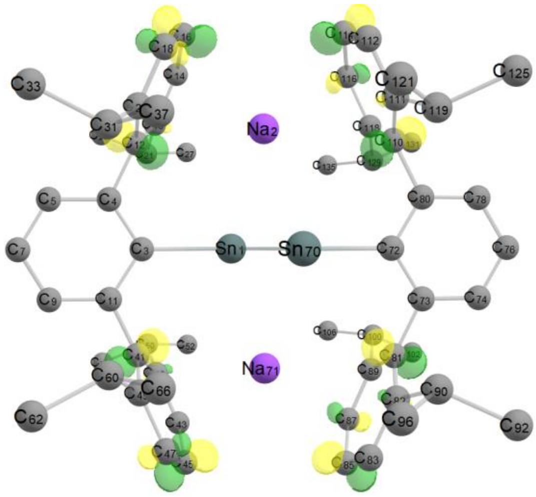

LUMO+2
0.93 eV

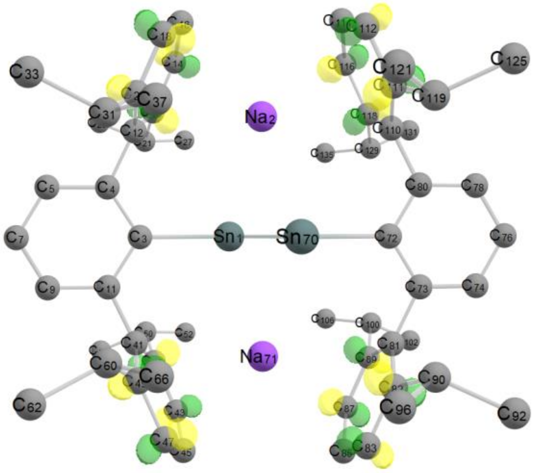

LUMO+3
1.02 eV

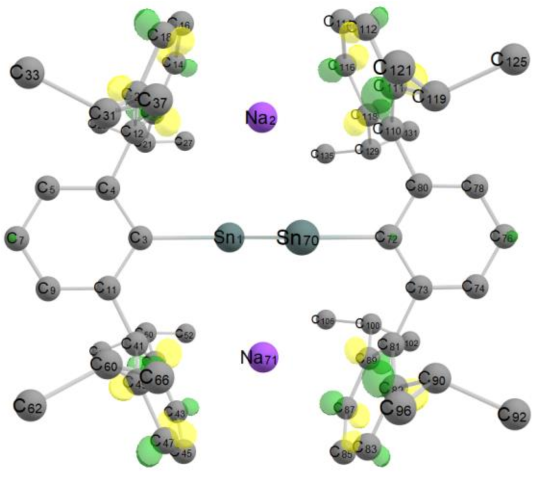

LUMO
0.80 eV

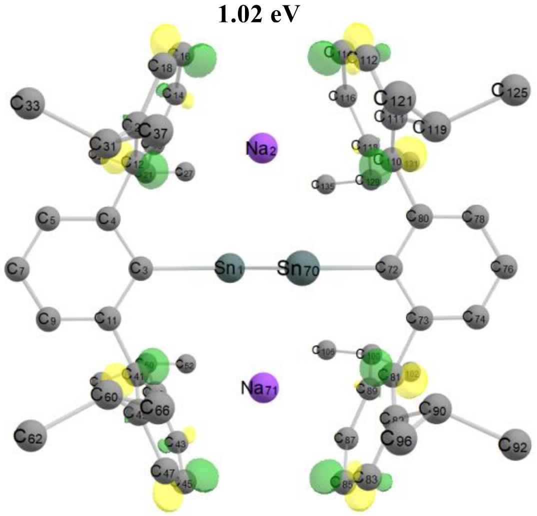

LUMO+1
0.82 eV

Figure S72. Beta molecular orbitals LUMO to LUMO+5 for compound **$[LSnNa]_2^{\bullet-}$** radical anion, **$1^{Na\bullet-}$**.

Table S30. Selected Mulliken spin density values for $\mathbf{[LSnNa]_2^{\bullet-}}$ radical anion ($\mathbf{1}^{\mathbf{Na}\bullet-}$) with hydrogens summed into heavy atoms. (All other atoms have a Mulliken spin density value < |±0.05|).

Atom	Mulliken Spin Density
Sn1	0.304
Na2	-0.063
C14	0.049
C20	0.049
C43	0.049
C49	0.049
Sn70	0.304
Na71	-0.063
C83	0.049
C89	0.049
C112	0.049
C118	0.049

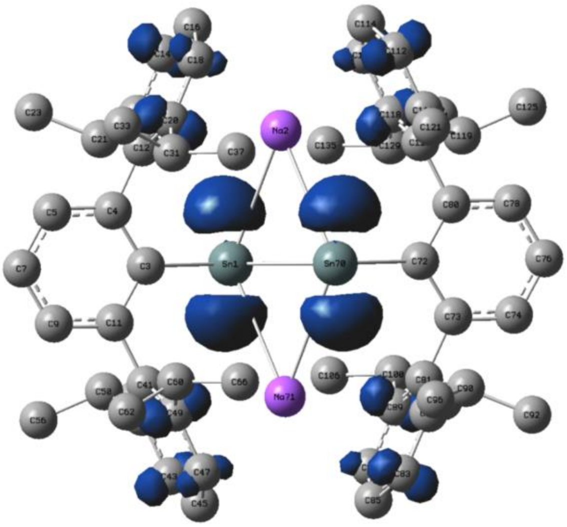

Figure S73. Spin density plot of compound $\mathbf{1}^{\mathbf{Na}\bullet-}$.

M = K

Table S31. Selected donor - acceptor interaction energies, $E^{(2)}$, in kcal mol^{-1} for **$[LSnK]_2^{\bullet-}$** radical anion, **$1^{K\bullet-}$**. (Alpha Spin NBO).

**Donor Orbital**	**Acceptor Orbital**	**$E^{(2)}$ kcal/mol**
97. LP(1) Sn1	260. LV(1) K2	2.0
98. LP(2) Sn1	260. LV(1) K2	2.7
99. LP(1) Sn70	260. LV(1) K2	2.0
100. LP(2) Sn70	260. LV(1) K2	2.6
102. BD(1) Sn1-Sn70	260. LV(1) K2	0.4
97. LP(1) Sn1	261. LV(1) K71	2.0
98. LP(2) Sn1	261. LV(1) K71	2.6
99. LP(1) Sn70	261. LV(1) K71	2.0
100. LP(2) Sn70	261. LV(1) K71	2.7
102. BD(1) Sn1-Sn70	261. LV(1) K71	0.4

Table S32. Selected donor - acceptor interaction energies, $E^{(2)}$, in kcal mol^{-1} for **$[LSnK]_2^{\bullet-}$** radical anion, **$1^{K\bullet-}$**. (Beta Spin NBO).

**Donor Orbital**	**Acceptor Orbital**	**$E^{(2)}$ kcal/mol**
97. LP(1) Sn1	259. LV(1) K2	2.0
98. LP(1) Sn70	259. LV(1) K2	1.9
100. BD(1) Sn1-Sn70	259. LV(1) K2	0.4
101. BD(2) Sn1-Sn70	259. LV(1) K2	4.4
97. LP(1) Sn1	260. LV(1) K71	1.9
98. LP(1) Sn70	260. LV(1) K71	2.0
100. BD(1) Sn1-Sn70	260. LV(1) K71	0.4
101. BD(2) Sn1-Sn70	260. LV(1) K71	4.4

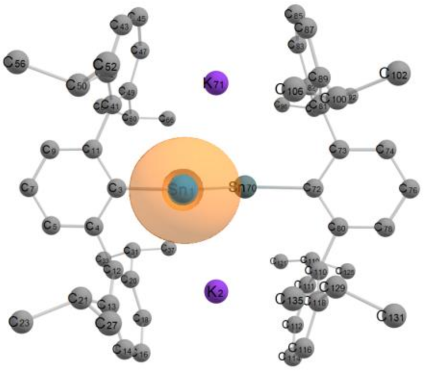

97. LP(1) Sn1

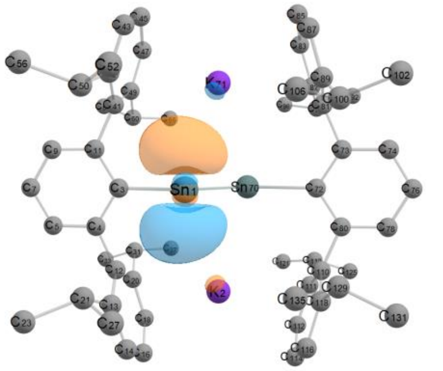

98. LP(2) Sn1

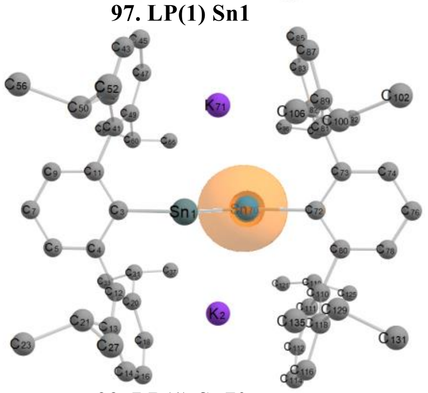

99. LP(1) Sn70

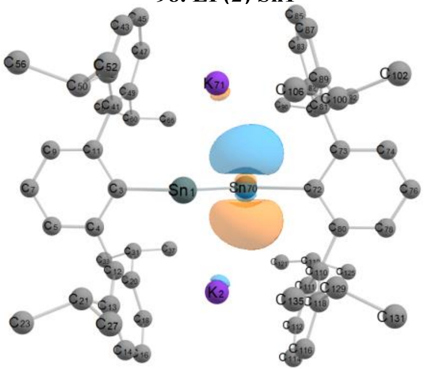

100. LP(2) Sn70

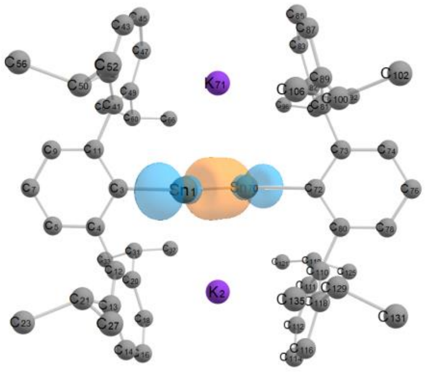

102. BD(1) Sn1-Sn70

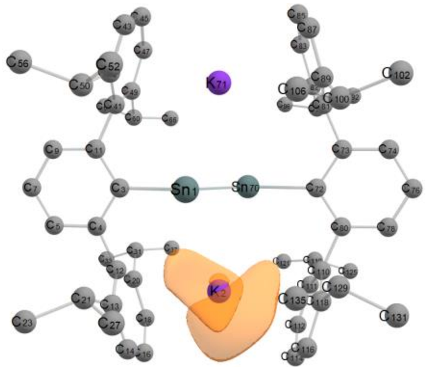

260. LV(1) K2

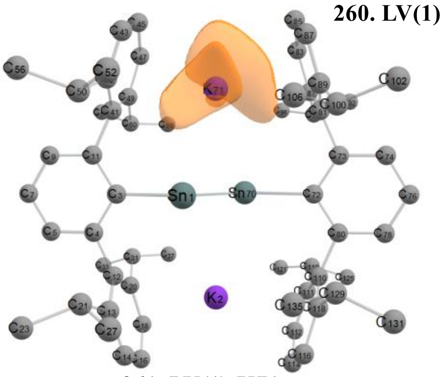

261. LV(1) K71

Figure S74. Selected alpha NBO orbitals for compound **$[LSnK]_2^{\bullet-}$** radical anion, **$1^{K\bullet-}$**.

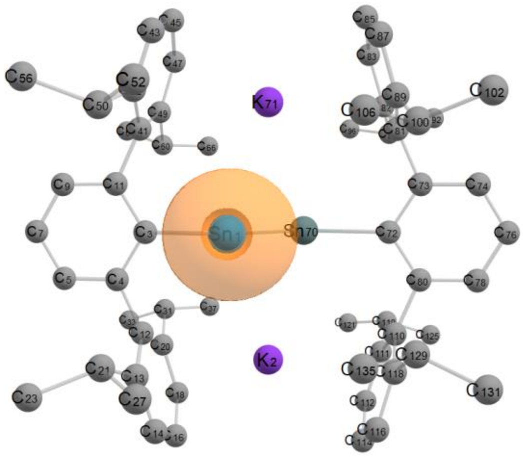

97. LP(1) Sn1

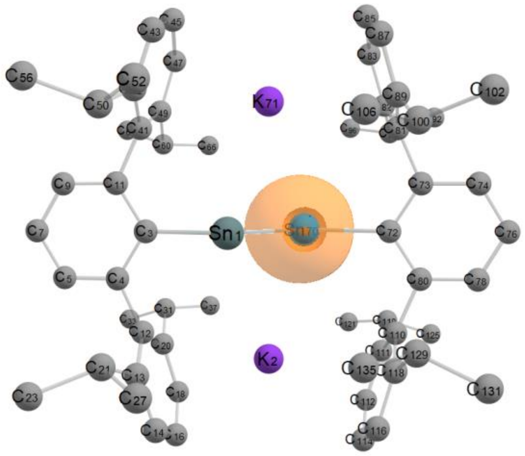

98. LP(2) Sn1

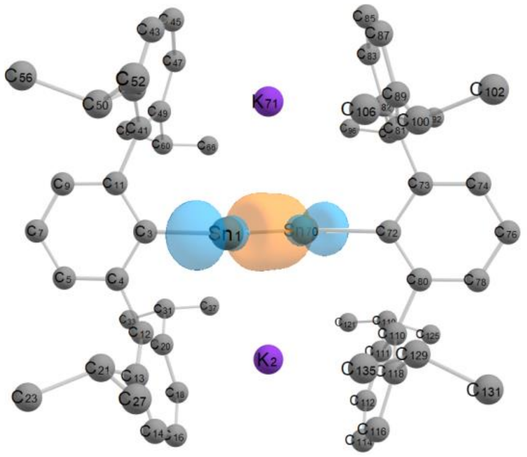

100. BD(1) Sn1-Sn70

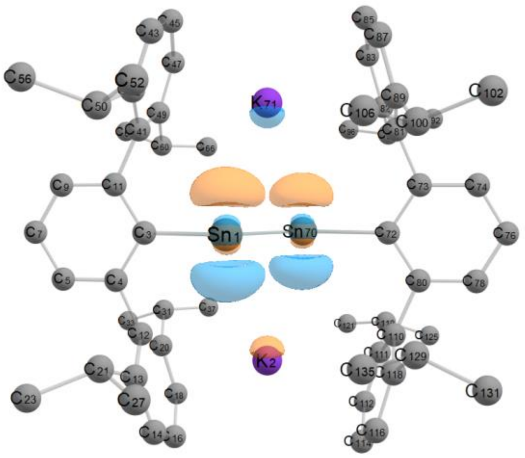

101. BD(2) Sn1-Sn70

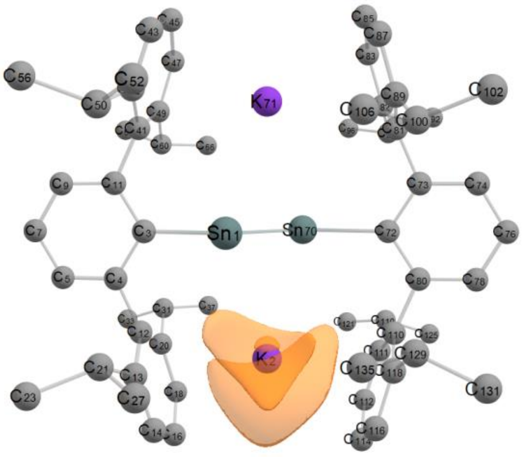

259. LV(1) K2

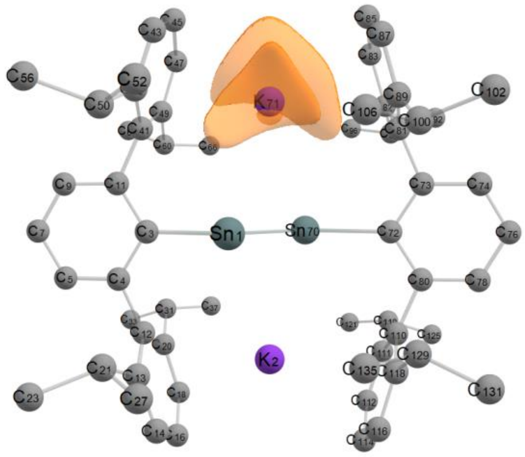

260. LV(1) K71

Figure S75. Selected beta NBO orbitals for compound **[LSnK]**$_2^{\bullet-}$ radical anion, **1**$^{\mathbf{K\bullet-}}$**.**

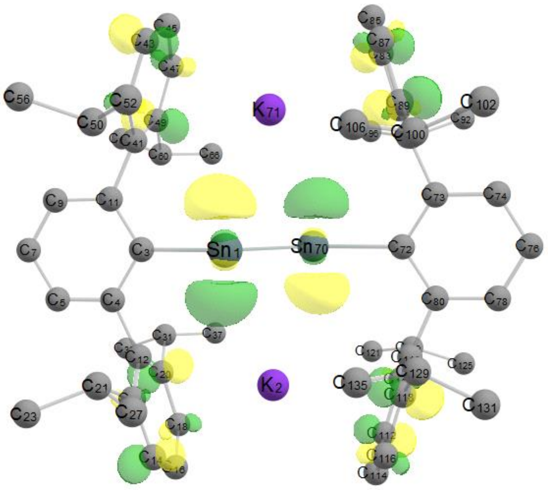

SOMO
0.48 eV

Figure S76. Alpha SOMO of **$[LSnK]_2^{\bullet-}$** radical anion, **$1^{K\bullet-}$**.

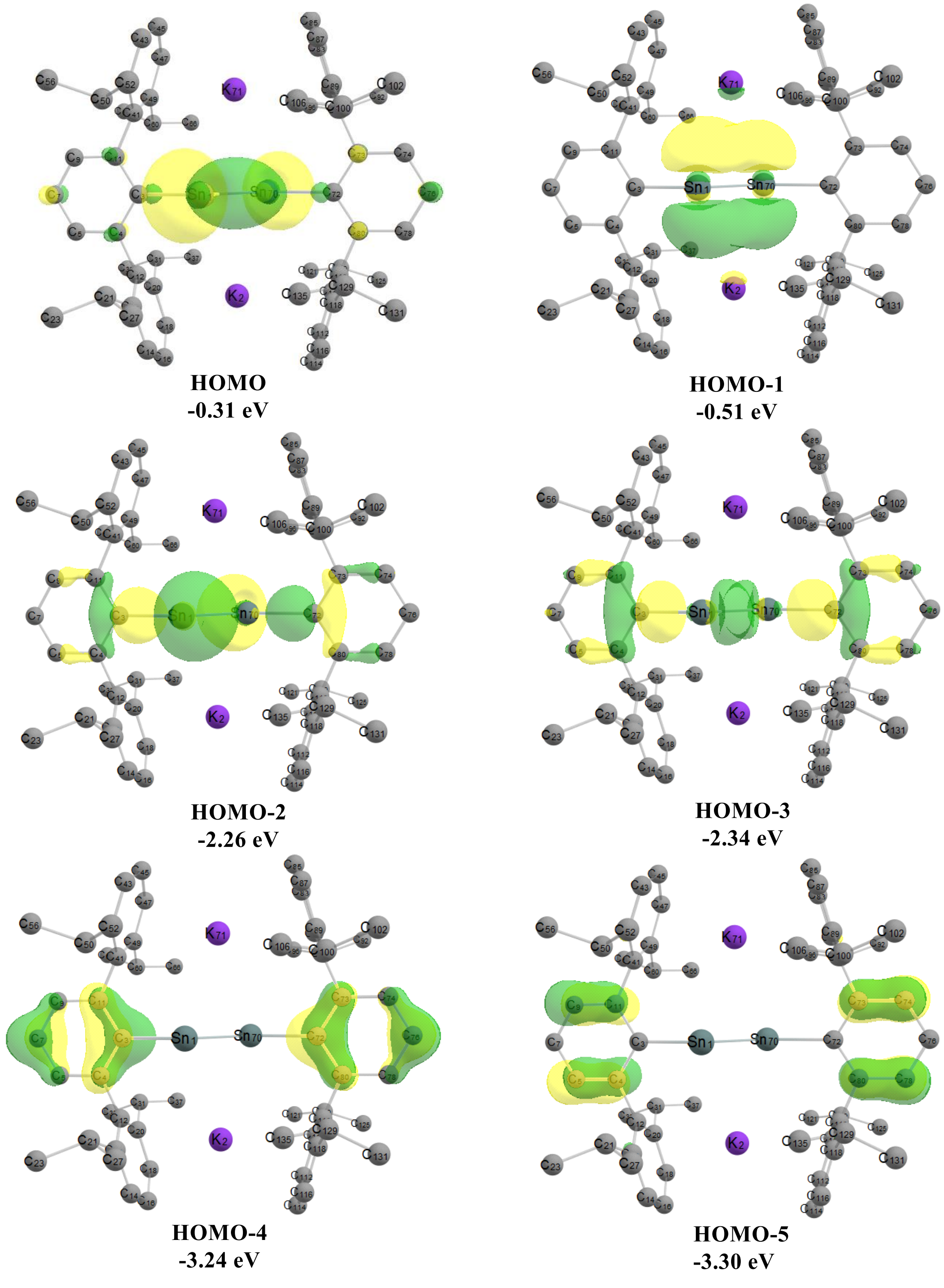

Figure S77. Alpha molecular orbitals HOMO-5 to HOMO for compound **$[LSnK]_2^{\bullet-}$** radical anion, **$1^{K\bullet-}$**.

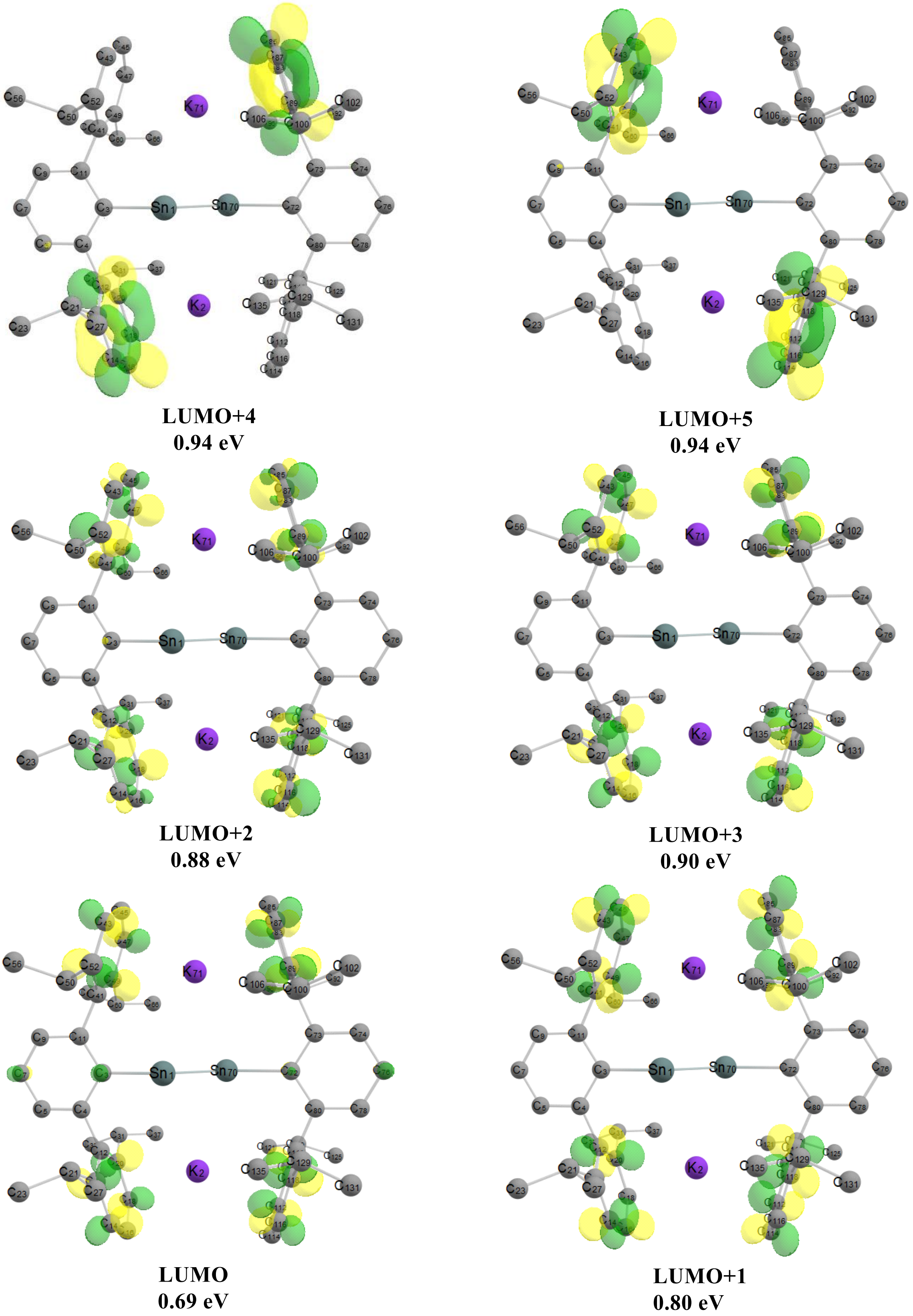

Figure S78. Alpha molecular orbitals LUMO to LUMO+5 for compound **$[LSnK]_2^{\bullet-}$** radical anion, **$1^{K\bullet-}$**.

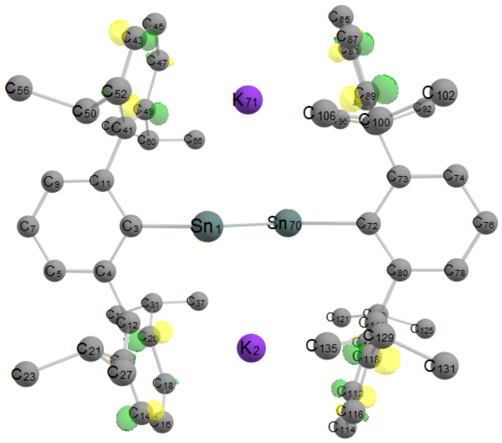

SOMO
0.67 eV

Figure S79. Beta SOMO of **[LSnK]$_2$$^{\bullet-}$** radical anion, **1$^{K\bullet-}$**.

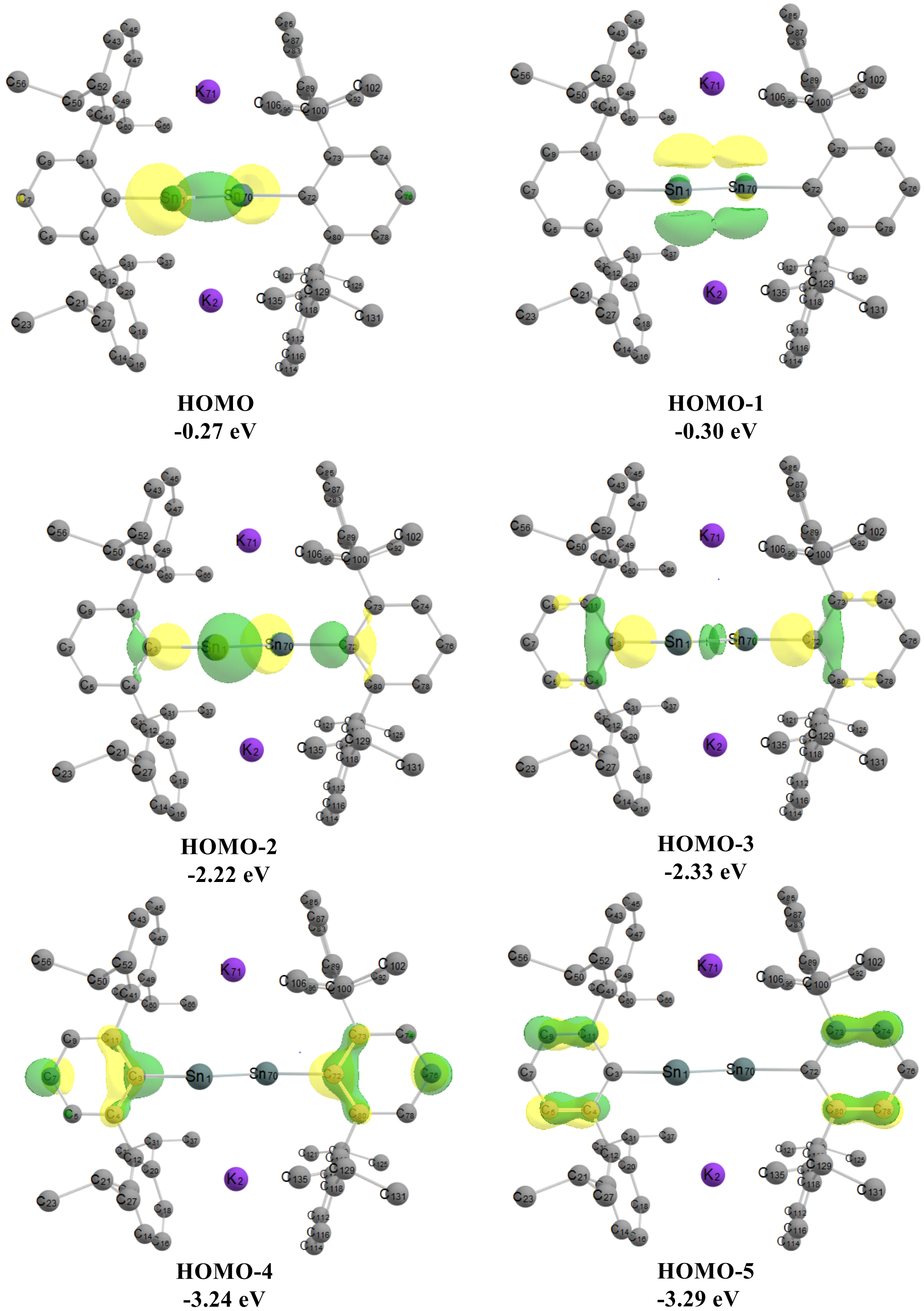

Figure S80. Beta molecular orbitals HOMO-5 to HOMO for compound **$[LSnK]_2^{\bullet-}$** radical anion, **$1^{K\bullet-}$**.

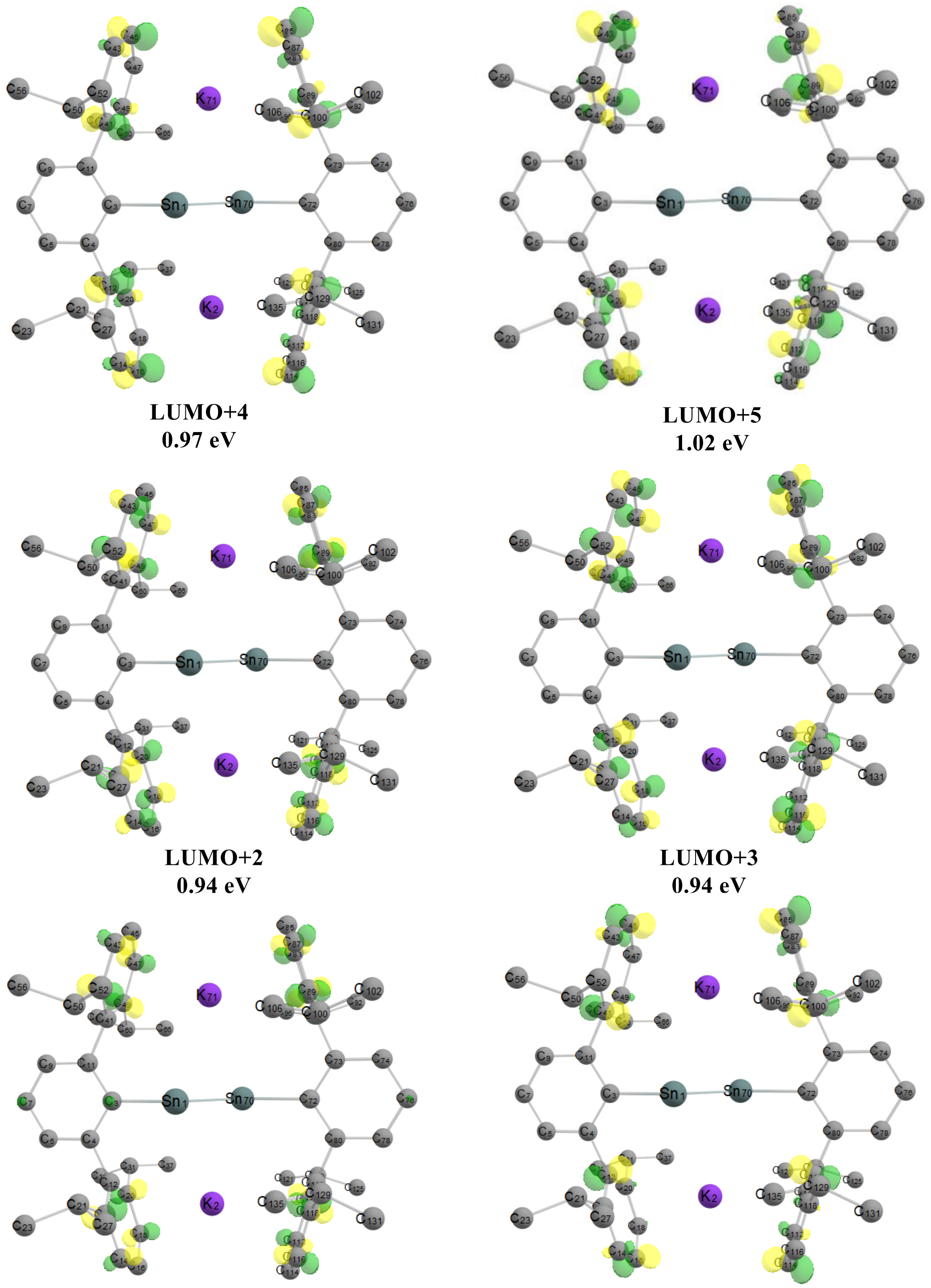

LUMO+4
0.97 eV

LUMO+5
1.02 eV

LUMO+2
0.94 eV

LUMO+3
0.94 eV

LUMO
0.77 eV

LUMO+1
0.84 eV

Figure S81. Beta molecular orbitals LUMO to LUMO+5 for compound **[LSnK]$_2$$^{\bullet-}$** radical anion, **1$^{K\bullet-}$**.

Table S33. Selected Mulliken spin density values for **$[LSnK]_2^{\bullet-}$** radical anion (**$1^{K\bullet-}$**) with hydrogens summed into heavy atoms. (All other atoms have a Mulliken spin density value < |±0.03|).

Atoms	Mulliken Spin Density
Sn1	0.229
K2	-0.014
C13	0.064
C18	0.026
C42	0.064
C47	0.055
Sn70	0.229
K71	-0.014
C87	0.054
C116	0.055

(*) K atoms show no detectable spin density within the set threshold.

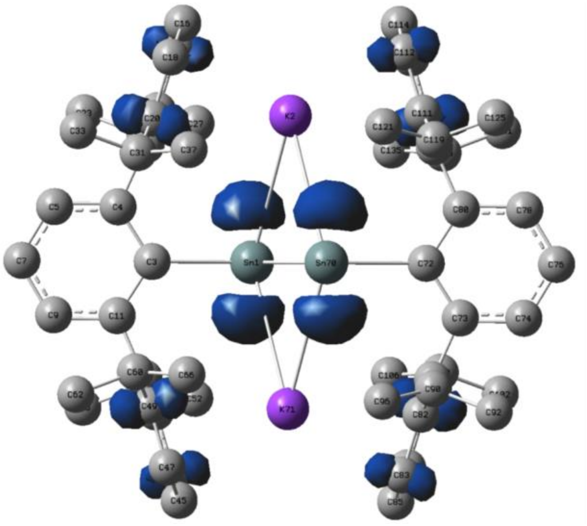

Figure S82. Spin density plot of compound **$1^{K\bullet-}$**.

M = Rb

Table S34. Selected donor - acceptor interaction energies, $E^{(2)}$, in kcal mol^{-1} for **[LSnRb]$_2^{\bullet-}$** radical anion, **1$^{Rb\bullet-}$**. (Alpha Spin NBO).

**Donor Orbital**	**Acceptor Orbital**	**E$^{(2)}$ kcal/mol**
87. LP(1) Sn1	250. LV(1) Rb2	1.7
88. LP(2) Sn1	250. LV(1) Rb2	7.4
89. LP(1) Sn70	250. LV(1) Rb2	1.5
90. LP(2) Sn70	250. LV(1) Rb2	7.1
92. BD(1) Sn1-Sn70	250. LV(1) Rb2	0.8
87. LP(1) Sn1	251. LV(1) Rb71	1.5
88. LP(2) Sn1	251. LV(1) Rb71	7.1
89. LP(1) Sn70	251. LV(1) Rb71	1.7
90. LP(2) Sn70	251. LV(1) Rb71	7.4
92. BD(1) Sn1-Sn70	251. LV(1) Rb71	0.8

Table S35. Selected donor - acceptor interaction energies, $E^{(2)}$, in kcal mol^{-1} for **[LSnRb]$_2^{\bullet-}$** radical anion, **1$^{Rb\bullet-}$**. (Beta Spin NBO).

**Donor Orbital**	**Acceptor Orbital**	**E$^{(2)}$ kcal/mol**
87. LP(1) Sn1	249. LV(1) Rb2	1.6
88. LP(1) Sn70	249. LV(1) Rb2	1.5
90. BD(1) Sn1-Sn70	249. LV(1) Rb2	0.9
91. BD(2) Sn1-Sn70	249. LV(1) Rb2	14.1
87. LP (1) Sn1	250. LV(1) Rb71	1.5
88. LP (1) Sn70	250. LV(1) Rb71	1.6
90. BD(1) Sn1-Sn70	250. LV(1) Rb71	0.9
91. BD(2) Sn1-Sn70	250. LV(1) Rb71	14.1

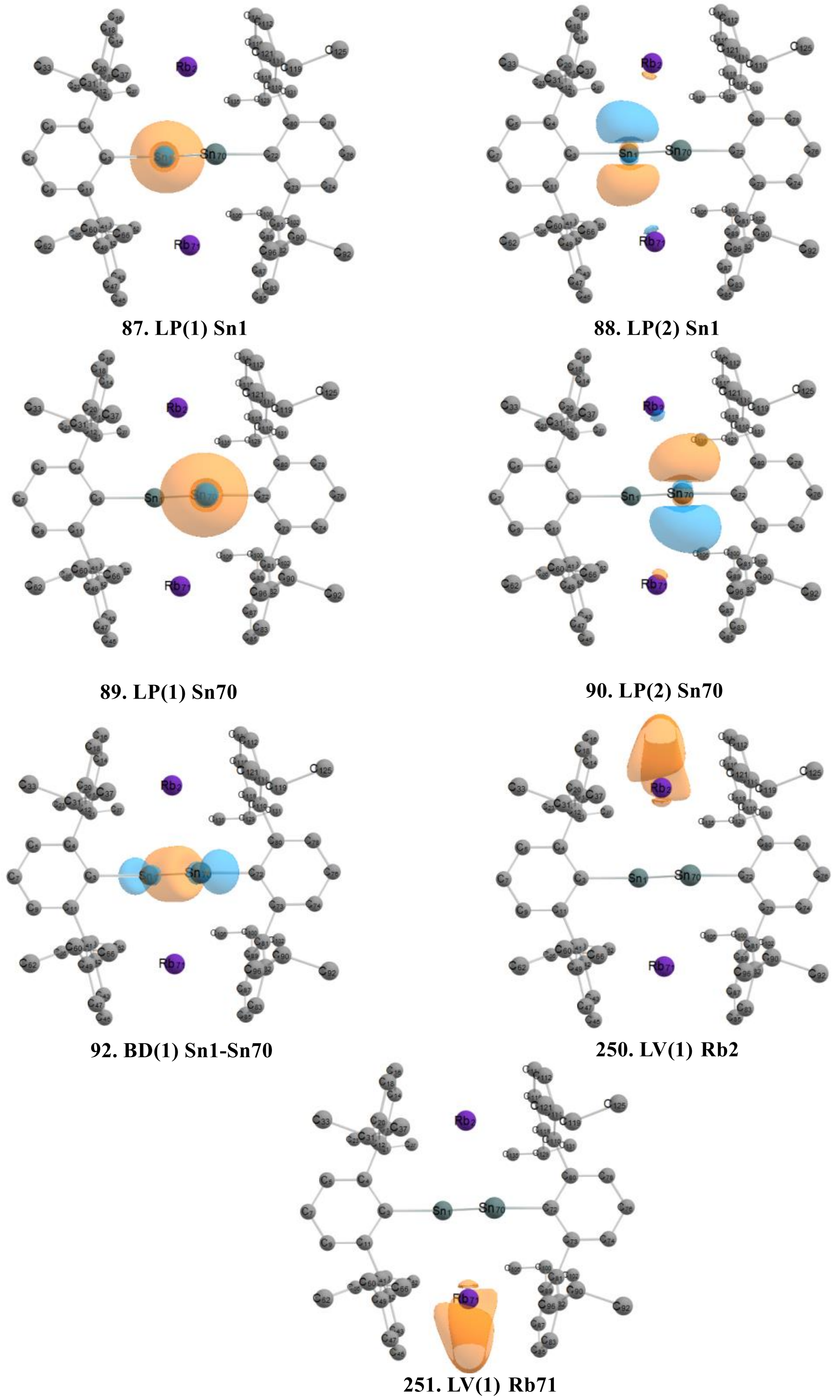

Figure S83. Selected alpha NBO orbitals for compound **[LSnRb]$_2$$^{\bullet-}$** radical anion, **1$^{Rb\bullet-}$**.

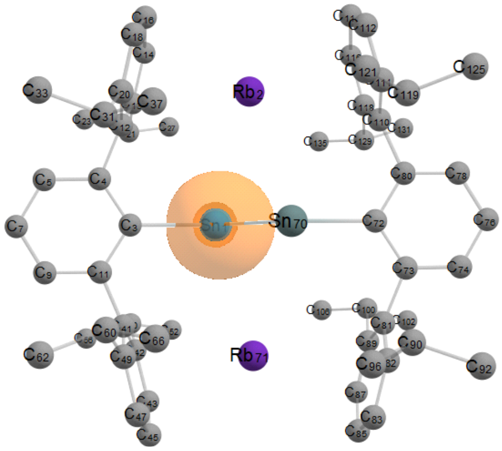

87. LP(1) Sn1

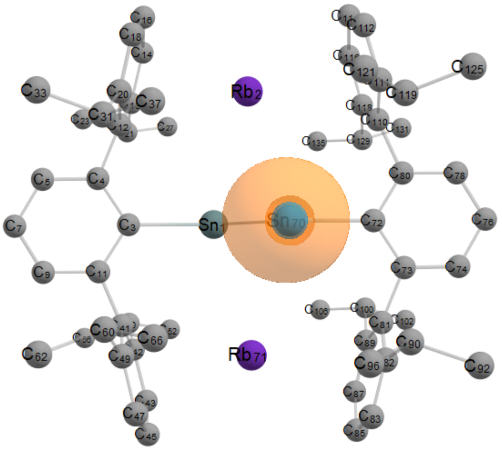

88. LP(2) Sn1

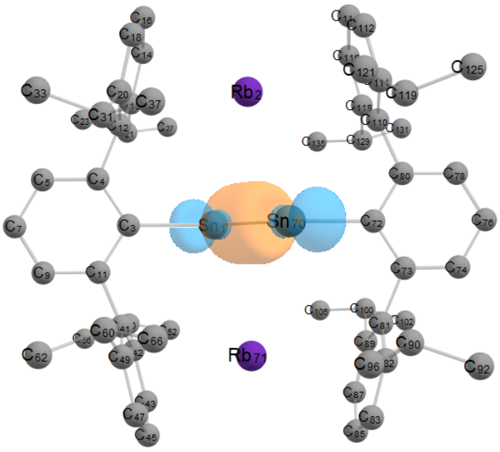

90. BD(1) Sn1-Sn70

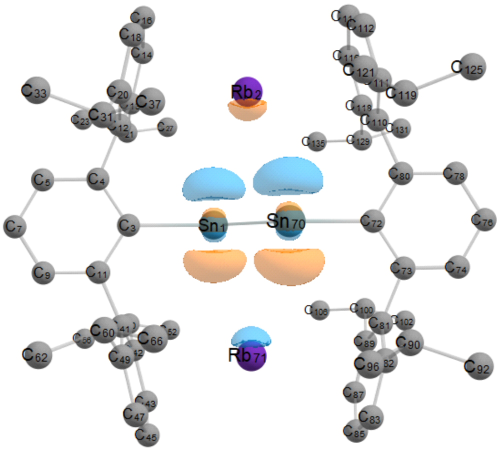

91. BD(2) Sn1-Sn70

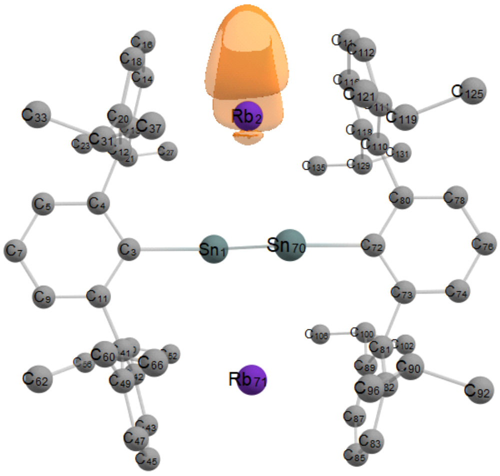

249. LV(1) Rb2

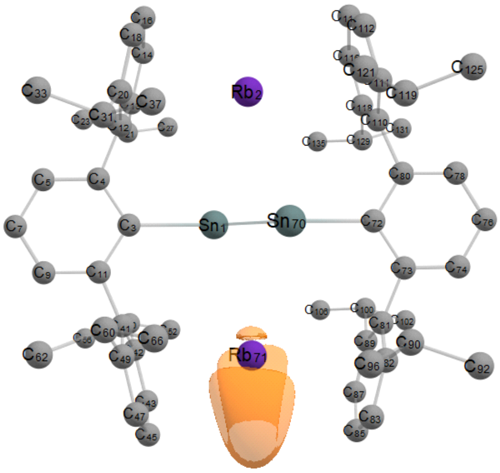

250. LV(1) Rb71

Figure S84. Selected beta NBO orbitals for compound **$[LSnRb]_2^{\bullet-}$** radical anion, **$1^{Rb\bullet-}$**.

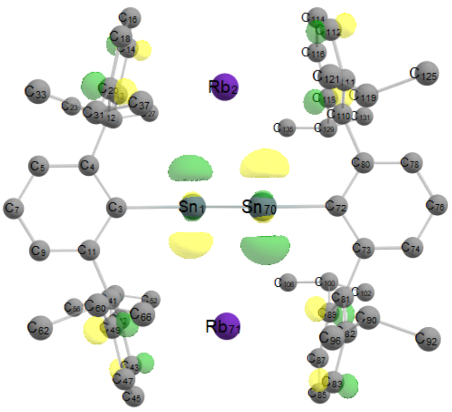

SOMO
0.48 eV

Figure S85. Alpha SOMO of **[LSnRb]$_2^{\bullet-}$** radical anion.

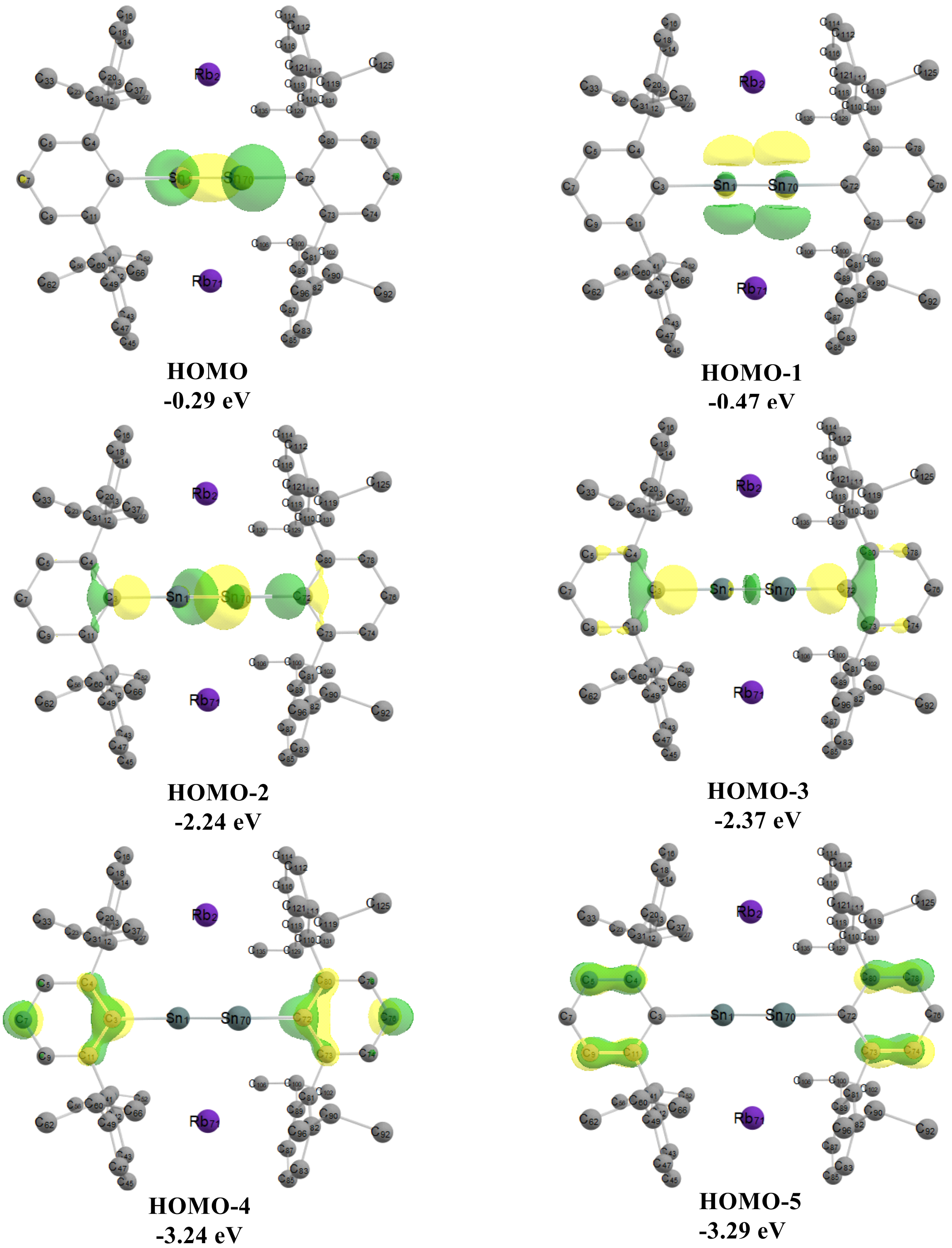

Figure S86. Alpha molecular orbitals HOMO-5 to HOMO for compound **[LSnRb]$_2$$^{\bullet-}$** radical anion, **1$^{Rb\bullet-}$**.

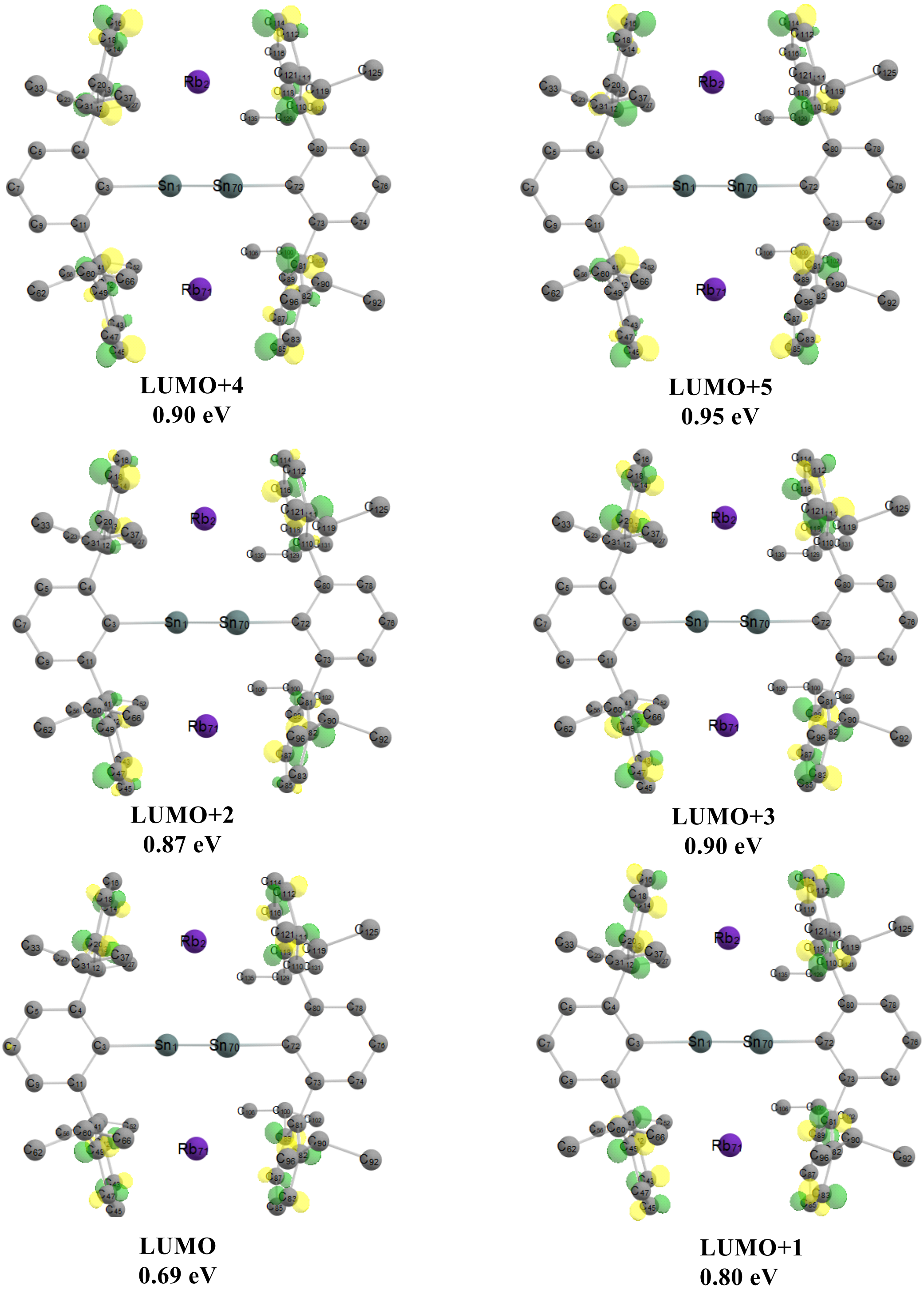

Figure S87. Alpha molecular orbitals LUMO to LUMO+5 for compound **[LSnRb]$_2$$^{\bullet-}$** radical anion, **1$^{Rb\bullet-}$**.

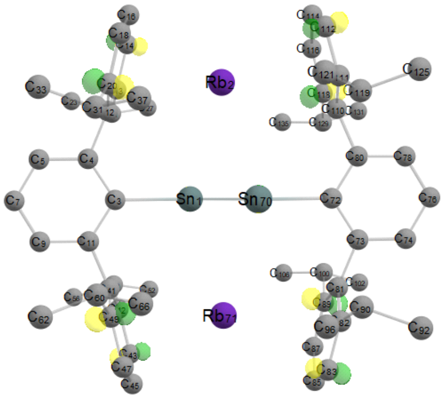

SOMO
0.67 eV

Figure S88. Beta SOMO of **[LSnRb]**$_2^{\cdot-}$ radical anion, **1**$^{\mathbf{Rb\bullet-}}$**.**

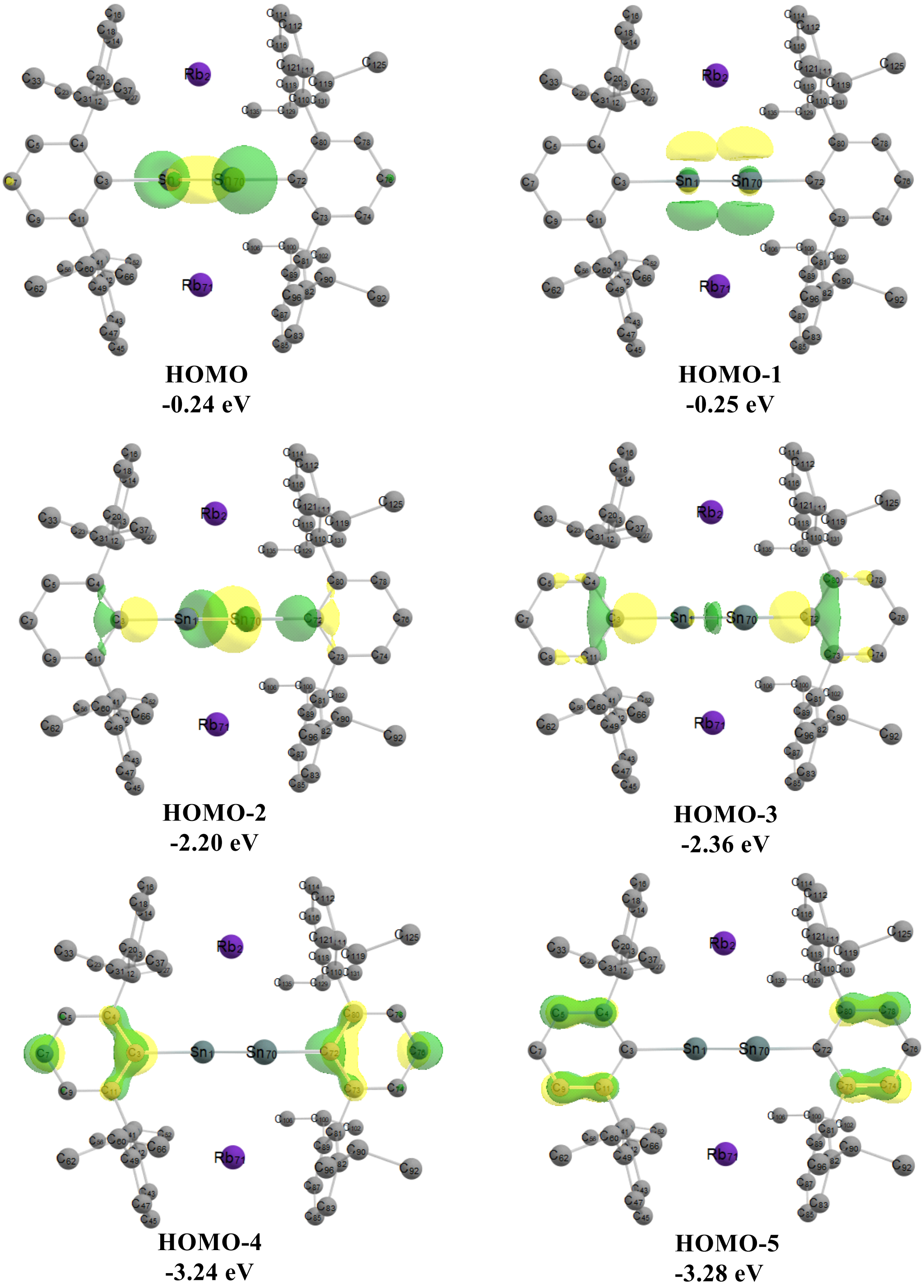

Figure S89. Beta molecular orbitals HOMO-5 to HOMO for compound **$[LSnRb]_2^{\bullet-}$** radical anion, **$1^{Rb\bullet-}$**.

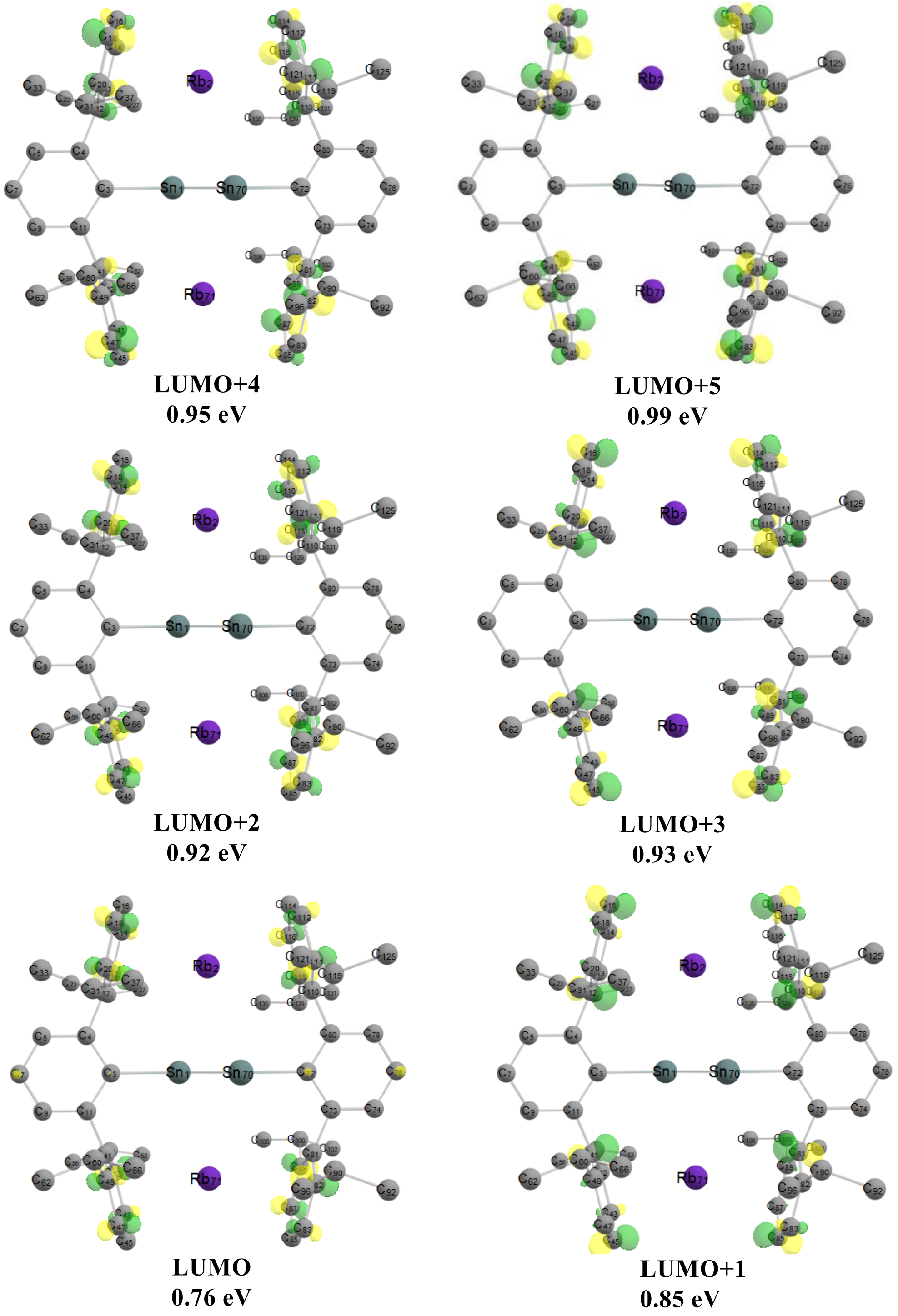

Figure S90. Beta molecular orbitals LUMO to LUMO+5 for compound **$[LSnRb]_2^{\bullet-}$** radical anion, **$1^{Rb\bullet-}$**.

Table S36. Selected Mulliken spin density values for **$[LSnRb]_2^{\bullet-}$** radical anion ($\mathbf{1}^{\mathbf{Rb}\bullet-}$) with hydrogens summed into heavy atoms. (All other atoms have a Mulliken spin density value < |±0.05|).

Atoms	Mulliken spin density
Sn1	0.268
Rb2	-0.001(*)
C14	0.053
C20	0.059
C43	0.053
C49	0.059
Sn70	0.268
Rb71	-0.001(*)
C83	0.059
C89	0.059
C112	0.053
C118	0.059

(*) Rb atoms show no detectable spin density within the set threshold.

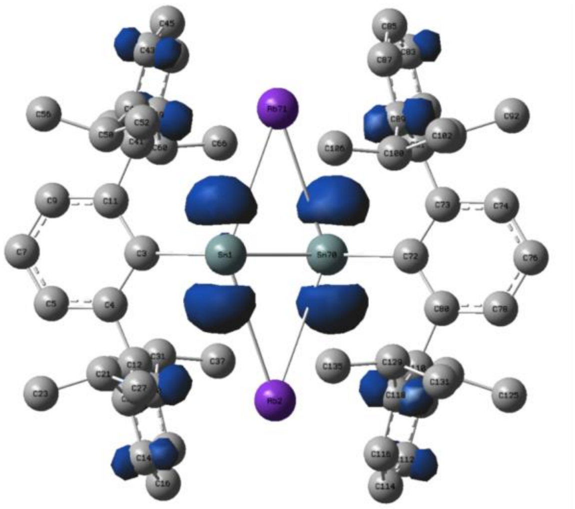

Figure S91. Spin density plot of compound $\mathbf{1}^{\mathbf{Rb}\bullet-}$.

M = Cs

Table S37. Selected donor - acceptor interaction energies, $E^{(2)}$, in kcal mol^{-1} for **$[LSnCs]_2^{\bullet-}$** radical anion, **$1^{Cs\bullet-}$.** (Alpha Spin NBO).

Donor Orbital	Acceptor Orbital	$E^{(2)}$ kcal/mol
87. LP(1) Sn1	250. LV(1) Cs2	1.5
88. LP(2) Sn1	250. LV(1) Cs2	10.5
89. LP(1) Sn70	250. LV(1) Cs2	1.3
90. LP(2) Sn70	250. LV(1) Cs2	10.4
92. BD(1) Sn1-Sn70	250. LV(1) Cs2	0.6
87. LP(1) Sn1	251. LV(1) Cs71	1.3
88. LP(2) Sn1	251. LV(1) Cs71	10.4
89. LP(1) Sn70	251. LV(1) Cs71	1.5
90. LP(2) Sn70	251. LV(1) Cs71	10.5
92. BD(1) Sn1-Sn70	251. LV(1) Cs71	0.6

Table S38. Selected donor - acceptor interaction energies, $E^{(2)}$, in kcal mol^{-1} for **$[LSnCs]_2^{\bullet-}$, $1^{Cs\bullet-}$.** radical anion. (Beta Spin NBO).

Donor Orbital	Acceptor Orbital	$E^{(2)}$ kcal/mol
87. LP(1) Sn1	249. LV(1) Cs2	1.4
88. LP(1) Sn70	249. LV(1) Cs2	1.3
90. BD(1) Sn1-Sn70	249. LV(1) Cs2	0.7
91. BD(2) Sn1-Sn70	249. LV(1) Cs2	20.6
87. LP (1) Sn1	250. LV(1) Cs71	1.3
88. LP (1) Sn70	250. LV(1) Cs71	1.4
90. BD(1) Sn1-Sn70	250. LV(1) Cs71	0.7
91. BD(2) Sn1-Sn70	250. LV(1) Cs71	20.6

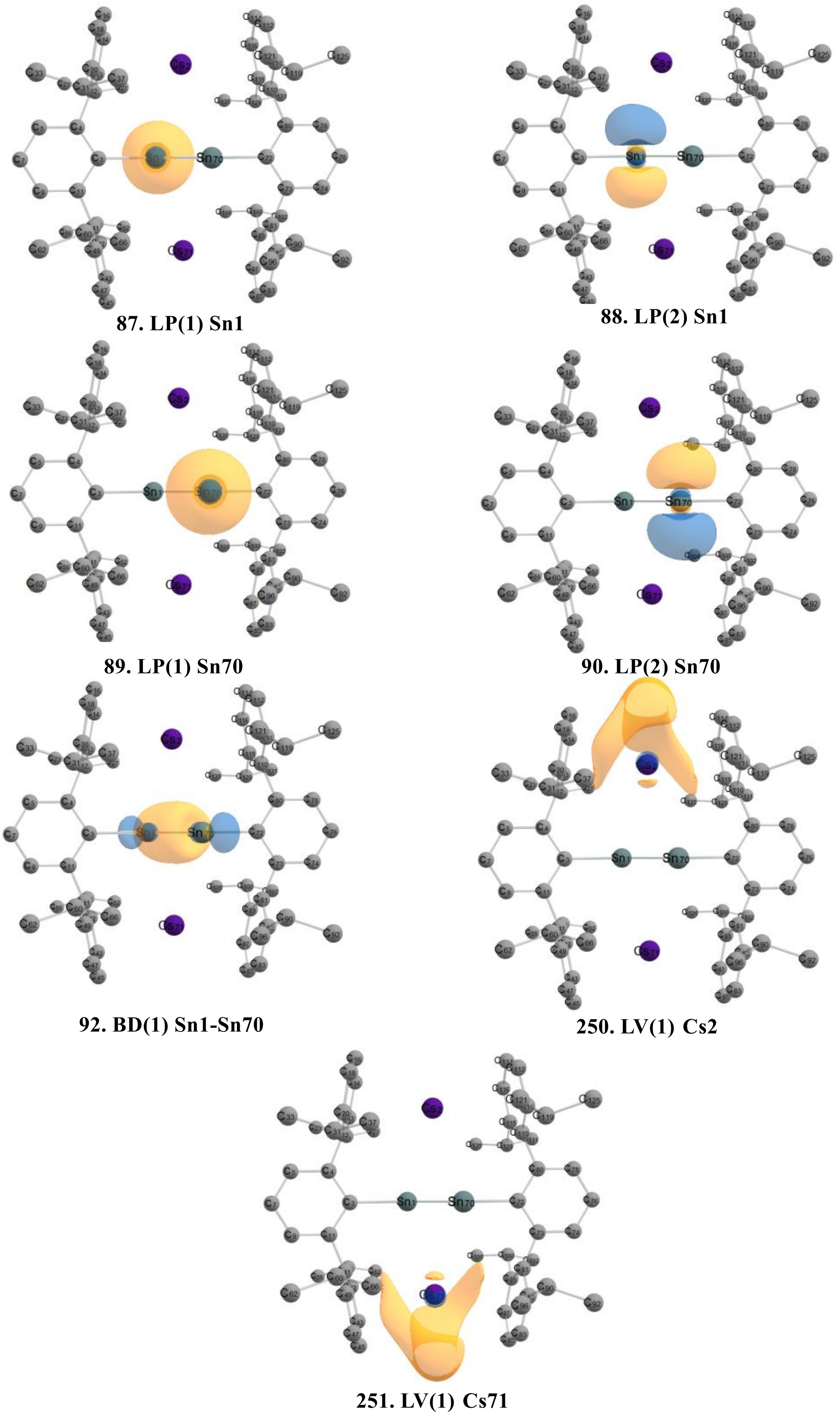

Figure S92. Selected alpha NBO orbitals for compound **$[LSnCs]_2^{\bullet-}$** radical anion, **$1^{Cs\bullet-}$.**

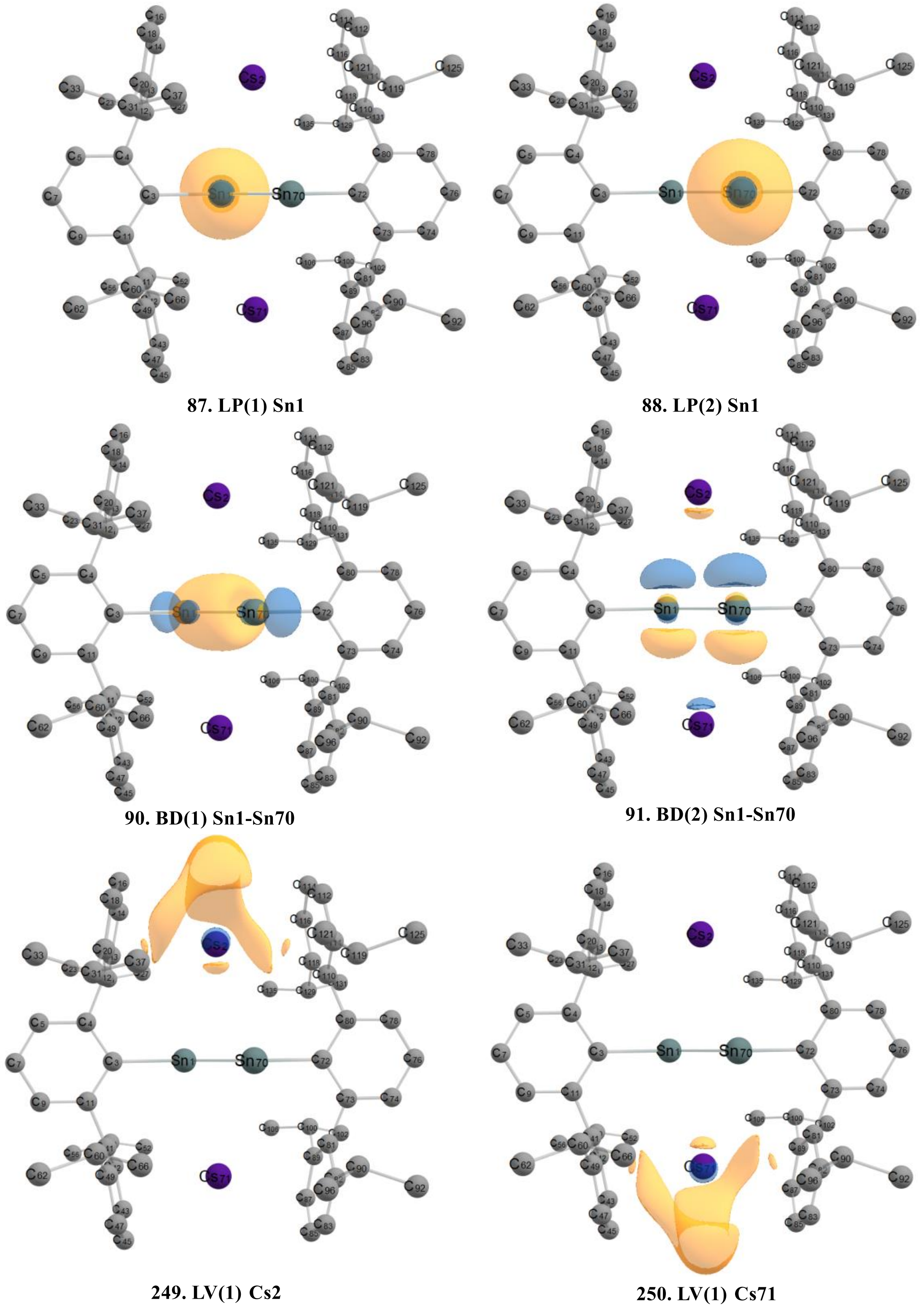

Figure S93. Selected beta NBO orbitals for compound **$[LSnCs]_2^{\bullet-}$** radical anion, **$1^{Cs\bullet-}$**.

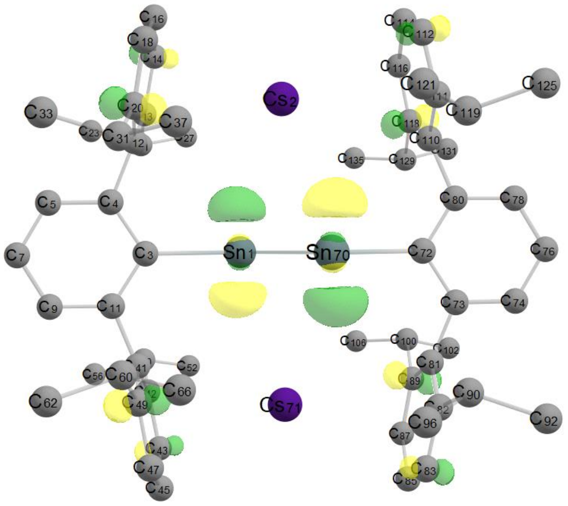

SOMO
0.48 eV

Figure S94. Alpha SOMO of **$[LSnCs]_2^{\bullet-}$** radical anion, **$1^{Cs\bullet-}$.**

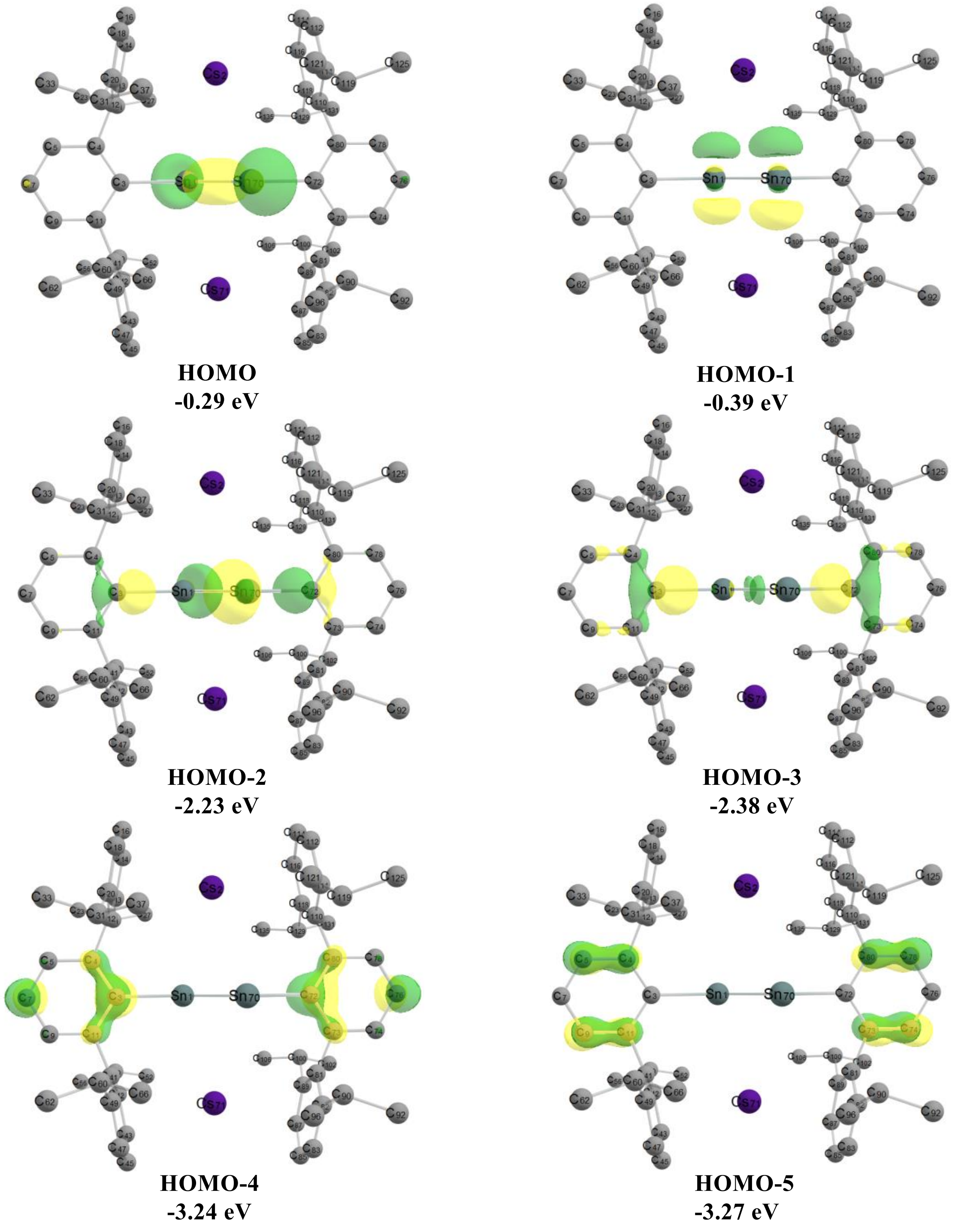

Figure S95. Alpha molecular orbitals HOMO-5 to HOMO for compound **$[LSnCs]_2^{\bullet-}$** radical anion, **$1^{Cs\bullet-}$**.

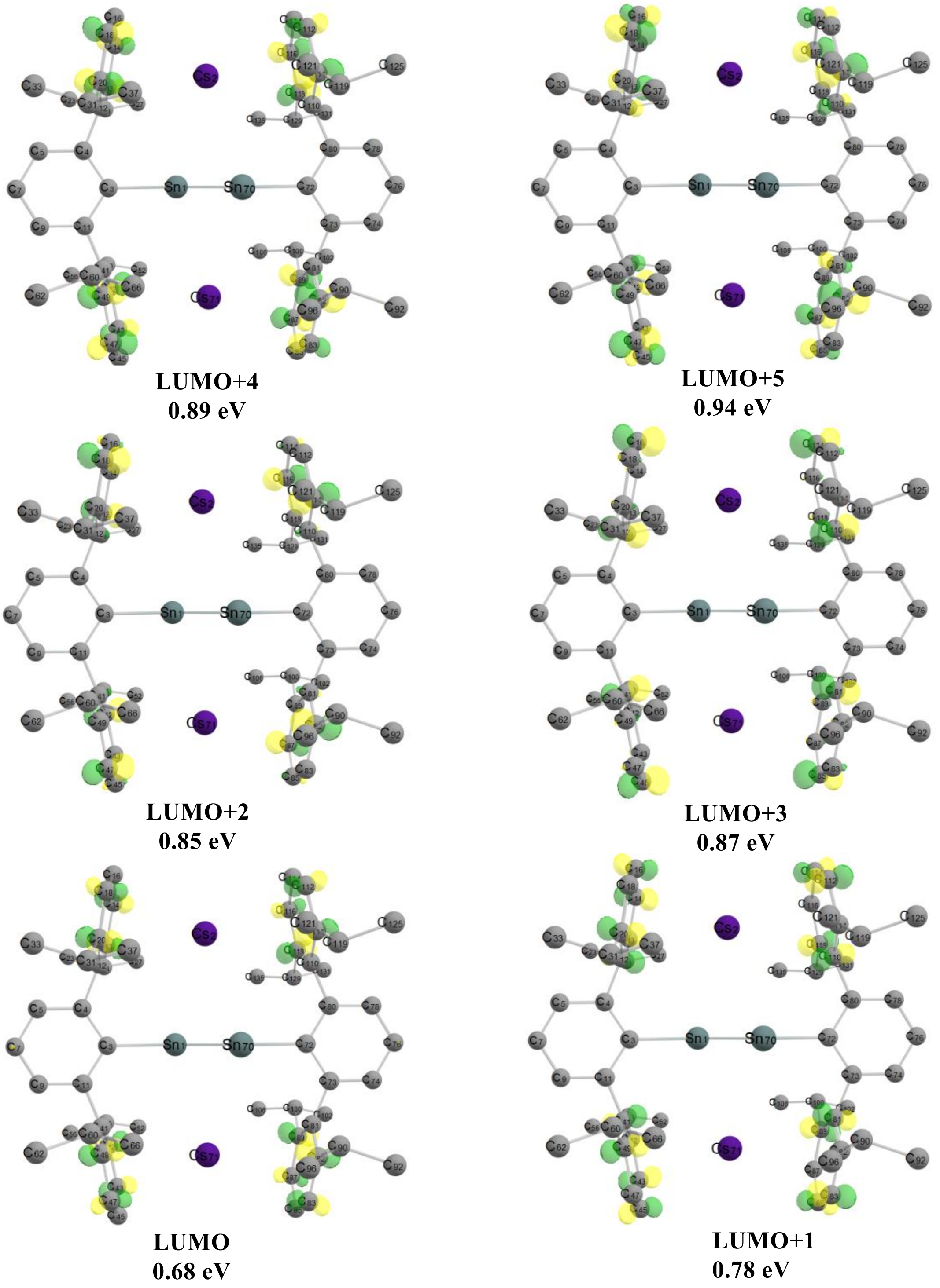

Figure S96. Alpha molecular orbitals LUMO to LUMO+5 for compound **[LSnCs]$_2$$^{\bullet-}$** radical anion, **1$^{Cs\bullet-}$.**

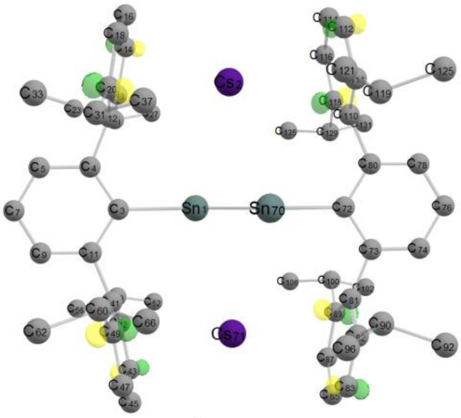

SOMO
0.67 eV

Figure S97. Beta SOMO of **[LSnCs]$_2^{\bullet-}$** radical anion, **1$^{Cs\bullet-}$.**

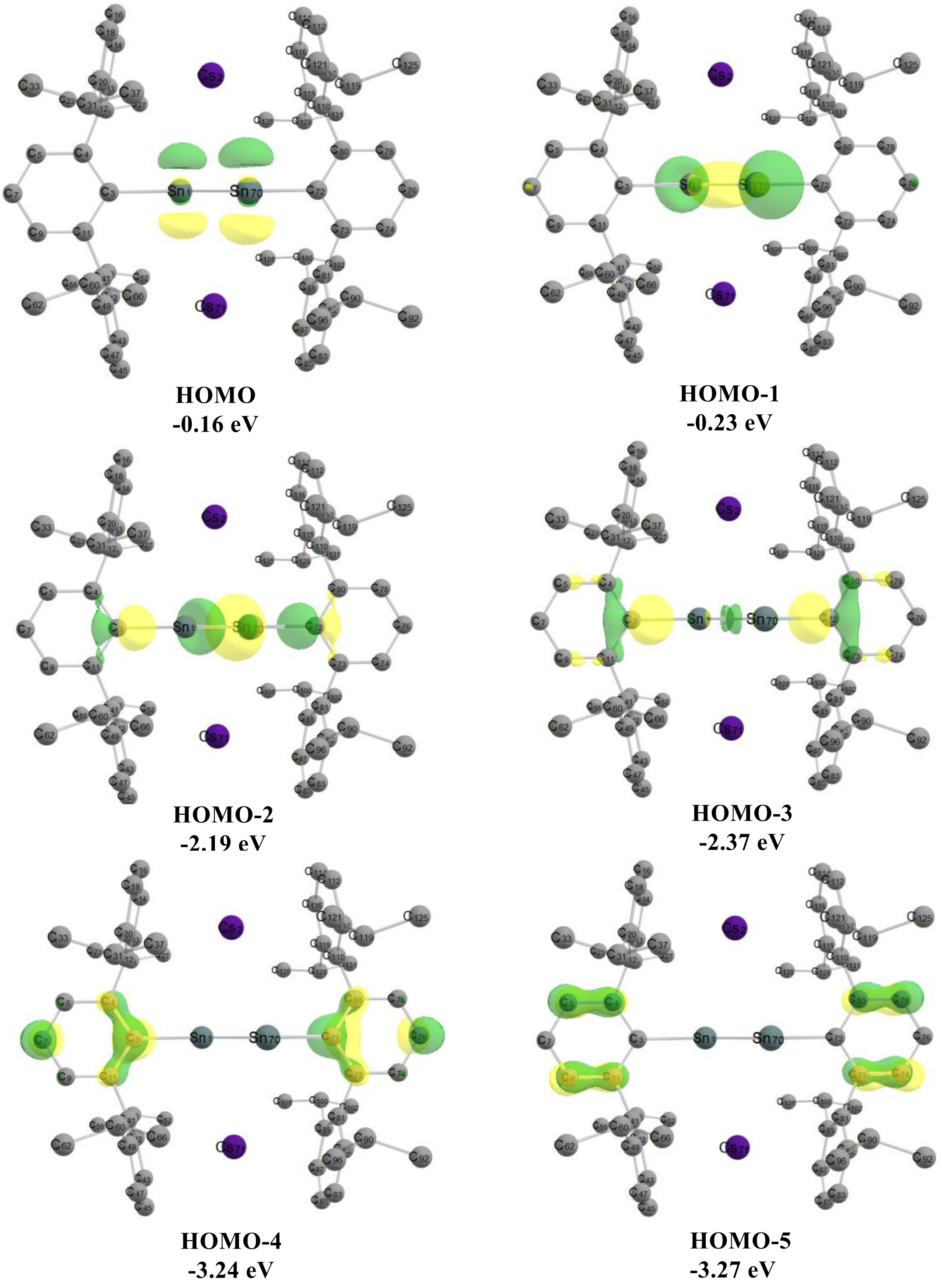

Figure S98. Beta molecular orbitals HOMO-5 to HOMO for compound **$[LSnCs]_2^{\bullet-}$** radical anion, **$1^{Cs\bullet-}$.**

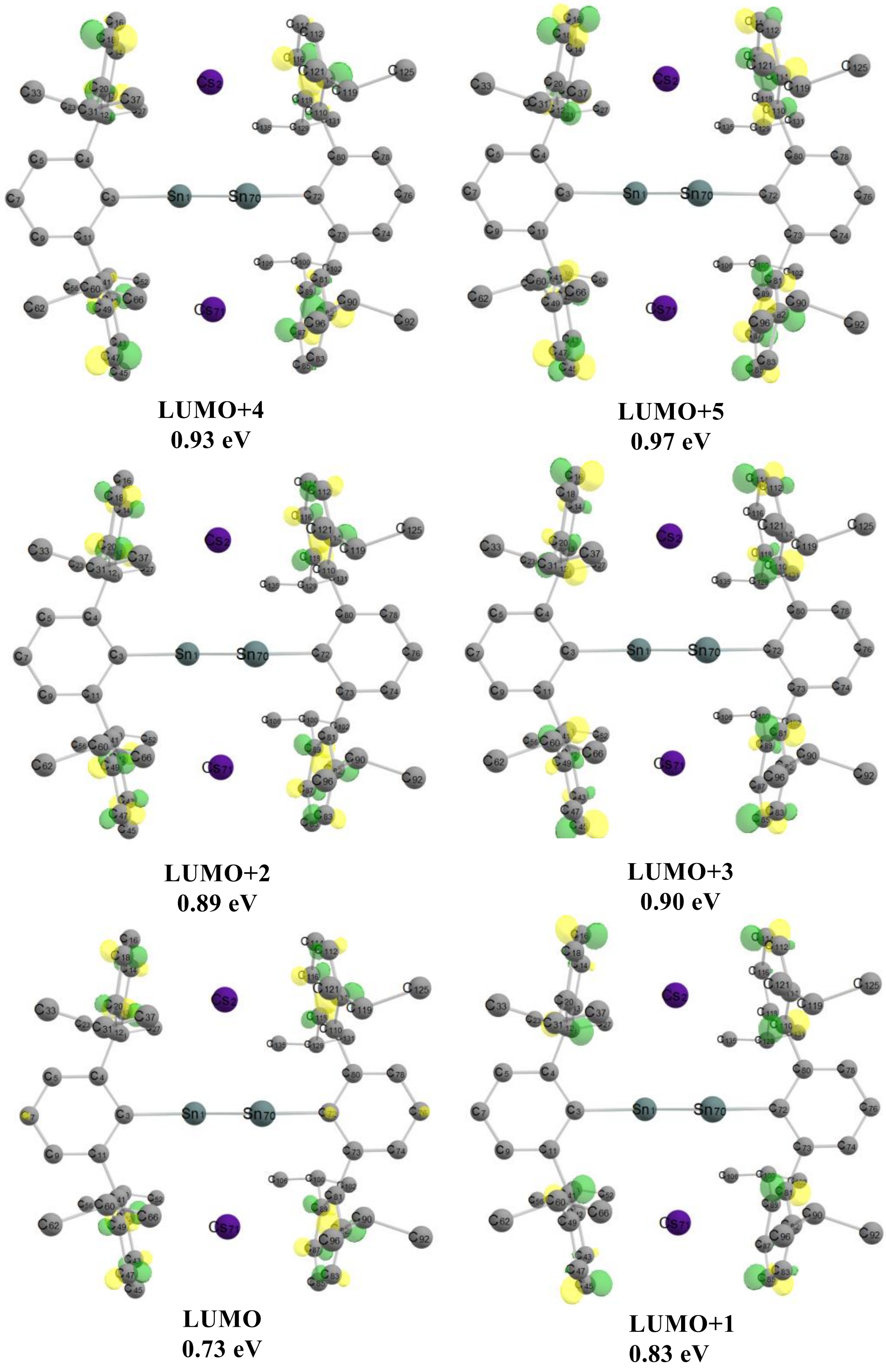

Figure S99. Beta molecular orbitals LUMO to LUMO+5 for compound **$[LSnCs]_2^{\bullet-}$** radical anion, **$1^{Cs\bullet-}$.**

Table S39. Selected Mulliken spin density values for **[LSnCs]$_2^{\bullet-}$** radical anion (**1**$^{Cs\bullet-}$) with hydrogens summed into heavy atoms. (Rest of atoms presents Mulliken spin density value < |±0.03|).

**Atoms**	**Mulliken spin density**
Sn1	0.290
Cs2$^{(*)}$	0.020
C3	-0.033
C14	0.049
C20	0.051
C43	0.048
C49	0.052
Sn70	0.290
Cs71$^{(*)}$	0.020
C72	-0.033
C83	0.049
C89	0.051
C112	0.048
C118	0.052

(*) Cs atoms show no detectable spin density within the set threshold.

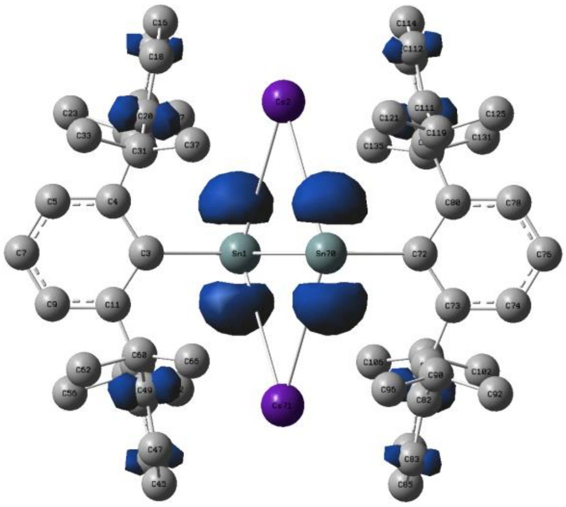

Figure S100. Spin density plot of compound **1**$^{Cs\bullet-}$**.**

Calculation of hyperfine coupling constant in Sn atoms, a(Sn) for compound 1$^{Na\bullet-}$.

The experimental hyperfine coupling constant (HFCC) from the reduction of **1**^{Na} by K(0) expressed in Gauss (G) was converted into megahertz (MHz) to allow direct comparison with the computed HFCCs following Eq 1. In Equation 1, a(G) and a(MHz) denote the HFCC expressed in Gauss and megahertz (MHz), respectively, whereas h and μ_B parameters represent the Planck constant (J.s) and the Bohr magneton (J.T^{-1}). **Note:** 1 T = 10^4 G.

$$a(T) = \frac{a(MHz) \times h}{\mu_B \times g} \rightarrow a(MHz) = \frac{a(T) \times \mu_B \times g}{h} \quad (Eq. 1)$$

If g = 2.0022 and a(Sn) = 20.5 G, a(Sn) = 57.4561 MHz, which can be directly compared to the computed HFCC at the ωB97X-D4/BS6 level of theory, 58.5078 MHz (an average of the HFCC of both Sn atoms). The computed Sn hyperfine coupling constant (58.5078 MHz) was converted to Gauss using the experimental g-value, in order to be able to compare it against Power's report[2] giving a a(Sn) = 20.8764 G.

Table S40. Methodology testing for the calculation of Sn-Sn hyperfine coupling constants in $\mathbf{1}^{\mathbf{Na\bullet-}}$.

Functional	Hartree-Fock Exchange	Basis Set	a(Sn0)/MHz	a(Sn69) /MHz
BP86- D3BJ [9]	0	BS3[18]	215.7426	215.7379
		BS6[28]	172.7277	172.7656
TPSS- D3BJ [9c]	0	BS3	152.2314	152.2430
		BS6	102.9659	103.0298
BLYP- D3BJ [30]	0	BS3	130.2026	130.1875
		BS6	86.7487	86.7685
M06-D4 [10]	27	BS3	1186.8201	1186.9850
		BS6	1203.6708	1203.7082
M06-2X-D3 [10]	54	BS3	-691.2257	-691.7332
		BS6	-760.1288	-760.1118
PBE0- D3BJ [31]	25	BS3	235.1947	235.3997
		BS6	175.9140	175.9579
TPSSh- D3BJ [9c]	10	BS3	159.0120	158.8828
		BS6	102.1848	102.2564
B3LYP- D3BJ [32]	20	BS3	157.6225	157.5418
		BS6	98.5969	98.6368
ωB97X-D4 [33]	22% short-range, 100% long-range	BS3	145.5468	145.5177
		BS6	58.5004	58.5152
CAM-B3LYP- D3BJ [34]	19% short-range, 65% long-range	BS3	239.0053	238.8723
		BS6	166.3735	166.4039

Among all tested combinations, ωB97X-D4 with the BS6 basis set produced the closest agreement with experimental values.

EPR Spectroscopy

The EPR spectrum of the putative species $\mathbf{1}^{\mathrm{Na}\cdot-}$ was recorded on a Bruker EMX spectrometer (T = 298 K), utilising an ER4119HS resonator at 100 kHz modulation frequency and 3.0 G modulation depth, at 10 mW microwave power (scans = 100). Simulation of the experimental data was performed using the pepper function for anisotropic spectra in the Easyspin toolbox for Matlab (version 6.0.0-dev51), employing natural isotopic abundances for the ^{117,119}Sn nuclei.[35].

Comparison of the experimentally observed hyperfine coupling to the theoretical values (a(^{117}Sn) = 1497.98 mT; a(^{119}Sn) = 1567.18 mT) leads to a calculated spin density on each Sn nucleus of ~ 0.13% (*cf.* the calculated Mulliken spin density values *ca.* 0.3% cited in Table S30].

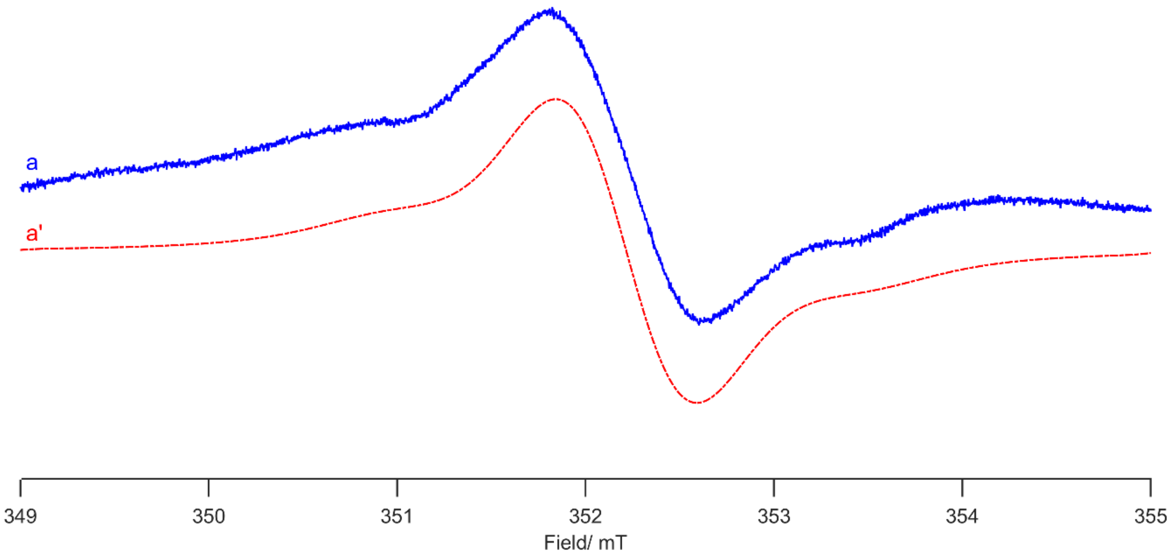

Figure S101. CW X-band EPR spectrum (T = 298 K) of $\mathbf{1}^{\mathrm{Na}\bullet-}$ (a) experimental, and (a') simulation. Experimental parameters: 100 kHz modulation, 3.0 G modulation depth, 10 mW power, 10.24 ms conversion time. Simulation parameters: g_{iso} = 2.0014; a_{iso}(Sn) = 57.49 MHz (implementing natural abundances); lwpp = [0.15 0.45] mT.

Computed Hartree Energies and Cartesian Coordinates

1^{Li}

Method A, B and C

SCF (BP86/BS1) Energy = -2352.92917103
Enthalpy 0K = -2351.784120
Enthalpy 298K = -2351.709736
Free Energy 298K = -2351.897197
Lowest Frequency = 11.4391 cm^{-1}
Second Frequency = 20.7920 cm^{-1}
SCF (BP86-$D3^{BJ}$) Energy = -2353.34439499
SCF (C_6H_6) Energy = -2352.93893446
SCF (BS2) Energy = -2775.47921934
SCF (BP86-$D3^{BJ}$(CPCM=C_6H_6)/BS3) Energy = - 14871.704791
SCF (BP86-$D3^{BJ}$(SMD=C_6H_6)/BS3) Energy = -14871.737696

Sn	-0.99955	-0.05630	1.00696
Li	0.00108	-2.90871	0.00101
C	-2.96642	-0.00734	-0.20866
C	-3.71307	1.20842	-0.29412
C	-5.06572	1.22577	-0.69765
H	-5.60369	2.18089	-0.73750
C	-5.72447	0.03763	-1.04475
H	-6.77734	0.05180	-1.34512
C	-5.00716	-1.16640	-1.01780
H	-5.49875	-2.10119	-1.31410
C	-3.65291	-1.19188	-0.61803
C	-2.92453	-2.50762	-0.67089
C	-2.30986	-2.91867	-1.89149
C	-1.59770	-4.13811	-1.91934
H	-1.12432	-4.46611	-2.85013
C	-1.50676	-4.94944	-0.77703
H	-0.96738	-5.90102	-0.82345
C	-2.13102	-4.54977	0.41626
H	-2.07404	-5.19796	1.29656
C	-2.85012	-3.33550	0.48972
C	-2.46783	-2.10686	-3.17981
H	-2.74333	-1.08043	-2.88258
C	-3.63261	-2.68197	-4.02255
H	-4.58109	-2.67691	-3.46116
H	-3.42180	-3.72329	-4.32601
H	-3.77542	-2.08405	-4.93968
C	-1.18355	-2.02077	-4.02743
H	-0.34251	-1.60550	-3.44452
H	-1.35363	-1.35902	-4.89430
H	-0.88076	-3.00415	-4.43029
C	-3.61420	-2.97385	1.76614
H	-3.76924	-1.88074	1.74984
C	-5.00753	-3.64915	1.75127
H	-5.59284	-3.34462	0.86837
H	-5.58012	-3.36994	2.65300
H	-4.91274	-4.74984	1.73570
C	-2.85918	-3.31724	3.06452
H	-1.86332	-2.84306	3.08152
H	-2.73587	-4.40670	3.20332
H	-3.42472	-2.94439	3.93556
C	-3.05263	2.52420	0.02082
C	-2.38641	3.23788	-1.02499
C	-1.75226	4.46287	-0.71470
H	-1.25584	5.03029	-1.50871
C	-1.78930	4.98750	0.59090
H	-1.30611	5.94625	0.80870
C	-2.46942	4.29633	1.60021
H	-2.51028	4.72013	2.60891
C	-3.10664	3.06272	1.33856
C	-2.43314	2.74588	-2.47608
H	-2.60720	1.65636	-2.43990
C	-1.13029	2.98399	-3.26390
H	-0.26245	2.50541	-2.77361
H	-0.91148	4.05806	-3.40191
H	-1.21753	2.54182	-4.27111
C	-3.63079	3.39341	-3.21301
H	-4.58428	3.16514	-2.71019
H	-3.69321	3.01892	-4.24984
H	-3.51945	4.49186	-3.25398
C	-3.90141	2.37046	2.44791
H	-4.07261	1.32998	2.12119
C	-5.28294	3.04911	2.61202
H	-5.85917	3.02423	1.67223
H	-5.17205	4.10613	2.91389
H	-5.87523	2.53552	3.38954
C	-3.15406	2.31558	3.79561
H	-2.17745	1.81573	3.67950
H	-3.74626	1.74133	4.52917
H	-2.99070	3.31992	4.22621
Sn	0.99906	-0.05583	-1.00597
Li	-0.00117	2.76481	0.00021
C	2.96637	-0.00565	0.20879
C	3.65372	-1.18974	0.61793
C	5.00819	-1.16348	1.01696
H	5.50048	-2.09800	1.31295
C	5.72482	0.04096	1.04348
H	6.77784	0.05577	1.34329
C	5.06521	1.22870	0.69661
H	5.60268	2.18412	0.73607
C	3.71238	1.21056	0.29373
C	3.05113	2.52590	-0.02125
C	2.38460	3.23931	1.02456
C	1.74964	4.46386	0.71419
H	1.25294	5.03107	1.50818
C	1.78623	4.98837	-0.59148
H	1.30236	5.94676	-0.80933
C	2.46669	4.29753	-1.60075
H	2.50725	4.72124	-2.60950
C	3.10467	3.06431	-1.33903
C	2.43196	2.74762	2.47572
H	2.60682	1.65823	2.43972
C	3.62927	3.39616	3.21231
H	4.58282	3.16843	2.70935
H	3.51719	4.49453	3.25306
H	3.69214	3.02193	4.24920
C	1.12902	2.98493	3.26363
H	0.26149	2.50573	2.77337
H	1.21659	2.54289	4.27087
H	0.90951	4.05886	3.40158
C	3.89967	2.37246	-2.44848
H	4.07233	1.33235	-2.12137
C	5.28028	3.05271	-2.61369
H	5.85702	3.02909	-1.67418
H	5.87280	2.53941	-3.39122
H	5.16797	4.10942	-2.91611
C	3.15156	2.31602	-3.79571
H	2.17579	1.81472	-3.67873
H	2.98636	3.31997	-4.22650

H	3.74415	1.74243	-4.52947
C	2.92630	-2.50601	0.67106
C	2.31141	-2.91700	1.89159
C	1.60066	-4.13725	1.91982
H	1.12723	-4.46528	2.85058
C	1.51127	-4.94937	0.77792
H	0.97304	-5.90159	0.82466
C	2.13555	-4.54962	-0.41529
H	2.07958	-5.19829	-1.29531
C	2.85326	-3.33455	-0.48913
C	2.46784	-2.10407	3.17940
H	2.74098	-1.07721	2.88142
C	1.18361	-2.02029	4.02728
H	0.34149	-1.60730	3.44430
H	0.88319	-3.00404	4.43102
H	1.35239	-1.35748	4.89359
C	3.63407	-2.67605	4.02227
H	4.58247	-2.66902	3.46077
H	3.77558	-2.07733	4.93909
H	3.42569	-3.71769	4.32627
C	3.61712	-2.97265	-1.76560
H	3.77265	-1.87962	-1.74884
C	5.01015	-3.64855	-1.75150
H	5.59582	-3.34479	-0.86856
H	4.91494	-4.74921	-1.73652
H	5.58264	-3.36908	-2.65321
C	2.86140	-3.31507	-3.06385
H	1.86600	-2.83989	-3.08040
H	3.42703	-2.94259	-3.93500
H	2.73697	-4.40439	-3.20281

Methods D and E

SCF (BP86/BS4) Energy = -2773.21804728
Enthalpy 0K = -2772.078571
Enthalpy 298K = -2772.004142
Free Energy 298K = -2772.191332
Lowest Frequency = 14.2643 cm^{-1}
Second Frequency = 20.1492 cm^{-1}
SCF (BP86-D3^{BJ}) Energy = -2773.63296143
SCF (PCM=C_6H_6) Energy = -2773.22742312
SCF (SMD=C_6H_6) Energy = -2773.25581052
SCF (BS5) Energy = -2775.72802664

Sn	-1.00055	-0.06321	0.98691
Li	0.00513	-2.84493	0.00376
C	-2.94855	-0.01091	-0.23862
C	-3.70022	1.20440	-0.29506
C	-5.04947	1.22176	-0.71489
H	-5.59882	2.17818	-0.73664
C	-5.69589	0.03696	-1.10154
H	-6.75127	0.05212	-1.41650
C	-4.97052	-1.16452	-1.09662
H	-5.45769	-2.09901	-1.42285
C	-3.61853	-1.19299	-0.68366
C	-2.88139	-2.50305	-0.75714
C	-2.24584	-2.88345	-1.97949
C	-1.52905	-4.10152	-2.02548
H	-1.03553	-4.40693	-2.96097
C	-1.45168	-4.93995	-0.90094
H	-0.90373	-5.89322	-0.96201
C	-2.09312	-4.56967	0.29387
H	-2.04277	-5.24083	1.16514
C	-2.81717	-3.35785	0.38739
C	-2.38214	-2.03824	-3.24887
H	-2.66248	-1.01502	-2.92268
C	-3.53419	-2.58112	-4.12591
H	-4.49638	-2.59929	-3.57601
H	-3.31964	-3.61640	-4.46890
H	-3.66935	-1.94681	-5.02750
C	-1.08355	-1.92596	-4.06780
H	-0.24656	-1.53009	-3.45321
H	-1.23276	-1.23061	-4.92023
H	-0.77104	-2.90057	-4.50089
C	-3.59172	-3.02670	1.66598
H	-3.74370	-1.92681	1.67164
C	-4.98681	-3.69253	1.62731
H	-5.56664	-3.37297	0.73837
H	-5.57311	-3.42160	2.53075
H	-4.90022	-4.80020	1.59735
C	-2.84602	-3.39647	2.96041
H	-1.83905	-2.93128	2.99275
H	-2.73192	-4.49518	3.08467
H	-3.41165	-3.03054	3.84239
C	-3.05376	2.51490	0.06817
C	-2.38288	3.26768	-0.94939
C	-1.77016	4.49421	-0.59626
H	-1.27246	5.09656	-1.37236
C	-1.83137	4.98156	0.72324
H	-1.36303	5.94647	0.97592
C	-2.51130	4.25029	1.70587
H	-2.56593	4.64617	2.73200
C	-3.12801	3.01403	1.40300
C	-2.41114	2.82218	-2.41653
H	-2.60309	1.72871	-2.41428
C	-1.08986	3.05755	-3.17010
H	-0.23555	2.53713	-2.68291
H	-0.83832	4.13566	-3.26424
H	-1.16226	2.65260	-4.20083
C	-3.58573	3.50431	-3.15460
H	-4.55604	3.28630	-2.66562
H	-3.64640	3.14888	-4.20510
H	-3.45574	4.60792	-3.17545
C	-3.92008	2.27664	2.48530
H	-4.05649	1.23430	2.12823
C	-5.32351	2.90512	2.64525
H	-5.88796	2.89329	1.69088
H	-5.25415	3.96140	2.98412
H	-5.91808	2.34653	3.39899
C	-3.18926	2.20672	3.83916
H	-2.18495	1.74913	3.72257
H	-3.76663	1.58097	4.55175
H	-3.07166	3.20705	4.30896
Sn	0.99986	-0.06150	-0.98665
Li	-0.00233	2.69313	-0.00080
C	2.94805	-0.00564	0.23825
C	3.62038	-1.18659	0.68271
C	4.97268	-1.15588	1.09456
H	5.46177	-2.08961	1.42008
C	5.69598	0.04684	1.09919
H	6.75159	0.06376	1.41330
C	5.04718	1.23060	0.71333
H	5.59485	2.18798	0.73492
C	3.69768	1.21097	0.29441
C	3.04870	2.52041	-0.06807
C	2.37619	3.27119	0.94990
C	1.76106	4.49672	0.59749
H	1.26185	5.09743	1.37387
C	1.82159	4.98512	-0.72165

H	1.35145	5.94930	-0.97374
C	2.50310	4.25586	-1.70468
H	2.55718	4.65258	-2.73051
C	3.12214	3.02057	-1.40256
C	2.40510	2.82471	2.41673
H	2.59777	1.73137	2.41365
C	3.57949	3.50712	3.15491
H	4.54979	3.29002	2.66553
H	3.44877	4.61062	3.17646
H	3.64066	3.15108	4.20516
C	1.08397	3.05874	3.17095
H	0.22968	2.53854	2.68350
H	1.15691	2.65270	4.20121
H	0.83207	4.13664	3.26638
C	3.91547	2.28524	-2.48533
H	4.05383	1.24297	-2.12883
C	5.31770	2.91641	-2.64520
H	5.88237	2.90509	-1.69097
H	5.91316	2.35935	-3.39938
H	5.24630	3.97275	-2.98346
C	3.18457	2.21471	-3.83912
H	2.18104	1.75541	-3.72262
H	3.06523	3.21505	-4.30844
H	3.76287	1.59025	-4.55207
C	2.88626	-2.49836	0.75670
C	2.25152	-2.87975	1.97926
C	1.53942	-4.10056	2.02637
H	1.04683	-4.40684	2.96206
C	1.46592	-4.94070	0.90280
H	0.92181	-5.89611	0.96482
C	2.10605	-4.56911	-0.29224
H	2.05838	-5.24129	-1.16289
C	2.82523	-3.35447	-0.38702
C	2.38419	-2.03247	3.24766
H	2.65896	-1.00817	2.92008
C	1.08564	-1.92599	4.06740
H	0.24627	-1.53475	3.45312
H	0.77836	-2.90171	4.50173
H	1.23198	-1.22904	4.91901
C	3.53957	-2.56839	4.12461
H	4.50154	-2.58196	3.57424
H	3.67182	-1.93245	5.02548
H	3.33062	-3.60443	4.46874
C	3.59776	-3.02137	-1.66630
H	3.74785	-1.92124	-1.67158
C	4.99400	-3.68485	-1.62975
H	5.57438	-3.36481	-0.74132
H	4.90938	-4.79269	-1.60030
H	5.57873	-3.41241	-2.53375
C	2.85081	-3.39168	-2.95990
H	1.84322	-2.92774	-2.99067
H	3.41480	-3.02470	-3.84251
H	2.73794	-4.49049	-3.08437

Method F

SCF (BP86/BS4) Energy = -2771.87768177

Enthalpy 0K = -2770.692377

Enthalpy 298K = -2770.621213

Free Energy 298K = -2770.799380

Lowest Frequency = 16.3793 cm^{-1}

Second Frequency = 18.7574 cm^{-1}

SCF (M062X-D3) Energy = -2771.89959494

SCF (SMD=C_6H_6) Energy = -2771.91544453

SCF (BS5) Energy = -2774.47334470

Sn	0.90520	-0.06690	-1.03571
Sn	-0.90511	-0.06652	1.03546
C	2.86186	-0.01307	0.15998
C	3.60167	1.19019	0.23455
C	4.94636	1.21247	0.62493
H	5.48172	2.16549	0.65426
C	5.60203	0.03435	0.97233
H	6.65476	0.04774	1.25766
C	2.93896	2.49949	-0.07441
C	2.94160	3.00835	-1.39001
C	2.31248	4.23660	-1.64474
H	2.30716	4.63944	-2.65883
C	1.69438	4.94857	-0.62351
H	1.21666	5.90755	-0.83435
C	1.68261	4.43503	0.67593
H	1.19545	5.00047	1.47157
C	2.29207	3.20738	0.97086
C	3.65807	2.27442	-2.51293
H	3.73457	1.21869	-2.21341
C	5.07968	2.82828	-2.67097
H	5.05122	3.89473	-2.94494
H	5.61814	2.28588	-3.46215
H	5.65147	2.73074	-1.73776
C	2.90275	2.31863	-3.84138
H	1.87681	1.94086	-3.72070
H	3.41219	1.68046	-4.57785
H	2.86566	3.33511	-4.26312
C	2.28161	2.67563	2.39891
H	2.32157	1.57749	2.33031
C	3.52497	3.15675	3.15738
H	4.44933	2.83433	2.66165
H	3.52720	2.75508	4.18152
H	3.53166	4.25633	3.21992
C	1.01868	3.04403	3.17977
H	0.97074	4.12102	3.40574
H	1.00832	2.50865	4.14034
H	0.09996	2.75440	2.64470
C	-2.86185	-0.01284	-0.16007
C	-3.53438	-1.17919	-0.59368
C	-4.88286	-1.15737	-0.97299
H	-5.37028	-2.08785	-1.27636
C	-5.60214	0.03452	-0.97199
H	-6.65492	0.04788	-1.25716
C	-2.79222	-2.47668	-0.71176
C	-2.17510	-2.80701	-1.94143
C	-1.46037	-4.01017	-2.03763
H	-0.97418	-4.27689	-2.97698
C	-1.36896	-4.87788	-0.95068
H	-0.81907	-5.81575	-1.04683
C	-1.96728	-4.53928	0.26262
H	-1.87955	-5.21715	1.11302
C	-2.67727	-3.33742	0.40359
C	-2.30971	-1.90676	-3.16150
H	-2.44909	-0.88058	-2.78883
C	-1.08259	-1.90904	-4.07335
H	-0.16641	-1.65566	-3.52075
H	-1.21293	-1.15441	-4.86282
H	-0.94466	-2.87919	-4.57600
C	-3.55672	-2.29934	-3.96575
H	-3.46115	-3.33294	-4.33422
H	-3.67349	-1.63611	-4.83558
H	-4.46966	-2.23391	-3.36077
C	-3.35724	-3.00548	1.72295

H	-3.44561	-1.91002	1.77572
C	-4.77191	-3.59731	1.74395
H	-5.37699	-3.21072	0.91263
H	-5.28047	-3.34346	2.68565
H	-4.73227	-4.69473	1.65952
C	-2.55880	-3.46201	2.94395
H	-2.54642	-4.55889	3.04260
H	-3.01449	-3.05838	3.85936
H	-1.52085	-3.09835	2.89867
Li	0.00037	2.71065	-0.00022
C	3.53433	-1.17940	0.59378
C	4.88275	-1.15754	0.97329
H	5.37012	-2.08800	1.27679
C	2.79208	-2.47683	0.71191
C	2.67700	-3.33759	-0.40340
C	1.96678	-4.53931	-0.26241
H	1.87891	-5.21718	-1.11278
C	1.36841	-4.87778	0.95090
H	0.81835	-5.81555	1.04709
C	1.45999	-4.01006	2.03784
H	0.97378	-4.27669	2.97720
C	2.17492	-2.80702	1.94160
C	3.35709	-3.00582	-1.72274
H	3.44541	-1.91036	-1.77566
C	4.77181	-3.59758	-1.74348
H	4.73220	-4.69498	-1.65889
H	5.28044	-3.34385	-2.68518
H	5.37678	-3.21083	-0.91217
C	2.55884	-3.46258	-2.94376
H	1.52083	-3.09907	-2.89862
H	3.01456	-3.05900	-3.85917
H	2.54662	-4.55948	-3.04229
C	2.30968	-1.90677	3.16166
H	2.44943	-0.88065	2.78898
C	3.55649	-2.29974	3.96603
H	4.46950	-2.23467	3.36111
H	3.67343	-1.63649	4.83582
H	3.46054	-3.33327	4.33455
C	1.08247	-1.90861	4.07338
H	0.94410	-2.87873	4.57598
H	1.21300	-1.15406	4.86289
H	0.16645	-1.65487	3.52068
C	-3.60168	1.19042	-0.23459
C	-4.94642	1.21267	-0.62476
H	-5.48179	2.16568	-0.65407
C	-2.93888	2.49968	0.07431
C	-2.29174	3.20736	-0.97096
C	-1.68206	4.43491	-0.67606
H	-1.19471	5.00020	-1.47169
C	-1.69389	4.94856	0.62335
H	-1.21600	5.90746	0.83416
C	-2.31224	4.23680	1.64456
H	-2.30697	4.63972	2.65861
C	-2.94155	3.00864	1.38986
C	-2.28127	2.67547	-2.39896
H	-2.32107	1.57733	-2.33022
C	-1.01845	3.04397	-3.17994
H	-0.09965	2.75460	-2.64487
H	-1.00804	2.50842	-4.14041
H	-0.97072	4.12093	-3.40609
C	-3.52475	3.15632	-3.15742
H	-3.53159	4.25589	-3.22008
H	-3.52696	2.75454	-4.18151
H	-4.44904	2.83382	-2.66161
C	-3.65817	2.27487	2.51279
H	-3.73480	1.21914	2.21333
C	-5.07970	2.82892	2.67078
H	-5.65151	2.73139	1.73756
H	-5.61824	2.28666	3.46198
H	-5.05111	3.89539	2.94467
C	-2.90287	2.31906	3.84126
H	-2.86568	3.33556	4.26295
H	-3.41241	1.68099	4.57775
H	-1.87697	1.94117	3.72062
Li	-0.00023	-2.86301	0.00014

Li(0)

Method A, B and C

SCF (BP86/BS1) Energy = -7.47902967105
Enthalpy 0K = -7.479030
Enthalpy 298K = -7.476669
Free Energy 298K = -7.492432
SCF (BP86-D3^{BJ}) Energy = -7.47902967105
SCF (C_6H_6) Energy = -7.48809027803
SCF (BS2) Energy = -7.47933192055
SCF (BP86-D3^{BJ}(CPCM=C_6H_6)/BS3) Energy = -7.48312598
SCF (BP86-D3^{BJ}(SMD=C_6H_6)/BS3) Energy = -7.483409706

Li	0.00000	0.00000	0.00000

Methods D and E

SCF (BP86/BS4) Energy = -7.47319055602
Enthalpy 0K = -7.473191
Enthalpy 298K = -7.470830
Free Energy 298K = -7.486593
SCF (BP86-D3^{BJ}) Energy = -7.47319055602
SCF (PCM=C_6H_6) Energy = -7.483692
SCF (SMD=C_6H_6) Energy = -7.474213
SCF (BS5) Energy = -7.48081937506

Li	0.00000	0.00000	0.00000

Method F

SCF (M062X/BS4) Energy =-7.47198000349
Enthalpy 0K = -7.471980
Enthalpy 298K = -7.470830
Free Energy 298K = -7.485382
SCF (M062X-D3) Energy = -7.47198000349
SCF (SMD=C_6H_6) Energy = -7.47299405698
SCF (BS5) Energy = -7.48194203767

Li	0.00000	0.00000	0.00000

1^{Na}

Methods A, B and C

SCF (BP86/BS1) Energy = -2338.31813583
Enthalpy 0K = -2337.173940
Enthalpy 298K = -2337.099346
Free Energy 298K = -2337.285885
Lowest Frequency = 13.3959 cm^{-1}
Second Frequency = 21.1590 cm^{-1}
SCF (BP86-D3^{BJ}) Energy = -2338.72425127
SCF (C_6H_6) Energy = -2338.32786034
SCF (BS2) Energy = -3085.04728493
SCF (BP86-D3^{BJ}(CPCM=C_6H_6)/BS3) Energy = -15181.956793

SCF (BP86-D3^{BJ}(SMD=C_6H_6)/BS3) Energy = -15181.988841

Sn	-1.00446	-0.00021	1.02247
Na	0.00050	-2.99908	-0.00001
C	-3.01890	-0.00095	-0.14019
C	-3.74835	1.21028	-0.36803
C	-5.11111	1.20245	-0.74221
H	-5.62737	2.15737	-0.90023
C	-5.80396	-0.00222	-0.91770
H	-6.86223	-0.00269	-1.19876
C	-5.11007	-1.20626	-0.74204
H	-5.62549	-2.16166	-0.89994
C	-3.74732	-1.21284	-0.36784
C	-3.10116	-2.57118	-0.25404
C	-2.59700	-3.20799	-1.42942
C	-2.04410	-4.50491	-1.32246
H	-1.67314	-5.00914	-2.22163
C	-1.98623	-5.16829	-0.08506
H	-1.56528	-6.17790	-0.02108
C	-2.49975	-4.54396	1.06317
H	-2.47506	-5.07602	2.01986
C	-3.07106	-3.25378	1.00001
C	-2.69744	-2.54614	-2.80735
H	-2.93303	-1.48163	-2.63843
C	-3.86314	-3.16500	-3.61481
H	-4.82246	-3.05898	-3.08207
H	-3.69247	-4.24152	-3.79626
H	-3.95986	-2.66735	-4.59565
C	-1.38332	-2.60821	-3.61223
H	-0.55375	-2.12145	-3.06726
H	-1.50364	-2.07616	-4.57177
H	-1.08702	-3.64544	-3.85176
C	-3.73176	-2.65206	2.24312
H	-3.80840	-1.56372	2.07347
C	-5.16769	-3.21047	2.39608
H	-5.78022	-2.99362	1.50561
H	-5.66533	-2.75734	3.27136
H	-5.15282	-4.30555	2.54241
C	-2.92464	-2.86101	3.53920
H	-1.90702	-2.44664	3.44122
H	-2.84976	-3.92686	3.82140
H	-3.42066	-2.33949	4.37583
C	-3.10332	2.56919	-0.25450
C	-2.59955	3.20612	-1.42998
C	-2.04757	4.50345	-1.32324
H	-1.67692	5.00778	-2.22248
C	-1.99022	5.16710	-0.08597
H	-1.56994	6.17700	-0.02217
C	-2.50337	4.54264	1.06235
H	-2.47904	5.07487	2.01895
C	-3.07375	3.25204	0.99940
C	-2.69940	2.54385	-2.80776
H	-2.93351	1.47905	-2.63855
C	-1.38560	2.60753	-3.61302
H	-0.55513	2.12228	-3.06810
H	-1.09093	3.64508	-3.85316
H	-1.50540	2.07484	-4.57228
C	-3.86612	3.16096	-3.61509
H	-4.82520	3.05362	-3.08218
H	-3.96229	2.66307	-4.59586
H	-3.69699	4.23770	-3.79666
C	-3.73401	2.65002	2.24260
H	-3.80966	1.56157	2.07318
C	-5.17045	3.20716	2.39537
H	-5.78278	2.98954	1.50495
H	-5.15655	4.30230	2.54142
H	-5.66770	2.75382	3.27076
C	-2.92720	2.85998	3.53870
H	-1.90914	2.44670	3.44087
H	-3.42272	2.33806	4.37536
H	-2.85347	3.92596	3.82077
Sn	1.00461	0.00023	-1.02287
Na	-0.00071	2.99909	-0.00040
C	3.01898	0.00098	0.14004
C	3.74846	-1.21028	0.36762
C	5.11126	-1.20254	0.74167
H	5.62753	-2.15748	0.89946
C	5.80411	0.00211	0.91732
H	6.86241	0.00254	1.19827
C	5.11019	1.20618	0.74203
H	5.62561	2.16154	0.90012
C	3.74740	1.21284	0.36795
C	3.10112	2.57116	0.25461
C	2.59664	3.20742	1.43016
C	2.04343	4.50423	1.32359
H	1.67219	5.00803	2.22289
C	1.98555	5.16805	0.08643
H	1.56427	6.17754	0.02276
C	2.49946	4.54431	-1.06195
H	2.47475	5.07669	-2.01845
C	3.07113	3.25425	-0.99916
C	2.69709	2.54507	2.80784
H	2.93237	1.48055	2.63851
C	3.86308	3.16335	3.61533
H	4.82231	3.05721	3.08243
H	3.69273	4.23987	3.79709
H	3.95979	2.66539	4.59600
C	1.38315	2.60721	3.61299
H	0.55329	2.12109	3.06790
H	1.50345	2.07458	4.57221
H	1.08729	3.64441	3.85319
C	3.73211	2.65314	-2.24241
H	3.80895	1.56475	-2.07315
C	5.16795	3.21188	-2.39501
H	5.78048	2.99476	-1.50461
H	5.66573	2.75925	-3.27047
H	5.15287	4.30703	-2.54086
C	2.92511	2.86244	-3.53850
H	1.90749	2.44806	-3.44072
H	2.85026	3.92838	-3.82041
H	3.42120	2.34115	-4.37523
C	3.10326	-2.56909	0.25405
C	2.59939	-3.20598	1.42951
C	2.04701	-4.50313	1.32269
H	1.67626	-5.00742	2.22191
C	1.98942	-5.16666	0.08536
H	1.56881	-6.17642	0.02148
C	2.50278	-4.54230	-1.06292
H	2.47831	-5.07447	-2.01954
C	3.07358	-3.25188	-0.99988
C	2.69975	-2.54393	2.80736
H	2.93387	-1.47913	2.63823
C	1.38629	-2.60766	3.61316
H	0.55560	-2.12227	3.06867
H	1.09164	-3.64522	3.85326
H	1.50654	-2.07514	4.57245
C	3.86676	-3.16126	3.61411

H	4.82563	-3.05388	3.08084
H	3.96336	-2.66356	4.59493
H	3.69763	-4.23803	3.79554
C	3.73409	-2.65002	-2.24302
H	3.81004	-1.56160	-2.07358
C	5.17039	-3.20756	-2.39569
H	5.78272	-2.99009	-1.50524
H	5.15621	-4.30270	-2.54172
H	5.66782	-2.75438	-3.27106
C	2.92731	-2.85976	-3.53918
H	1.90932	-2.44629	-3.44136
H	3.42297	-2.33788	-4.37579
H	2.85340	-3.92571	-3.82130

Methods D and E
SCF (BP86/BS4) Energy = -3082.68960097
Enthalpy 0K = -3081.550552
Enthalpy 298K = -3081.475952
Free Energy 298K = -3081.663030
Lowest Frequency = 8.8552 cm^{-1}
Second Frequency = 20.2543 cm^{-1}
SCF (BP86-D3^{BJ}) Energy = -3083.09611769
SCF (PCM=C_6H_6) Energy = -3082.69815412
SCF (SMD=C_6H_6) Energy = -3082.72684055
SCF (BS5) Energy = -3085.30310049

Sn	-1.00871	0.00067	0.99960
Na	-0.00042	-2.94657	0.00039
C	-3.00141	0.00013	-0.17693
C	-3.72411	1.21497	-0.41045
C	-5.08173	1.20684	-0.80753
H	-5.60057	2.16609	-0.97430
C	-5.77158	0.00028	-0.99707
H	-6.83071	0.00034	-1.29955
C	-5.08209	-1.20635	-0.80675
H	-5.60118	-2.16556	-0.97294
C	-3.72446	-1.21463	-0.40965
C	-3.07505	-2.56891	-0.27375
C	-2.54505	-3.21300	-1.43566
C	-1.97687	-4.50288	-1.30526
H	-1.57793	-5.01425	-2.19617
C	-1.92811	-5.15071	-0.05843
H	-1.49080	-6.15870	0.02444
C	-2.46731	-4.52070	1.07593
H	-2.44552	-5.04406	2.04459
C	-3.05441	-3.23735	0.99061
C	-2.63083	-2.56635	-2.82203
H	-2.90401	-1.50291	-2.66327
C	-3.75753	-3.21863	-3.65400
H	-4.73725	-3.14860	-3.13929
H	-3.54997	-4.29454	-3.84054
H	-3.85471	-2.71780	-4.64050
C	-1.29424	-2.58732	-3.58687
H	-0.49181	-2.07092	-3.01541
H	-1.39858	-2.05735	-4.55675
H	-0.95348	-3.61978	-3.81738
C	-3.73793	-2.62899	2.21833
H	-3.80840	-1.53594	2.03674
C	-5.17775	-3.17760	2.34913
H	-5.77702	-2.97188	1.43945
H	-5.69676	-2.70769	3.21130
H	-5.17401	-4.27690	2.51302
C	-2.95422	-2.82704	3.52795
H	-1.92199	-2.43006	3.43907
H	-2.89931	-3.89447	3.83289
H	-3.45320	-2.28083	4.35543
C	-3.07431	2.56916	-0.27534
C	-2.54348	3.21209	-1.43753
C	-1.97486	4.50186	-1.30791
H	-1.57514	5.01226	-2.19902
C	-1.92663	5.15079	-0.06163
H	-1.48904	6.15871	0.02059
C	-2.46670	4.52196	1.07298
H	-2.44536	5.04619	2.04118
C	-3.05407	3.23867	0.98847
C	-2.62906	2.56436	-2.82340
H	-2.90174	1.50091	-2.66379
C	-1.29262	2.58537	-3.58849
H	-0.48977	2.07007	-3.01664
H	-0.95256	3.61779	-3.82017
H	-1.39684	2.05431	-4.55779
C	-3.75613	3.21553	-3.65575
H	-4.73580	3.14539	-3.14097
H	-3.85310	2.71399	-4.64190
H	-3.54905	4.29141	-3.84300
C	-3.73817	2.63145	2.21644
H	-3.80898	1.53829	2.03559
C	-5.17781	3.18068	2.34654
H	-5.77697	2.97456	1.43687
H	-5.17369	4.28010	2.50967
H	-5.69719	2.71156	3.20892
C	-2.95471	2.83011	3.52611
H	-1.92261	2.43269	3.43777
H	-3.45410	2.28467	4.35385
H	-2.89947	3.89774	3.83033
Sn	1.00887	-0.00035	-1.00004
Na	-0.00002	2.94693	0.00145
C	3.00148	-0.00024	0.17671
C	3.72435	-1.21506	0.40972
C	5.08197	-1.20691	0.80683
H	5.60092	-2.16616	0.97326
C	5.77163	-0.00033	0.99687
H	6.83077	-0.00035	1.29933
C	5.08195	1.20628	0.80711
H	5.60091	2.16549	0.97372
C	3.72430	1.21453	0.41006
C	3.07471	2.56882	0.27496
C	2.54451	3.21205	1.43727
C	1.97627	4.50199	1.30770
H	1.57714	5.01269	2.19892
C	1.92765	5.15071	0.06133
H	1.49029	6.15873	-0.02088
C	2.46701	4.52153	-1.07340
H	2.44533	5.04559	-2.04169
C	3.05413	3.23813	-0.98895
C	2.63026	2.56444	2.82319
H	2.90278	1.50094	2.66365
C	3.75754	3.21556	3.65528
H	4.73709	3.14529	3.14028
H	3.55061	4.29148	3.84250
H	3.85468	2.71407	4.64144
C	1.29391	2.58566	3.58846
H	0.49099	2.07015	3.01688
H	1.39825	2.05488	4.55790
H	0.95385	3.61813	3.81989
C	3.73762	2.63058	-2.21709
H	3.80787	1.53737	-2.03632
C	5.17754	3.17901	-2.34745

H	5.77677	2.97249	-1.43792
H	5.69648	2.70967	-3.20997
H	5.17400	4.27844	-2.51051
C	2.95403	2.82976	-3.52662
H	1.92169	2.43299	-3.43814
H	2.89940	3.89742	-3.83081
H	3.45291	2.28402	-4.35447
C	3.07463	-2.56923	0.27409
C	2.54428	-3.21286	1.43610
C	1.97553	-4.50253	1.30590
H	1.57624	-5.01352	2.19687
C	1.92659	-5.15058	0.05919
H	1.48874	-6.15834	-0.02355
C	2.46624	-4.52107	-1.07523
H	2.44438	-5.04464	-2.04377
C	3.05387	-3.23794	-0.99013
C	2.63041	-2.56605	2.82237
H	2.90394	-1.50272	2.66343
C	1.29391	-2.58643	3.58739
H	0.49160	-2.06981	3.01597
H	0.95280	-3.61873	3.81810
H	1.39857	-2.05636	4.55718
C	3.75698	-3.21860	3.65429
H	4.73666	-3.14898	3.13945
H	3.85446	-2.71767	4.64071
H	3.54907	-4.29442	3.84099
C	3.73777	-2.63011	-2.21789
H	3.80858	-1.53703	-2.03654
C	5.17742	-3.17924	-2.34837
H	5.77665	-2.97351	-1.43867
H	5.17332	-4.27858	-2.51201
H	5.69670	-2.70971	-3.21059
C	2.95419	-2.82818	-3.52758
H	1.92209	-2.43084	-3.43894
H	3.45348	-2.28232	-4.35511
H	2.89897	-3.89567	-3.83230

Method F

SCF (M062X/BS4) Energy = -3081.28963295

Enthalpy 0K = -3080.104729

Enthalpy 298K = -3080.033356

Free Energy 298K = -3080.211341

Lowest Frequency = 18.1435 cm^{-1}

Second Frequency = 20.6826 cm^{-1}

SCF (M062X-D3) Energy = -3081.31096349

SCF (SMD=C_6H_6) Energy = -3081.32777404

SCF (BS5) Energy = -3083.96716642

Sn	-0.89299	0.05498	1.05620
Na	0.00224	-2.85754	0.00227
C	-2.88720	0.00395	-0.09053
C	-3.57557	1.18037	-0.47694
C	-4.92800	1.15863	-0.84538
H	-5.42361	2.09527	-1.11492
C	-5.64046	-0.03614	-0.87780
H	-6.69496	-0.04743	-1.15666
C	-4.97491	-1.21828	-0.56815
H	-5.50563	-2.17313	-0.61707
C	-3.62687	-1.20117	-0.18695
C	-2.98112	-2.53359	0.06891
C	-2.49258	-3.28480	-1.02703
C	-1.92278	-4.54533	-0.78684
H	-1.55929	-5.14281	-1.62544
C	-1.79747	-5.03857	0.51170
H	-1.34017	-6.01474	0.68480
C	-2.26521	-4.28520	1.58730
H	-2.16519	-4.67867	2.59989
C	-2.87753	-3.04070	1.38535
C	-2.57862	-2.76285	-2.45550
H	-2.72984	-1.67534	-2.39390
C	-3.78556	-3.37317	-3.17674
H	-4.72235	-3.14407	-2.65123
H	-3.68597	-4.46852	-3.23655
H	-3.86142	-2.98031	-4.20137
C	-1.29494	-3.00171	-3.25502
H	-0.41607	-2.57070	-2.74848
H	-1.36915	-2.51265	-4.23739
H	-1.11050	-4.07208	-3.43690
C	-3.49565	-2.29467	2.55798
H	-3.51601	-1.22777	2.29217
C	-4.94430	-2.76240	2.75156
H	-5.54005	-2.60073	1.84257
H	-5.41864	-2.20936	3.57570
H	-4.97451	-3.83585	2.99606
C	-2.70318	-2.42832	3.85717
H	-1.65643	-2.12388	3.71250
H	-2.72887	-3.45512	4.25411
H	-3.13959	-1.77305	4.62460
C	-2.88015	2.50999	-0.56701
C	-2.31862	2.91094	-1.80433
C	-1.70552	4.16934	-1.89470
H	-1.26903	4.49033	-2.84184
C	-1.64961	5.02229	-0.79199
H	-1.17629	6.00183	-0.88226
C	-2.19428	4.61702	0.42599
H	-2.14018	5.28570	1.28675
C	-2.80988	3.36244	0.55859
C	-2.41582	2.02690	-3.04056
H	-2.51703	0.98965	-2.68714
C	-1.18396	2.08924	-3.94392
H	-0.26616	1.84111	-3.39147
H	-1.06657	3.07682	-4.41678
H	-1.28759	1.35410	-4.75537
C	-3.67314	2.38697	-3.84348
H	-4.58491	2.27770	-3.24266
H	-3.76286	1.73469	-4.72471
H	-3.61515	3.42946	-4.19445
C	-3.43104	2.95711	1.88643
H	-3.45269	1.85731	1.91084
C	-4.87644	3.46325	1.96744
H	-5.48535	3.06227	1.14584
H	-4.90359	4.56290	1.91149
H	-5.33791	3.15664	2.91769
C	-2.62366	3.42878	3.09605
H	-1.56676	3.13361	3.01068
H	-3.02398	2.97242	4.01257
H	-2.67570	4.52102	3.22600
Sn	0.89263	0.05637	-1.05520
Na	-0.00380	2.94968	0.00009
C	2.88733	0.01004	0.09076
C	3.62975	-1.19340	0.18675
C	4.97792	-1.20752	0.56765
H	5.51083	-2.16116	0.61627
C	5.64086	-0.02393	0.87733
H	6.69545	-0.03285	1.15596
C	4.92569	1.16925	0.84519
H	5.41927	2.10699	1.11466

C	3.57313	1.18798	0.47710
C	2.87468	2.51601	0.56717
C	2.31233	2.91582	1.80447
C	1.69622	4.17276	1.89469
H	1.25900	4.49284	2.84181
C	1.63804	5.02532	0.79182
H	1.16217	6.00364	0.88189
C	2.18343	4.62110	-0.42619
H	2.12736	5.28939	-1.28712
C	2.80211	3.36803	-0.55861
C	2.41131	2.03198	3.04069
H	2.51385	0.99488	2.68721
C	3.66830	2.39386	3.84332
H	4.58013	2.28572	3.24240
H	3.60894	3.43633	4.19414
H	3.75902	1.74186	4.72465
C	1.17965	2.09260	3.94443
H	0.26199	1.84369	3.39211
H	1.28429	1.35723	4.75554
H	1.06134	3.07987	4.41772
C	3.42393	2.96386	-1.88650
H	3.44791	1.86410	-1.91080
C	4.86824	3.47306	-1.96779
H	5.47812	3.07351	-1.14622
H	5.33022	3.16730	-2.91806
H	4.89303	4.57278	-1.91200
C	2.61539	3.43370	-3.09606
H	1.55907	3.13648	-3.01045
H	2.66524	4.52602	-3.22622
H	3.01644	2.97796	-4.01256
C	2.98695	-2.52719	-0.06944
C	2.50012	-3.27978	1.02627
C	1.93260	-4.54126	0.78564
H	1.57043	-5.13982	1.62406
C	1.80793	-5.03413	-0.51308
H	1.35227	-6.01100	-0.68655
C	2.27425	-4.27952	-1.58843
H	2.17478	-4.67280	-2.60114
C	2.88428	-3.03400	-1.38607
C	2.58604	-2.75854	2.45502
H	2.73545	-1.67075	2.39405
C	1.30329	-3.00002	3.25523
H	0.42346	-2.56963	2.74984
H	1.12042	-4.07081	3.43619
H	1.37753	-2.51187	4.23805
C	3.79450	-3.36731	3.17506
H	4.73051	-3.13631	2.64899
H	3.87046	-2.97494	4.19987
H	3.69679	-4.46286	3.23426
C	3.50081	-2.28623	-2.55843
H	3.51868	-1.21937	-2.29229
C	4.95057	-2.75054	-2.75204
H	5.54591	-2.58769	-1.84300
H	4.98327	-3.82386	-2.99679
H	5.42364	-2.19622	-3.57604
C	2.70880	-2.42134	-3.85775
H	1.66126	-2.11964	-3.71307
H	3.14365	-1.76469	-4.62489
H	2.73711	-3.44793	-4.25506

Na(0)

Methods A, B and C

SCF (BP86/BS1) Energy = -0.190251935461
Enthalpy 0K = -0.190252
Enthalpy 298K = -0.187891
Free Energy 298K = -0.205335
SCF (BP86-$D3^{BJ}$) Energy = -0.190252
SCF (C_6H_6) Energy = -0.194926
SCF (BS2) Energy = -162.278274773
SCF (BP86-$D3^{BJ}$(CPCM=C_6H_6)/BS3) Energy = -162.627446
SCF (BP86-$D3^{BJ}$(SMD=C_6H_6)/BS3) Energy = -162.626238

Na	0.00000	0.00000	0.00000

Methods D and E

SCF (BP86/BS4) Energy = -162.221825680
Enthalpy 0K = -162.221826
Enthalpy 298K = -162.219465
Free Energy 298K = -162.236909
SCF (BP86-$D3^{BJ}$) Energy = -162.221825680
SCF (PCM=C_6H_6) Energy = -162.226635
SCF (SMD=C_6H_6) Energy = -162.220913
SCF (BS5) Energy = -162.282753971

Na	0.00000	0.00000	0.00000

Method F

SCF (M062X/BS4) Energy = -162.192480
Enthalpy 0K = -162.192480
Enthalpy 298K = -162.190119
Free Energy 298K = -162.207563
SCF (M062X-D3) Energy = -162.192480
SCF (SMD=C_6H_6) Energy = -162.191477
SCF (BS5) Energy = -162.245236

Na	0.00000	0.00000	0.00000

1^K

Methods A, B and C

SCF (BP86/BS1) Energy = -2394.56568685
Enthalpy 0K = -2393.422802
Enthalpy 298K = -2393.347664
Free Energy 298K = -2393.538726
Lowest Frequency = 8.7577 cm^{-1}
Second Frequency = 17.3446 cm^{-1}
SCF (BP86-$D3^{BJ}$) Energy = -2394.95540078
SCF (C_6H_6) Energy = -2394.57459024
SCF (BS2) Energy = -3960.41723208
SCF (BP86-$D3^{BJ}$(CPCM=C_6H_6)/BS3) Energy = -16063.956754
SCF (BP86-$D3^{BJ}$(SMD=C_6H_6)/BS3) Energy = -16063.987946

Sn	-0.29075	0.99269	0.99689
K	-1.94519	-2.18920	1.63141
C	-2.18770	2.16084	0.27784
C	-2.08130	3.43352	-0.37587
C	-3.17599	4.32486	-0.46948
H	-3.03592	5.28623	-0.97910
C	-4.42630	3.99711	0.06690
H	-5.26722	4.69503	-0.00134
C	-4.57860	2.74670	0.67668
H	-5.55531	2.45122	1.07950
C	-3.49391	1.84429	0.77937
C	-3.86210	0.52958	1.42670

C	-4.47621	-0.48736	0.63284
C	-4.99315	-1.63882	1.26916
H	-5.49357	-2.40706	0.66819
C	-4.90451	-1.79959	2.66155
H	-5.33296	-2.68629	3.14332
C	-4.29258	-0.80044	3.43672
H	-4.24576	-0.91802	4.52505
C	-3.77617	0.37299	2.84360
C	-4.64028	-0.33462	-0.88284
H	-4.05073	0.54746	-1.18493
C	-6.11799	-0.06016	-1.24605
H	-6.49065	0.84670	-0.74175
H	-6.76686	-0.90419	-0.95029
H	-6.22779	0.08405	-2.33515
C	-4.09436	-1.54544	-1.66906
H	-3.01357	-1.69148	-1.48586
H	-4.21962	-1.38144	-2.75333
H	-4.62716	-2.48088	-1.41816
C	-3.22538	1.49103	3.73318
H	-2.66048	2.17721	3.07784
C	-4.38893	2.28864	4.36941
H	-5.04794	2.71920	3.59769
H	-3.99645	3.11740	4.98437
H	-5.00393	1.64337	5.02232
C	-2.25001	0.98632	4.81561
H	-1.39254	0.46331	4.35781
H	-2.73814	0.30942	5.54028
H	-1.84995	1.84091	5.38794
C	-0.83054	3.93974	-1.05598
C	-0.69978	3.78588	-2.47051
C	0.37627	4.41721	-3.13520
H	0.46332	4.32769	-4.22439
C	1.31714	5.18258	-2.42735
H	2.13238	5.68610	-2.96003
C	1.18666	5.32722	-1.03606
H	1.90812	5.94533	-0.49043
C	0.11783	4.72701	-0.33432
C	-1.72723	3.00150	-3.29327
H	-2.39175	2.48838	-2.57772
C	-1.08002	1.91774	-4.18151
H	-0.53181	1.17348	-3.57510
H	-0.38687	2.34848	-4.92712
H	-1.85838	1.36999	-4.74038
C	-2.59659	3.96094	-4.13851
H	-3.11760	4.69384	-3.50062
H	-3.35861	3.39569	-4.70312
H	-1.98313	4.52080	-4.86727
C	-0.06256	5.01250	1.15925
H	-0.76671	4.25708	1.55055
C	-0.69989	6.40853	1.35998
H	-1.67297	6.48189	0.84729
H	-0.04435	7.20539	0.96475
H	-0.86406	6.60577	2.43386
C	1.24248	4.88529	1.97020
H	1.67492	3.87561	1.86361
H	1.03473	5.04577	3.04208
H	1.99946	5.63275	1.67051
Sn	0.29075	-0.99269	-0.99689
K	1.94519	2.18920	-1.63141
C	2.18770	-2.16084	-0.27784
C	2.08130	-3.43352	0.37587
C	3.17599	-4.32486	0.46948
H	3.03592	-5.28623	0.97910
C	4.42630	-3.99711	-0.06690

H	5.26722	-4.69503	0.00134
C	4.57860	-2.74670	-0.67668
H	5.55531	-2.45122	-1.07950
C	3.49391	-1.84429	-0.77937
C	3.86210	-0.52958	-1.42670
C	4.47621	0.48736	-0.63284
C	4.99315	1.63882	-1.26916
H	5.49357	2.40706	-0.66819
C	4.90451	1.79959	-2.66155
H	5.33296	2.68629	-3.14332
C	4.29258	0.80044	-3.43672
H	4.24576	0.91802	-4.52505
C	3.77617	-0.37299	-2.84360
C	4.64028	0.33462	0.88284
H	4.05073	-0.54746	1.18493
C	6.11799	0.06016	1.24605
H	6.49065	-0.84670	0.74175
H	6.76686	0.90419	0.95029
H	6.22779	-0.08405	2.33515
C	4.09436	1.54544	1.66906
H	3.01357	1.69148	1.48586
H	4.21962	1.38144	2.75333
H	4.62716	2.48088	1.41816
C	3.22538	-1.49103	-3.73318
H	2.66048	-2.17721	-3.07784
C	4.38893	-2.28864	-4.36941
H	5.04794	-2.71920	-3.59769
H	3.99645	-3.11740	-4.98437
H	5.00393	-1.64337	-5.02232
C	2.25001	-0.98632	-4.81561
H	1.39254	-0.46331	-4.35781
H	2.73814	-0.30942	-5.54028
H	1.84995	-1.84091	-5.38794
C	0.83054	-3.93974	1.05598
C	0.69978	-3.78588	2.47051
C	-0.37627	-4.41721	3.13520
H	-0.46332	-4.32769	4.22439
C	-1.31714	-5.18258	2.42735
H	-2.13238	-5.68610	2.96003
C	-1.18666	-5.32722	1.03606
H	-1.90812	-5.94533	0.49043
C	-0.11783	-4.72701	0.33432
C	1.72723	-3.00150	3.29327
H	2.39175	-2.48838	2.57772
C	1.08002	-1.91774	4.18151
H	0.53181	-1.17348	3.57510
H	0.38687	-2.34848	4.92712
H	1.85838	-1.36999	4.74038
C	2.59659	-3.96094	4.13851
H	3.11760	-4.69384	3.50062
H	3.35861	-3.39569	4.70312
H	1.98313	-4.52080	4.86727
C	0.06256	-5.01250	-1.15925
H	0.76671	-4.25708	-1.55055
C	0.69989	-6.40853	-1.35998
H	1.67297	-6.48189	-0.84729
H	0.04435	-7.20539	-0.96475
H	0.86406	-6.60577	-2.43386
C	-1.24248	-4.88529	-1.97020
H	-1.67492	-3.87561	-1.86361
H	-1.03473	-5.04577	-3.04208
H	-1.99946	-5.63275	-1.67051

Methods D and E

SCF (BP86/BS4) Energy = -3958.00655322
Enthalpy 0K = -3956.868854
Enthalpy 298K = -3956.793867
Free Energy 298K = -3956.983944
Lowest Frequency = 5.9755 cm^{-1}
Second Frequency = 17.5930 cm^{-1}
SCF (BP86-$D3^{BJ}$) Energy = -3958.39737173
SCF (PCM=C_6H_6) Energy = -3958.01416074
SCF (SMD=C_6H_6) Energy = -3958.04366663
SCF (BS5) Energy = -3960.65928373

Sn	-0.29667	0.98467	0.97974
K	-1.92603	-2.16491	1.61411
C	-2.18236	2.15146	0.27574
C	-2.07037	3.42618	-0.37430
C	-3.16579	4.31912	-0.46360
H	-3.02798	5.28685	-0.97542
C	-4.41688	3.99052	0.07558
H	-5.26271	4.69285	0.00831
C	-4.56933	2.73897	0.68755
H	-5.55184	2.44558	1.09503
C	-3.48549	1.83295	0.78582
C	-3.84957	0.51733	1.43300
C	-4.46788	-0.49992	0.63925
C	-4.98394	-1.65313	1.27693
H	-5.48580	-2.42810	0.67431
C	-4.88615	-1.81783	2.66904
H	-5.30922	-2.71342	3.15317
C	-4.26819	-0.82058	3.44422
H	-4.20832	-0.94684	4.53719
C	-3.75291	0.35544	2.85122
C	-4.63073	-0.34875	-0.87685
H	-4.06142	0.55647	-1.17152
C	-6.10900	-0.11283	-1.25474
H	-6.52017	0.77992	-0.74091
H	-6.74405	-0.98318	-0.98147
H	-6.21185	0.04688	-2.34899
C	-4.03425	-1.53671	-1.65693
H	-2.94601	-1.65025	-1.45459
H	-4.14287	-1.37591	-2.75007
H	-4.54143	-2.49673	-1.41777
C	-3.19055	1.46791	3.74079
H	-2.61562	2.14828	3.07778
C	-4.33906	2.28494	4.37505
H	-5.00326	2.72056	3.60161
H	-3.93264	3.12074	4.98316
H	-4.96304	1.65238	5.04291
C	-2.21817	0.95372	4.81831
H	-1.37433	0.39704	4.35937
H	-2.71709	0.29615	5.56289
H	-1.78388	1.80758	5.37915
C	-0.81747	3.92968	-1.05164
C	-0.68390	3.77678	-2.46789
C	0.39598	4.40762	-3.13051
H	0.48997	4.31526	-4.22523
C	1.34010	5.16704	-2.41901
H	2.16628	5.66616	-2.95168
C	1.20899	5.30925	-1.02620
H	1.94141	5.92088	-0.47534
C	0.13627	4.71094	-0.32543
C	-1.70624	2.98784	-3.29294
H	-2.40196	2.51205	-2.57203
C	-1.05486	1.85644	-4.11254
H	-0.53690	1.12420	-3.45410
H	-0.32496	2.24150	-4.85771
H	-1.82674	1.29156	-4.67626
C	-2.53861	3.92731	-4.19174
H	-3.05488	4.70613	-3.59412
H	-3.31250	3.35427	-4.74504
H	-1.90295	4.44378	-4.94318
C	-0.04135	4.99054	1.16975
H	-0.74737	4.22597	1.55718
C	-0.68457	6.37967	1.38429
H	-1.66134	6.46000	0.86608
H	-0.02805	7.19094	1.00178
H	-0.85774	6.56563	2.46548
C	1.26316	4.85681	1.97636
H	1.70987	3.84901	1.84666
H	1.05743	4.99012	3.05900
H	2.01882	5.62123	1.69301
Sn	0.29667	-0.98467	-0.97974
K	1.92603	2.16491	-1.61411
C	2.18236	-2.15146	-0.27574
C	2.07037	-3.42618	0.37430
C	3.16579	-4.31912	0.46360
H	3.02798	-5.28685	0.97542
C	4.41688	-3.99052	-0.07558
H	5.26271	-4.69285	-0.00831
C	4.56933	-2.73897	-0.68755
H	5.55184	-2.44558	-1.09503
C	3.48549	-1.83295	-0.78582
C	3.84957	-0.51733	-1.43300
C	4.46788	0.49992	-0.63925
C	4.98394	1.65313	-1.27693
H	5.48580	2.42810	-0.67431
C	4.88615	1.81783	-2.66904
H	5.30922	2.71342	-3.15317
C	4.26819	0.82058	-3.44422
H	4.20832	0.94684	-4.53719
C	3.75291	-0.35544	-2.85122
C	4.63073	0.34875	0.87685
H	4.06142	-0.55647	1.17152
C	6.10900	0.11283	1.25474
H	6.52017	-0.77992	0.74091
H	6.74405	0.98318	0.98147
H	6.21185	-0.04688	2.34899
C	4.03425	1.53671	1.65693
H	2.94601	1.65025	1.45459
H	4.14287	1.37591	2.75007
H	4.54143	2.49673	1.41777
C	3.19055	-1.46791	-3.74079
H	2.61562	-2.14828	-3.07778
C	4.33906	-2.28494	-4.37505
H	5.00326	-2.72056	-3.60161
H	3.93264	-3.12074	-4.98316
H	4.96304	-1.65238	-5.04291
C	2.21817	-0.95372	-4.81831
H	1.37433	-0.39704	-4.35937
H	2.71709	-0.29615	-5.56289
H	1.78388	-1.80758	-5.37915
C	0.81747	-3.92968	1.05164
C	0.68390	-3.77678	2.46789
C	-0.39598	-4.40762	3.13051
H	-0.48997	-4.31526	4.22523
C	-1.34010	-5.16704	2.41901
H	-2.16628	-5.66616	2.95168
C	-1.20899	-5.30925	1.02620
H	-1.94141	-5.92088	0.47534

C	-0.13627	-4.71094	0.32543
C	1.70624	-2.98784	3.29294
H	2.40196	-2.51205	2.57203
C	1.05486	-1.85644	4.11254
H	0.53690	-1.12420	3.45410
H	0.32496	-2.24150	4.85771
H	1.82674	-1.29156	4.67626
C	2.53861	-3.92731	4.19174
H	3.05488	-4.70613	3.59412
H	3.31250	-3.35427	4.74504
H	1.90295	-4.44378	4.94318
C	0.04135	-4.99054	-1.16975
H	0.74737	-4.22597	-1.55718
C	0.68457	-6.37967	-1.38429
H	1.66134	-6.46000	-0.86608
H	0.02805	-7.19094	-1.00178
H	0.85774	-6.56563	-2.46548
C	-1.26316	-4.85681	-1.97636
H	-1.70987	-3.84901	-1.84666
H	-1.05743	-4.99012	-3.05900
H	-2.01882	-5.62123	-1.69301

Method F

SCF (M062X/BS4) Energy = -3956.54062489

Enthalpy 0K = -3955.356939

Enthalpy 298K = -3955.284986

Free Energy 298K = -3955.467259

Lowest Frequency = 4.9049 cm^{-1}

Second Frequency = 16.3211 cm^{-1}

SCF (M062X-D3) Energy = -3956.56054713

SCF (SMD=C_6H_6) Energy = -3956.57941363

SCF (BS5) Energy = -3959.27018091

Sn	-0.21676	0.93466	1.00035
K	-1.93158	-2.17368	1.60153
C	-2.10206	2.09091	0.29105
C	-2.00727	3.33637	-0.38206
C	-3.10728	4.19400	-0.53660
H	-2.97010	5.14357	-1.06175
C	-4.35758	3.85354	-0.03502
H	-5.20915	4.52569	-0.14883
C	-4.49570	2.62828	0.60581
H	-5.47084	2.32339	0.99611
C	-3.39995	1.76565	0.76290
C	-3.75578	0.46854	1.43518
C	-4.38732	-0.54332	0.67482
C	-4.91336	-1.66480	1.33413
H	-5.43224	-2.43386	0.75715
C	-4.78895	-1.80831	2.71483
H	-5.21246	-2.68031	3.21705
C	-4.12935	-0.82551	3.45427
H	-4.03699	-0.94019	4.53585
C	-3.61706	0.32349	2.83590
C	-4.53895	-0.43280	-0.83635
H	-3.92701	0.41856	-1.16607
C	-5.99540	-0.14641	-1.21545
H	-6.35239	0.78026	-0.74482
H	-6.65418	-0.96859	-0.89386
H	-6.09532	-0.03859	-2.30551
C	-4.01222	-1.67586	-1.55776
H	-2.94292	-1.83370	-1.34337
H	-4.10324	-1.55033	-2.64669
H	-4.57299	-2.58368	-1.28345
C	-3.01146	1.43362	3.68096
H	-2.38145	2.04269	3.01657
C	-4.12527	2.33046	4.23547
H	-4.72733	2.76270	3.42414
H	-3.69627	3.15632	4.82202
H	-4.79576	1.75479	4.89306
C	-2.11694	0.91346	4.80571
H	-1.33565	0.24644	4.41228
H	-2.69080	0.37671	5.57728
H	-1.61344	1.75660	5.30006
C	-0.75218	3.85284	-1.02908
C	-0.60341	3.72731	-2.43004
C	0.46219	4.38369	-3.06524
H	0.56450	4.31749	-4.15105
C	1.38812	5.12299	-2.33166
H	2.20688	5.63692	-2.83903
C	1.25610	5.21364	-0.94514
H	1.98003	5.79880	-0.37512
C	0.18790	4.59814	-0.27818
C	-1.58456	2.92370	-3.27292
H	-2.25238	2.39064	-2.58208
C	-0.87516	1.86698	-4.12376
H	-0.33276	1.14678	-3.48980
H	-0.17080	2.31796	-4.84122
H	-1.60978	1.28956	-4.70414
C	-2.44222	3.85164	-4.13894
H	-2.99295	4.57380	-3.51997
H	-3.17275	3.26968	-4.71982
H	-1.81811	4.41719	-4.84881
C	-0.00538	4.82271	1.21369
H	-0.64685	4.01074	1.58567
C	-0.73682	6.15092	1.44551
H	-1.70892	6.16323	0.93322
H	-0.13769	6.99471	1.06813
H	-0.91294	6.30906	2.51981
C	1.30018	4.77225	2.00633
H	1.83842	3.83114	1.82088
H	1.08289	4.82243	3.08293
H	1.96374	5.61790	1.76707
Sn	0.21676	-0.93466	-1.00035
K	1.93158	2.17368	-1.60153
C	2.10206	-2.09091	-0.29105
C	2.00727	-3.33637	0.38206
C	3.10728	-4.19400	0.53660
H	2.97010	-5.14357	1.06175
C	4.35758	-3.85354	0.03502
H	5.20915	-4.52569	0.14883
C	4.49570	-2.62828	-0.60581
H	5.47084	-2.32339	-0.99611
C	3.39995	-1.76565	-0.76290
C	3.75578	-0.46854	-1.43518
C	4.38732	0.54332	-0.67482
C	4.91336	1.66480	-1.33413
H	5.43224	2.43386	-0.75715
C	4.78895	1.80831	-2.71483
H	5.21246	2.68031	-3.21705
C	4.12935	0.82551	-3.45427
H	4.03699	0.94019	-4.53585
C	3.61706	-0.32349	-2.83590
C	4.53895	0.43280	0.83635
H	3.92701	-0.41856	1.16607
C	5.99540	0.14641	1.21545
H	6.35239	-0.78026	0.74482
H	6.65418	0.96859	0.89386

H	6.09532	0.03859	2.30551
C	4.01222	1.67586	1.55776
H	2.94292	1.83370	1.34337
H	4.10324	1.55033	2.64669
H	4.57299	2.58368	1.28345
C	3.01146	-1.43362	-3.68096
H	2.38145	-2.04269	-3.01657
C	4.12527	-2.33046	-4.23547
H	4.72733	-2.76270	-3.42414
H	3.69627	-3.15632	-4.82202
H	4.79576	-1.75479	-4.89306
C	2.11694	-0.91346	-4.80571
H	1.33565	-0.24644	-4.41228
H	2.69080	-0.37671	-5.57728
H	1.61344	-1.75660	-5.30006
C	0.75218	-3.85284	1.02908
C	0.60341	-3.72731	2.43004
C	-0.46219	-4.38369	3.06524
H	-0.56450	-4.31749	4.15105
C	-1.38812	-5.12299	2.33166
H	-2.20688	-5.63692	2.83903
C	-1.25610	-5.21364	0.94514
H	-1.98003	-5.79880	0.37512
C	-0.18790	-4.59814	0.27818
C	1.58456	-2.92370	3.27292
H	2.25238	-2.39064	2.58208
C	0.87516	-1.86698	4.12376
H	0.33276	-1.14678	3.48980
H	0.17080	-2.31796	4.84122
H	1.60978	-1.28956	4.70414
C	2.44222	-3.85164	4.13894
H	2.99295	-4.57380	3.51997
H	3.17275	-3.26968	4.71982
H	1.81811	-4.41719	4.84881
C	0.00538	-4.82271	-1.21369
H	0.64685	-4.01074	-1.58567
C	0.73682	-6.15092	-1.44551
H	1.70892	-6.16323	-0.93322
H	0.13769	-6.99471	-1.06813
H	0.91294	-6.30906	-2.51981
C	-1.30018	-4.77225	-2.00633
H	-1.83842	-3.83114	-1.82088
H	-1.08289	-4.82243	-3.08293
H	-1.96374	-5.61790	-1.76707

K(0)

Methods A, B and C

SCF (BP86/BS1) Energy = -28.3228762473
Enthalpy 0K = -28.322876
Enthalpy 298K = -28.320516
Free Energy 298K = -28.338707
SCF (BP86-D3^{BJ}) Energy = -28.32287625
SCF (C_6H_6) Energy = -28.3255799
SCF (BS2) Energy = -599.9635241
SCF (BP86-D3^{BJ}(CPCM=C_6H_6)/BS3) Energy = -603.637299
SCF (BP86-D3^{BJ}(SMD=C_6H_6)/BS3) Energy = -603.635585

K	0.00000	0.00000	0.00000

Methods D and E

SCF (BP86/BS4) Energy = -599.883546653
Enthalpy 0K = -599.883547
Enthalpy 298K = -599.881186
Free Energy 298K = -599.899377
SCF (BP86-D3^{BJ}) Energy = -599.883546653
SCF (PCM=C_6H_6) Energy = -599.886566
SCF (SMD=C_6H_6) Energy = -599.881959
SCF (BS5) Energy = -599.961730916

K	0.00000	0.00000	0.00000

Method F

SCF (M062X/BS4) Energy = -599.822764
Enthalpy 0K = -599.822764
Enthalpy 298K = -599.820403
Free Energy 298K = -599.838594
SCF (M062X-D3) Energy = -599.822764
SCF (SMD=C_6H_6) Energy = -599.821168
SCF (BS5) Energy = -599.898396

K	0.00000	0.00000	0.00000

1^{Rb}

Methods A, B and C

SCF (BP86/BS1) Energy = -2386.14957720
Enthalpy 0K = -2385.007245
Enthalpy 298K = -2384.931755
Free Energy 298K = -2385.125235
Lowest Frequency = 7.2194 cm^{-1}
Second Frequency = 15.9419 cm^{-1}
SCF (BP86-D3^{BJ}) Energy = -2386.53897568
SCF (C_6H_6) Energy = -2386.15883899
SCF (BS2) Energy = -2808.71546832
SCF (BP86-D3^{BJ}(CPCM=C_6H_6)/BS3) Energy = -20865.318358
SCF (BP86-D3^{BJ}(SMD=C_6H_6)/BS3) Energy = -20865.348855

Sn	-0.32651	1.00880	0.98095
Rb	-2.03545	-2.28343	1.71318
C	-2.23204	2.18991	0.26542
C	-2.12523	3.46930	-0.37688
C	-3.21495	4.36967	-0.45202
H	-3.07116	5.33371	-0.95533
C	-4.46272	4.04842	0.09218
H	-5.29920	4.75286	0.03742
C	-4.61631	2.79449	0.69255
H	-5.59074	2.50115	1.10215
C	-3.53720	1.88241	0.77763
C	-3.93494	0.57436	1.42203
C	-4.57631	-0.42235	0.62430
C	-5.16626	-1.53813	1.25968
H	-5.68939	-2.28762	0.65405
C	-5.12404	-1.68433	2.65535
H	-5.61231	-2.54028	3.13604
C	-4.47823	-0.71015	3.43413
H	-4.46436	-0.81618	4.52482
C	-3.88667	0.42725	2.84145
C	-4.69219	-0.28258	-0.89707
H	-4.08502	0.59066	-1.18935
C	-6.15391	0.00100	-1.31281
H	-6.53477	0.91521	-0.82819
H	-6.82124	-0.83424	-1.03369
H	-6.22520	0.13764	-2.40610
C	-4.12941	-1.50721	-1.65002
H	-3.05282	-1.64949	-1.44099

H	-4.22982	-1.36365	-2.73975
H	-4.66853	-2.43765	-1.39332
C	-3.29023	1.51957	3.73367
H	-2.70454	2.18857	3.07875
C	-4.41601	2.35574	4.38718
H	-5.06574	2.81582	3.62466
H	-3.98671	3.16508	5.00344
H	-5.04897	1.73036	5.04231
C	-2.32420	0.96786	4.80207
H	-1.48425	0.42916	4.32949
H	-2.82843	0.29225	5.51690
H	-1.89494	1.79943	5.38703
C	-0.89004	3.99600	-1.07076
C	-0.78633	3.86403	-2.48961
C	0.23931	4.55574	-3.17254
H	0.30162	4.48393	-4.26491
C	1.15838	5.35907	-2.47911
H	1.93131	5.91203	-3.02588
C	1.06100	5.47475	-1.08281
H	1.76586	6.11996	-0.54644
C	0.04256	4.81281	-0.36229
C	-1.79350	3.03763	-3.29620
H	-2.43544	2.51059	-2.57032
C	-1.11342	1.96489	-4.17385
H	-0.54855	1.23953	-3.55980
H	-0.42903	2.40970	-4.91955
H	-1.87333	1.39122	-4.73206
C	-2.70152	3.95486	-4.14710
H	-3.24156	4.67788	-3.51371
H	-3.44874	3.35637	-4.69712
H	-2.11500	4.52581	-4.88929
C	-0.10131	5.05971	1.14210
H	-0.78867	4.28861	1.53281
C	-0.74117	6.44471	1.39967
H	-1.72845	6.52463	0.91580
H	-0.10277	7.25769	1.00923
H	-0.87706	6.61091	2.48274
C	1.22832	4.91766	1.91032
H	1.65555	3.90930	1.77162
H	1.05510	5.05703	2.99126
H	1.97613	5.67020	1.60023
Sn	0.32651	-1.00880	-0.98095
Rb	2.03545	2.28343	-1.71318
C	2.23204	-2.18991	-0.26542
C	2.12523	-3.46930	0.37688
C	3.21495	-4.36967	0.45202
H	3.07116	-5.33371	0.95533
C	4.46272	-4.04842	-0.09218
H	5.29920	-4.75286	-0.03742
C	4.61631	-2.79449	-0.69255
H	5.59074	-2.50115	-1.10215
C	3.53720	-1.88241	-0.77763
C	3.93494	-0.57436	-1.42203
C	4.57631	0.42235	-0.62430
C	5.16626	1.53813	-1.25968
H	5.68939	2.28762	-0.65405
C	5.12404	1.68433	-2.65535
H	5.61231	2.54028	-3.13604
C	4.47823	0.71015	-3.43413
H	4.46436	0.81618	-4.52482
C	3.88667	-0.42725	-2.84145
C	4.69219	0.28258	0.89707
H	4.08502	-0.59066	1.18935
C	6.15391	-0.00100	1.31281
H	6.53477	-0.91521	0.82819
H	6.82124	0.83424	1.03369
H	6.22520	-0.13764	2.40610
C	4.12941	1.50721	1.65002
H	3.05282	1.64949	1.44099
H	4.22982	1.36365	2.73975
H	4.66853	2.43765	1.39332
C	3.29023	-1.51957	-3.73367
H	2.70454	-2.18857	-3.07875
C	4.41601	-2.35574	-4.38718
H	5.06574	-2.81582	-3.62466
H	3.98671	-3.16508	-5.00344
H	5.04897	-1.73036	-5.04231
C	2.32420	-0.96786	-4.80207
H	1.48425	-0.42916	-4.32949
H	2.82843	-0.29225	-5.51690
H	1.89494	-1.79943	-5.38703
C	0.89004	-3.99600	1.07076
C	0.78633	-3.86403	2.48961
C	-0.23931	-4.55574	3.17254
H	-0.30162	-4.48393	4.26491
C	-1.15838	-5.35907	2.47911
H	-1.93131	-5.91203	3.02588
C	-1.06100	-5.47475	1.08281
H	-1.76586	-6.11996	0.54644
C	-0.04256	-4.81281	0.36229
C	1.79350	-3.03763	3.29620
H	2.43544	-2.51059	2.57032
C	1.11342	-1.96489	4.17385
H	0.54855	-1.23953	3.55980
H	0.42903	-2.40970	4.91955
H	1.87333	-1.39122	4.73206
C	2.70152	-3.95486	4.14710
H	3.24156	-4.67788	3.51371
H	3.44874	-3.35637	4.69712
H	2.11500	-4.52581	4.88929
C	0.10131	-5.05971	-1.14210
H	0.78867	-4.28861	-1.53281
C	0.74117	-6.44471	-1.39967
H	1.72845	-6.52463	-0.91580
H	0.10277	-7.25769	-1.00923
H	0.87706	-6.61091	-2.48274
C	-1.22832	-4.91766	-1.91032
H	-1.65555	-3.90930	-1.77162
H	-1.05510	-5.05703	-2.99126
H	-1.97613	-5.67020	-1.60023

Methods D and E

SCF (BP86/BS4) Energy = -2806.46228593
Enthalpy 0K = -2805.325411
Enthalpy 298K = -2805.249906
Free Energy 298K = -2805.443704
Lowest Frequency = 5.9626 cm^{-1}
Second Frequency = 14.9512 cm^{-1}
SCF (BP86-D3^{BJ}) Energy = -2806.85162854
SCF (PCM=C_6H_6) Energy = -2806.47064535
SCF (SMD=C_6H_6) Energy = -2806.50719756
SCF (BS5) Energy = -2808.96133483

Sn	-0.33788	1.00487	0.96164
Rb	-2.01516	-2.26346	1.70095
C	-2.23400	2.18698	0.25931
C	-2.12162	3.47018	-0.37640
C	-3.20981	4.37549	-0.43882

H	-3.06732	5.34779	-0.94053
C	-4.45717	4.05529	0.11158
H	-5.29653	4.76691	0.06431
C	-4.61215	2.79749	0.70784
H	-5.59187	2.50679	1.12376
C	-3.53613	1.87863	0.78119
C	-3.93542	0.56885	1.42040
C	-4.57623	-0.42636	0.61639
C	-5.17356	-1.54233	1.24832
H	-5.69844	-2.29568	0.63748
C	-5.13587	-1.69223	2.64508
H	-5.62962	-2.55384	3.12456
C	-4.48914	-0.72122	3.42981
H	-4.47648	-0.83468	4.52619
C	-3.89015	0.41700	2.84192
C	-4.68408	-0.28174	-0.90550
H	-4.07387	0.60055	-1.18743
C	-6.13947	0.00301	-1.33495
H	-6.53556	0.91265	-0.83926
H	-6.81409	-0.84286	-1.08004
H	-6.19947	0.16009	-2.43277
C	-4.10461	-1.49569	-1.65884
H	-3.02607	-1.64191	-1.42908
H	-4.17927	-1.34146	-2.75590
H	-4.64959	-2.43649	-1.42543
C	-3.29009	1.50363	3.73938
H	-2.70012	2.17460	3.07999
C	-4.40613	2.34748	4.39530
H	-5.06625	2.80939	3.63365
H	-3.96832	3.16551	5.00577
H	-5.04118	1.72852	5.06555
C	-2.32259	0.94583	4.80056
H	-1.48645	0.39181	4.32378
H	-2.82973	0.27527	5.52798
H	-1.87367	1.77741	5.38317
C	-0.88918	3.99570	-1.07539
C	-0.79136	3.86183	-2.49670
C	0.23044	4.55692	-3.18543
H	0.29133	4.48415	-4.28421
C	1.15273	5.36217	-2.49584
H	1.92786	5.91768	-3.04959
C	1.06232	5.47876	-1.09757
H	1.77658	6.12602	-0.56300
C	0.04819	4.81406	-0.37004
C	-1.80059	3.03087	-3.29679
H	-2.45014	2.51767	-2.55852
C	-1.12414	1.93660	-4.14665
H	-0.55484	1.22157	-3.51301
H	-0.43682	2.36095	-4.91101
H	-1.88755	1.34224	-4.69149
C	-2.70479	3.93450	-4.16236
H	-3.23793	4.68393	-3.54255
H	-3.46798	3.32843	-4.69496
H	-2.11895	4.48502	-4.93002
C	-0.08675	5.05980	1.13576
H	-0.77663	4.28266	1.52740
C	-0.72803	6.43973	1.40553
H	-1.71965	6.52858	0.91769
H	-0.08689	7.26481	1.02633
H	-0.87082	6.59649	2.49575
C	1.24421	4.91116	1.89661
H	1.67935	3.90115	1.74363
H	1.07652	5.03406	2.98710
H	1.99418	5.67423	1.59456
Sn	0.33788	-1.00487	-0.96164
Rb	2.01516	2.26346	-1.70095
C	2.23400	-2.18698	-0.25931
C	2.12162	-3.47018	0.37640
C	3.20981	-4.37549	0.43882
H	3.06732	-5.34779	0.94053
C	4.45717	-4.05529	-0.11158
H	5.29653	-4.76691	-0.06431
C	4.61215	-2.79749	-0.70784
H	5.59187	-2.50679	-1.12376
C	3.53613	-1.87863	-0.78119
C	3.93542	-0.56885	-1.42040
C	4.57623	0.42636	-0.61639
C	5.17356	1.54233	-1.24832
H	5.69844	2.29568	-0.63748
C	5.13587	1.69223	-2.64508
H	5.62962	2.55384	-3.12456
C	4.48914	0.72122	-3.42981
H	4.47648	0.83468	-4.52619
C	3.89015	-0.41700	-2.84192
C	4.68408	0.28174	0.90550
H	4.07387	-0.60055	1.18743
C	6.13947	-0.00301	1.33495
H	6.53556	-0.91265	0.83926
H	6.81409	0.84286	1.08004
H	6.19947	-0.16009	2.43277
C	4.10461	1.49569	1.65884
H	3.02607	1.64191	1.42908
H	4.17927	1.34146	2.75590
H	4.64959	2.43649	1.42543
C	3.29009	-1.50363	-3.73938
H	2.70012	-2.17460	-3.07999
C	4.40613	-2.34748	-4.39530
H	5.06625	-2.80939	-3.63365
H	3.96832	-3.16551	-5.00577
H	5.04118	-1.72852	-5.06555
C	2.32259	-0.94583	-4.80056
H	1.48645	-0.39181	-4.32378
H	2.82973	-0.27527	-5.52798
H	1.87367	-1.77741	-5.38317
C	0.88918	-3.99570	1.07539
C	0.79136	-3.86183	2.49670
C	-0.23044	-4.55692	3.18543
H	-0.29133	-4.48415	4.28421
C	-1.15273	-5.36217	2.49584
H	-1.92786	-5.91768	3.04959
C	-1.06232	-5.47876	1.09757
H	-1.77658	-6.12602	0.56300
C	-0.04819	-4.81406	0.37004
C	1.80059	-3.03087	3.29679
H	2.45014	-2.51767	2.55852
C	1.12414	-1.93660	4.14665
H	0.55484	-1.22157	3.51301
H	0.43682	-2.36095	4.91101
H	1.88755	-1.34224	4.69149
C	2.70479	-3.93450	4.16236
H	3.23793	-4.68393	3.54255
H	3.46798	-3.32843	4.69496
H	2.11895	-4.48502	4.93002
C	0.08675	-5.05980	-1.13576
H	0.77663	-4.28266	-1.52740
C	0.72803	-6.43973	-1.40553
H	1.71965	-6.52858	-0.91769
H	0.08689	-7.26481	-1.02633

H	0.87082	-6.59649	-2.49575
C	-1.24421	-4.91116	-1.89661
H	-1.67935	-3.90115	-1.74363
H	-1.07652	-5.03406	-2.98710
H	-1.99418	-5.67423	-1.59456

Method F

SCF (M062X/BS4) Energy = -2804.92312857
Enthalpy 0K = -2803.740885
Enthalpy 298K = -2803.668221
Free Energy 298K = -2803.854671
Lowest Frequency = 4.4804 cm^{-1}
Second Frequency = 9.4927 cm^{-1}
SCF (M062X-D3) Energy = -2804.94250839
SCF (SMD=C_6H_6) Energy = -2804.96891046
SCF (BS5) Energy = -2807.50539167

Sn	-0.25728	0.95512	0.98214
Rb	-2.03706	-2.28360	1.68400
C	-2.15059	2.12164	0.27611
C	-2.05453	3.37581	-0.38183
C	-3.14857	4.24588	-0.51480
H	-3.00636	5.19931	-1.03163
C	-4.39697	3.91390	-0.00516
H	-5.24345	4.59488	-0.10328
C	-4.53728	2.68372	0.62370
H	-5.51055	2.38222	1.02131
C	-3.44768	1.80851	0.76010
C	-3.83828	0.51953	1.42907
C	-4.50314	-0.46673	0.66389
C	-5.11720	-1.54244	1.32188
H	-5.66572	-2.28723	0.73972
C	-5.05109	-1.66615	2.70856
H	-5.55187	-2.49639	3.21093
C	-4.35148	-0.71588	3.45332
H	-4.30487	-0.81426	4.53989
C	-3.74543	0.38591	2.83399
C	-4.59611	-0.37465	-0.85320
H	-3.97115	0.47171	-1.17042
C	-6.03445	-0.09319	-1.29767
H	-6.41061	0.83739	-0.84976
H	-6.70837	-0.91210	-1.00010
H	-6.08659	0.00650	-2.39182
C	-4.03729	-1.62893	-1.53097
H	-2.97314	-1.77279	-1.28219
H	-4.09473	-1.52943	-2.62508
H	-4.60053	-2.53436	-1.25182
C	-3.08690	1.46312	3.68227
H	-2.43942	2.05284	3.01706
C	-4.15356	2.39992	4.26169
H	-4.74833	2.86396	3.46248
H	-3.68217	3.20202	4.84869
H	-4.83874	1.84779	4.92446
C	-2.19940	0.89009	4.78757
H	-1.44120	0.20955	4.37160
H	-2.78323	0.35330	5.55170
H	-1.66434	1.70509	5.29595
C	-0.81697	3.91570	-1.04377
C	-0.69974	3.81269	-2.44933
C	0.30118	4.54326	-3.10644
H	0.37264	4.49862	-4.19616
C	1.19602	5.33654	-2.39055
H	1.95772	5.91678	-2.91556
C	1.10460	5.39642	-0.99942
H	1.80408	6.02333	-0.44251
C	0.10157	4.70158	-0.30960
C	-1.65078	2.95293	-3.27096
H	-2.28661	2.39963	-2.56603
C	-0.89538	1.91657	-4.10778
H	-0.32135	1.23019	-3.46403
H	-0.21314	2.38996	-4.83272
H	-1.60296	1.29643	-4.67789
C	-2.55966	3.82240	-4.14465
H	-3.13789	4.52810	-3.53157
H	-3.26743	3.19555	-4.70671
H	-1.97196	4.40437	-4.87212
C	-0.04755	4.88235	1.19326
H	-0.67250	4.05585	1.56169
C	-0.77588	6.19841	1.49148
H	-1.76340	6.22142	1.00964
H	-0.19345	7.05823	1.12400
H	-0.91969	6.32062	2.57525
C	1.28583	4.81032	1.93767
H	1.80879	3.86738	1.71795
H	1.10907	4.84239	3.02244
H	1.94674	5.65510	1.68766
Sn	0.25728	-0.95512	-0.98214
Rb	2.03706	2.28360	-1.68400
C	2.15059	-2.12164	-0.27611
C	2.05453	-3.37581	0.38183
C	3.14857	-4.24588	0.51480
H	3.00636	-5.19931	1.03163
C	4.39697	-3.91390	0.00516
H	5.24345	-4.59488	0.10328
C	4.53728	-2.68372	-0.62370
H	5.51055	-2.38222	-1.02131
C	3.44768	-1.80851	-0.76010
C	3.83828	-0.51953	-1.42907
C	4.50314	0.46673	-0.66389
C	5.11720	1.54244	-1.32188
H	5.66572	2.28723	-0.73972
C	5.05109	1.66615	-2.70856
H	5.55187	2.49639	-3.21093
C	4.35148	0.71588	-3.45332
H	4.30487	0.81426	-4.53989
C	3.74543	-0.38591	-2.83399
C	4.59611	0.37465	0.85320
H	3.97115	-0.47171	1.17042
C	6.03445	0.09319	1.29767
H	6.41061	-0.83739	0.84976
H	6.70837	0.91210	1.00010
H	6.08659	-0.00650	2.39182
C	4.03729	1.62893	1.53097
H	2.97314	1.77279	1.28219
H	4.09473	1.52943	2.62508
H	4.60053	2.53436	1.25182
C	3.08690	-1.46312	-3.68227
H	2.43942	-2.05284	-3.01706
C	4.15356	-2.39992	-4.26169
H	4.74833	-2.86396	-3.46248
H	3.68217	-3.20202	-4.84869
H	4.83874	-1.84779	-4.92446
C	2.19940	-0.89009	-4.78757
H	1.44120	-0.20955	-4.37160
H	2.78323	-0.35330	-5.55170
H	1.66434	-1.70509	-5.29595
C	0.81697	-3.91570	1.04377

C	0.69974	-3.81269	2.44933
C	-0.30118	-4.54326	3.10644
H	-0.37264	-4.49862	4.19616
C	-1.19602	-5.33654	2.39055
H	-1.95772	-5.91678	2.91556
C	-1.10460	-5.39642	0.99942
H	-1.80408	-6.02333	0.44251
C	-0.10157	-4.70158	0.30960
C	1.65078	-2.95293	3.27096
H	2.28661	-2.39963	2.56603
C	0.89538	-1.91657	4.10778
H	0.32135	-1.23019	3.46403
H	0.21314	-2.38996	4.83272
H	1.60296	-1.29643	4.67789
C	2.55966	-3.82240	4.14465
H	3.13789	-4.52810	3.53157
H	3.26743	-3.19555	4.70671
H	1.97196	-4.40437	4.87212
C	0.04755	-4.88235	-1.19326
H	0.67250	-4.05585	-1.56169
C	0.77588	-6.19841	-1.49148
H	1.76340	-6.22142	-1.00964
H	0.19345	-7.05823	-1.12400
H	0.91969	-6.32062	-2.57525
C	-1.28583	-4.81032	-1.93767
H	-1.80879	-3.86738	-1.71795
H	-1.10907	-4.84239	-3.02244
H	-1.94674	-5.65510	-1.68766

Rb(0)

Methods A, B and C

SCF (BP86/BS1) Energy = -24.1221090118
Enthalpy 0K = -24.122109
Enthalpy 298K = -24.119749
Free Energy 298K = -24.139043
SCF (BP86-D3^{BJ}) Energy = -24.12210901
SCF (C_6H_6) Energy = -24.12422605
SCF (BS2) Energy = -24.12210901
SCF (BP86-D3^{BJ}(CPCM=C_6H_6)/BS3) Energy = -3004.32186991
SCF (BP86-D3^{BJ}(SMD=C_6H_6)/BS3) Energy = -3004.319623

Rb	0.00000	0.00000	0.00000

Methods D and E

SCF (BP86/BS4) Energy = -24.1218579598
Enthalpy 0K = -24.121858
Enthalpy 298K = -24.119497
Free Energy 298K = -24.138792
SCF (BP86-D3^{BJ}) Energy = -24.1218579598
SCF (PCM=C_6H_6) Energy = -24.124000
SCF (SMD=C_6H_6) Energy = -24.124613
SCF (BS5) Energy = -24.1222139551

Rb	0.00000	0.00000	0.00000

Method F

SCF (M062X/BS4) Energy = -24.0228179274
Enthalpy 0K = -24.022818
Enthalpy 298K = -24.020457
Free Energy 298K = -24.039752
SCF (M062X-D3) Energy = -24.022818
SCF (SMD=C_6H_6) Energy = -24.025556
SCF (BS5) Energy = -24.023166

Rb	0.00000	0.00000	0.00000

1^{Cs}

Methods A, B and C

SCF (BP86/BS1) Energy = -2378.27992722
Enthalpy 0K = -2377.137740
Enthalpy 298K = -2377.062184
Free Energy 298K = -2377.256631
Lowest Frequency = 7.8247 cm^{-1}
Second Frequency = 12.0120 cm^{-1}
SCF (BP86-D3^{BJ}) Energy = -2378.67043104
SCF (C_6H_6) Energy = -2378.28889340
SCF (BS2) Energy = -2800.84723655
SCF (BP86-D3^{BJ}(CPCM=C_6H_6)/BS3) Energy = -30689.478551
SCF (BP86-D3^{BJ}(SMD=C_6H_6)/BS3) Energy = -30689.507952

Sn	-0.35060	1.02136	0.96507
Cs	-2.14211	-2.40260	1.80697
C	-2.26530	2.21139	0.25754
C	-2.15865	3.49702	-0.37264
C	-3.24322	4.40614	-0.42771
H	-3.09690	5.37298	-0.92484
C	-4.48729	4.09054	0.12649
H	-5.31943	4.80112	0.08691
C	-4.64176	2.83211	0.71559
H	-5.61357	2.54065	1.13263
C	-3.56821	1.91056	0.78003
C	-3.99279	0.60724	1.41796
C	-4.65713	-0.36867	0.61318
C	-5.32412	-1.44285	1.24330
H	-5.86603	-2.17300	0.63046
C	-5.33307	-1.57178	2.64101
H	-5.87969	-2.39456	3.11666
C	-4.65308	-0.62702	3.42668
H	-4.67091	-0.72263	4.51846
C	-3.98529	0.46983	2.83903
C	-4.71941	-0.24288	-0.91249
H	-4.06825	0.60166	-1.19450
C	-6.15400	0.09255	-1.38012
H	-6.51326	1.02680	-0.91787
H	-6.86295	-0.71242	-1.11484
H	-6.18357	0.21906	-2.47657
C	-4.18066	-1.49857	-1.63142
H	-3.11494	-1.67333	-1.39459
H	-4.24828	-1.37000	-2.72561
H	-4.75753	-2.40581	-1.37341
C	-3.34438	1.53166	3.73721
H	-2.73284	2.18085	3.08581
C	-4.43000	2.40855	4.40409
H	-5.06400	2.90119	3.64874
H	-3.96361	3.19445	5.02372
H	-5.08514	1.80573	5.05864
C	-2.39737	0.92837	4.79547
H	-1.57646	0.36893	4.31351
H	-2.92517	0.25844	5.49870
H	-1.93758	1.73400	5.39350
C	-0.93967	4.04245	-1.08151
C	-0.86339	3.92659	-2.50353
C	0.10195	4.68196	-3.20580

H	0.13908	4.62286	-4.30019
C	0.99303	5.52914	-2.52881
H	1.71864	6.12992	-3.08969
C	0.93404	5.61736	-1.12847
H	1.62192	6.28922	-0.60250
C	-0.02610	4.89295	-0.38858
C	-1.84087	3.04817	-3.29136
H	-2.45197	2.50133	-2.55360
C	-1.11692	1.99576	-4.15911
H	-0.52826	1.29553	-3.53800
H	-0.44687	2.46198	-4.90499
H	-1.85152	1.38852	-4.71588
C	-2.79691	3.91092	-4.14594
H	-3.36256	4.61756	-3.51637
H	-3.52173	3.27146	-4.67959
H	-2.24492	4.49723	-4.90254
C	-0.12867	5.10362	1.12457
H	-0.79131	4.31239	1.51808
C	-0.77821	6.47166	1.43939
H	-1.78135	6.54954	0.98899
H	-0.16426	7.30453	1.05151
H	-0.88191	6.60699	2.53029
C	1.22871	4.96049	1.84353
H	1.65986	3.95872	1.67205
H	1.09290	5.07760	2.93250
H	1.95823	5.72541	1.52004
Sn	0.35060	-1.02136	-0.96507
Cs	2.14211	2.40260	-1.80697
C	2.26530	-2.21139	-0.25754
C	2.15865	-3.49702	0.37264
C	3.24322	-4.40614	0.42771
H	3.09690	-5.37298	0.92484
C	4.48729	-4.09054	-0.12649
H	5.31943	-4.80112	-0.08691
C	4.64176	-2.83211	-0.71559
H	5.61357	-2.54065	-1.13263
C	3.56821	-1.91056	-0.78003
C	3.99279	-0.60724	-1.41796
C	4.65713	0.36867	-0.61318
C	5.32412	1.44285	-1.24330
H	5.86603	2.17300	-0.63046
C	5.33307	1.57178	-2.64101
H	5.87969	2.39456	-3.11666
C	4.65308	0.62702	-3.42668
H	4.67091	0.72263	-4.51846
C	3.98529	-0.46983	-2.83903
C	4.71941	0.24288	0.91249
H	4.06825	-0.60166	1.19450
C	6.15400	-0.09255	1.38012
H	6.51326	-1.02680	0.91787
H	6.86295	0.71242	1.11484
H	6.18357	-0.21906	2.47657
C	4.18066	1.49857	1.63142
H	3.11494	1.67333	1.39459
H	4.24828	1.37000	2.72561
H	4.75753	2.40581	1.37341
C	3.34438	-1.53166	-3.73721
H	2.73284	-2.18085	-3.08581
C	4.43000	-2.40855	-4.40409
H	5.06400	-2.90119	-3.64874
H	3.96361	-3.19445	-5.02372
H	5.08514	-1.80573	-5.05864
C	2.39737	-0.92837	-4.79547
H	1.57646	-0.36893	-4.31351
H	2.92517	-0.25844	-5.49870
H	1.93758	-1.73400	-5.39350
C	0.93967	-4.04245	1.08151
C	0.86339	-3.92659	2.50353
C	-0.10195	-4.68196	3.20580
H	-0.13908	-4.62286	4.30019
C	-0.99303	-5.52914	2.52881
H	-1.71864	-6.12992	3.08969
C	-0.93404	-5.61736	1.12847
H	-1.62192	-6.28922	0.60250
C	0.02610	-4.89295	0.38858
C	1.84087	-3.04817	3.29136
H	2.45197	-2.50133	2.55360
C	1.11692	-1.99576	4.15911
H	0.52826	-1.29553	3.53800
H	0.44687	-2.46198	4.90499
H	1.85152	-1.38852	4.71588
C	2.79691	-3.91092	4.14594
H	3.36256	-4.61756	3.51637
H	3.52173	-3.27146	4.67959
H	2.24492	-4.49723	4.90254
C	0.12867	-5.10362	-1.12457
H	0.79131	-4.31239	-1.51808
C	0.77821	-6.47166	-1.43939
H	1.78135	-6.54954	-0.98899
H	0.16426	-7.30453	-1.05151
H	0.88191	-6.60699	-2.53029
C	-1.22871	-4.96049	-1.84353
H	-1.65986	-3.95872	-1.67205
H	-1.09290	-5.07760	-2.93250
H	-1.95823	-5.72541	-1.52004

Methods D and E

SCF (BP86/BS4) Energy = -2798.59007703
Enthalpy 0K = -2797.453356
Enthalpy 298K = -2797.377802
Free Energy 298K = -2797.572490
Lowest Frequency = 6.2155 cm^{-1}
Second Frequency = 11.6036 cm^{-1}
SCF (BP86-D3^{BJ}) Energy = -2798.97956904
SCF (PCM=C_6H_6) Energy = -2798.59811696
SCF (SMD=C_6H_6) Energy = -2798.63351687
SCF (BS5) Energy = -2801.09320974

Sn	-0.36595	1.02313	0.94326
Cs	-2.14802	-2.41163	1.81554
C	-2.27435	2.21387	0.24815
C	-2.16297	3.50308	-0.37676
C	-3.24803	4.41429	-0.42563
H	-3.10271	5.38908	-0.92149
C	-4.49361	4.09800	0.12898
H	-5.33020	4.81345	0.09134
C	-4.64803	2.83745	0.71752
H	-5.62573	2.54756	1.13855
C	-3.57552	1.91211	0.77786
C	-4.00478	0.61232	1.41962
C	-4.67854	-0.36233	0.61745
C	-5.36083	-1.42569	1.25337
H	-5.91360	-2.15862	0.64214
C	-5.37272	-1.54733	2.65302
H	-5.93389	-2.36512	3.13487
C	-4.68145	-0.60634	3.43617
H	-4.70108	-0.70007	4.53445
C	-3.99751	0.48015	2.84374

C	-4.73298	-0.24475	-0.90968
H	-4.08889	0.61441	-1.18768
C	-6.16348	0.06985	-1.39677
H	-6.55156	0.99892	-0.93169
H	-6.87021	-0.75257	-1.15234
H	-6.17991	0.20824	-2.49862
C	-4.15996	-1.49009	-1.61588
H	-3.09108	-1.65166	-1.35428
H	-4.20148	-1.36457	-2.71833
H	-4.73086	-2.41274	-1.37158
C	-3.34306	1.53818	3.73763
H	-2.72051	2.17671	3.07578
C	-4.41021	2.43893	4.39820
H	-5.04895	2.93359	3.63891
H	-3.92759	3.23389	5.00539
H	-5.07327	1.85498	5.07276
C	-2.40105	0.93002	4.79464
H	-1.59069	0.34191	4.31399
H	-2.93802	0.27804	5.51759
H	-1.91278	1.73579	5.38189
C	-0.94651	4.05549	-1.08436
C	-0.87445	3.95010	-2.50961
C	0.08092	4.72243	-3.21047
H	0.11650	4.67362	-4.31177
C	0.96786	5.57306	-2.52961
H	1.68838	6.18972	-3.09214
C	0.91543	5.64918	-1.12673
H	1.60584	6.32589	-0.59701
C	-0.03404	4.90753	-0.38680
C	-1.84621	3.06551	-3.29874
H	-2.46745	2.52798	-2.55343
C	-1.11602	1.99650	-4.13706
H	-0.52491	1.30757	-3.49440
H	-0.43876	2.44721	-4.89557
H	-1.84722	1.36754	-4.68720
C	-2.79659	3.91247	-4.17135
H	-3.36302	4.64219	-3.55747
H	-3.53162	3.26322	-4.69257
H	-2.24294	4.48162	-4.94921
C	-0.12978	5.10278	1.12939
H	-0.78795	4.29597	1.51598
C	-0.79188	6.45681	1.46853
H	-1.80045	6.53900	1.01547
H	-0.18227	7.31011	1.09983
H	-0.90313	6.57172	2.56763
C	1.22957	4.95791	1.84001
H	1.67628	3.96006	1.64480
H	1.09896	5.04695	2.93887
H	1.95533	5.74192	1.53228
Sn	0.36595	-1.02313	-0.94326
Cs	2.14802	2.41163	-1.81554
C	2.27435	-2.21387	-0.24815
C	2.16297	-3.50308	0.37676
C	3.24803	-4.41429	0.42563
H	3.10271	-5.38908	0.92149
C	4.49361	-4.09800	-0.12898
H	5.33020	-4.81345	-0.09134
C	4.64803	-2.83745	-0.71752
H	5.62573	-2.54756	-1.13855
C	3.57552	-1.91211	-0.77786
C	4.00478	-0.61232	-1.41962
C	4.67854	0.36233	-0.61745
C	5.36083	1.42569	-1.25337
H	5.91360	2.15862	-0.64214

C	5.37272	1.54733	-2.65302
H	5.93389	2.36512	-3.13487
C	4.68145	0.60634	-3.43617
H	4.70108	0.70007	-4.53445
C	3.99751	-0.48015	-2.84374
C	4.73298	0.24475	0.90968
H	4.08889	-0.61441	1.18768
C	6.16348	-0.06985	1.39677
H	6.55156	-0.99892	0.93169
H	6.87021	0.75257	1.15234
H	6.17991	-0.20824	2.49862
C	4.15996	1.49009	1.61588
H	3.09108	1.65166	1.35428
H	4.20148	1.36457	2.71833
H	4.73086	2.41274	1.37158
C	3.34306	-1.53818	-3.73763
H	2.72051	-2.17671	-3.07578
C	4.41021	-2.43893	-4.39820
H	5.04895	-2.93359	-3.63891
H	3.92759	-3.23389	-5.00539
H	5.07327	-1.85498	-5.07276
C	2.40105	-0.93002	-4.79464
H	1.59069	-0.34191	-4.31399
H	2.93802	-0.27804	-5.51759
H	1.91278	-1.73579	-5.38189
C	0.94651	-4.05549	1.08436
C	0.87445	-3.95010	2.50961
C	-0.08092	-4.72243	3.21047
H	-0.11650	-4.67362	4.31177
C	-0.96786	-5.57306	2.52961
H	-1.68838	-6.18972	3.09214
C	-0.91543	-5.64918	1.12673
H	-1.60584	-6.32589	0.59701
C	0.03404	-4.90753	0.38680
C	1.84621	-3.06551	3.29874
H	2.46745	-2.52798	2.55343
C	1.11602	-1.99650	4.13706
H	0.52491	-1.30757	3.49440
H	0.43876	-2.44721	4.89557
H	1.84722	-1.36754	4.68720
C	2.79659	-3.91247	4.17135
H	3.36302	-4.64219	3.55747
H	3.53162	-3.26322	4.69257
H	2.24294	-4.48162	4.94921
C	0.12978	-5.10278	-1.12939
H	0.78795	-4.29597	-1.51598
C	0.79188	-6.45681	-1.46853
H	1.80045	-6.53900	-1.01547
H	0.18227	-7.31011	-1.09983
H	0.90313	-6.57172	-2.56763
C	-1.22957	-4.95791	-1.84001
H	-1.67628	-3.96006	-1.64480
H	-1.09896	-5.04695	-2.93887
H	-1.95533	-5.74192	-1.53228

Method F

SCF (M062X/BS4) Energy = -2797.02561745

Enthalpy 0K = -2795.843476

Enthalpy 298K = -2795.770780

Free Energy 298K = -2795.957656

Lowest Frequency = 9.2903 cm^{-1}

Second Frequency = 10.0201 cm^{-1}

SCF (M062X-D3) Energy = -2797.04444870

SCF (SMD=C_6H_6) Energy = -2797.07008386
SCF (BS5) Energy = -2799.61059471

Sn	-0.29748	0.96908	0.96458
Cs	-2.15265	-2.42084	1.82994
C	-2.19597	2.15253	0.25888
C	-2.10142	3.41568	-0.38312
C	-3.19038	4.29731	-0.49048
H	-3.04533	5.25529	-0.99789
C	-4.43539	3.97148	0.02858
H	-5.27737	4.66040	-0.05113
C	-4.57592	2.73482	0.64270
H	-5.54654	2.43451	1.04752
C	-3.49130	1.84880	0.75550
C	-3.91385	0.56746	1.42091
C	-4.59821	-0.39943	0.64880
C	-5.30120	-1.42200	1.30141
H	-5.86542	-2.14857	0.71092
C	-5.30711	-1.51304	2.69215
H	-5.88094	-2.29766	3.18960
C	-4.57850	-0.59240	3.44555
H	-4.58141	-0.66802	4.53532
C	-3.87813	0.45385	2.82949
C	-4.61934	-0.33610	-0.87216
H	-3.97005	0.49680	-1.17558
C	-6.03021	-0.04756	-1.39233
H	-6.41667	0.89357	-0.97569
H	-6.72840	-0.85447	-1.11916
H	-6.02633	0.03791	-2.48894
C	-4.03891	-1.61041	-1.49261
H	-2.98144	-1.74141	-1.20917
H	-4.06406	-1.54892	-2.59073
H	-4.60819	-2.50763	-1.19808
C	-3.16868	1.49273	3.68424
H	-2.51016	2.06999	3.01877
C	-4.18508	2.45810	4.30394
H	-4.77718	2.95947	3.52532
H	-3.67083	3.23039	4.89491
H	-4.87852	1.92220	4.97144
C	-2.28194	0.85740	4.75655
H	-1.54388	0.17768	4.30296
H	-2.86968	0.30329	5.50535
H	-1.71953	1.63787	5.28928
C	-0.88492	3.98038	-1.06442
C	-0.81034	3.90917	-2.47485
C	0.11809	4.71263	-3.15157
H	0.15320	4.69081	-4.24398
C	0.98477	5.55046	-2.45216
H	1.68629	6.19015	-2.99163
C	0.94646	5.57162	-1.05782
H	1.62711	6.22878	-0.51194
C	0.01765	4.79865	-0.34750
C	-1.73331	3.00362	-3.27906
H	-2.35423	2.44709	-2.56369
C	-0.93777	1.97190	-4.08490
H	-0.37483	1.29696	-3.41904
H	-0.23928	2.45269	-4.79011
H	-1.61657	1.33741	-4.67388
C	-2.66710	3.82089	-4.17585
H	-3.26188	4.52841	-3.58059
H	-3.35977	3.15826	-4.71520
H	-2.10024	4.39653	-4.92443
C	-0.07010	4.92636	1.16538
H	-0.67681	4.08491	1.53069

C	-0.78468	6.22795	1.54609
H	-1.79185	6.26672	1.10747
H	-0.22015	7.10391	1.18889
H	-0.88269	6.30712	2.63891
C	1.29758	4.82607	1.84227
H	1.79917	3.88301	1.57576
H	1.17639	4.83249	2.93519
H	1.95395	5.66998	1.57734
Sn	0.29748	-0.96908	-0.96458
Cs	2.15265	2.42084	-1.82994
C	2.19597	-2.15253	-0.25888
C	2.10142	-3.41568	0.38312
C	3.19038	-4.29731	0.49048
H	3.04533	-5.25529	0.99789
C	4.43539	-3.97148	-0.02858
H	5.27737	-4.66040	0.05113
C	4.57592	-2.73482	-0.64270
H	5.54654	-2.43451	-1.04752
C	3.49130	-1.84880	-0.75550
C	3.91385	-0.56746	-1.42091
C	4.59821	0.39943	-0.64880
C	5.30120	1.42200	-1.30141
H	5.86542	2.14857	-0.71092
C	5.30711	1.51304	-2.69215
H	5.88094	2.29766	-3.18960
C	4.57850	0.59240	-3.44555
H	4.58141	0.66802	-4.53532
C	3.87813	-0.45385	-2.82949
C	4.61934	0.33610	0.87216
H	3.97005	-0.49680	1.17558
C	6.03021	0.04756	1.39233
H	6.41667	-0.89357	0.97569
H	6.72840	0.85447	1.11916
H	6.02633	-0.03791	2.48894
C	4.03891	1.61041	1.49261
H	2.98144	1.74141	1.20917
H	4.06406	1.54892	2.59073
H	4.60819	2.50763	1.19808
C	3.16868	-1.49273	-3.68424
H	2.51016	-2.06999	-3.01877
C	4.18508	-2.45810	-4.30394
H	4.77718	-2.95947	-3.52532
H	3.67083	-3.23039	-4.89491
H	4.87852	-1.92220	-4.97144
C	2.28194	-0.85740	-4.75655
H	1.54388	-0.17768	-4.30296
H	2.86968	-0.30329	-5.50535
H	1.71953	-1.63787	-5.28928
C	0.88492	-3.98038	1.06442
C	0.81034	-3.90917	2.47485
C	-0.11809	-4.71263	3.15157
H	-0.15320	-4.69081	4.24398
C	-0.98477	-5.55046	2.45216
H	-1.68629	-6.19015	2.99163
C	-0.94646	-5.57162	1.05782
H	-1.62711	-6.22878	0.51194
C	-0.01765	-4.79865	0.34750
C	1.73331	-3.00362	3.27906
H	2.35423	-2.44709	2.56369
C	0.93777	-1.97190	4.08490
H	0.37483	-1.29696	3.41904
H	0.23928	-2.45269	4.79011
H	1.61657	-1.33741	4.67388
C	2.66710	-3.82089	4.17585

H	3.26188	-4.52841	3.58059
H	3.35977	-3.15826	4.71520
H	2.10024	-4.39653	4.92443
C	0.07010	-4.92636	-1.16538
H	0.67681	-4.08491	-1.53069
C	0.78468	-6.22795	-1.54609
H	1.79185	-6.26672	-1.10747
H	0.22015	-7.10391	-1.18889
H	0.88269	-6.30712	-2.63891
C	-1.29758	-4.82607	-1.84227
H	-1.79917	-3.88301	-1.57576
H	-1.17639	-4.83249	-2.93519
H	-1.95395	-5.66998	-1.57734

Cs(0)

Methods A, B and C

SCF (BP86/BS1) Energy =
-20.1880347311
Enthalpy 0K = -20.188035
Enthalpy 298K = -20.185674
Free Energy 298K = -20.205603
SCF (BP86-$D3^{BJ}$) Energy =
-20.18803473
SCF (C_6H_6) Energy = -20.18979028
SCF (BS2) Energy = -20.18803473
SCF (BP86-$D3^{BJ}$(CPCM=C_6H_6)/BS3) Energy =
-7916.40420542
SCF (BP86-$D3^{BJ}$(SMD=C_6H_6)/BS3) Energy =
-7916.401348

Cs	0.00000	0.00000	0.00000

Methods D and E

SCF (BP86/BS4) Energy =
-20.1884145529
Enthalpy 0K = -20.188415
Enthalpy 298K = -20.186054
Free Energy 298K = -20.205983
SCF (BP86-$D3^{BJ}$) Energy =
-20.1884145529
SCF (PCM=C_6H_6) Energy = -20.190246
SCF (SMD=C_6H_6) Energy = -20.190490
SCF (BS5) Energy = -20.1884145529

Cs	0.00000	0.00000	0.00000

Method F

SCF (M062X/BS4) Energy =
-20.0750573672
Enthalpy 0K = -20.075057
Enthalpy 298K = -20.072697
Free Energy 298K = -20.092626
SCF (M062X-D3) Energy =
-20.0750573672
SCF (SMD=C_6H_6) Energy = -20.0771754158
SCF (BS5) Energy = -20.0750573672

Cs	0.00000	0.00000	0.00000

$1^{Li\bullet-}$

SCF (BP86/BS1) Energy = -2352.94853556
Enthalpy 0K = -2351.808010
Enthalpy 298K = -2351.733610
Free Energy 298K = -2351.921817
Lowest Frequency = 9.6391 cm^{-1}
Second Frequency = 18.7105 cm^{-1}

Sn	-1.06737	-0.10642	-1.01562
Sn	1.06709	0.10698	1.01602
C	-2.85514	-0.99235	0.17610
C	-2.98866	-2.40088	0.40178
C	-4.21665	-2.99039	0.77593
H	-4.26859	-4.07750	0.91934
C	-5.36277	-2.20461	0.96381
H	-6.31652	-2.66459	1.24626
C	-1.80828	-3.32803	0.25924
C	-1.47493	-3.87362	-1.01970
C	-0.36597	-4.75392	-1.12963
H	-0.09084	-5.15943	-2.10751
C	0.39689	-5.09680	0.00918
H	1.26572	-5.75546	-0.09224
C	0.04763	-4.59078	1.26550
H	0.64338	-4.86532	2.14145
C	-1.05130	-3.69456	1.42151
C	-2.38013	-3.64129	-2.23266
H	-2.96944	-2.73176	-2.02499
C	-3.36555	-4.82777	-2.37760
H	-2.82271	-5.77476	-2.55130
H	-4.04554	-4.66358	-3.23301
H	-3.98129	-4.94865	-1.47076
C	-1.61545	-3.40470	-3.54817
H	-0.93979	-2.53858	-3.44444
H	-2.32886	-3.18648	-4.36282
H	-1.02643	-4.28712	-3.85906
C	-1.45053	-3.22432	2.82234
H	-2.09322	-2.33637	2.69149
C	-2.29225	-4.31364	3.53270
H	-3.20414	-4.55509	2.96229
H	-2.59788	-3.97444	4.53923
H	-1.70739	-5.24428	3.65225
C	-0.25678	-2.81532	3.70875
H	0.39226	-3.67621	3.95421
H	-0.62347	-2.40480	4.66661
H	0.36095	-2.04468	3.21280
C	2.85496	0.99217	-0.17612
C	2.98867	2.40062	-0.40215
C	4.21676	2.98988	-0.77641
H	4.26884	4.07695	-0.92009
C	5.36279	2.20391	-0.96403
H	6.31660	2.66369	-1.24658
C	1.80851	3.32811	-0.25988
C	1.05146	3.69429	-1.42227
C	-0.04702	4.59108	-1.26659
H	-0.64278	4.86537	-2.14261
C	-0.39581	5.09809	-0.01055
H	-1.26428	5.75725	0.09061
C	0.36712	4.75557	1.12834
H	0.09240	5.16186	2.10602
C	1.47562	3.87469	1.01876
C	1.45029	3.22306	-2.82288
H	2.09225	2.33464	-2.69161
C	0.25621	2.81458	-3.70910
H	-0.36211	2.04465	-3.21278
H	0.62259	2.40333	-4.66676
H	-0.39216	3.67587	-3.95497
C	2.29287	4.31135	-3.53380
H	1.70874	5.24239	-3.65379
H	2.59818	3.97140	-4.54017

H	3.20497	4.55234	-2.96355
C	2.38071	3.64245	2.23182
H	2.96975	2.73267	2.02446
C	3.36650	4.82866	2.37647
H	3.98231	4.94911	1.46962
H	4.04641	4.66448	3.23194
H	2.82395	5.77586	2.54990
C	1.61584	3.40649	3.54733
H	1.02682	4.28909	3.85774
H	2.32912	3.18858	4.36218
H	0.94013	2.54038	3.44390
Li	0.43173	-2.51576	-0.07831
C	-4.03656	-0.21569	0.41668
C	-5.25823	-0.81597	0.79721
H	-6.13529	-0.17777	0.96694
C	-4.04226	1.28981	0.31084
C	-4.41010	1.92762	-0.90840
C	-4.49473	3.33392	-0.94855
H	-4.78285	3.82986	-1.88253
C	-4.21901	4.10826	0.18644
H	-4.29630	5.20121	0.13990
C	-3.86118	3.47793	1.38573
H	-3.66391	4.08513	2.27692
C	-3.77076	2.07276	1.47050
C	-4.77733	1.10948	-2.14944
H	-4.39278	0.08732	-1.98574
C	-6.31361	1.01914	-2.30576
H	-6.75771	2.02268	-2.44037
H	-6.58232	0.40873	-3.18731
H	-6.77865	0.55929	-1.41755
C	-4.12157	1.64102	-3.43952
H	-3.02515	1.67477	-3.32250
H	-4.35647	0.97358	-4.28815
H	-4.48310	2.65098	-3.70752
C	-3.45757	1.41594	2.81824
H	-3.21915	0.35811	2.61509
C	-4.70224	1.44893	3.73603
H	-5.55726	0.93540	3.26481
H	-4.49180	0.95044	4.69983
H	-5.00824	2.48923	3.95179
C	-2.23483	2.03109	3.52637
H	-2.38568	3.09755	3.77454
H	-2.03446	1.49913	4.47357
H	-1.33183	1.93932	2.89661
C	4.03631	0.21529	-0.41640
C	5.25806	0.81534	-0.79705
H	6.13504	0.17697	-0.96662
C	4.04196	-1.29020	-0.31028
C	3.77162	-2.07330	-1.47009
C	3.86232	-3.47846	-1.38515
H	3.66594	-4.08575	-2.27648
C	4.21928	-4.10861	-0.18552
H	4.29684	-5.20153	-0.13882
C	4.49374	-3.33411	0.94968
H	4.78115	-3.82994	1.88393
C	4.40882	-1.92785	0.90936
C	3.45915	-1.41678	-2.81814
H	3.22164	-0.35865	-2.61548
C	2.23590	-2.03126	-3.52600
H	1.33292	-1.93826	-2.89639
H	2.03611	-1.49978	-4.47359
H	2.38582	-3.09803	-3.77336
C	4.70382	-1.45122	-3.73587
H	5.00895	-2.49186	-3.95123
H	4.49386	-0.95292	-4.69986
H	5.55928	-0.93826	-3.26481
C	4.77489	-1.10948	2.15059
H	4.38927	-0.08770	1.98693
C	6.31104	-1.01756	2.30730
H	6.77580	-0.55706	1.41929
H	6.57887	-0.40701	3.18903
H	6.75613	-2.02066	2.44187
C	4.11941	-1.64185	3.44046
H	4.48219	-2.65131	3.70865
H	4.35316	-0.97406	4.28914
H	3.02307	-1.67704	3.32312
Li	-0.43142	2.51631	0.07819

1$^{Na\bullet-}$

SCF (BP86/BS1) Energy = -2338.33217428
Enthalpy 0K = -2337.194778
Enthalpy 298K = -2337.119278
Free Energy 298K = -2337.310146
Lowest Frequency = 10.2539 cm^{-1}
Second Frequency = 15.2551 cm^{-1}

Sn	-0.98483	0.00013	1.09443
Na	-0.00109	-2.90433	-0.00030
C	-3.01528	0.00076	-0.04554
C	-3.75192	1.21210	-0.26217
C	-5.12983	1.20622	-0.57792
H	-5.64950	2.16264	-0.72150
C	-5.83366	0.00146	-0.71640
H	-6.90460	0.00173	-0.94911
C	-5.13056	-1.20366	-0.57739
H	-5.65079	-2.15983	-0.72058
C	-3.75266	-1.21023	-0.26157
C	-3.09324	-2.56707	-0.20940
C	-2.63767	-3.16629	-1.42381
C	-2.06640	-4.46581	-1.37645
H	-1.71859	-4.93591	-2.30310
C	-1.95562	-5.16210	-0.16460
H	-1.51610	-6.16649	-0.14650
C	-2.42635	-4.57753	1.02534
H	-2.34957	-5.13257	1.96615
C	-3.00026	-3.28185	1.02201
C	-2.80474	-2.47038	-2.77712
H	-3.03627	-1.41177	-2.56921
C	-4.00506	-3.07088	-3.54798
H	-4.93959	-2.97235	-2.97081
H	-3.84425	-4.14516	-3.75461
H	-4.14407	-2.55665	-4.51644
C	-1.53124	-2.50202	-3.64692
H	-0.67983	-2.02971	-3.12261
H	-1.69924	-1.94438	-4.58572
H	-1.24287	-3.53195	-3.92752
C	-3.62934	-2.72896	2.30374
H	-3.73147	-1.63816	2.16727
C	-5.04694	-3.32246	2.49530
H	-5.69612	-3.08986	1.63483
H	-5.52177	-2.90871	3.40355
H	-5.00467	-4.42183	2.60425
C	-2.77085	-2.95307	3.56312
H	-1.77397	-2.49879	3.43097
H	-2.64964	-4.02543	3.80506
H	-3.25024	-2.47454	4.43583
C	-3.09163	2.56854	-0.21049
C	-2.63561	3.16709	-1.42505

C	-2.06322	4.46613	-1.37799
H	-1.71499	4.93570	-2.30475
C	-1.95182	5.16260	-0.16630
H	-1.51144	6.16662	-0.14844
C	-2.42304	4.57872	1.02379
H	-2.34583	5.13393	1.96446
C	-2.99805	3.28352	1.02076
C	-2.80363	2.47112	-2.77821
H	-3.03599	1.41275	-2.57004
C	-1.53048	2.50156	-3.64854
H	-0.67923	2.02865	-3.12451
H	-1.24137	3.53121	-3.92943
H	-1.69937	1.94394	-4.58719
C	-4.00374	3.07247	-3.54875
H	-4.93817	2.97465	-2.97130
H	-4.14342	2.55825	-4.51714
H	-3.84220	4.14661	-3.75552
C	-3.62773	2.73151	2.30256
H	-3.73127	1.64083	2.16615
C	-5.04458	3.32678	2.49412
H	-5.69403	3.09497	1.63364
H	-5.00094	4.42610	2.60303
H	-5.51994	2.91364	3.40236
C	-2.76889	2.95455	3.56189
H	-1.77270	2.49872	3.42976
H	-3.24897	2.47689	4.43470
H	-2.64604	4.02677	3.80365
Sn	0.98467	-0.00032	-1.09451
Na	0.00091	2.90420	0.00014
C	3.01520	-0.00069	0.04544
C	3.75198	-1.21203	0.26163
C	5.12987	-1.20615	0.57748
H	5.64962	-2.16257	0.72074
C	5.83357	-0.00137	0.71642
H	6.90451	-0.00161	0.94917
C	5.13035	1.20372	0.57784
H	5.65048	2.15990	0.72141
C	3.75245	1.21029	0.26201
C	3.09294	2.56713	0.21049
C	2.63755	3.16585	1.42521
C	2.06623	4.46537	1.37845
H	1.71849	4.93506	2.30534
C	1.95529	5.16217	0.16692
H	1.51573	6.16656	0.14929
C	2.42590	4.57812	-1.02334
H	2.34906	5.13357	-1.96389
C	2.99983	3.28244	-1.02060
C	2.80496	2.46951	2.77826
H	3.03671	1.41103	2.56998
C	4.00526	3.07003	3.54914
H	4.93974	2.97184	2.97183
H	3.84427	4.14422	3.75611
H	4.14449	2.55554	4.51744
C	1.53162	2.50060	3.64830
H	0.68018	2.02838	3.12395
H	1.69988	1.94264	4.58686
H	1.24313	3.53037	3.92935
C	3.62879	2.73008	-2.30261
H	3.73047	1.63915	-2.16681
C	5.04664	3.32311	-2.49374
H	5.69568	3.08965	-1.63340
H	5.52132	2.90977	-3.40224
H	5.00483	4.42257	-2.60195
C	2.77050	2.95532	-3.56193
H	1.77327	2.50173	-3.43009
H	2.65008	4.02788	-3.80342
H	3.24959	2.47683	-4.43482
C	3.09185	-2.56853	0.20936
C	2.63573	-3.16757	1.42364
C	2.06351	-4.46665	1.37603
H	1.71523	-4.93662	2.30257
C	1.95241	-5.16273	0.16407
H	1.51217	-6.16680	0.14579
C	2.42380	-4.57839	-1.02572
H	2.34686	-5.13329	-1.96659
C	2.99864	-3.28312	-1.02214
C	2.80337	-2.47205	2.77709
H	3.03585	-1.41363	2.56936
C	1.52987	-2.50267	3.64691
H	0.67893	-2.02933	3.12276
H	1.24045	-3.53238	3.92722
H	1.69848	-1.94548	4.58588
C	4.00317	-3.07372	3.54783
H	4.93779	-2.97579	2.97070
H	4.14258	-2.55980	4.51641
H	3.84150	-4.14792	3.75422
C	3.62850	-2.73059	-2.30363
H	3.73183	-1.63994	-2.16684
C	5.04548	-3.32557	-2.49507
H	5.69472	-3.09392	-1.63438
H	5.00206	-4.42486	-2.60433
H	5.52096	-2.91208	-3.40309
C	2.76995	-2.95339	-3.56320
H	1.77364	-2.49783	-3.43110
H	3.25011	-2.47533	-4.43576
H	2.64738	-4.02555	-3.80534

$1^{K\bullet-}$

SCF (BP86/BS1) Energy = -2394.57783317
Enthalpy 0K = -2393.441118
Enthalpy 298K = -2393.365561
Free Energy 298K = -2393.556959
Lowest Frequency = 9.1050 cm-1
Second Frequency = 19.0143 cm-1

Sn	-0.28736	1.01353	1.03594
K	-1.89460	-2.13415	1.58132
C	-2.18452	2.16919	0.29760
C	-2.07899	3.44176	-0.35822
C	-3.16494	4.34610	-0.42807
H	-3.02310	5.30899	-0.93614
C	-4.41074	4.02758	0.12865
H	-5.24533	4.73628	0.08156
C	-4.56952	2.76819	0.72213
H	-5.54609	2.47463	1.12905
C	-3.49192	1.85451	0.79953
C	-3.85926	0.52094	1.40979
C	-4.46128	-0.47692	0.57756
C	-4.97759	-1.65377	1.17784
H	-5.46055	-2.41069	0.54807
C	-4.90200	-1.85499	2.56281
H	-5.32201	-2.76335	3.01229
C	-4.30681	-0.87157	3.37863
H	-4.26315	-1.02388	4.46285
C	-3.79272	0.32445	2.82058
C	-4.61307	-0.27967	-0.93308
H	-4.00566	0.59993	-1.20626
C	-6.08316	0.03407	-1.29856

H	-6.43576	0.93932	-0.77651
H	-6.75040	-0.80205	-1.01865
H	-6.18904	0.20123	-2.38596
C	-4.08624	-1.47426	-1.75650
H	-3.01052	-1.64764	-1.56597
H	-4.20139	-1.27370	-2.83669
H	-4.63887	-2.40700	-1.53756
C	-3.27542	1.42738	3.74838
H	-2.71638	2.14123	3.11792
C	-4.46002	2.18267	4.39805
H	-5.11698	2.62623	3.63150
H	-4.09148	2.99851	5.04610
H	-5.07163	1.50436	5.02091
C	-2.29857	0.91194	4.82363
H	-1.42776	0.42982	4.34558
H	-2.77565	0.19554	5.51820
H	-1.92404	1.75583	5.43011
C	-0.83402	3.92038	-1.06998
C	-0.73565	3.73656	-2.48643
C	0.34032	4.34185	-3.18648
H	0.40773	4.22397	-4.27473
C	1.30329	5.10653	-2.51430
H	2.11914	5.58150	-3.07329
C	1.20407	5.28436	-1.11979
H	1.94880	5.89694	-0.59960
C	0.13696	4.70953	-0.38576
C	-1.79177	2.95476	-3.27238
H	-2.43236	2.44458	-2.53297
C	-1.18673	1.86726	-4.18587
H	-0.61570	1.12363	-3.59984
H	-0.51974	2.29598	-4.95702
H	-1.98938	1.32369	-4.71563
C	-2.69016	3.91560	-4.08674
H	-3.19309	4.64195	-3.42666
H	-3.46803	3.35374	-4.63519
H	-2.09746	4.48303	-4.82792
C	-0.02073	5.04328	1.10051
H	-0.73988	4.31694	1.51876
C	-0.61440	6.46295	1.26987
H	-1.59252	6.54738	0.76780
H	0.05714	7.22920	0.84083
H	-0.75806	6.69840	2.34007
C	1.28696	4.89843	1.90334
H	1.66739	3.86480	1.82720
H	1.09985	5.10938	2.97139
H	2.07087	5.60070	1.56344
Sn	0.28736	-1.01353	-1.03594
K	1.89460	2.13415	-1.58132
C	2.18452	-2.16919	-0.29760
C	2.07899	-3.44176	0.35822
C	3.16494	-4.34610	0.42807
H	3.02310	-5.30899	0.93614
C	4.41074	-4.02758	-0.12865
H	5.24533	-4.73628	-0.08156
C	4.56952	-2.76819	-0.72213
H	5.54609	-2.47463	-1.12905
C	3.49192	-1.85451	-0.79953
C	3.85926	-0.52094	-1.40979
C	4.46128	0.47692	-0.57756
C	4.97759	1.65377	-1.17784
H	5.46055	2.41069	-0.54807
C	4.90200	1.85499	-2.56281
H	5.32201	2.76335	-3.01229
C	4.30681	0.87157	-3.37863

H	4.26315	1.02388	-4.46285
C	3.79272	-0.32445	-2.82058
C	4.61307	0.27967	0.93308
H	4.00566	-0.59993	1.20626
C	6.08316	-0.03407	1.29856
H	6.43576	-0.93932	0.77651
H	6.75040	0.80205	1.01865
H	6.18904	-0.20123	2.38596
C	4.08624	1.47426	1.75650
H	3.01052	1.64764	1.56597
H	4.20139	1.27370	2.83669
H	4.63887	2.40700	1.53756
C	3.27542	-1.42738	-3.74838
H	2.71638	-2.14123	-3.11792
C	4.46002	-2.18267	-4.39805
H	5.11698	-2.62623	-3.63150
H	4.09148	-2.99851	-5.04610
H	5.07163	-1.50436	-5.02091
C	2.29857	-0.91194	-4.82363
H	1.42776	-0.42982	-4.34558
H	2.77565	-0.19554	-5.51820
H	1.92404	-1.75583	-5.43011
C	0.83402	-3.92038	1.06998
C	0.73565	-3.73656	2.48643
C	-0.34032	-4.34185	3.18648
H	-0.40773	-4.22397	4.27473
C	-1.30329	-5.10653	2.51430
H	-2.11914	-5.58150	3.07329
C	-1.20407	-5.28436	1.11979
H	-1.94880	-5.89694	0.59960
C	-0.13696	-4.70953	0.38576
C	1.79177	-2.95476	3.27238
H	2.43236	-2.44458	2.53297
C	1.18673	-1.86726	4.18587
H	0.61570	-1.12363	3.59984
H	0.51974	-2.29598	4.95702
H	1.98938	-1.32369	4.71563
C	2.69016	-3.91560	4.08674
H	3.19309	-4.64195	3.42666
H	3.46803	-3.35374	4.63519
H	2.09746	-4.48303	4.82792
C	0.02073	-5.04328	-1.10051
H	0.73988	-4.31694	-1.51876
C	0.61440	-6.46295	-1.26987
H	1.59252	-6.54738	-0.76780
H	-0.05714	-7.22920	-0.84083
H	0.75806	-6.69840	-2.34007
C	-1.28696	-4.89843	-1.90334
H	-1.66739	-3.86480	-1.82720
H	-1.09985	-5.10938	-2.97139
H	-2.07087	-5.60070	-1.56344

$1^{Rb•−}$

SCF (BP86/BS1) Energy = -2386.16119521
Enthalpy 0K = -2385.024636
Enthalpy 298K = -2384.948792
Free Energy 298K = -2385.142067
Lowest Frequency = 9.3114 cm^{-1}
Second Frequency = 18.1024 cm^{-1}

Sn	-1.07923	-1.03372	0.07339
Rb	2.22916	-2.24097	1.24749
C	-1.12442	-2.20952	-1.96270
C	-0.50246	-3.49670	-2.09449

C	-0.87229	-4.41506	-3.10629	H	0.26827	1.58520	-3.44387
H	-0.36323	-5.38664	-3.15256	Sn	1.07923	1.03372	-0.07339
C	-1.86129	-4.09972	-4.04680	Rb	-2.22916	2.24097	-1.24749
H	-2.15115	-4.81835	-4.82177	C	1.12442	2.20952	1.96270
C	-2.45035	-2.83033	-3.98564	C	0.50246	3.49670	2.09449
H	-3.19948	-2.53825	-4.73315	C	0.87229	4.41506	3.10629
C	-2.09088	-1.90223	-2.97900	H	0.36323	5.38664	3.15256
C	0.62507	-3.99612	-1.21963	C	1.86129	4.09972	4.04680
C	0.35490	-4.82174	-0.08890	H	2.15115	4.81835	4.82177
C	1.42903	-5.45577	0.57952	C	2.45035	2.83033	3.98564
H	1.22271	-6.09617	1.44475	H	3.19948	2.53825	4.73315
C	2.75575	-5.30563	0.12760	C	2.09088	1.90223	2.97900
H	3.57427	-5.83108	0.63511	C	-0.62507	3.99612	1.21963
C	3.01811	-4.50129	-0.98948	C	-0.35490	4.82174	0.08890
H	4.04851	-4.40111	-1.35239	C	-1.42903	5.45577	-0.57952
C	1.97365	-3.83339	-1.67543	H	-1.22271	6.09617	-1.44475
C	-1.08227	-5.10962	0.35509	C	-2.75575	5.30563	-0.12760
H	-1.72786	-4.36590	-0.14485	H	-3.57427	5.83108	-0.63511
C	-1.52069	-6.51950	-0.10809	C	-3.01811	4.50129	0.98948
H	-2.56731	-6.71668	0.18698	H	-4.04851	4.40111	1.35239
H	-0.88713	-7.30396	0.34512	C	-1.97365	3.83339	1.67543
H	-1.45109	-6.61688	-1.20428	C	1.08227	5.10962	-0.35509
C	-1.29169	-4.94062	1.87330	H	1.72786	4.36590	0.14485
H	-0.68187	-5.64916	2.46438	C	1.52069	6.51950	0.10809
H	-2.34985	-5.12289	2.13273	H	2.56731	6.71668	-0.18698
H	-1.04697	-3.90720	2.17680	H	0.88713	7.30396	-0.34512
C	2.30617	-3.00460	-2.91924	H	1.45109	6.61688	1.20428
H	1.38519	-2.46880	-3.20560	C	1.29169	4.94062	-1.87330
C	2.70051	-3.92158	-4.10081	H	0.68187	5.64916	-2.46438
H	3.60482	-4.51197	-3.86367	H	2.34985	5.12289	-2.13273
H	2.91509	-3.32416	-5.00559	H	1.04697	3.90720	-2.17680
H	1.88752	-4.62722	-4.34040	C	-2.30617	3.00460	2.91924
C	3.39591	-1.94106	-2.66304	H	-1.38519	2.46880	3.20560
H	3.07977	-1.21913	-1.88739	C	-2.70051	3.92158	4.10081
H	3.59259	-1.36522	-3.58495	H	-3.60482	4.51197	3.86367
H	4.35511	-2.39592	-2.35222	H	-2.91509	3.32416	5.00559
C	-2.79083	-0.56934	-3.12071	H	-1.88752	4.62722	4.34040
C	-4.08418	-0.36539	-2.55587	C	-3.39591	1.94106	2.66304
C	-4.80938	0.80356	-2.88810	H	-3.07977	1.21913	1.88739
H	-5.80649	0.95872	-2.46043	H	-3.59259	1.36522	3.58495
C	-4.28457	1.75263	-3.78857	H	-4.35511	2.39592	2.35222
H	-4.87321	2.63675	-4.06336	C	2.79083	0.56934	3.12071
C	-3.01707	1.54598	-4.34912	C	4.08418	0.36539	2.55587
H	-2.61763	2.27541	-5.06457	C	4.80938	-0.80356	2.88810
C	-2.25005	0.39867	-4.02773	H	5.80649	-0.95872	2.46043
C	-4.74041	-1.43318	-1.67569	C	4.28457	-1.75263	3.78857
H	-3.94208	-2.13266	-1.37027	H	4.87321	-2.63675	4.06336
C	-5.35455	-0.85924	-0.38312	C	3.01707	-1.54598	4.34912
H	-4.57337	-0.36261	0.21940	H	2.61763	-2.27541	5.06457
H	-5.77968	-1.67466	0.22890	C	2.25005	-0.39867	4.02773
H	-6.17097	-0.14124	-0.58640	C	4.74041	1.43318	1.67569
C	-5.79624	-2.22603	-2.48258	H	3.94208	2.13266	1.37027
H	-6.60938	-1.56474	-2.83423	C	5.35455	0.85924	0.38312
H	-6.24894	-3.01685	-1.85723	H	4.57337	0.36261	-0.21940
H	-5.34251	-2.70679	-3.36515	H	5.77968	1.67466	-0.22890
C	-0.88961	0.19650	-4.70036	H	6.17097	0.14124	0.58640
H	-0.41552	-0.67308	-4.21427	C	5.79624	2.22603	2.48258
C	-1.06200	-0.14185	-6.19970	H	6.60938	1.56474	2.83423
H	-1.67074	-1.05215	-6.33076	H	6.24894	3.01685	1.85723
H	-0.08045	-0.31274	-6.67817	H	5.34251	2.70679	3.36515
H	-1.56217	0.68266	-6.74062	C	0.88961	-0.19650	4.70036
C	0.05916	1.40036	-4.51394	H	0.41552	0.67308	4.21427
H	-0.35224	2.32591	-4.95862	C	1.06200	0.14185	6.19970
H	1.02787	1.20469	-5.00739	H	1.67074	1.05215	6.33076

H	0.08045	0.31274	6.67817
H	1.56217	-0.68266	6.74062
C	-0.05916	-1.40036	4.51394
H	0.35224	-2.32591	4.95862
H	-1.02787	-1.20469	5.00739
H	-0.26827	-1.58520	3.44387

$1^{Cs\bullet -}$

SCF (BP86/BS1) Energy = -2378.29329584
Enthalpy 0K = -2377.156680
Enthalpy 298K = -2377.080626
Free Energy 298K = -2377.276315
Lowest Frequency = 7.1443 cm^{-1}
Second Frequency = 14.7140 cm^{-1}

Sn	-1.07690	-1.05061	0.03865
Cs	2.33591	-2.33857	1.29357
C	-1.13302	-2.23952	-2.00052
C	-0.52523	-3.53413	-2.12404
C	-0.92259	-4.46662	-3.11317
H	-0.42081	-5.44223	-3.15304
C	-1.92650	-4.15978	-4.03987
H	-2.23678	-4.88850	-4.79729
C	-2.49983	-2.88320	-3.99050
H	-3.25728	-2.59471	-4.73092
C	-2.11268	-1.94097	-3.00655
C	0.61880	-4.04312	-1.27640
C	0.36984	-4.90468	-0.16743
C	1.45078	-5.58575	0.43834
H	1.25899	-6.25183	1.28773
C	2.76371	-5.45384	-0.05673
H	3.58607	-6.01724	0.40102
C	3.00648	-4.60628	-1.14608
H	4.02644	-4.51096	-1.53874
C	1.95690	-3.88551	-1.76446
C	-1.05590	-5.16704	0.32659
H	-1.70506	-4.41022	-0.14889
C	-1.54066	-6.56759	-0.11648
H	-2.58033	-6.74104	0.21559
H	-0.90941	-7.36547	0.31618
H	-1.51183	-6.66856	-1.21415
C	-1.20239	-4.99093	1.85182
H	-0.57509	-5.70307	2.42002
H	-2.25108	-5.16084	2.15467
H	-0.93707	-3.95863	2.14175
C	2.26816	-3.00696	-2.97934
H	1.34646	-2.44997	-3.21902
C	2.62397	-3.87412	-4.20938
H	3.52858	-4.48191	-4.02240
H	2.82055	-3.23913	-5.09233
H	1.79795	-4.56131	-4.45772
C	3.37450	-1.96623	-2.70070
H	3.08032	-1.26903	-1.89460
H	3.56288	-1.35738	-3.60293
H	4.33294	-2.44366	-2.42288
C	-2.80530	-0.60774	-3.17864
C	-4.11888	-0.40321	-2.66187
C	-4.85341	0.73577	-3.06467
H	-5.86511	0.89032	-2.67164
C	-4.32253	1.65348	-3.99334
H	-4.91894	2.51261	-4.32409
C	-3.03131	1.45131	-4.49927
H	-2.62125	2.15945	-5.22999
C	-2.25223	0.33850	-4.10176
C	-4.77737	-1.43580	-1.74212
H	-3.98167	-2.12865	-1.41493
C	-5.37652	-0.80644	-0.46781
H	-4.58453	-0.30313	0.11493
H	-5.81165	-1.59239	0.17508
H	-6.18105	-0.08178	-0.69402
C	-5.84441	-2.25171	-2.50918
H	-6.65818	-1.60038	-2.87772
H	-6.29486	-3.01661	-1.85092
H	-5.40122	-2.76697	-3.37752
C	-0.86455	0.13903	-4.71730
H	-0.39163	-0.70061	-4.18018
C	-0.97535	-0.26086	-6.20702
H	-1.56299	-1.18695	-6.32235
H	0.02609	-0.43232	-6.64198
H	-1.46920	0.53222	-6.79851
C	0.05240	1.36907	-4.54297
H	-0.36479	2.27077	-5.02939
H	1.03947	1.17852	-5.00075
H	0.22800	1.59278	-3.47460
Sn	1.07690	1.05061	-0.03865
Cs	-2.33591	2.33857	-1.29357
C	1.13302	2.23952	2.00052
C	0.52523	3.53413	2.12404
C	0.92259	4.46662	3.11317
H	0.42081	5.44223	3.15304
C	1.92650	4.15978	4.03987
H	2.23678	4.88850	4.79729
C	2.49983	2.88320	3.99050
H	3.25728	2.59471	4.73092
C	2.11268	1.94097	3.00655
C	-0.61880	4.04312	1.27640
C	-0.36984	4.90468	0.16743
C	-1.45078	5.58575	-0.43834
H	-1.25899	6.25183	-1.28773
C	-2.76371	5.45384	0.05673
H	-3.58607	6.01724	-0.40102
C	-3.00648	4.60628	1.14608
H	-4.02644	4.51096	1.53874
C	-1.95690	3.88551	1.76446
C	1.05590	5.16704	-0.32659
H	1.70506	4.41022	0.14889
C	1.54066	6.56759	0.11648
H	2.58033	6.74104	-0.21559
H	0.90941	7.36547	-0.31618
H	1.51183	6.66856	1.21415
C	1.20239	4.99093	-1.85182
H	0.57509	5.70307	-2.42002
H	2.25108	5.16084	-2.15467
H	0.93707	3.95863	-2.14175
C	-2.26816	3.00696	2.97934
H	-1.34646	2.44997	3.21902
C	-2.62397	3.87412	4.20938
H	-3.52858	4.48191	4.02240
H	-2.82055	3.23913	5.09233
H	-1.79795	4.56131	4.45772
C	-3.37450	1.96623	2.70070
H	-3.08032	1.26903	1.89460
H	-3.56288	1.35738	3.60293
H	-4.33294	2.44366	2.42288
C	2.80530	0.60774	3.17864
C	4.11888	0.40321	2.66187
C	4.85341	-0.73577	3.06467
H	5.86511	-0.89032	2.67164

C	4.32253	-1.65348	3.99334
H	4.91894	-2.51261	4.32409
C	3.03131	-1.45131	4.49927
H	2.62125	-2.15945	5.22999
C	2.25223	-0.33850	4.10176
C	4.77737	1.43580	1.74212
H	3.98167	2.12865	1.41493
C	5.37652	0.80644	0.46781
H	4.58453	0.30313	-0.11493
H	5.81165	1.59239	-0.17508
H	6.18105	0.08178	0.69402
C	5.84441	2.25171	2.50918
H	6.65818	1.60038	2.87772
H	6.29486	3.01661	1.85092

H	5.40122	2.76697	3.37752
C	0.86455	-0.13903	4.71730
H	0.39163	0.70061	4.18018
C	0.97535	0.26086	6.20702
H	1.56299	1.18695	6.32235
H	-0.02609	0.43232	6.64198
H	1.46920	-0.53222	6.79851
C	-0.05240	-1.36907	4.54297
H	0.36479	-2.27077	5.02939
H	-1.03947	-1.17852	5.00075
H	-0.22800	-1.59278	3.47460

References

1. L. E. English, R. A. Jackson, N. J. Evans, D. J. Babula, H. J. Draper, S. R. Brown, J. Fletcher, D. J. Liptrot and K. G. Pearce, *RSC Mechanochem.*, 2025, **2**¸503-506.
2. L. Pu, A. D. Phillips, A. F. Richards, M. Stender, R. S. Simons, M. M. Olmstead and P. P. Power, *J. Am. Chem. Soc.*, 2003, **125**, 11626-11636.
3. O. V. Dolomanov, L. J. Bourhis, R. J. Gildea, J. A. K. Howard and H. Puschmann, J. Appl. Cryst. 2009, 42, 339-341
4. G. M. Sheldrick, Acta Cryst. 2015, A71, 3-8.
5. G. M. Sheldrick, Acta Cryst. 2015, C71, 3-8.
6. K. G. Pearce, H. -Y. Liu, S. E. Neale, H. M. Goff, M. F. Mahon, C. L. McMullin and M. S. Hill, *Nat. Commun.* 2023, **14**, 8147-8152.
7. K. G. Pearce, A. Morales, M. S. Hill and C. L. McMullin, *Chem. Eur. J.* **2025**, **0**, e202502197
8. M. J. Frisch, G. W. Trucks, H. B. Schlegel, G. E. Scuseria, M. A. Robb, J. R. Cheeseman, G. Scalmani, V. Barone, G. A. Petersson, H. Nakatsuji, X. Li, M. Caricato, A. V. Marenich, J. Bloino, B. G. Janesko, R. Gomperts, B. Mennucci, H. P. Hratchian, J. V. Ortiz, A. F. Izmaylov, J. L. Sonnenberg, Williams, F. Ding, F. Lipparini, F. Egidi, J. Goings, B. Peng, A. Petrone, T. Henderson, D. Ranasinghe, V. G. Zakrzewski, J. Gao, N. Rega, G. Zheng, W. Liang, M. Hada, M. Ehara, K. Toyota, R. Fukuda, J. Hasegawa, M. Ishida, T. Nakajima, Y. Honda, O. Kitao, H. Nakai, T. Vreven, K. Throssell, J. A. Montgomery Jr., J. E. Peralta, F. Ogliaro, M. J. Bearpark, J. J. Heyd, E. N. Brothers, K. N. Kudin, V. N. Staroverov, T. A. Keith, R. Kobayashi, J. Normand, K. Raghavachari, A. P. Rendell, J. C. Burant, S. S. Iyengar, J. Tomasi, M. Cossi, J. M. Millam, M. Klene, C. Adamo, R. Cammi, J. W. Ochterski, R. L. Martin, K. Morokuma, O. Farkas, J. B. Foresman, D. J. Fox, Wallingford, CT, **2016**.
9. a) A. D. Becke, *Phys. Rev. A* 1988, **38**, 3098-3100; b) J. P. Perdew, *Phys. Rev. B* 1986, **33**, 8822-8824. c) V. N. Staroverov, G. E. Scuseria, J. Tao, J. P. Perdew, *J. Chem. Phys.* 2003, **119**, 12129.
10. Y. Zhao and D. G. Truhlar, *Theor. Chem. Acc.* 2008, **120**, 215–241.
11. a) S. Grimme, S. Ehrlich and L. Goerigk, *J. Comp. Chem.* 2011, **32**, 1456-1465. b) S. Grimme, J. Antony, S. Ehrlich and H. A. Krieg, *J. Chem. Phys.* 2010, **132**, 154104.
12. J. Tomasi, B. Mennucci and R. Cammi, *Chem. Rev.* 2005, **105**, 2999-3094.
13. D. Andrae, U. Häußermann, M. Dolg, H. Stoll and H. Preuß, *Theor. Chim. Acta* 1990, **77**, 123-141.
14. a) P. C. Hariharan and J. A. Pople, *Theor. Chim. Acta* 1973, **28**, 213-222; b) W. J. Hehre, R. Ditchfield and J. A. Pople, *J. Chem. Phys.* 1972, **56**, 2257-2261.
15. A. Höllwarth, M. Böhme, S. Dapprich, A. W. Ehlers, A. Gobbi, V. Jonas, K. F. Köhler, R. Stegmann, A. Veldkamp and G. Frenking, *Chem. Phys. Lett.* 1993, **208**, 237.
16. a) R. Krishnan; J. S. Binkley; R. Seeger and J. A. Pople, *J. Chem. Phys.* 1980, **72**, 650-654; b) A. D. McLean and G. S. J. Chandler, *Chem. Phys.* 1980, **72**, 5639-5648; c) M. M. Francl, W. J Pietro; W. J. Hehre, J. S. Binkley, M. S. Gordon, D. J. DeFrees and J. A. Pople, *J. Chem. Phys.*1982 **77**, 3654-3665; d) T. Clark, J. Chandrasekhar, G. W. Spitznagel and P. v. R. Schleyer, J. *Comput. Chem.* 1983, **4**, 294-301; e) G. W. Spitznagel, T. Clark, P. v. R. Schleyer and J. W. Hehre, *J. Comput. Chem.* 1987, **8**, 1109-1116. f) J.- M. Blaudeau, M. P. McGrath, L. A. Curtiss; L. Radom, *J. Chem. Phys.* 1997, **107**, 5016-5021.
17. a) T. Leininger, A. Nicklass, W. Küchle, H. Stoll, M. Dolg and A. Bergner, *Chem. Phys. Lett.* 1996, **255**, 274-280; b) F. Weigend and R. Ahlrichs, *Phys. Chem. Chem. Phys.* 2005 **7**, 3297.
18. a) D. A. Pantazis, X.-Y. Chen, C. R. Landis and F. Neese, *J. Chem. Theory Comput.* 2008, **4**, 908; b) M. Buhl, C. Reimann, D. A. Pantazis; T. Bredow and F. Neese, *J. Chem. Theory Comput.* 2008, **4**, 1449; c) D. A Pantazis and F. Neese, *J. Chem. Theory Comput.* 2009, **5**, 2229; d) D. A. Pantazis and F. Neese, *J. Chem. Theory Comput.* 2011, **7**, 677; e) D. A. Pantazis and F. Neese, *Theor. Chem. Acc.* 2012, **131**, 1292; f) J. D. Rolfes, F. Neese and D. A. Pantazis, *J. Comput. Chem.* 2020, **41**, 1842.

19. F. Neese, *IREs Comput Mol Sci*. 2022; **12**:e1606.
20. *AIMAll* (Version 19.10.12), T. A. Keith, TK Gristmill Software, Overland ParkKS, USA, **2019**.
21. a) B. Metz, H. Stoll and M. Dolg, *J. Chem. Phys.* 2000, **113**, 2563–2569; b) K. A. Peterson, *J. Chem. Phys.*2003, **119**, 11099–11112.
22. NBO 7.0, E. D. Glendening, J. K. Badenhoop, A. E. Reed, J. E. Carpenter,J. A. Bohmann, C. M. Morales, P. Karafiloglou, C. R. Landis, F. Weinhold,Theoretical Chemistry Institute, University of Wisconsin, Madison, USA,**2003**.
23. Zhurko G. A. Chemcraft - graphical program for visualization of quantum chemistry computations. Ivanovo, Russia, **2005**. https://chemcraftprog.com
24. GaussView, Version 6.1, Roy Dennington, Todd A. Keith, and John M. Millam, Semichem Inc., Shawnee Mission, KS, **2016**.
25. F**.** Neese, *Comput. Mol. Sci.* 2025, **15**, *2*, e70019.
26. F. Neese, *J. Chem. Phys.* 2005, **122**, 034107.
27. F. Neese, F. Wennmohs, A. Hansen, U. Becker, *Chem. Phys.* 2009, **356**, 98.
28. P. Pollak, F. Weigend, *J. Chem. Theory Comput.* 2017, **13**, 3696.
29. Y. J. Franzke, J. M. Yu., *J. Chem. Theory Comput.* 2022, **18**, 323.
30. B. Miehlich, A. Savin, H. Stoll, H. Preuss, *Chem. Phys. Lett* 1989, **157**, 200–206.
31. C. Adamo, V. Barone, *J. Chem. Phys.* 1999, **110**, 6158.
32. P. J. Stephens, F. J. Devlin, C. F. Chabalowski, M. J. Frisch, *J. Phys. Chem.* 1994, **98**, 11623.
33. A. Najibi, L. Goerigk, *J. Comput. Chem.* 2020, **41**, 2562.
34. T. Yanai, D. P. Tew, N. C. Handy, *Chem. Phys. Lett.* 2004, **393**, 51.
35. S. Stoll, A. Schweiger, *J. Magn. Reson.* 2006. **178**(1), 42-55.
